# Exploring Cancer Survivor Needs and Preferences for Communicating Personalized Cancer Statistics From Registry Data: Qualitative Multimethod Study

**DOI:** 10.2196/25659

**Published:** 2021-10-25

**Authors:** Ruben D Vromans, Mies C van Eenbergen, Gijs Geleijnse, Steffen Pauws, Lonneke V van de Poll-Franse, Emiel J Krahmer

**Affiliations:** 1 Department of Communication and Cognition Tilburg University Tilburg Netherlands; 2 Department of Research and Development Netherlands Comprehensive Cancer Organisation Utrecht Netherlands; 3 Department of Remote Patient Management and Chronic Care Philips Research Eindhoven Netherlands; 4 Division of Psychosocial Research & Epidemiology The Netherlands Cancer Institute Amsterdam Netherlands; 5 Department of Medical and Clinical Psychology Tilburg University Tilburg Netherlands

**Keywords:** breast cancer, cancer statistics, personalization, prostate cancer, risk communication, cancer registry, cancer, patient needs and preferences

## Abstract

**Background:**

Disclosure of cancer statistics (eg, survival or incidence rates) based on a representative group of patients can help increase cancer survivors’ understanding of their own diagnostic and prognostic situation, and care planning. More recently, there has been an increasing interest in the use of cancer registry data for disclosing and communicating personalized cancer statistics (tailored toward personal and clinical characteristics) to cancer survivors and relatives.

**Objective:**

The aim of this study was to explore breast cancer (BCa) and prostate cancer (PCa) survivor needs and preferences for disclosing (what) and presenting (how) personalized statistics from a large Dutch population-based data set, the Netherlands Cancer Registry (NCR).

**Methods:**

To elicit survivor needs and preferences for communicating personalized NCR statistics, we created different (non)interactive tools visualizing hypothetical scenarios and adopted a qualitative multimethod study design. We first conducted 2 focus groups (study 1; n=13) for collecting group data on BCa and PCa survivor needs and preferences, using noninteractive sketches of what a tool for communicating personalized statistics might look like. Based on these insights, we designed a revised interactive tool, which was used to further explore the needs and preferences of another group of cancer survivors during individual think-aloud observations and semistructured interviews (study 2; n=11). All sessions were audio-recorded, transcribed verbatim, analyzed using thematic (focus groups) and content analysis (think-aloud observations), and reported in compliance with qualitative research reporting criteria.

**Results:**

In both studies, cancer survivors expressed the need to receive personalized statistics from a representative source, with especially a need for survival and conditional survival rates (ie, survival rate for those who have already survived for a certain period). Personalized statistics adjusted toward personal and clinical factors were deemed more relevant and useful to know than generic or average-based statistics. Participants also needed support for correctly interpreting the personalized statistics and putting them into perspective, for instance by adding contextual or comparative information. Furthermore, while thinking aloud, participants experienced a mix of positive (sense of hope) and negative emotions (feelings of distress) while viewing the personalized survival data. Overall, participants preferred simplicity and conciseness, and the ability to tailor the type of visualization and amount of (detailed) statistical information.

**Conclusions:**

The majority of our sample of cancer survivors wanted to receive personalized statistics from the NCR. Given the variation in patient needs and preferences for presenting personalized statistics, designers of similar information tools may consider potential tailoring strategies on multiple levels, as well as effective ways for providing supporting information to make sure that the personalized statistics are properly understood. This is encouraging for cancer registries to address this unmet need, but also for those who are developing or implementing personalized data-driven information tools for patients and relatives.

## Introduction

### Background

In cancer care, many newly diagnosed patients and survivors prefer disclosure of cancer statistics and prognostic information [1-4]. For instance, patients may wish to receive information about the chances of surviving the disease (survival data), whereas others are in need of knowing the exact number of people who are diagnosed with the same type of cancer (incidence data). Such cancer statistics are increasingly being presented on the internet through various sources, such as general cancer websites for both patients and relatives [5] and health care professionals [6], but also in decision-support tools such as patient decision aids [7] or publicly available prediction models [8]. Cancer statistics may help increase patients’ understanding of their own diagnosis, prognosis, and involvement in different stages of the shared decision-making process (eg, option talk stage) with their clinician [9,10]. Moreover, both patients and clinicians may use cancer statistics to start a conversation about complex health topics such as survival or cancer recurrence, and to discuss its role in making a decision about treatment [11]. It is therefore important that patients, relatives, and clinicians have access to representative and reliable cancer statistics about topics that could contribute to informed decision making and advance care planning.

However, current cancer statistics are typically generic and population based [12-14], thereby making it hard for patients to apply the numbers to their own individual situation [15]. For instance, when a man of 50 years old is diagnosed with prostate cancer (PCa) and is asking about his life expectancy, population-based statistics about survival (which will mostly be based on substantially older men) may be of limited value. In light of the strong movements toward personalized health care [16], patient-centered care, and open access of “big health data,” [17,18] there has been an increasing interest in the use of population-based cancer registries for disclosing *personalized cancer statistics* to survivors and relatives [19]. This allows survivors to be provided with more specific statistical information of certain health outcomes by comparing their own characteristics (eg, age, gender, type of tumor, tumor stage) with specific patient groups with similar characteristics. An illustrative example of this is the American Surveillance, Epidemiology, and End Results Cancer Survival Calculator (SEER*CSC) [11], which draws on an extensive cancer statistics database for communicating personalized cancer statistics (cancer incidence, survival rates) in multiple formats to patients via a publicly available web-based tool. Other initiatives that used registry data or other patient-reported data in patient–clinician communication are decision-support tools for estimating personalized health statistics, such as treatment (side) effects or quality of life outcomes [8,20,21]. Given these developments, the question arises, then, what the needs and preferences for communicating personalized cancer statistics are among cancer survivors.

### Present Study and Objectives

In this study, we focus on the disclosure of personalized cancer statistics from the Netherlands Cancer Registry (NCR), a Dutch nationwide population-based registry maintained by the Netherlands Comprehensive Cancer Organisation (IKNL). The NCR records all new cancer diagnoses and contains information about diagnosis (eg, tumor characteristics), sociodemographic (eg, age, gender), treatment, and vital status of millions of patients with cancer in the Netherlands since 1989 [22], and primarily enables health care professionals, policy makers, and others to reflect on and improve cancer care and prevention in the Netherlands. Basic and generic NCR statistics such data on incidence and survival are already being provided through websites of patient organizations, hospitals, and online cancer communities (all aimed at cancer survivors and their relatives), with more detailed NCR statistics according to site, gender, age, and region being available through the web-based tool NKR-Cijfers [6] (aimed at health care professionals). Our main project goal is to explore whether important NCR statistics on incidence, survival, and conditional survival could be disclosed via a web-based interactive tool, in which visitors (eg, patients or relatives) will have the opportunity to enter certain personal (eg, age, gender) and clinical characteristic (eg, tumor stage, years since diagnosis), with the aim of receiving personalized statistical information based on real-life patient data with similar characteristics. However, this development raises a number of questions. What types of personalized cancer statistics do cancer survivors want to receive? How should these personalized statistics be presented to patients? What potential barriers or challenges are involved in communicating personalized survival statistics to survivors via a public website? Answers to these questions will not only be useful for the development of a real-life web-based tool for displaying personalized statistics from the NCR to cancer survivors, but also for research groups outside the oncology context working on the design and implementation of similar statistical information tools based on registry or other medical data for patients and relatives.

The purpose of this study is therefore to explore the needs and preferences of breast cancer (BCa) and PCa survivors for communicating personalized cancer statistics from the NCR. Although previous research has shown that most (but not all) patients want to receive prognostic information [1-4,23], it is unclear which pieces of prognostic and statistical information patients wish to receive. Therefore, we first aim to explore patients’ need for prognostic information on a deeper level, and more specifically by investigating *what* type of personalized cancer risks, statistics, and probabilities patients need to receive from the NCR and other data sources. Furthermore, it is much more difficult for survivors and relatives than for health care professionals to translate group-based statistics to their personal situation [24,25]. For instance, some individuals have inherently more difficulties than others in understanding numeric information, even when supported with visual aids, whereas others are experiencing emotions while processing sensitive health data such as survival or mortality rates. Hence, our second aim is to examine *how* patients want to receive personalized statistics from the NCR. To achieve our aims, we designed different (non)interactive tools to probe participant responses on their needs and preferences.

## Methods

### Overview

**Figure 1:**
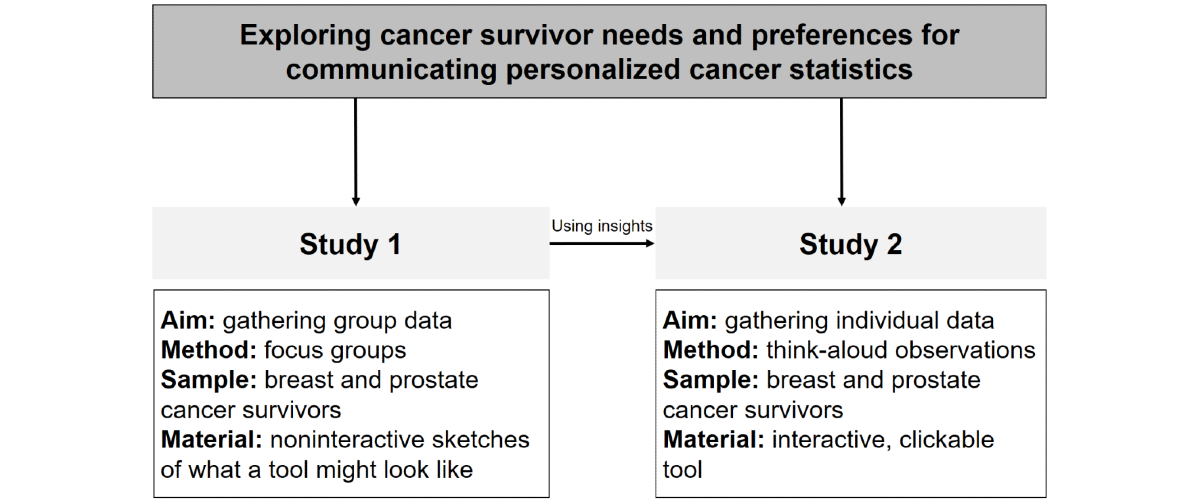
Overview of studies.

We conducted a multimethod qualitative study among BCa and PCa survivors (Figure 1). BCa and PCa are among the most prevalent types of cancer among men and women, respectively, which also makes it feasible to calculate personalized statistics based on a subgroup of patient data that is sizeable enough to provide statistically sound and meaningful information. Moreover, in general, the prognostic outcomes are relatively favorable for these 2 cancer types, thereby making it a suitable starting point for our initiative for disclosing personalized cancer statistics. We first conducted 2 focus groups (study 1) for collecting group data on needs and preferences of BCa and PCa survivors for communicating personalized NCR data, using noninteractive sketches of what a tool for communicating personalized statistics might look like. Based on these insights, we designed a revised interactive version of the tool, which was used to further explore the needs and preferences of another group of BCa and PCa survivors during individual think-aloud observations and semistructured interviews (study 2). We complied with the 32-item Consolidated Criteria for Reporting Qualitative Research (Multimedia Appendix 1) [26]. Ethical approval was granted by the Research Ethics and Data Management Committee of the Tilburg School of Humanities and Digital Sciences of Tilburg University (REDC 2019-44).

### Study 1: Focus Groups

#### Overview

To explore cancer survivor needs and preferences for communicating personalized statistics from the NCR, this first study employed 2 separate focus groups (1 with BCa survivors and 1 with PCa survivors). Focus group methodology is particularly useful for exploring people’s perceptions, beliefs, opinions, and attitudes about a certain topic [27].

#### Sampling and Recruitment

For the BCa focus group, female participants were recruited from the Dutch Breast Cancer Patient Association (Borstkankervereniging Nederland [BVN]); for the PCa focus group, male participants were identified from the Dutch Prostate Cancer Foundation (Prostaatkankerstichting [PKS]). Participants were included if they were diagnosed with BCa or PCa in the past (at least 1 year after diagnosis). Each eligible participant was approached by email by one of the representatives of the BVN or PKS. Members of our research team did not have any prior relationship with the participants at study commencement, and we were unaware of who from the patient organizations were approached to participate in the focus groups. Participants were reimbursed for their time with a €15 (US $17.4) gift card (unannounced).

#### Materials

To elicit patients’ needs and preferences, we designed noninteractive sketches of what a tool for calculating personalized statistics from the NCR might look like (Multimedia Appendix 2). This tool consisted of 3 parts: (1) patient data entry, (2) tumor data entry, and (3) output display. The patient data entry part was the same for both cancer groups (eg, gender, year of birth), but the tumor data entry part differed between the 2 versions. The PCa version contained items such as year of diagnosis, prostate-specific antigen value, Gleason score (ie, the aggressiveness of the cancer), and tumor stage (ie, where the cancer is present in the body). The BCa version contained items such as year of diagnosis, tumor stage, and—in case tumor stage was unknown—metastases (ie, whether the cancer has spread beyond the breast and nearby lymph nodes to other parts of the body). The output display showed a summary of the patient and tumor characteristics filled out by the patient, followed by the personalized absolute incidence rate of their year of diagnosis, the 5- and 10-year overall survival rate, and the conditional survival rate (ie, survival rate for those who have already survived for a certain period [28]). All statistics were shown numerically, and the survival statistics were also shown visually in 4 different, conventional ways (ie, icon array, pie chart, bar chart, and line graphs). Participants could also switch between the 4 types of visualization.

#### Data Collection

We used a semistructured topic guide for both focus groups to facilitate discussion and elicit participants’ needs and preferences for the disclosure and presentation of personalized statistics from NCR data. After a round of introduction, we first explained the purpose of the project and the NCR to the participants. We then asked them to what extent they were in need of receiving the (NCR) statistics incidence, survival, and conditional survival rates in a personalized way, either at their time of diagnosis or at a later moment. After this, we posed a final question by asking what other personalized statistics they were interested in after diagnosis and treatment. During the second part of the discussion, we showed participants sketches of what such a tool could look like (Multimedia Appendix 2). Participants were asked to take a critical look at each slide and provide comments about the tool. They were also encouraged to express their needs and preferences regarding the information presented in the data entry part and the output display of the tool.

The PCa focus group was moderated by RV (male, PhD-candidate, risk communication scientist), MvE (female, health communication scientist with expertise in qualitative research), and GG (male, PhD, with expertise in clinical data science), and the BCa focus group by RV and MvE. The moderators were not known to the participants. Both focus groups lasted 90 minutes and were conducted at the IKNL in Utrecht (The Netherlands) in November 2018 (PCa focus group) and March 2019 (BCa focus group). Field notes were taken in each focus group by RV.

#### Data Analysis

Qualitative data obtained from the focus groups were audio-recorded (with permission of the participants), transcribed verbatim, and analyzed thematically [29]. For this, we developed a deductive coding scheme based on the study objectives, discussion guide, and focus group content. First, 2 investigators (RV and MvE) developed a preliminary conceptual schema and codebook by independently reading the focus group transcripts. The codebook was designed to capture broad coding categories of needs and preferences for (1) disclosing different types of personalized statistics, and (2) presenting personalized statistics. Then, both investigators independently coded each transcript using MAXQDA 2020 (Verbi Software) [30], and disagreements were resolved through discussion. Finally, both investigators jointly generated a report from the coded transcripts by format to identify themes. Quotes for supporting (sub)themes were translated into English.

### Study 2: Think-Aloud Observations

#### Overview

A think-aloud methodology was used to further assess the needs and preferences of another group of cancer survivors for communicating personalized statistics from the NCR. This involved asking participants to verbalize their thoughts, impressions, and feelings while working with a revised, clickable, and interactive version of the tool to calculate personalized cancer statistics [31]. These revisions were based on input from cancer survivors participating in the focus group (study 1). Semistructured interview techniques were used to allow participants to elaborate on their statements and experience with the tool, and to put them into context. The semistructured interviews also allowed us to capture participant preferences for a specific presentation format in case the think-aloud observations would not cover this information [32].

#### Sampling and Recruitment

Eligible participants were recruited from the same 2 patient organizations (BVN and PKS) as the first focus groups, and from a Dutch online cancer community (Kanker.nl [33]). Participants were included if they (1) were diagnosed with BCa or PCa in the past (at least 1 year after diagnosis), and (2) had not participated in the focus groups before. The recruitment procedure was identical to the focus groups, meaning that the members of our research team did not have any prior relationship with the participants at study commencement, and we were unaware of who from the patient organization or online cancer community were approached to participate in the think-aloud observations. Participants were reimbursed for their time with a €15 (US $17.4) gift card (unannounced).

#### Materials

**Figure 2:**
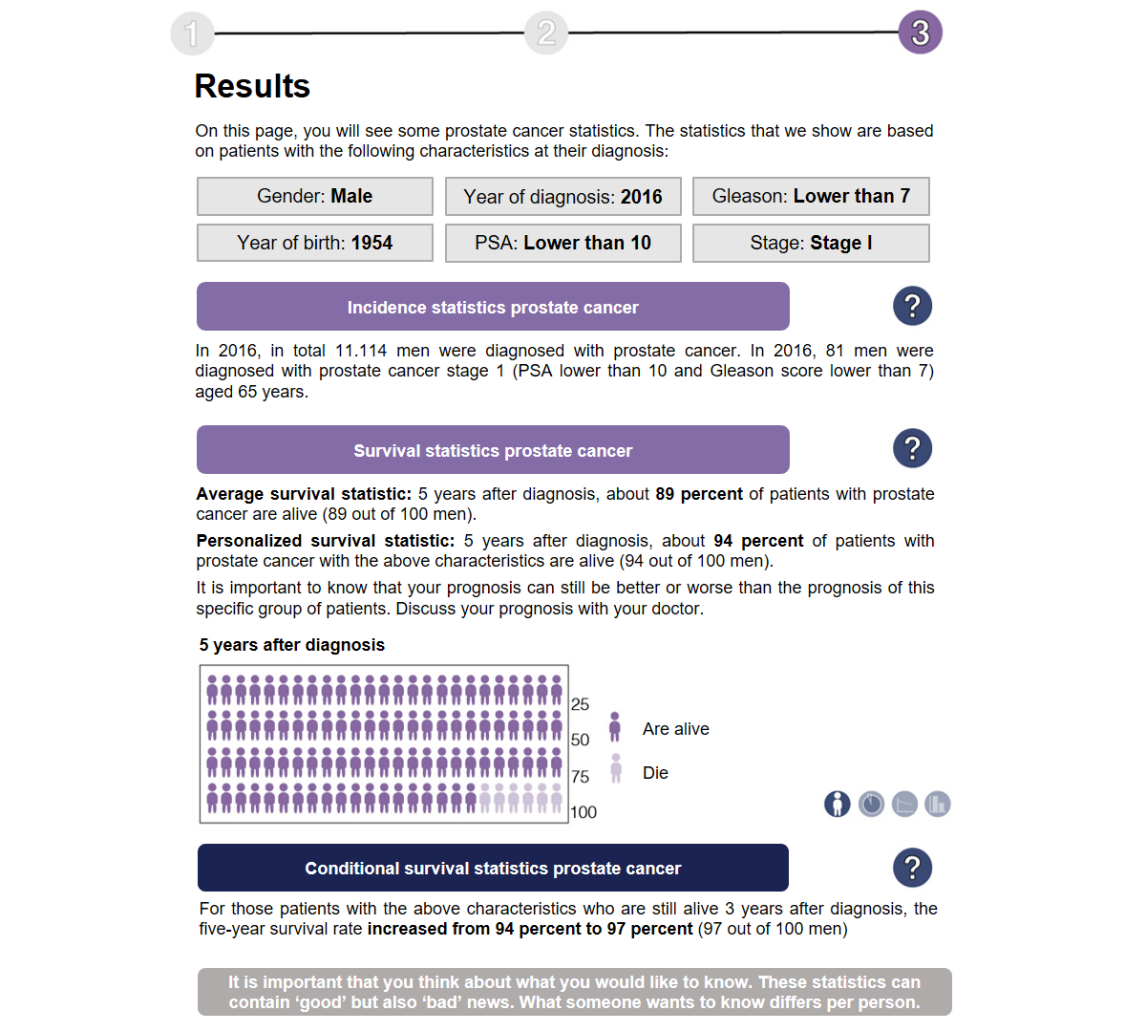
Example of the output display (translated to English) in the short (concise) version of the interactive tool, communicating a favorable survival rate to PCa survivors. All information is presented at the same time. PCa: prostate cancer.

**Figure 3:**
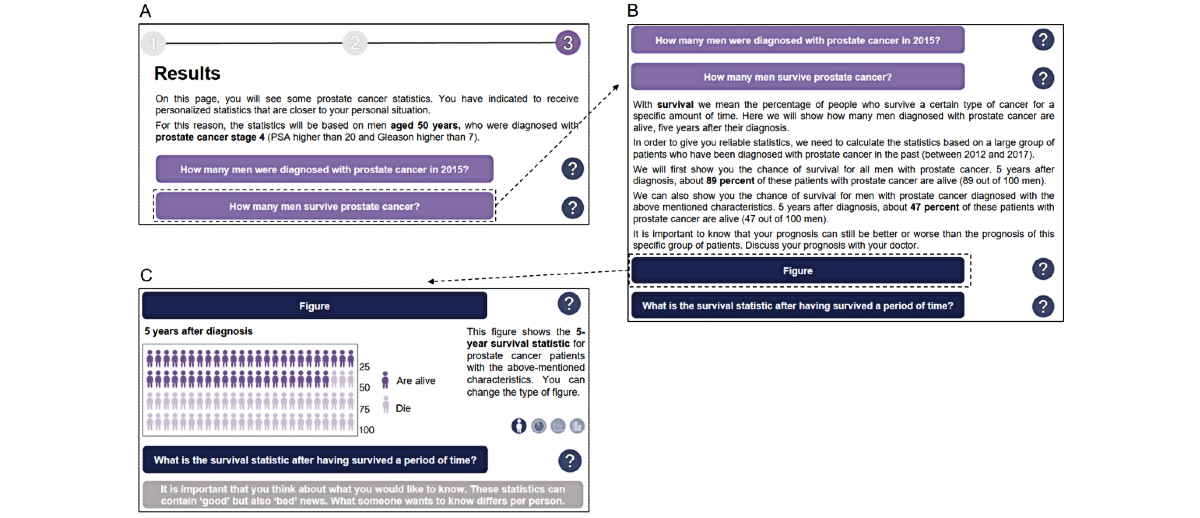
Example of the output display in the long (detailed) version of the interactive tool, communicating a less favorable survival rate to PCa survivors. Participants started at the left top figure (A), and could decide what type of information they wished to see (B, C). PCa: prostate cancer.

We designed a clickable interactive version of the tool (for screenshots, see Multimedia Appendix 3), which allowed participants to manually enter patient and tumor characteristics, to view the associated personalized statistics, and to modify the type of visualization (ie, icon array [as a default option], pie chart, bar chart, and line graphs) according to their preference. Based on the input from cancer survivors during the focus groups on the sketches of the tool, the following revisions were made. First, the interactive tool now started with a supporting page, including statements such as that the statistics may contain good or bad news (taking emotional aspects into account), that the statistics were based on prior patients (taking contextual information into account), and that we could not provide exact estimates for each individual patient (taking uncertainty into account). Second, the data entry part contained explanations in plain language about certain tumor characteristics (eg, Gleason score or tumor stage). Third, the output display was kept the same, except that we now included comparative information by providing both generic, population-based survival statistics and the personalized survival statistics altogether. Fourth, and finally, to take the survivors’ preference of amount of information into account, we created 2 tool versions: (1) a short, concise version and (2) a long, detailed version. The short version only provided the raw statistics and the minimally required explanation of the statistics on the output display, which was all presented simultaneously (Figure 2). The long version contained more textual information and gave users the option to expand texts when supplementary information was needed or to see information visually (Figure 3). All screens of the interactive tool were created using Adobe Illustrator CS6, and the tool was developed and implemented using InVision, a digital product design platform [34].

#### Data Collection

Each session started with an explanation of the procedure, signing informed consent, and a questionnaire that assessed sociodemographic information (age, gender, education, work, marital status, and children) and disease-related information (year of diagnosis, type of cancer). Participants were then instructed on how to think aloud. Participants were then asked to enter information into the tool and to view the results using 2 hypothetical case examples: (1) a patient with a favorable 5-year overall survival rate (89% for the BCa group and 94% for the PCa group), and (2) a patient with a less favorable overall 5-year survival rate (38% for participants with BCa and 47% for participants with PCa). Participants with PCa history would use a PCa case, and participants with BCa history would be presented with a BCa case. The case examples contained patient and disease-related information about 2 hypothetical patients [11]. We informed them that this may evoke some unpleasant memories/thoughts related to participants’ own cancer (diagnostic) situation. Therefore, participants were told that (1) they always have the opportunity to withdraw their participation whenever they want to, without any negative consequences, and without providing any explanation; (2) the hypothetical personalized statistics used in this study were not real. In addition, because participants might feel anxious about reflecting on their diagnostic situation, they were referred to an online expert therapist of Kanker.nl who is specialized in dealing with cancer-related anxiety.

One case example was performed using the short version of the tool, and the other with the long version of the tool. The order and combination of the tool version with the case scenario were randomized and counterbalanced across participants. While entering the information and viewing the statistics, participants were instructed to think aloud. Prompts were used when participants fell silent (eg, “Keep talking?”), and reassuring sounds were made to enhance thinking aloud (eg, “Uhuh”) [35].

After the think-aloud session, we conducted a semistructured interview to provide participants with the opportunity to elaborate on statements made during the think-aloud sessions, and to further capture participants’ preferences for communicating the statistics. For this, we used a semistructured topic guide (Multimedia Appendix 4). At the end of the sessions, participants were debriefed and informed about the full purpose of the study.

The think-aloud sessions and semistructured interviews were led by 2 interviewers, RV and a research assistant (female, research assistant in communication science with expertise in new media design). Both interviewers were not known to the participants. The sessions lasted between 21 and 67 minutes (average duration 44 minutes), and were performed at either the IKNL (in Amsterdam, Rotterdam, Utrecht, or Eindhoven) or at the participants’ home. Data were collected in April and May 2019. Field notes were taken from each session by RV.

#### Data Analysis

All think-aloud sessions and semistructured interviews were audio-recorded (with permission of the participants), transcribed verbatim, and analyzed using content analysis [36]. For this, 2 investigators (RV and MvE) developed a deductive coding scheme based on the interview guide (Multimedia Appendix 3) and the themes and subthemes that emerged from the thematic analysis of the focus group study. The same investigators then independently coded 4 transcripts, and resolved disagreements through discussion. The remaining 7 transcripts were then coded by RV. All coding activities were performed using MAXQDA 2020 (Verbi Software) [30]. Quotes for supporting the findings were translated into English.

## Results

### Patient Characteristics

Characteristics of participants in the 2 focus groups (n for the BCa group=9 females; n for the PCa group=4 males) and 11 think-aloud sessions (n for the patients with BCa=7 females; n for the patients with PCa=4 males) are summarized in Table 1. In both groups, there were more BCa survivors than PCa survivors (69% and 64%, respectively). The participants in both groups were comparable in terms of sociodemographic and disease-related characteristics (all *P* values >.20), except for the distribution of year since diagnosis (*P*=.033), with more recently diagnosed survivors in the think-aloud group.

**Table 1:** Participant characteristics for the focus groups and think-aloud sessions.

Characteristics			Study 1: Focus groups (n=13)		Study 2: Think-aloud observations (n=11)
**Gender, n**					
	Female	9		7	
	Male	4		4	
**Age (years) at time of study, mean (SD)**			59.8 (10.9)		57.1 (10.3)
	<50	3		2	
	50-65	6		6	
	>65	4		3	
**Education, n**					
	Secondary education or practical education	2		4	
	College or applied university	6		4	
	University	5		3	
**Type of cancer, n**					
	Breast cancer	9		7	
	Prostate cancer	4		4	
**Year since diagnosis, median**			9		4
	0-5	4		7	
	6-10	3		4	
	>10	6		0	
**Work situation, n**					
	Work	4		5	
	Ill (insurance)	2		0	
	No work/retired	7		6	
**Marital status, n**					
	Married/partner	10		6	
	No partner	3		5	
**Children, n**					
	No	3		4	
	Yes, living with	4		2	
	Yes, living somewhere else	6		5	

### Study 1: Focus Groups

#### Themes Identified

**Figure 4:**
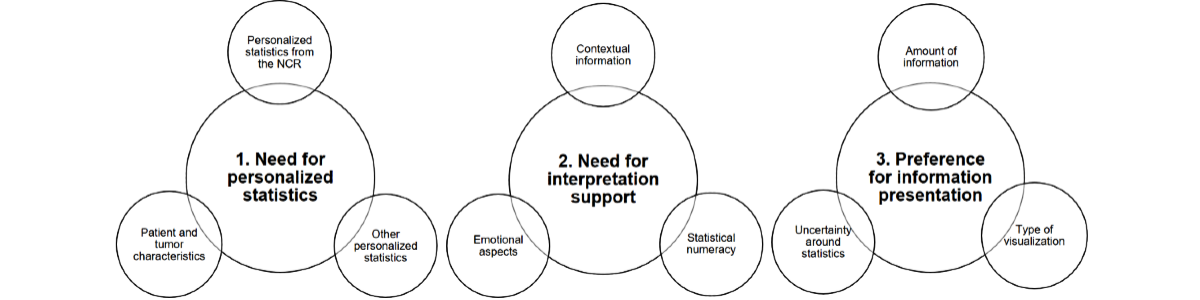
Schematic representation of themes and sub-themes identified from the focus group data. NCR: Netherlands Cancer Registry.

Three themes were identified from the focus group data (Figure 4): (1) the need for personalized statistics, (2) the need for interpretation support, and (3) preference for information presentation. Subthemes are introduced below within each of the main themes’ sections.

#### Theme 1: Need for Personalized Statistics

##### Summary

Participants reported the needs for receiving personalized statistics from the NCR as well as other personalized statistics, and also on how to establish this by taking several patient and tumor characteristics into account.

##### Personalized Statistics From the NCR

All participants found the (5- and 10-year) survival rate the most important statistic from the NCR. However, at their time of diagnosis, participants wanted to know their personalized survival chance based on their own situation. Participants mentioned that a personalized survival rate seems more relevant and useful to know than the generic or average survival rate, and that characteristics such as tumor stage and lymph nodes involvement could have a significant impact on survival rates.

You really want to know your personalized survival chances for your own type of cancer. So, if you are having a T4-stage cancer, you want to know the survival rate for that specific situation.

P04, aged 71 years

For the personalized incidence rate, participants found this type of information to be important, especially because this may help them know how many other patients like them have this specific disease and whether it is something rare or not. Being aware of the high or low incidence rate could also “help patients to see where they are in the bigger picture” [P04]. However, there were also participants who did not really see the added value of this statistic, especially because they already had been diagnosed with cancer and cannot really change this diagnosis.

You have already been diagnosed with breast cancer. So, what does it matter that other people also have breast cancer?

B04, aged 55 years

Finally, when showing personalized conditional survival rates, participants with BCa and PCa both initially found the term difficult to understand and rather confusing. However, after explaining the concept in more detail and showing them what it might look like in the tool, participants agreed that this type of statistical information might be useful to communicate. Participants mentioned that communicating the personalized conditional survival statistic “can be very reassuring and psychologically beneficial for patients” [P3]. Another participant said:

For instance, in the case of triple-negative for breast cancer, after having survived the first three years, your survival chance increases enormously! This could be very interesting and important to communicate [to patients].

B03, aged 57 years

##### Other Personalized Statistics

Participants’ need for disclosing other personalized statistics based on NCR or other data sets spanned a broad range. Participants expressed a need for receiving information about personalized risks of treatment outcomes, such as the likelihood of experiencing treatment side effects.

I would have liked to know my [personalized] risk of experiencing a side effect after treatment, and whether this risk would change over time or not.

P01, aged 72 years

Moreover, participants reported the need for personalized statistical information about cancer recurrence, risk of cancer in the family, and impact on quality of life such as physical, cognitive, and psychosocial functioning. Furthermore, participants with BCa in particular wanted to receive statistics on the chances of getting metastatic cancer, whereas participants with PCa specifically expressed a need for treatments chosen by other patients with PCa over time and performance statistics of different hospitals.

##### Patient and Tumor Characteristics

Participants had several comments on the characteristics that patients should fill out, and simultaneously expressed their need for extending this with other patient and tumor features. In both groups, participants voiced concerns about asking for a patient’s tumor stage, because most of the participants were unfamiliar with the term.

Based on my education materials from 2012, I can see that I received information about tumor grade and HER2, but not about my tumor stage.

B04, aged 55 years

Moreover, for the metastatic feature, patients found it important to indicate whether the tumor had spread to the lymph nodes or to other parts of the body. Participants therefore suggested providing clear explanations of the patient and tumor characteristics. Additional features proposed by the PCa survivors were information about a person’s health status and information about comorbidity. Additional features requested by BCa survivors were tumor grade, HER2 status, and specific types of BCa such as triple negative. Finally, both groups asked for a feature dealing with a person’s family history of cancer (ie, genetics).

#### Theme 2: Need for Interpretation Support

##### Summary

Both PCa and BCa survivors identified challenges that could hinder the correct interpretation of the personalized cancer statistics by future users, and expressed the following needs for supporting patients with this.

##### Contextual Information

Both groups of participants expressed their wishes to see supplementary information that should accompany the personalized statistics. For instance, they commented that the current survival rates are actually better than those that were displayed by the tool, because patients with newly diagnosed cancer can benefit from advances in treatment options.

It is important to mention that all statistics here are about the past and are based on former treatment options. You should really communicate this to users…So the current statistics can only be more positive.

B01, aged 50 years

Furthermore, some BCa survivors thought that providing comparative information such as the chance of 10-year cancer recurrence related to the chance of getting cancer for the first time. Similarly, the participants with PCa stated that the 5- and 10-year survival statistics for patients with cancer should be placed in context by comparing them with the survival rates of people who do not have cancer.

Providing the survival rate for the norm population would be very useful. The survival rate of the normal population isn’t that great as well. If I see a 10-year survival rate of 21 percent for PCa patients [with stage 4], what does this 21 percent mean, and how does it compare [to the normal population]?

P03, aged 67 years

##### Statistical Numeracy

Several participants expressed their concerns about communicating personalized statistics to patients with low health or numeracy skills. They considered it important to explain that the personalized survival rates are still average statistics, and that supplementary information is highly needed especially for those patients who are lacking prior knowledge in statistics.

It is important that these statistics are not communicated in a scientific manner, but instead in a way that is understandable for those who do not have a background in statistics.

P02, aged 79 years

##### Emotional Aspects

Participants emphasized the importance of taking emotional aspects such as anxiety into account that may be evoked by viewing information about survival rates. Especially in the scenario with the less favorable survival statistic, some participants found the information shocking and uneasy to see and offered suggestions for adding warning statements about this.

I think it would be a good idea to advice people to see this information together with someone else. I could imagine that some people may find this [statistical] information emotionally difficult to interpret…Something like a disclaimer.

B05, aged 41 years

However, other participants did not experience this, and felt that disclosing personalized statistical information via this tool is of utmost importance for those who need it to become well informed, even though the statistics could be bad and provoke negative emotions. They felt that this would not destroy patients’ hope, but instead would create a more realistic picture.

Those people who want hope will not read this [personalized statistical information]. I think that if you have the [statistical] information, it should become available for everyone

B01, aged 50 years

I have searched for statistical information all night long. Having that knowledge [statistical information] makes me feel calm

B06, aged 63 years

#### Theme 3: Preference for Information Presentation

##### Summary

While viewing the tool, participants reported their preferences for presenting the personalized cancer statistics in terms of type of visualization, amount of information, and uncertainty around statistics.

##### Type of Visualization

Regarding the different types of visualization that we used for communicating the survival rates, almost all PCa and BCa survivors expressed a preference for the icon arrays. However, 1 participant with PCa commented that the icon arrays increased levels of anxiety because “they seemed too personal” [P03]. Overall, participants found the option to switch between different types of visualization valuable and helpful.

##### Amount of Information

In both groups, participants shared their views on whether we should give users a conscious choice of what information they would like to see, for instance, by giving them the option to expand texts when supplementary information about specific terms or statistics is preferred. Some participants argued that this would then satisfy both users who want detailed or supplementary information about the statistics and users who want to see as little as possible. This was also true for showing the visualizations by default, or providing patients the option to decide for themselves whether they want to see the information visually or not.

I was thinking of the graphic. Do you always want to show this to all patients, regardless of the type? You could also first show them the textual information, and then give them the option to view the information in a graphic, and which type of graphic. Because…what if the survival rate turns out to be very low. Then the icon arrays can very confrontational.

B01, aged 50 years

##### Uncertainty Around Statistics

Not all participants were aware of the imprecision of the statistics (ie, epistemic uncertainty), and they had conflicting views on whether or not we should disclose and communicate this. Some participants thought it might be too difficult and confusing to communicate, whereas others stated it may help patients understand that the statistics are less reliable and could be no more than an indication of what could happen. The participants with BCa showed a preference for communicating this kind of uncertainty only when calculating survival rates for small groups (eg, patients with BCa with triple-negative), or when the statistics were relatively poor (eg, less favorable survival rate). As one BCa survivor put it:

Here [sees a 5-year survival rate of 44% for a stage 4 BCa patient] you want to know the variation, because it may give the patient hope. If you have a poor statistic, but you see that the range is big, then you may think that you could still be on the positive side of the range. Whereas if you have a good statistic, then providing a range becomes less relevant.

B03, aged 57 years

This concludes the findings of the focus groups. In the next section, we will discuss the results from the think-aloud observations, which allow us to get a better insight into what cancer survivors might actually think and feel when confronted with personalized cancer statistics.

### Study 2: Think-Aloud Observations

#### Overview

The results of the think-aloud observations are presented below, structured around the 3 main themes that were identified from the focus group data (need for personalized statistics, need for interpretation support, and preference for information presentation). Table 2 displays an overview of the main results obtained during the think-aloud observations.

**Table 2:** Overview of results and statements made by participants during the think-aloud sessions (N=11).

Item		Value, n (%)
**Need for personalized statistics**		
	Mentioned that receiving personalized survival rate is valuable	9 (82)
	Showed less interest in (personalized) incidence rate	11 (100)
	Appreciated the conditional survival rates	10 (91)
	Wanted more clinical characteristics and treatment history for specifying statistics even further	6 (55)
**Need for interpretation support**		
	Found the supporting statements helpful and important	11 (100)
	Would not recommend using verbal labels for interpreting statistics (eg, to tell patients they will receive “good or bad” news)	3 (27)
	Experienced positive emotions (eg, sense of hope) while viewing the personalized statistics	9 (82)
	Experienced negative emotions (eg, shocked) while viewing the personalized statistics	7 (64)
	Mentioned that both favorable and unfavorable personalized statistics should be disclosed	11 (100)
	Found comparative information confronting when their personalized statistics were below average	5 (45)
	Appreciated comparative information when their personalized statistics were above average	5 (45)
**Preference for information presentation**		
	Preferred icon arrays for displaying personalized survival rates	6 (55)
	Preferred pie charts for displaying personalized survival rates	4 (36)
	Preferred bar charts for displaying personalized survival rates	1 (9)
	Appreciated the function of tailoring the type of visualization	8 (73)
	Preferred a short and concise result page	10 (91)
	Expressed a preference for tailoring the amount of information	5 (45)
	Appreciated verbal descriptions of uncertainty around personalized statistics	5 (45)
	Wanted to see confidence intervals along with the personalized statistics	2 (18)

#### Need for Personalized Statistics

Overall, most participants (n=9) mentioned that receiving the personalized survival rate was very valuable, of which 7 mentioned that they would use this tool after their diagnosis, and 2 only after a few years after diagnosis. Participants showed less interest in the information about cancer incidence, and 3 were even surprised by the personalized incidence rate, because they expected this statistic to be much higher. Similar to the focus group study, almost all participants (n=10) greatly appreciated the conditional survival rates, especially when initially being confronted with a less favorable survival rate. As participants put it, while thinking aloud:

Well, I think this [conditional survival rate] is very valuable… Indeed, if you have survived some years after diagnosis, you are no longer part of the group of patients that died, so from that moment your chances of survival increase enormously.

B03, aged 45 years

Yes, I get it. The survival rate increased from 47 percent to 87 percent. Well, then I am a real survivor! 87 out of 100 men, that’s high, isn’t?

P01, aged 68 years

However, similar to the focus group, 6 participants expressed their need for adding more clinical characteristics and treatment history to the tool for better personalizing the statistics.

#### Need for Interpretation Support

All participants found the supporting statements at the start of the tool very helpful and important, as they may help users become better prepared for receiving and interpreting the statistics. However, 3 participants explicitly mentioned that we should not use labels by telling users that the numbers they will see will be good or bad news. One participant commented, while thinking aloud:

I do not think that you can decide for someone else whether something is good or bad news. That is not up to you. It is also relative. I mean, if you see this [survival rate] you may think it’s good news, but I may think it’s bad news.

B05, aged 50 years

The same participant offered suggestions for replacing “good or bad news” with “favorable or less favorable than expected” [B05].

Participants also experienced and expressed a mix of positive and negative emotions while viewing the personalized statistics. The majority of the participants (n=9) expressed positive emotions such as a sense of hope, while viewing the conditional survival rates (n=8), or the favorable survival rate. However, 7 participants were “shocked” or felt “uneasy” when seeing the less favorable survival rate in comparison with the favorable generic, population-based survival rate. Those participants were surprised that so few people would survive after 5 years with these specific characteristics.

Oh god, this [less favorable personalized survival rate] is still after five years. Well this number is very different from the generic statistic [generic, population-based survival rate]. Pff, that really sucks!

B02, aged 60 years

Nevertheless, participants found it important to disclose the less favorable survival rates as well to create a realistic and fair picture. Some patients (n=5) found that emotions should be taken into account, but at the same time commented that those who do not want to see the personalized statistics will not visit the tool.

I did not experience any feelings, but I am also a rationally and realistically oriented person. I know some women who don’t want to see this kind of information, but the question is whether they will look for these statistics at all.

B03, aged 45 years

Furthermore, participants had mixed views on the comparative information between the personalized and generic, population-based statistics. This view typically depended on whether the personalized survival rate was above or below the generic statistic. Some participants (n=5) found the less favorable survival rate confronting when it was shown in comparison with the favorable generic survival rate. However, when participants’ personalized survival rate was higher than the average, others (n=5) thought it was supportive:

The [generic] survival rate is 89 percent… Oh well, that is a lot. Survival rate for patients with the above characteristics is 94 percent. Okay, so my prognosis is better than the average [prognosis]. Well that’s good news.

P03, aged 60 years

This [seeing both personalized and generic survival rate] is fine, and seems like an added value to me. This way, you can see whether you are below or above the average survival rate.

P04, aged 69 years

Participants further expressed concerns about terminology used in the tool. For instance, 7 participants were not familiar with the term “tumor stage,” but rather with alternative features such as TNM stage or the presence of metastases or not. Participants further recommended to avoid complex terms such as “incidence” or “conditional survival” (Figure 2), and preferred the tool version in which these terms were explained in plain language (Figure 3).

#### Preference for Information Presentation

Participant preferences for visualizing the personalized survival rates were in line with those of participants in the focus group, with the majority preferring icon arrays (n=6), followed by pie (n=4) and bar charts (n=1). However, participant reactions to the “human aspect” of the icon arrays varied, with some appreciating the pictographs since the survival rates are about people, while others expressed concerns that they were too confronting. Despite this variation in preferences and (emotional) reaction, most participants appreciated the function of tailoring the type of visualization (n=8).

I didn’t like to be confronted with this figure [icon array], because 38 percent [chance of survival]...Here you should have the option to switch between figures. When the percentage was displayed by means of a pie chart, I experienced it as less shocking than when it is presented with pictographs. I think here you should be able to make a choice in how you want to see it.

B01, aged 54 years

Furthermore, regarding the amount of information, most participants preferred the short and concise result page of the tool (n=10). Participants typically commented that they primarily used the tool to see statistics and survival rates as soon as possible, and therefore expected to see numerical information rather than large pieces of text. Almost half of the participants expressed a preference for tailoring the amount of information and expanding the text for certain topics (eg, complex terms, supplementary information about the NCR) if desired (n=5). Again, this was mostly preferred by participants who were shocked by the less favorable survival rates. Finally, 5 participants appreciated the verbal descriptions of uncertainty around the statistics that we presented as part of the supporting statements, and 2 participants wanted to see confidence intervals along with the statistics.

## Discussion

### Principal Findings

This study aimed to explore needs and preferences of cancer survivors for communicating personalized statistics from a Dutch nationwide population-based registry, the NCR [22]. We developed different versions of a tool that allows patients to enter personal and disease-related characteristics for determining personalized incidence, survival, and conditional survival rates. We applied a qualitative multimethod study approach, by collecting group data through focus groups and individual data via think-aloud observations combined with semistructured interviews.

Our study suggests that the majority of our selective sample of cancer survivors (in both the focus group study and think-aloud sessions) have a desire to receive personalized cancer statistics. Survivors expressed an overarching desire for especially receiving tailored survival rates and conditional survival rates; they showed less interest in the personalized incidence rate, but they still thought it could be useful for some patients. Overall, the majority expressed intention to use the tool for viewing personalized statistics, regardless of the outcome. Furthermore, survivors wanted to receive a range of personalized statistics, such as personalized risk information about treatment outcomes (eg, side effects, survival, recurrence rate, or quality of life). These results support previous findings that most (but not all) patients want detailed and individualized information about their prognostic situation [2-4,37,38], with especially a strong need for personalized (conditional) survival rates and treatment outcomes (eg, risks of side effects, quality of life, or recurrence rates).

When it comes to communicating personalized statistics to patients, we found that survivors expressed a need for being provided with supporting information that should help correctly interpreting the statistics. For instance, in both focus groups and think-aloud observations, cancer survivors mentioned the importance of adding contextual information (eg, explaining the influence of treatment on survival over time, providing comparative information including generic, population-based statistics), which should help put the personalized statistics into perspective [39,40]. Next to that, survivors in the focus groups reported that they processed personalized survival statistics emotionally, and were viewing the information under the influence of emotions such as feelings of distress. Indeed, this was captured during the think-aloud observations, in which some participants were confronted by the less favorable survival statistic compared with the favorable generic survival statistic. Reminding or preparing patients about this was found to be helpful, although the use of specific interpretation labels such as “good” or “bad” news were strongly discouraged. At the same time, we observed that the disclosure of conditional survival rates had a positive effect on cancer survivors’ sense of hope, which is in line with previous work on the link between hope and disclosure of prognostic information [37].

Regarding the preference of cancer survivors for presenting the personalized statistical information, participants expressed an overarching preference for simplicity and conciseness. They found it important that the key information (survival rates) was immediately visible to them. Although some participants wished to see more information about the details of the statistics, others did not appreciate this. This challenge of finding a balance between fully informing patients about the statistics while not simultaneously overwhelming them by providing too much information has also been found elsewhere [41,42]. There were survivors who appreciated the option to tailor the amount of information, by extending texts when more detail was preferred [43], or by choosing whether or not one wants to see the visual representation of the survival statistic. Finally, regarding the type of visualization, most participants preferred the pictographs, which is in line with previous research [44], although some found the use of pictographs inappropriate and frightening for communicating survival rates [45]. We further found that the option to switch between different types of visualization was greatly appreciated by our participants, which may therefore solve the variety in presentation preferences among cancer survivors [46].

### Strengths and Limitations

A strength of this study is that we employed multiple rigorous qualitative methods (focus groups and think-aloud observations combined with semistructured interviews) that complied with reporting standards [26]. The focus groups (study 1) allowed us to gather group data on cancer survivors’ needs, preferences, and perceptions about disclosing personalized cancer statistics, while the think-aloud observations (study 2) revealed spontaneous thoughts and feelings of survivors while being confronted with personalized statistics. At the same time, the think-aloud method has sometimes been criticized regarding its validity and reliability [47,48], as it may be cognitively demanding for participants to complete a task while simultaneously verbalizing their thoughts, opinions, and feelings. However, following previous research [32], we partially tackled this issue by conducting semistructured interviews after the think-aloud sessions during which participants could elaborate on their verbal statements and experiences with the tool. Even though we conducted all studies with cancer survivors (who have experience with being confronted with a cancer diagnosis), we had to make use of hypothetical case examples instead of participants’ own patient and tumor characteristics. This may have limited the ecological validity of the results, and may have influenced the emotional processes that patients did (or did not) experience while interacting with the tool.

Another limitation is that we recruited (active) cancer survivors involved in online cancer communities or patient organizations. It has been demonstrated that this selection of cancer survivors may not be fully representative of the general cancer population, as they are typically somewhat higher educated and make more extensive use of the internet [49]. Several studies suggest that lower education is associated with lower eHealth use [50]. Furthermore, we did not measure participants’ health literacy or numeracy skills, although some participants in our study expressed their concerns about communicating statistics to patients with low health or numeracy skills. Therefore, supplementary information or advice to discuss the results with clinician is highly needed especially for those patients who are lacking prior knowledge in statistics, or who may have less education. Despite this shortcoming, our interactive tools did comply with best practices and risk communication guidelines for communicating statistical information to the general public [24,51-54], and their content was developed by using a plain language approach (eg, using everyday language, and using logically structured and focused information) [55]. A related limitation is that we only included BCa and PCa survivors, which makes it challenging to generalize our results to other oncology populations and those patients in active treatment. However, a recent study showed that internet use and wishes for online health information and statistics do not differ between patients with different cancer types [49]. Nevertheless, for future developments and eventual release of a possible real-life web-based NCR tool, it is important to test the understanding of the tool also among the general cancer population, preferably with variation in terms of cancer type, educational background, health literacy, and numeracy skills.

### Implications and Future Directions

Our results contribute to the rapidly expanding field of personalized risk communication and tailored health communication, as they further enhance our understanding of how and why we should make efforts in disclosing and communicating personalized risks statistics from registry data to patients. For instance, our data provide support for a novel recommendation of allowing users to modify the type of visualization in line with their preferences. Over the years, several best practices and communication guidelines have been developed for the delivery of risk and statistical information to patients [24,51,52,54,56], particularly with an emphasis on searching for a single-best strategy. However, preferences for certain visualizations may vary between individuals [57], and therefore tailoring the type of visual aid toward the user’s preference may be a promising additional risk communication strategy to consider. Another novel finding of our study is that some of the risk communication guidelines for communicating generic, population-based statistics may yield unexpected effects when they are used for communicating risks or statistics that are personalized. For instance, icon arrays—a recommended type of visualization for explaining risks and statistics—were preferred by most participants in our study (consistent with other studies [58,59]), but they also evoked feelings of distress as they became too personal to some patients [45]. Therefore, systematic knowledge about how patients will perceive and process visual aids that communicate personalized risks statistics is needed, as well as future investigations about the effects of tailoring the type of visual aid or the amount of information on associated risk perception and comprehension outcomes.

Furthermore, our results are encouraging for research into needs and preferences of patients with cancer with respect to personalized information provision and the disclosure of big health data [11,17]. The majority of our sample expressed a need for receiving personalized statistics on different topics before and after their initial treatment, ranging from survival rates to risk information about treatment side effects. We therefore recommend further development and implementation of data-driven personalized decision aids and disease risk prediction models (either based on registry, clinical, or patient-reported outcome data) in and outside The Netherlands [8,11,15,20,21], and support their availability to patients and clinicians in daily routine practice and to laypersons on the internet. At the same time, this development comes with several challenges, which may explain why some (personalized) cancer statistics are not currently available to the general public. For instance, some additional items for personalizing survival statistics as requested by participants are not readily available within the Dutch registry (eg, information on genetic factors or comorbidity). Relatedly, increasing the number of items in this case may lead to smaller subgroups, which in turn may lead to uncertain and less reliable personalized statistics. As such, the utility of and preference for personalized statistics may differ markedly depending on how reliable the information is, and further exploration on these aspects is highly warranted.

The results of our study also have a number of novel practical implications for the design and implementation of personalized, data-driven information support tools for cancer survivors (Textbox 1). We have shown that making such tools available to patients and the general public comes with several challenges such as avoiding technical language that is needed to describe statistical or medical terms, making sure that all patients will correctly interpret the statistical information, and not overwhelming them with visualizations that display less favorable survival outcomes. A key lesson from our qualitative studies is that there does not seem to exist a single perfect communication format for the delivery of personalized cancer statistics. We therefore believe that many of the issues identified with our potential NCR tool could be solved by applying a number of different personalization techniques, such as tailoring the amount of information (eg, expanding text boxes for those who want detailed and supplementary information) [43], or tailoring the type of visualization in line with patient preferences. Furthermore, as some patients may experience difficulties with correctly interpreting the statistical information, several strategies could be taken into account such as the provision of contextual information about the statistics, or comparative information by showing average statistical outcomes of other patients.

Finally, although it has been shown that personalized statistics are typically perceived as more relevant [25], and hence better processed than generic information [60,61], our findings suggest that tool developers should not underestimate the role of affect in this process [62]. We observed that some participants processed statistical information emotionally, and expressed to be confronted by the less favorable survival rates. Making web-based prediction tools publicly available to patients and relatives thus faces the challenge of avoiding discouraging patients with less favorable survival rates of prognosis from having hope. This is especially challenging for tools that rely on automatically generated textual explanations, for instance produced by robot writers that cannot easily provide contextual information in a similar way as a doctor can do during a consultation [63]. However, in line with previous information needs studies, our participants indicated that for those patients who really want honest prognostic information the levels of hope will maintain, even when the news is bad [38]. We recommend tool developers to provide supporting or preparatory information about the emotional aspects, and to find ways on how to tailor automatically generated sentences and explanations on poor prognosis and treatment outcomes to patients.

**Recommendations for the development of tools that communicate personalized health statistics to the public.**

**The need for personalized statistics**

Regarding the type of statistics:

- Consider communicating personalized survival statistics together with conditional survival statistics.
- Communicate not only statistics about personalized cancer incidence, but also about survival, conditional survival, and treatment outcomes (eg, side effects, quality of life).
- Consider and evaluate multiple patient (age, gender, lifestyle) and clinical (disease stage, tumor characteristics) characteristics for tailoring the statistics.

**The need for interpretation support**

Regarding difficulties with interpreting personalized statistical information:

- Provide contextual information about the statistics and use clear explanations on the intended use.
- Consider communicating comparative information by showing statistics of the average patient in addition to the personalized statistics.
- Use plain and appropriate language and make sure that data entry characteristics are known by patients (or at least provided by their health care providers).

Regarding emotions or feelings of distress that may arise while viewing (less favorable) statistics:

- Prepare patients for the less favorable survival statistics via reminders or warning statements.
- Avoid using evaluative labels such as “good” or “bad” survival statistics.

**Preferences for information presentation**

Regarding variation in preference for type of visualization:

- Incorporate multiple types of visualization for displaying the statistical information.
- Allow patients to modify the type of visualization according to their preference.

Regarding variation in preference for the amount of information:

- Keep the amount of information short and concise.
- Allow patients to tailor the amount of information, for instance, by incorporating the option to expand text for showing detailed information.

### Conclusions

The majority of our sample of cancer survivors expressed a desire for receiving personalized cancer statistics such as specific and relevant data on survival and conditional survival. This is encouraging for those who are developing personalized information tools for patients that are drawing on cancer registry data or other medical databases, especially in an era of personalized health care and open access of big health data. Presenting personalized statistics to the public remains challenging and calls for tailoring strategies, as cancer survivors in our study demonstrated variation in their preferences for communicating the statistics. As a result of these findings, our research group is currently developing a real-life web-based tool that communicates personalized NCR statistics, which will be further evaluated among different stakeholders including patients, relatives, and health care providers. Given the valuable information generated in collaboration with cancer survivors, we suggest that this approach and findings can be used to design data-driven personalized information (and decision-support tools) tools for patients with cancer and other disease conditions.

## Appendix

Multimedia Appendix 1 Consolidated Criteria for Reporting Qualitative Research (COREQ).

Multimedia Appendix 2 Sketches of a non-interactive version of the tool, used during the focus groups.

Multimedia Appendix 3 Screenshots of an interactive version of the tool, used during the think-aloud observations.

Multimedia Appendix 4 Topic guide for the semi-structured interviews.

## Abbreviations

BCa: breast cancer
BVN: Borstkankervereniging Nederland (Dutch Breast Cancer Association)
IKNL: Netherlands Comprehensive Cancer Organisation
NCR: Netherlands Cancer Registry
PCa: prostate cancer
PKS: Prostaatkankerstichting (Dutch Prostate Cancer Foundation)
SEER*CSC: American Surveillance, Epidemiology, and End Results Cancer Survival Calculator
