# Supplementary material for: Exploring Cancer Survivor Needs and Preferences for Communicating Personalized Cancer Statistics From Registry Data: Qualitative Multimethod Study
Source: JMIR Cancer. 2021 Oct 25;7(4):e25659. doi: 10.2196/25659 (PMC8576563; doi:10.2196/25659)
Supplement: Multimedia Appendix 2 [file cancer_v7i4e25659_app2.pdf]

Multimedia 2

Materials: Non-interactive sketches of a tool that communicates personalized cancer statistics from the NCR

Study 1: Focus groups

Authors: Vromans et al.

Content

- **Sketches example 1: breast cancer**
  - Survival statistic: Favorable
- **Sketches example 2: breast cancer**
  - Survival statistic: Less favorable

Sketches breast cancer example 1

- Survival statistic: Favorable

Overlevingscijfers borstkanker

Printen | Opslaan

Belangrijk om te weten voordat u verder leest:

Met **overleving** bedoelen we hoeveel mensen met een bepaalde vorm van kanker na een bepaalde periode nog in leven zijn.

De algemene cijfers gelden voor een groep patiënten. Deze zijn niet zomaar te vertalen naar uw individuele situatie.

Het is ook mogelijk om de cijfers te ontvangen op basis van een relevante groep patiënten. Deze cijfers kunnen dichter bij uw situatie staan. U kunt dan kenmerken zoals geboortejaar, tumorstadium en tijd sinds diagnose opgeven om zo cijfers op maat te ontvangen. Ook voor deze cijfers op maat geldt dat uw vooruitzichten beter of slechter kunnen zijn dan die van een hele groep. Bespreek uw vooruitzichten met uw arts.

Wilt u liever algemene overlevingscijfers zien, of cijfers op maat?

Algemene cijfers | Cijfers op maat

Translation:
With survival we mean the percentage of people with cancer still alive after a particular amount of time.

The generic statistics apply to a group of patients. These cannot relate to your personal situation.

It is also possible to receive statistics based on a relevant group of patients. These statistics may better relate to your situation. You may enter characteristics like year of birth, tumor stage, or time since diagnosis, in order to receive tailored statistics. However, your prognosis may still be better or worse than the prognosis of this group. Please discuss your prognosis with your doctor.

Would you like to see generic or personalized statistics?

Overlevingscijfers borstkanker

Printen | Opslaan

1 — 2 — 3

Persoonlijke gegevens

Wat is uw geslacht?

Vrouw

In welk jaar bent u geboren?

1948

Vorige | Volgende

Translation:
Personal data
What is your gender? (female)
What is your year of birth? (1948)

Overlevingscijfers borstkanker

Printen | Opslaan

1 — 2 — 3

Gegevens over uw ziekte bij diagnose

In welk jaar kreeg u de diagnose?

2016

Wat was het stadium van uw tumor bij diagnose? ?

Stadium II

Vorige | Toon resultaten

Translation:
Disease-related data
Year of diagnosis? (2016)
Tumor stage? (Stage II)

Overlevingscijfers borstkanker

Printen | Opslaan

1 — 2 — 3

Gegevens over uw ziekte bij diagnose

In welk jaar kreeg u de diagnose?

2016

Wat was het stadium van uw tumor bij diagnose? ?

Weet ik niet

Waren er uitzaaiingen in andere delen van uw lichaam bij diagnose? ?

Kies
- Ja
- Nee
- Weet ik niet

Vorige | Toon resultaten

Translation:
Disease-related data
Year of diagnosis? (2016)
Tumor stage? (I don't know)
Did the cancer spread to other parts of your body? (yes, no, I don't know)

Overlevingscijfers borstkanker

Printen Opslaan

1 2 3

Met **overleving** bedoelen we hoeveel mensen met een bepaalde vorm van kanker na een bepaalde periode nog in leven zijn. De kans om borstkanker te overleven is de laatste jaren gestegen.

Overlevingscijfer op maat

U gaf aan de volgende kenmerken te hebben bij diagnose:

Geslacht: Vrouw | Geboortejaar: 1948 | Diagnosejaar: 2016 | Stadium: II

5 jaar na diagnose is ongeveer **82%** van de borstkanker patiënten nog in leven (82 van de 100 vrouwen). Deze kans is berekend op basis van een groep patiënten met vergelijkbare kenmerken als u bij diagnose.

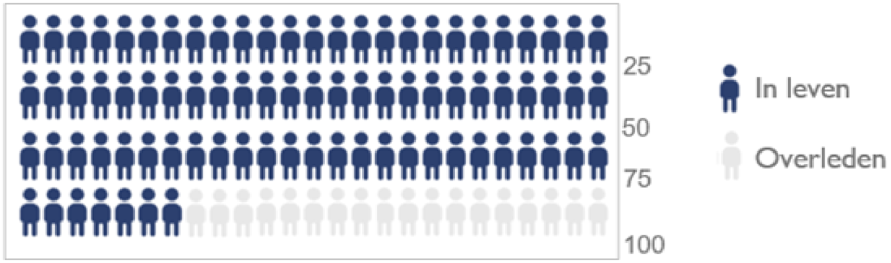

5 jaar na diagnose

Uw vooruitzichten kunnen nog steeds beter of slechter zijn dan de vooruitzichten voor deze specifieke groep. Bespreek uw vooruitzichten met uw arts.

Translation:
With survival we mean the percentage of people with cancer still alive after a particular amount of time. Overall, breast cancer survival rates have been improving in recent decades.

Personalized statistic
U entered the following data:

5 years after diagnosis, about 82 percent of women diagnosed with breast cancer are still alive (82 out of 100 women). This statistic is based on patients like you.

Your prognosis may be better or worse than the prognosis of this specific group of patients. Please discuss your prognosis with your doctor.

Overlevingscijfers borstkanker

Printen Opslaan

1 2 3

Met **overleving** bedoelen we hoeveel mensen met een bepaalde vorm van kanker na een bepaalde periode nog in leven zijn. De kans om borstkanker te overleven is de laatste jaren gestegen.

Overlevingscijfer op maat

U gaf aan de volgende kenmerken te hebben bij diagnose:

Geslacht: Vrouw | Geboortejaar: 1948 | Diagnosejaar: 2016 | Stadium: II

5 jaar na diagnose is ongeveer **82%** van de borstkanker patiënten nog in leven (82 van de 100 vrouwen). Deze kans is berekend op basis van een groep patiënten met vergelijkbare kenmerken als u bij diagnose.

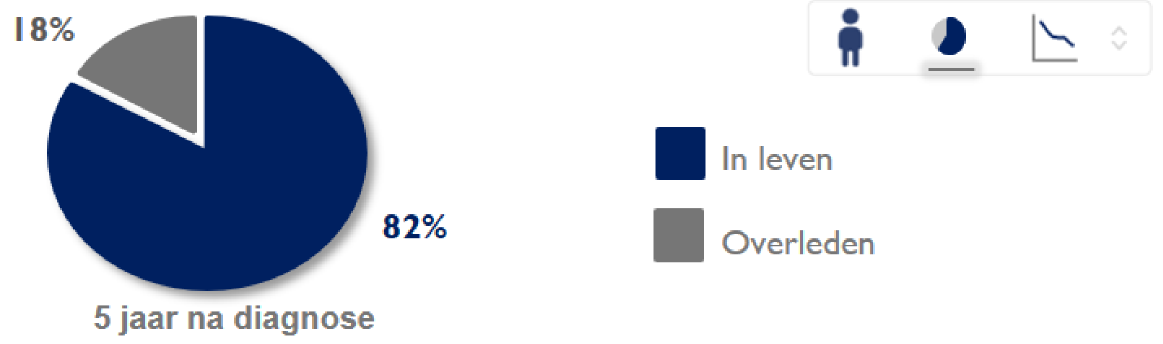

Uw vooruitzichten kunnen nog steeds beter of slechter zijn dan de vooruitzichten voor deze specifieke groep. Bespreek uw vooruitzichten met uw arts.

Overlevingscijfers borstkanker

Printen Opslaan

1 2 3

Met **overleving** bedoelen we hoeveel mensen met een bepaalde vorm van kanker na een bepaalde periode nog in leven zijn. De kans om borstkanker te overleven is de laatste jaren gestegen.

Overlevingscijfer op maat

U gaf aan de volgende kenmerken te hebben bij diagnose:

Geslacht: Vrouw	Geboortejaar: 1948	Diagnosejaar: 2016	Stadium: II

5 jaar na diagnose is ongeveer **82%** van de borstkanker patiënten nog in leven (82 van de 100 vrouwen). Deze kans is berekend op basis van een groep patiënten met vergelijkbare kenmerken als u bij diagnose.

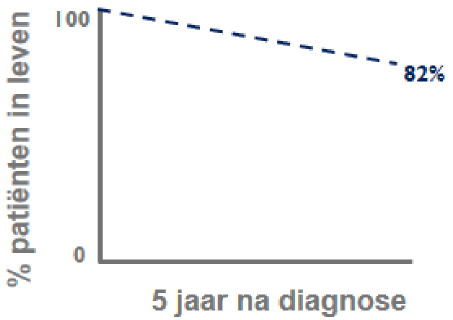

Uw vooruitzichten kunnen nog steeds beter of slechter zijn dan de vooruitzichten voor deze specifieke groep. Bespreek uw vooruitzichten met uw arts.

Overlevingscijfers borstkanker

Printen Opslaan

1 2 3

Overlevingscijfer op maat

U gaf aan de volgende kenmerken te hebben bij diagnose:

Geslacht: Vrouw	Geboortejaar: 1948	Diagnosejaar: 2016	Stadium: II

5 jaar na diagnose is ongeveer **82%** van de borstkanker patiënten nog in leven (82 van de 100 vrouwen). Deze kans is berekend op basis van een groep patiënten met vergelijkbare kenmerken als u bij diagnose.

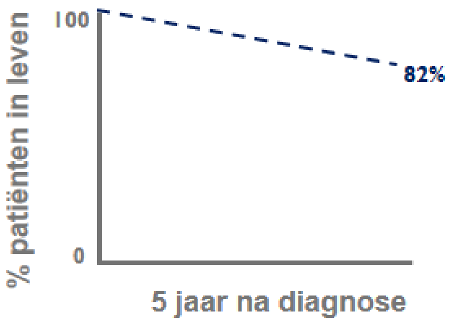

Uw vooruitzichten kunnen nog steeds beter of slechter zijn dan de vooruitzichten voor deze specifieke groep. Bespreek uw vooruitzichten met uw arts.

De overlevingscijfers worden vaak beter met ieder jaar dat de patiënt overleeft.
Van de patiënten die 2 jaar na diagnose nog in leven zijn, is de kans dat iemand 5 jaar na diagnose nog leeft gestegen van 82 procent naar 90 procent (90 van de 100 vrouwen).

Translation:
With survival we mean the percentage of people with cancer still alive after a particular amount of time. Overall, breast cancer survival rates have been improving in recent decades.

Personalized statistic
U entered the following data:

5 years after diagnosis, about 82 percent of women diagnosed with breast cancer are still alive (82 out of 100 women). This statistic is based on patients like you.

Your prognosis may be better or worse than the prognosis of this specific group of patients. Please discuss your prognosis with your doctor.

Survival statistics typically improve the longer a patient survives. Of those patients like you who were still alive 2 years after diagnosis, the chance that they would still be alive 5 years after diagnosis increased from 82 percent to 90 percent (90 out of 100 women).

Sketches breast cancer example 2

- Survival statistic: Less favorable

Overlevingscijfers borstkanker

Printen | Opslaan

Belangrijk om te weten voordat u verder leest:

Met **overleving** bedoelen we hoeveel mensen met een bepaalde vorm van kanker na een bepaalde periode nog in leven zijn.

De algemene cijfers gelden voor een groep patiënten. Deze zijn niet zomaar te vertalen naar uw individuele situatie.

Het is ook mogelijk om de cijfers te ontvangen op basis van een relevante groep patiënten. Deze cijfers kunnen dichter bij uw situatie staan. U kunt dan kenmerken zoals geboortejaar, tumorstadium en tijd sinds diagnose opgeven om zo cijfers op maat te ontvangen. Ook voor deze cijfers op maat geldt dat uw vooruitzichten beter of slechter kunnen zijn dan die van een hele groep. Bespreek uw vooruitzichten met uw arts.

Wilt u liever algemene overlevingscijfers zien, of cijfers op maat?

Algemene cijfers | Cijfers op maat

Translation:
With survival we mean the percentage of people with cancer still alive after a particular amount of time.

The generic statistics apply to a group of patients. These cannot relate to your personal situation.

It is also possible to receive statistics based on a relevant group of patients. These statistics may better relate to your situation. You may enter characteristics like year of birth, tumor stage, or time since diagnosis, in order to receive tailored statistics. However, your prognosis may still be better or worse than the prognosis of this group. Please discuss your prognosis with your doctor.

Would you like to see generic or personalized statistics?

Overlevingscijfers borstkanker

Printen | Opslaan

1 — 2 — 3

Persoonlijke gegevens

Wat is uw geslacht?

Vrouw

In welk jaar bent u geboren?

1960

Vorige | Volgende

Translation:
Personal data
What is your gender? (female)
What is your year of birth? (1960)

Overlevingscijfers borstkanker

Printen   Opslaan

1 — 2 — 3

Gegevens over uw ziekte bij diagnose

In welk jaar kreeg u de diagnose?

2016

Wat was het stadium van uw tumor bij diagnose? ?

Weet ik niet

Waren er uitzaaiingen in andere delen van uw lichaam bij diagnose? ?

Ja

Vorige   Toon resultaten

Translation:
Disease-related data
Year of diagnosis? (2016)
Tumor stage? (I don't know)
Did the cancer spread to other parts of your body? (yes)

Overlevingscijfers borstkanker

Printen Opslaan

1 2 3

Met **overleving** bedoelen we hoeveel mensen met een bepaalde vorm van kanker na een bepaalde periode nog in leven zijn. De kans om borstkanker te overleven is de laatste jaren gestegen.

Overlevingscijfer op maat

U gaf aan de volgende kenmerken te hebben bij diagnose:

Geslacht: Vrouw | Geboortejaar: 1960 | Diagnosejaar: 2017 | Uitzaaiingen: Ja

5 jaar na diagnose is ongeveer **44%** van de borstkanker patiënten nog in leven (44 van de 100 vrouwen). Deze kans is berekend op basis van een groep patiënten met vergelijkbare kenmerken als u bij diagnose.

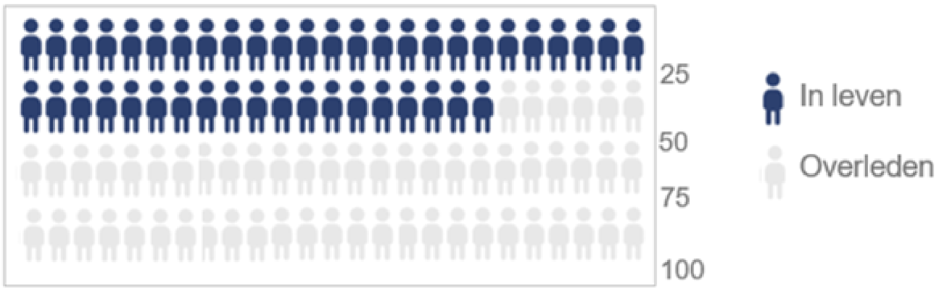

5 jaar na diagnose

Uw vooruitzichten kunnen nog steeds beter of slechter zijn dan de vooruitzichten voor deze specifieke groep. Bespreek uw vooruitzichten met uw arts.

Translation:
With survival we mean the percentage of people with cancer still alive after a particular amount of time. Overall, breast cancer survival rates have been improving in recent decades.

Personalized statistic
U entered the following data:

5 years after diagnosis, about 44 percent of women diagnosed with breast cancer are still alive (44 out of 100 women). This statistic is based on patients like you.

Your prognosis may be better or worse than the prognosis of this specific group of patients. Please discuss your prognosis with your doctor.

Overlevingscijfers borstkanker

Printen Opslaan

1 2 3

Met **overleving** bedoelen we hoeveel mensen met een bepaalde vorm van kanker na een bepaalde periode nog in leven zijn. De kans om borstkanker te overleven is de laatste jaren gestegen.

Overlevingscijfer op maat

U gaf aan de volgende kenmerken te hebben bij diagnose:

Geslacht: Vrouw | Geboortejaar: 1948 | Diagnosejaar: 2016 | Stadium: II

5 jaar na diagnose is ongeveer **44%** van de borstkanker patiënten nog in leven (44 van de 100 vrouwen). Deze kans is berekend op basis van een groep patiënten met vergelijkbare kenmerken als u bij diagnose.

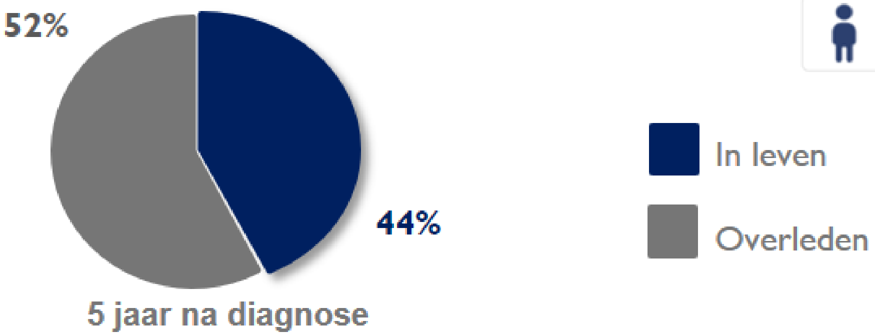

Uw vooruitzichten kunnen nog steeds beter of slechter zijn dan de vooruitzichten voor deze specifieke groep. Bespreek uw vooruitzichten met uw arts.

Translation:
With survival we mean the percentage of people with cancer still alive after a particular amount of time. Overall, breast cancer survival rates have been improving in recent decades.

Overlevingscijfers borstkanker

Printen Opslaan

1 2 3

Met **overleving** bedoelen we hoeveel mensen met een bepaalde vorm van kanker na een bepaalde periode nog in leven zijn. De kans om borstkanker te overleven is de laatste jaren gestegen.

Overlevingscijfer op maat

U gaf aan de volgende kenmerken te hebben bij diagnose:

Geslacht: Vrouw	Geboortejaar: 1948	Diagnosejaar: 2016	Stadium: II

5 jaar na diagnose is ongeveer **44%** van de borstkanker patiënten nog in leven (44 van de 100 vrouwen). Deze kans is berekend op basis van een groep patiënten met vergelijkbare kenmerken als u bij diagnose.

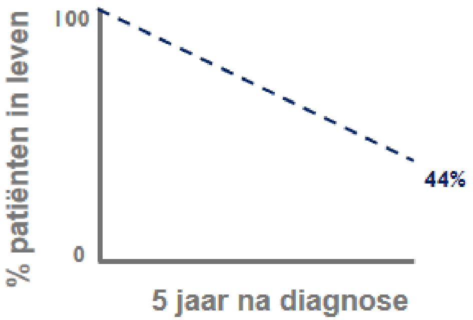

Uw vooruitzichten kunnen nog steeds beter of slechter zijn dan de vooruitzichten voor deze specifieke groep. Bespreek uw vooruitzichten met uw arts.

Overlevingscijfers borstkanker

Printen | Opslaan

1 — 2 — 3

Overlevingscijfer op maat

U gaf aan de volgende kenmerken te hebben bij diagnose:

Geslacht: Vrouw	Geboortejaar: 1948	Diagnosejaar: 2016	Stadium: II

5 jaar na diagnose is ongeveer **44%** van de borstkanker patiënten nog in leven (44 van de 100 vrouwen). Deze kans is berekend op basis van een groep patiënten met vergelijkbare kenmerken als u bij diagnose.

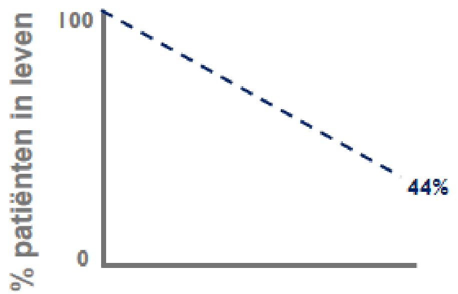

Uw vooruitzichten kunnen nog steeds beter of slechter zijn dan de vooruitzichten voor deze specifieke groep. Bespreek uw vooruitzichten met uw arts.

De overlevingscijfers worden vaak beter met ieder jaar dat de patiënt overleeft. Van de patiënten die 2 jaar na diagnose nog in leven zijn, is de kans dat iemand 5 jaar na diagnose nog leeft gestegen van 44 procent naar 60 procent (60 van de 100 vrouwen).

Translation:
With survival we mean the percentage of people with cancer still alive after a particular amount of time. Overall, breast cancer survival rates have been improving in recent decades.

Personalized statistic
U entered the following data:

5 years after diagnosis, about 82 percent of women diagnosed with breast cancer are still alive (82 out of 100 women). This statistic is based on patients like you.

Your prognosis may be better or worse than the prognosis of this specific group of patients. Please discuss your prognosis with your doctor.

Survival statistics typically improve the longer a patient survives. Of those patients like you who were still alive 2 years after diagnosis, the chance that they would still be alive 5 years after diagnosis increased from 44 percent to 60 percent (60 out of 100 women).
