# Supplementary material for: Exploring Cancer Survivor Needs and Preferences for Communicating Personalized Cancer Statistics From Registry Data: Qualitative Multimethod Study
Source: JMIR Cancer. 2021 Oct 25;7(4):e25659. doi: 10.2196/25659 (PMC8576563; doi:10.2196/25659)
Supplement: Multimedia Appendix 3 [file cancer_v7i4e25659_app3.pdf]

Multimedia 3

Materials: Screenshots of an interactive version of a tool that communicates personalized cancer statistics from the NCR

Study 2: Think-aloud observations

Authors: Vromans et al.

Content

- **Example 1: prostate cancer**
  - Survival statistics: Favorable
  - Version: Short and concise tool version
- **Example 2: prostate cancer**
  - Survival statistics: Less favorable
  - Version: Long and detailed tool version
- **Example 3: breast cancer**
  - Survival statistics: Favorable
  - Version: Long and detailed version
- **Example 4: breast cancer**
  - Survival statistics: Less favorable
  - Version: Short and concise version

Example 1: prostate cancer

- Survival statistic: Favorable
- Version: Short and concise tool version

Menu Zoeken Inloggen Registreren

Prostaatkanker

Wat is..

Onderz

Behand

Nieuwe

Gevolge

Niet me

Wat is...?

- Prostaatkanker
- Symptomen bij prostaatkanker
- Risicofactoren van prostaatkanker
- Bevolkingsonderzoek prostaatkanker
- Erfelijkheid en prostaatkanker
- Patiëntenorganisatie voor prostaatkanker
- **Overlevingscijfers prostaatkanker**
- Overige cijfers

Overlevingscijfers prostaatkanker

De volgende pagina's geven cijfers over prostaatkanker. U kunt zien hoeveel mensen jaarlijks prostaatkanker krijgen en hoeveel mensen deze ziekte overleven. De statistische berekeningen worden gedaan met de gegevens uit de Nederlandse Kankerregistratie (NKR).

Als u vragen heeft, is het belangrijk om de betreffende pagina te printen en mee te nemen naar het ziekenhuis, zodat u de informatie kunt bespreken met uw arts.

Volgende

Translation:

Survival statistics prostate cancer

The next pages will show you statistics about prostate cancer. You will see how many men are diagnosed with prostate cancer, and how many of these men will survive prostate cancer. The statistics are based on data from the Netherlands Cancer Registry (NCR).

If you have any questions, you could always print the respective pages and take them with you to the next visit with your doctor, so you can discuss the results with him/her.

Menu | Zoeken | Inloggen | Registreren

Prostaatkanker

Wat is... | Onderz | Behand | Nieuwe | Gevolge | Niet me

Wat is...?

- Prostaatkanker
- Symptomen bij prostaatkanker
- Risicofactoren van prostaatkanker
- Bevolkingsonderzoek prostaatkanker
- Erfelijkheid en prostaatkanker
- Patiëntenorganisatie voor prostaatkanker
- **Overlevingscijfers prostaatkanker**
- Overige cijfers

1 — 2 — 3

Belangrijk om te weten voordat u verder gaat:

- Het is belangrijk dat u nadenkt over wat u wilt weten. Deze cijfers kunnen ‘goed’ nieuws bevatten maar ook ‘slecht’ nieuws. Wat iemand wil weten, verschilt per persoon.
- Alle getallen die wij geven, zijn gebaseerd op het verloop in het verleden. Onder invloed van betere diagnostiek en behandelingen, kunnen overlevingskansen voor huidige en toekomstige patiënten beter zijn dan de cijfers die wij hier tonen.
- Het lijkt soms alsof wij het heel ‘precies’ weten. Er is echter altijd sprake van onzekerheid. Hoe kleiner de aantallen, hoe groter de onzekerheid over het getal.

Vorige | Volgende

Translation:

Important to know before you continue:

- It is important that you think about what you want to know. These statistics may contain ‘good’ or ‘bad’ news. What someone wants to know, differs per person.
- All statistics that we provide are based on what happened in the past. Given the improvements in terms of diagnosis and treatments, the survival statistics for recently diagnosed and future patients may be better than those statistics that we show here.
- It may seem that we know everything without any uncertainty. However, there is always uncertainty around statistics.

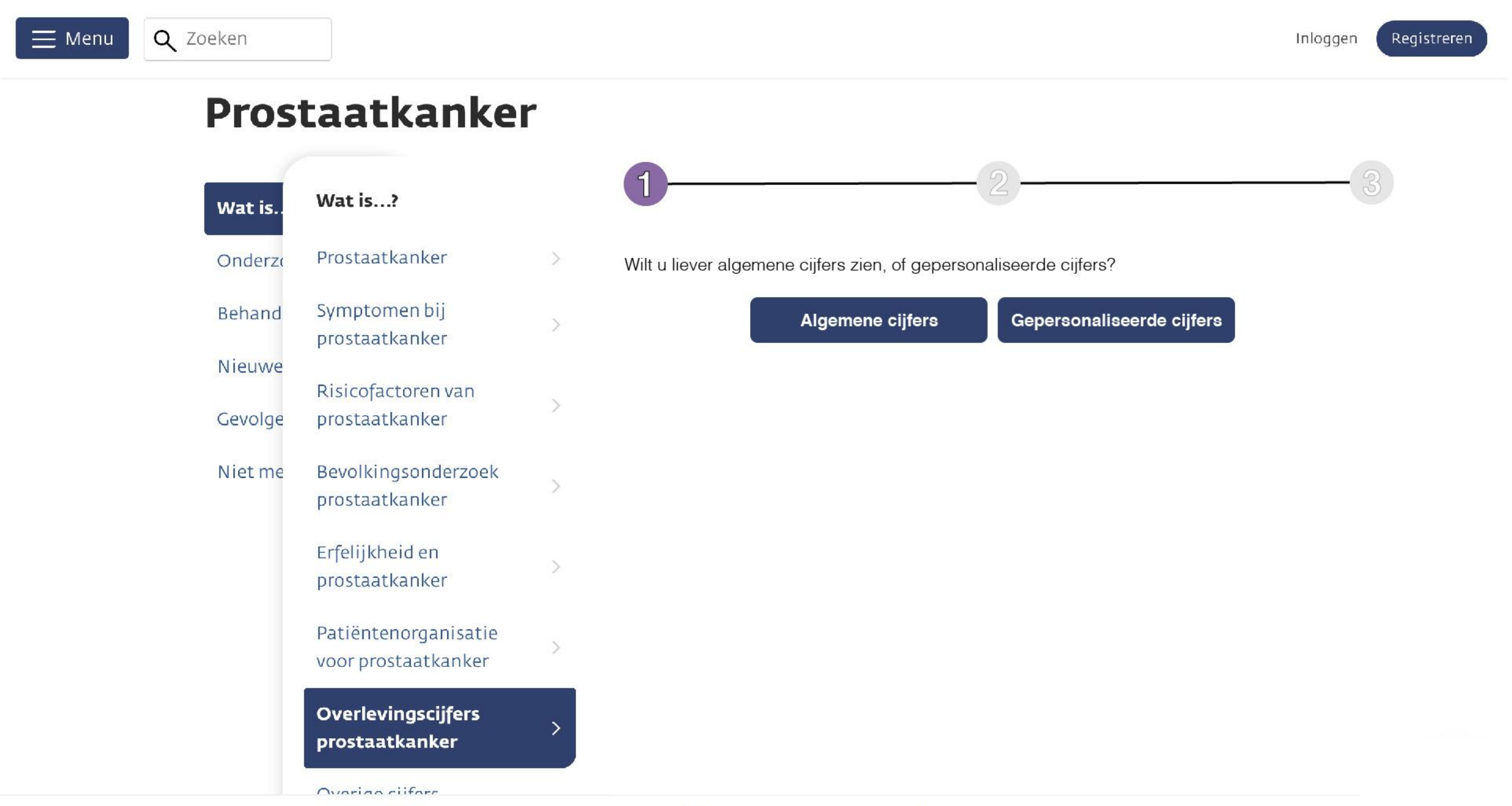

Translation:
Would you like to see generic statistics, or personalized statistics?

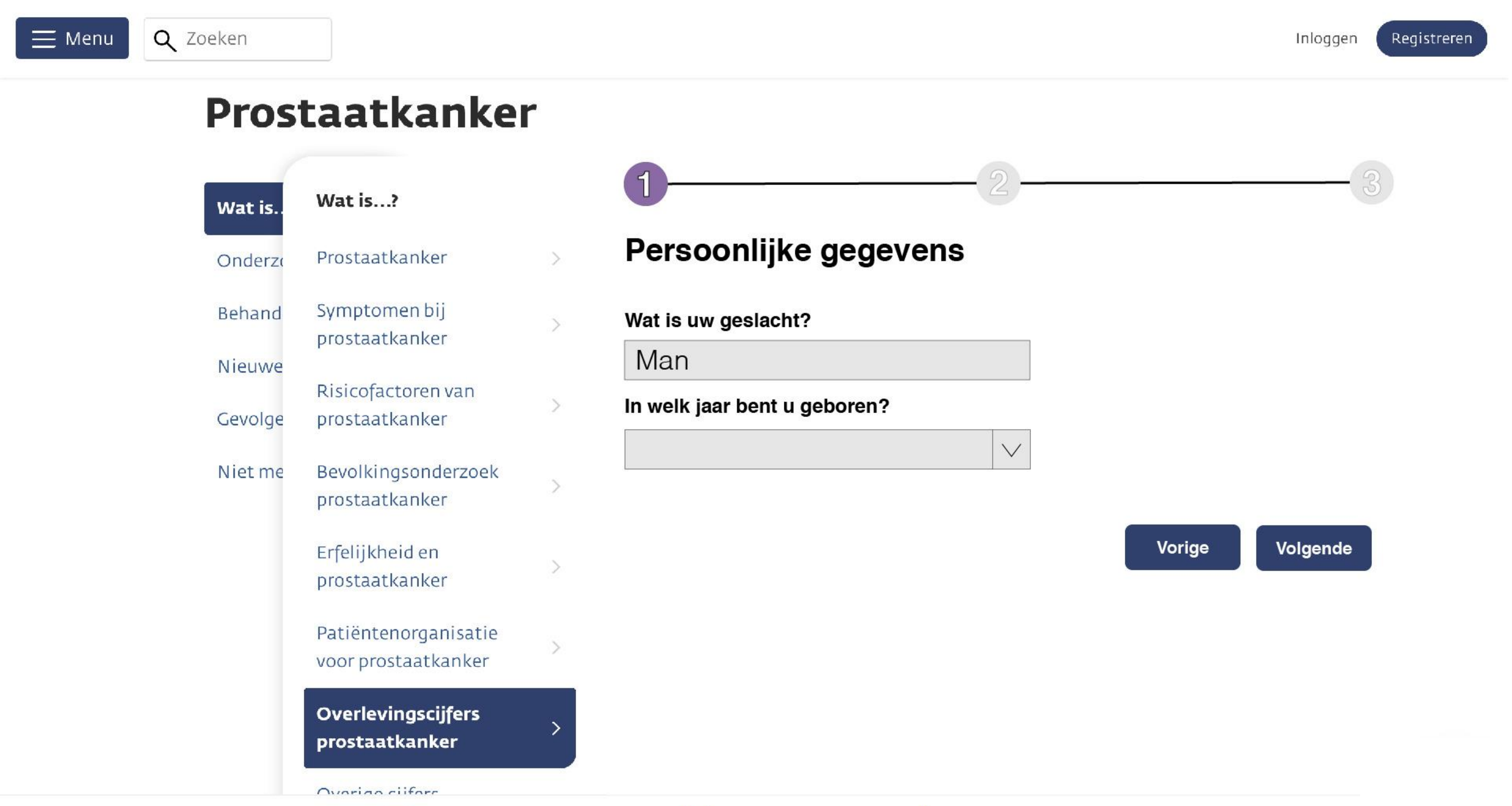

Translation:

Personal data

- What is your gender? (men)
- What is your year of birth?

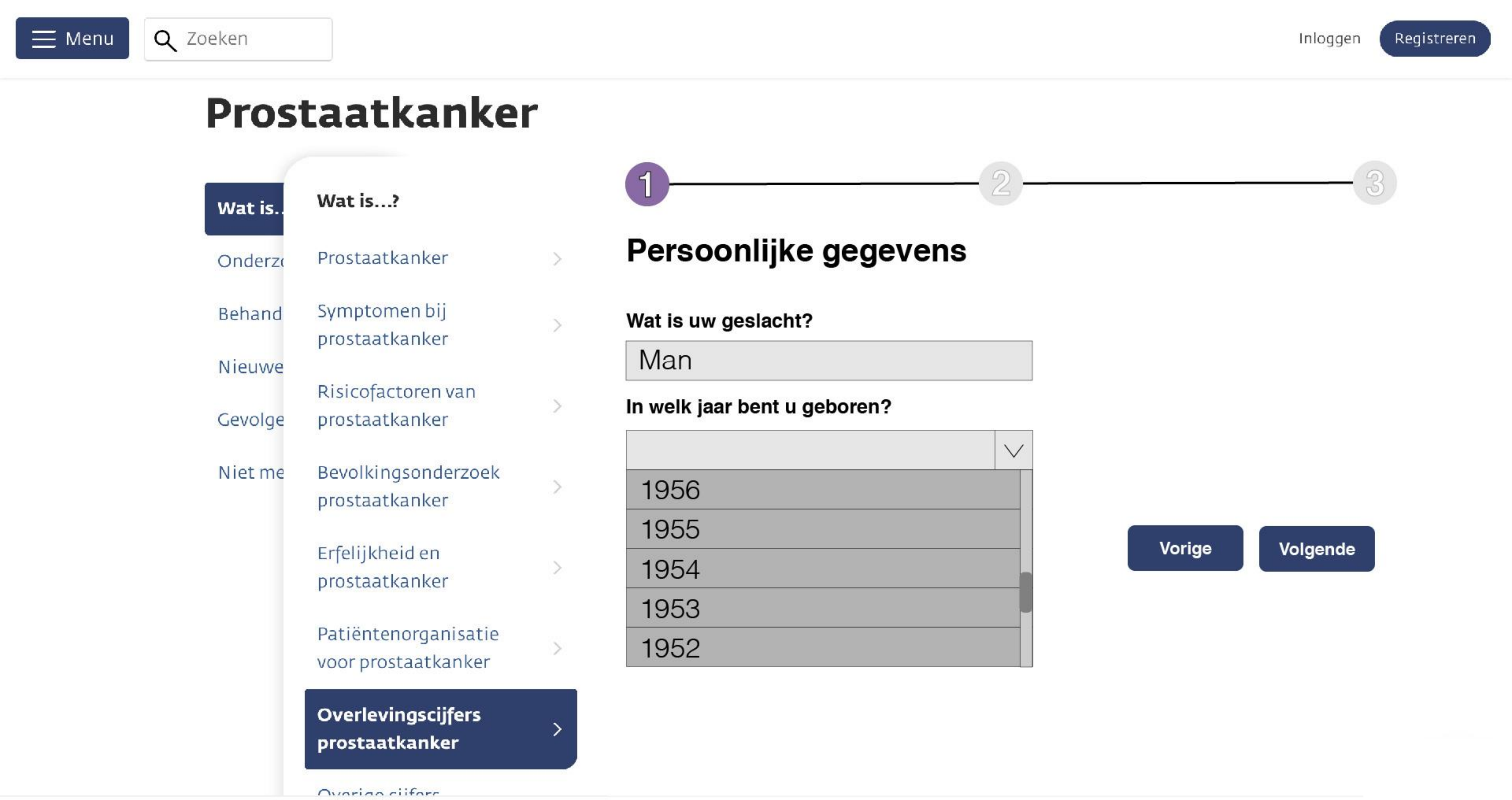

Translation:

Personal data

- What is your gender? (men)
- What is your year of birth?

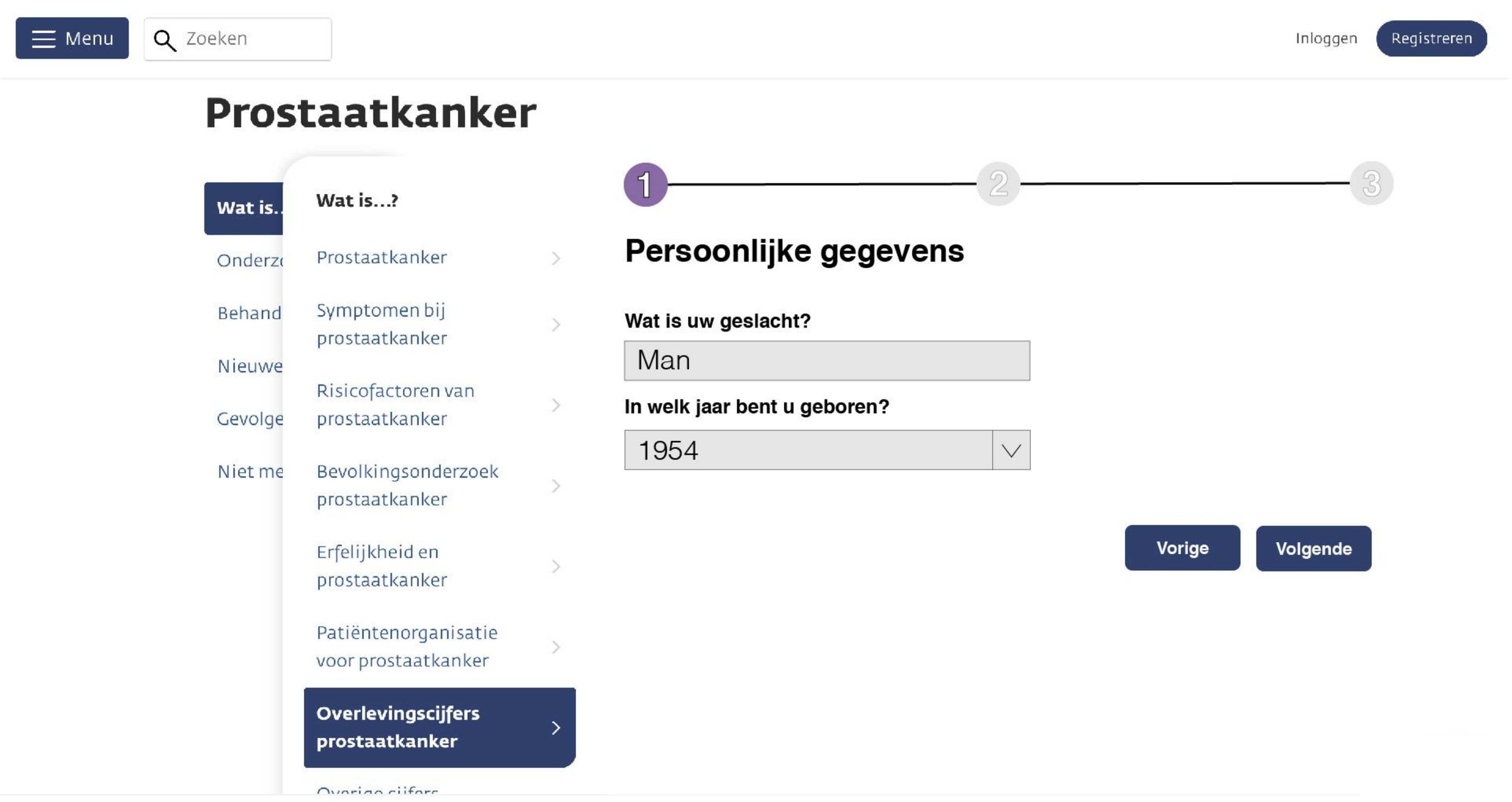

Translation:

Personal data

- What is your gender? (men)
- What is your year of birth?

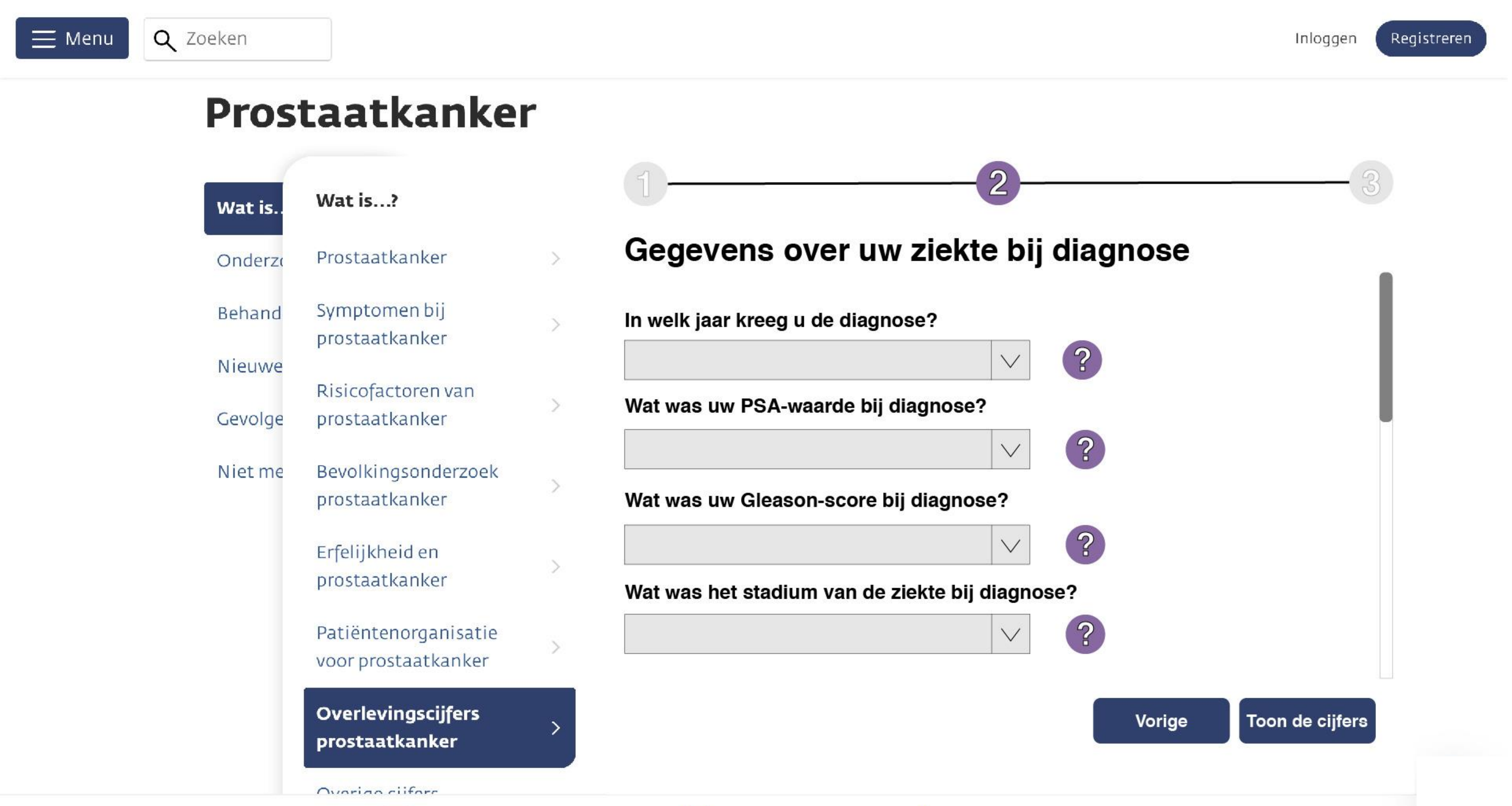

Translation:

Disease-related data

- Year of diagnosis?
- PSA-value at diagnosis?
- Gleason-score at diagnosis?
- Tumor stage at diagnosis?

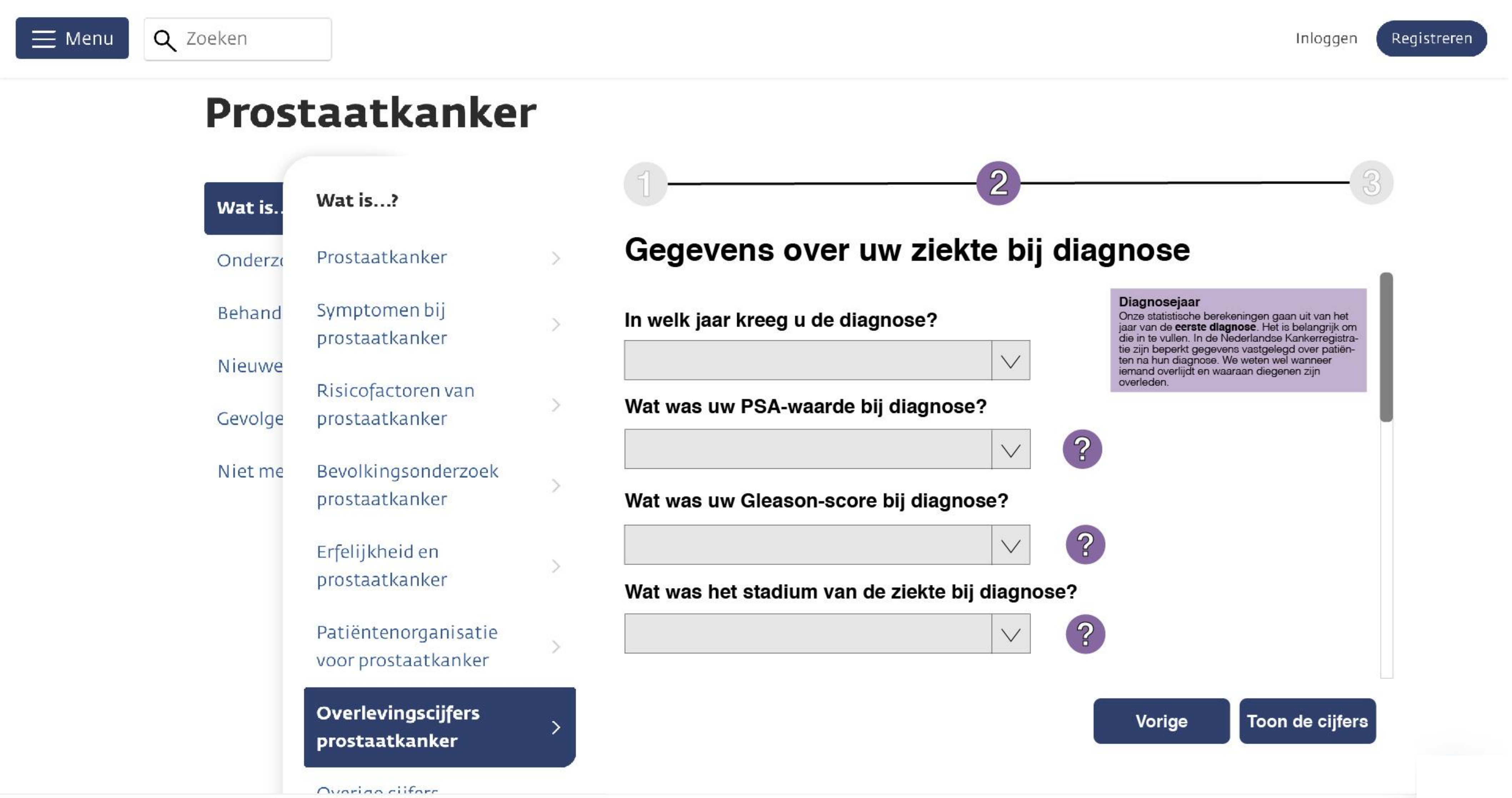

Translation:
Our calculations are based on the first cancer diagnosis. It is important to fill out this one, because the Netherlands Cancer Registry contains limited data of patients after their diagnosis. However, the NCR does know when a patient died (and its cause).

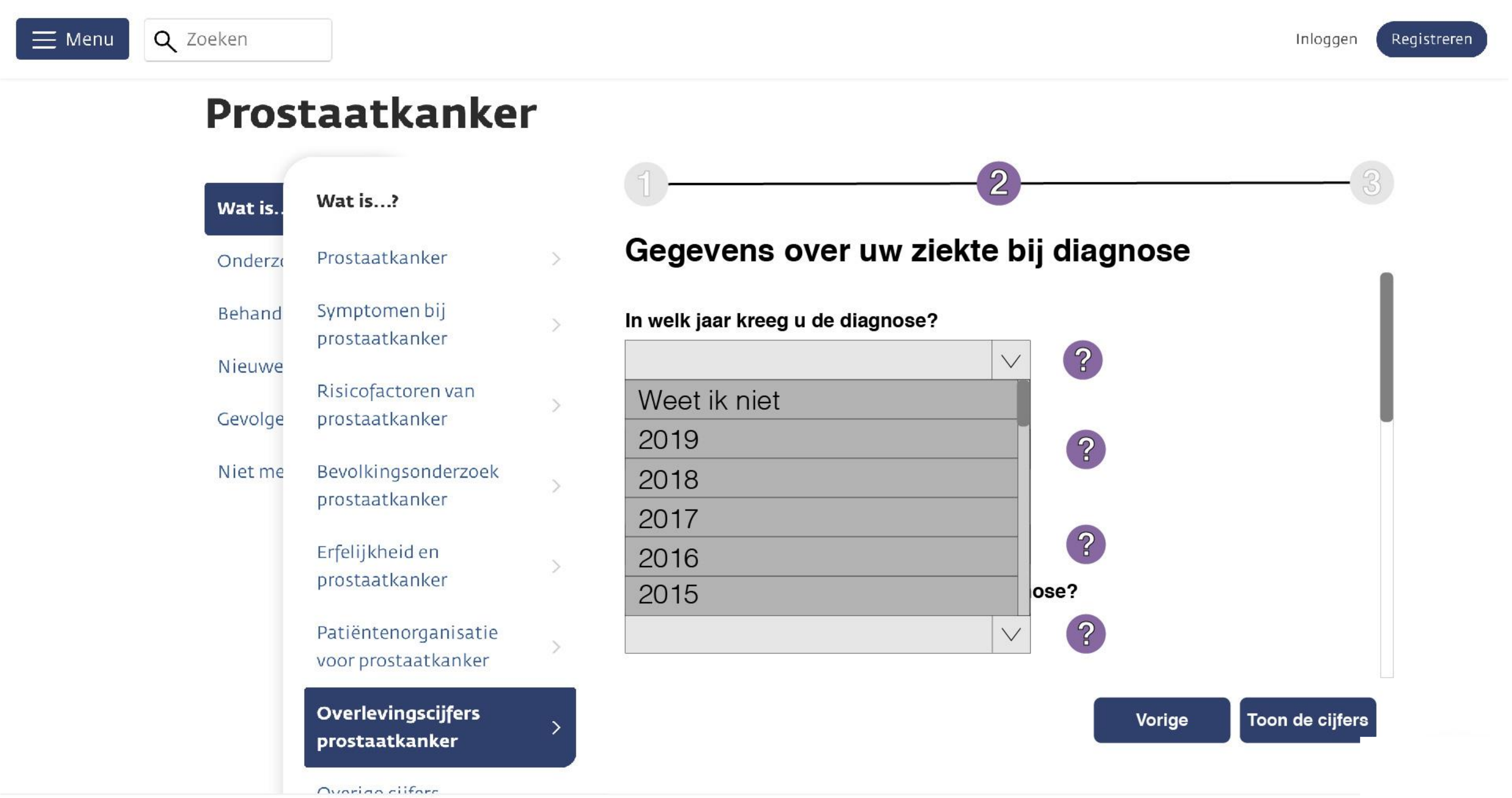

Translation:

Disease-related data

- Year of diagnosis (I don’t know, 2019, 2018, etc.)

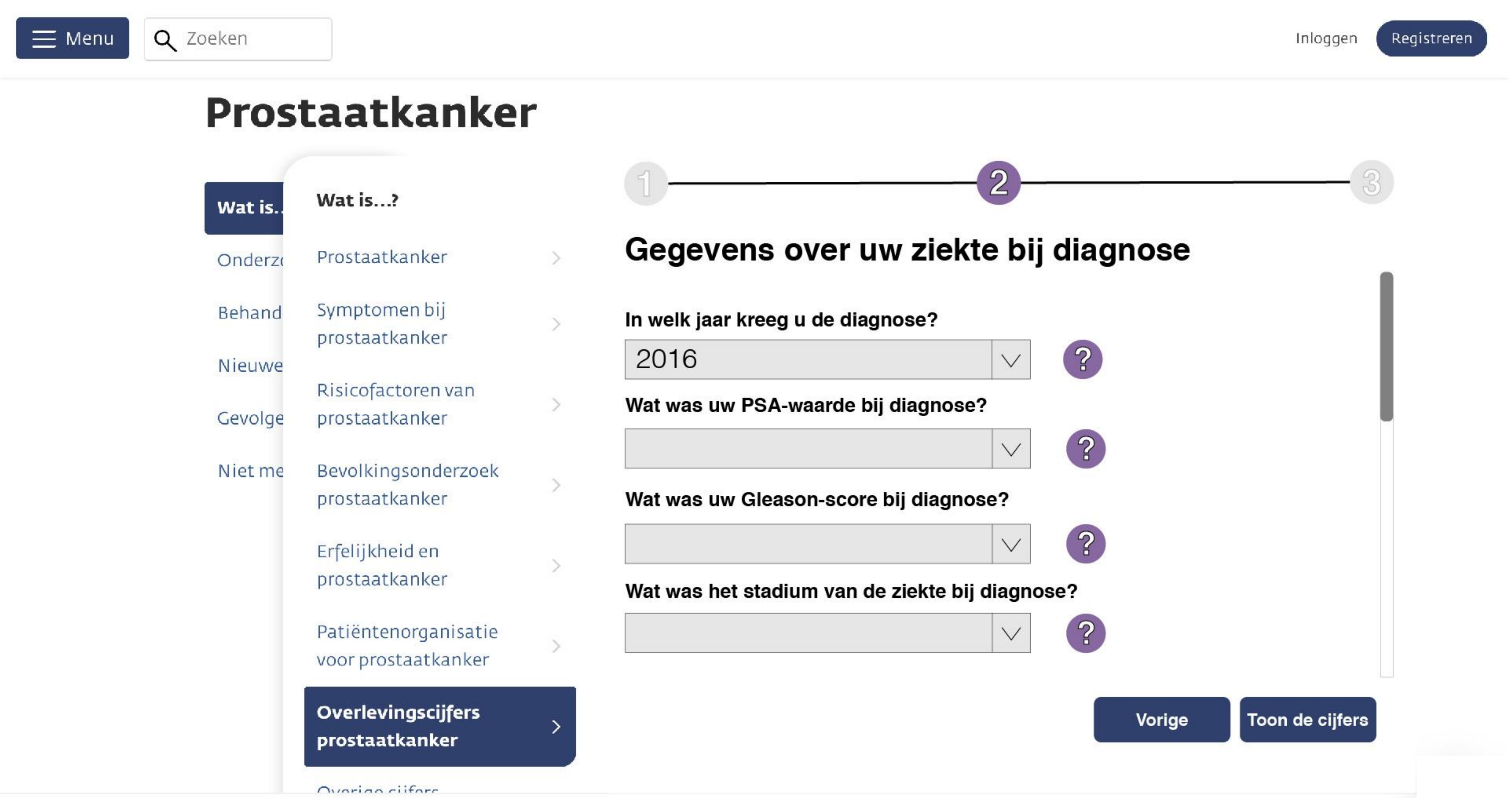

Translation:

Disease-related data

- Year of diagnosis? (2016)
- PSA-value at diagnosis?
- Gleason-score at diagnosis?
- Tumor stage at diagnosis?

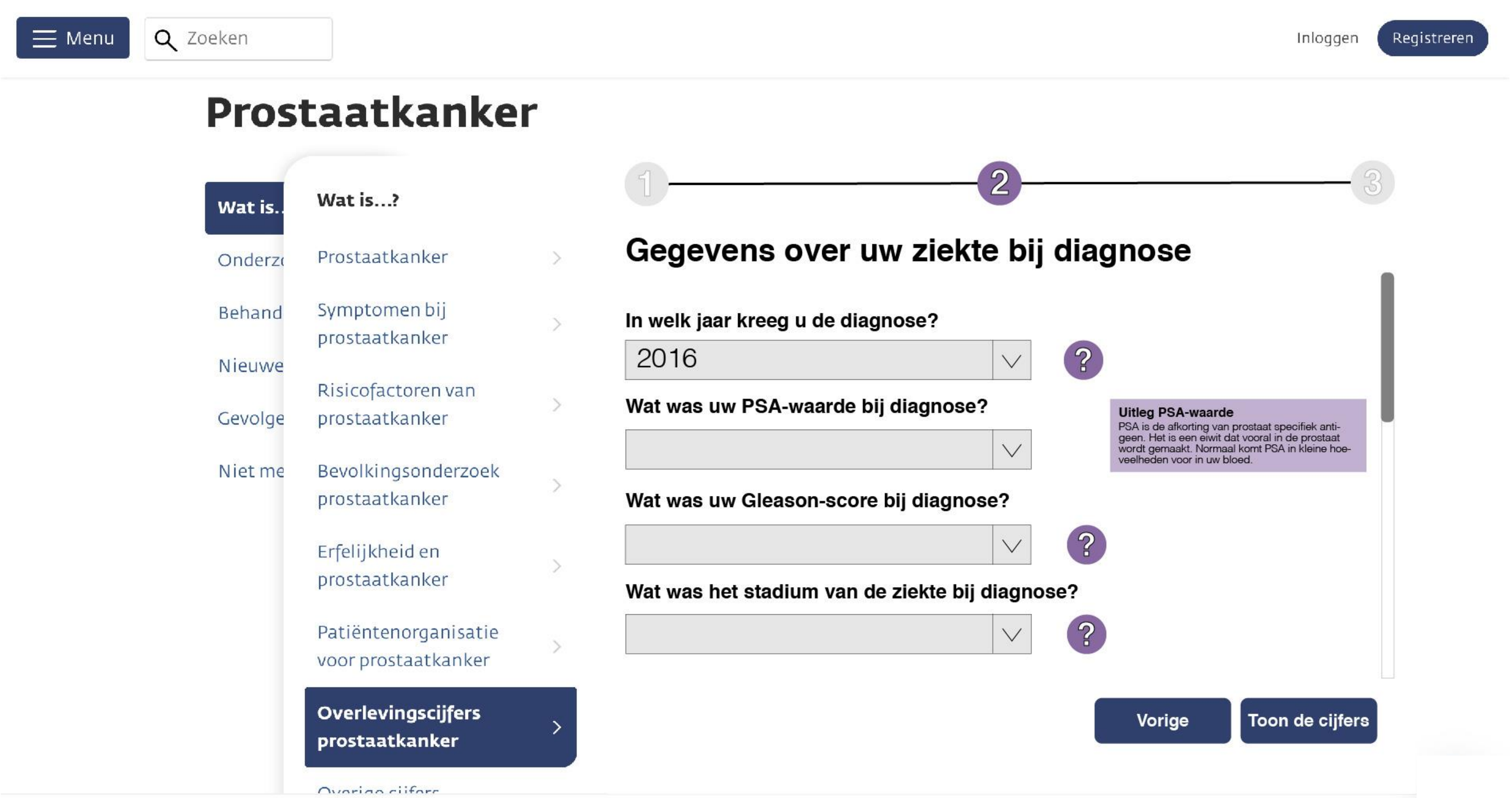

Translation:
Explanation PSA-value: PSA stands for Prostate-specific antigen. This is a protein produced by normal, as well as malignant, cells of the prostate gland.

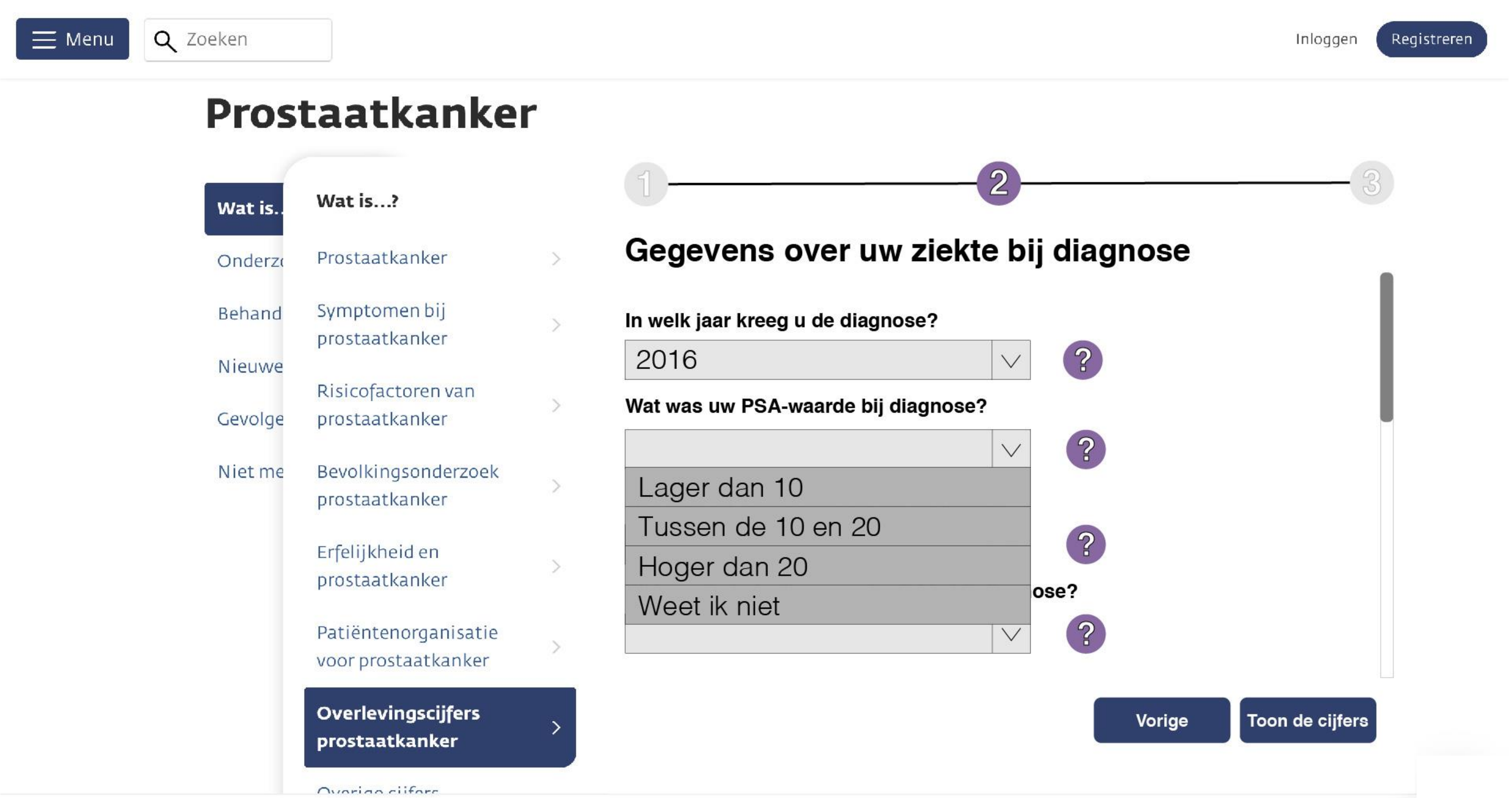

Translation:

Disease-related data

- Year of diagnosis? (2016)
- PSA-value at diagnosis? (Lower than 10, Between 10 and 20, Higher than 20, I don’t know)
- Gleason-score at diagnosis?
- Tumor stage at diagnosis?

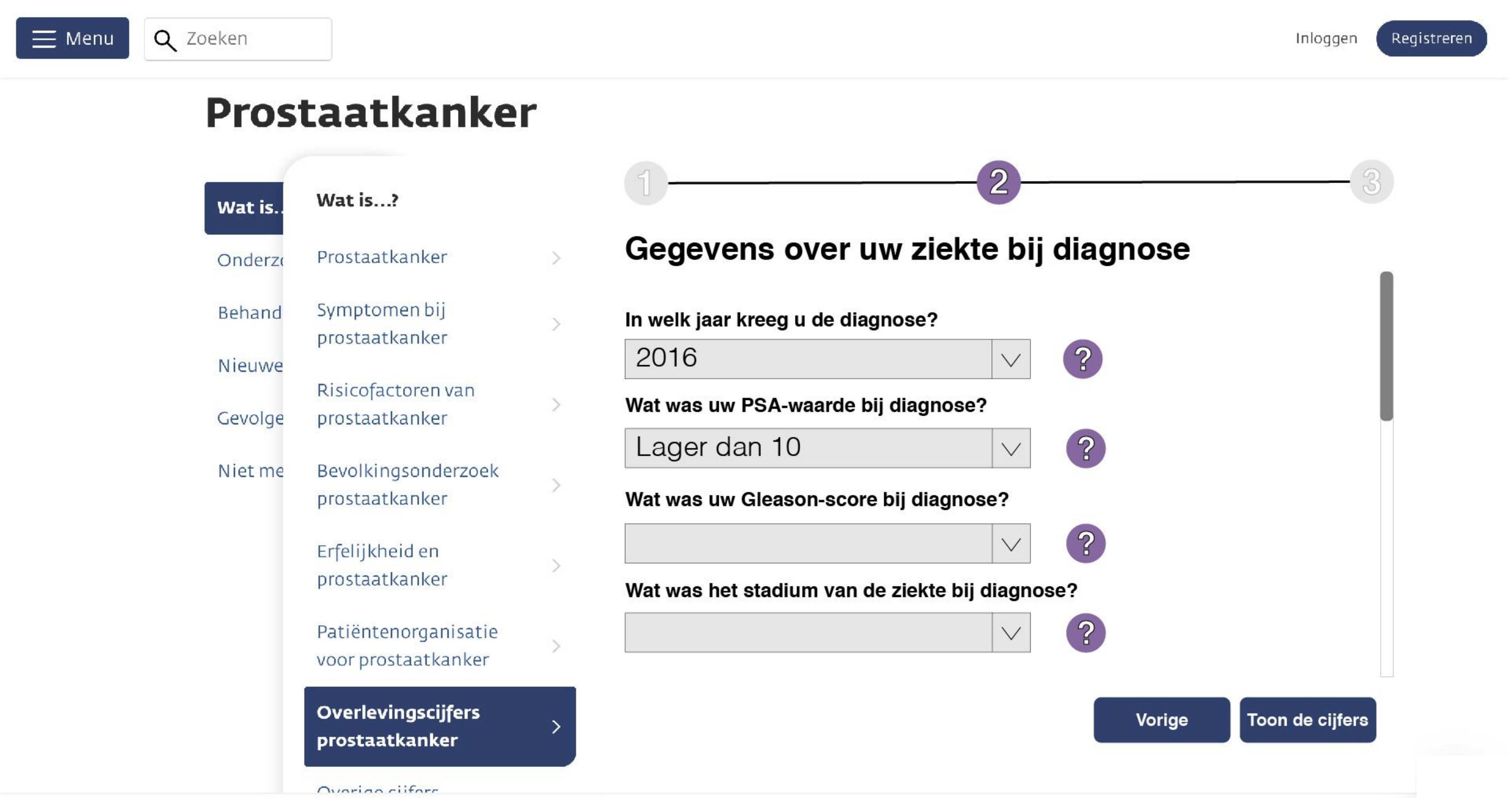

Translation:

Disease-related data

- Year of diagnosis? (2016)
- PSA-value at diagnosis? (Lower than 10)
- Gleason-score at diagnosis?
- Tumor stage at diagnosis?

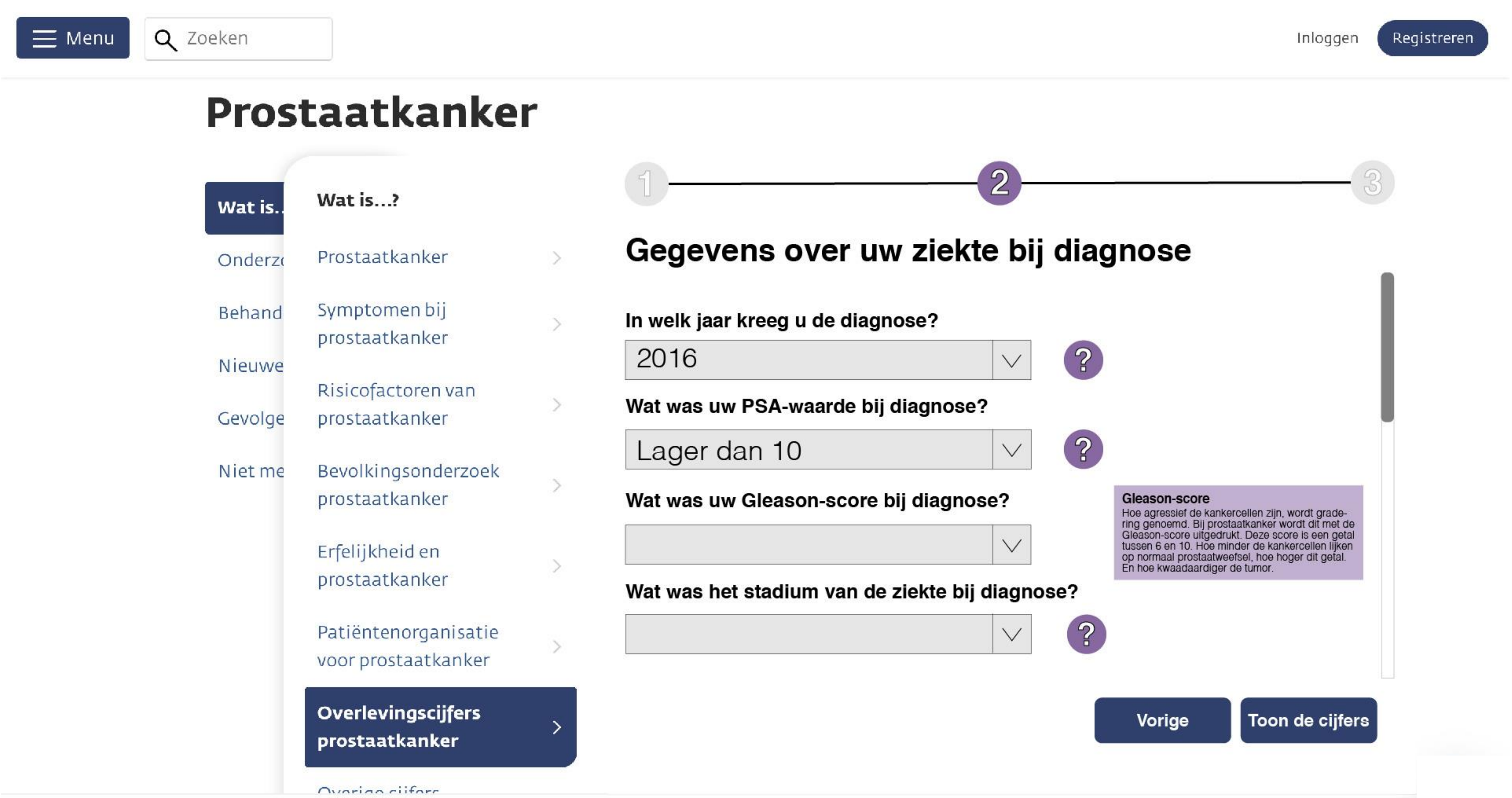

Translation:

Gleason-score: The tumor grade says something about the aggressiveness of the tumor. For prostate cancer, this is expressed by means of the Gleason-score. This score is a number between 6 and 10. Cancer cells that deviate from healthy cells receive a high score. The higher this score, the more aggressive the tumor is.

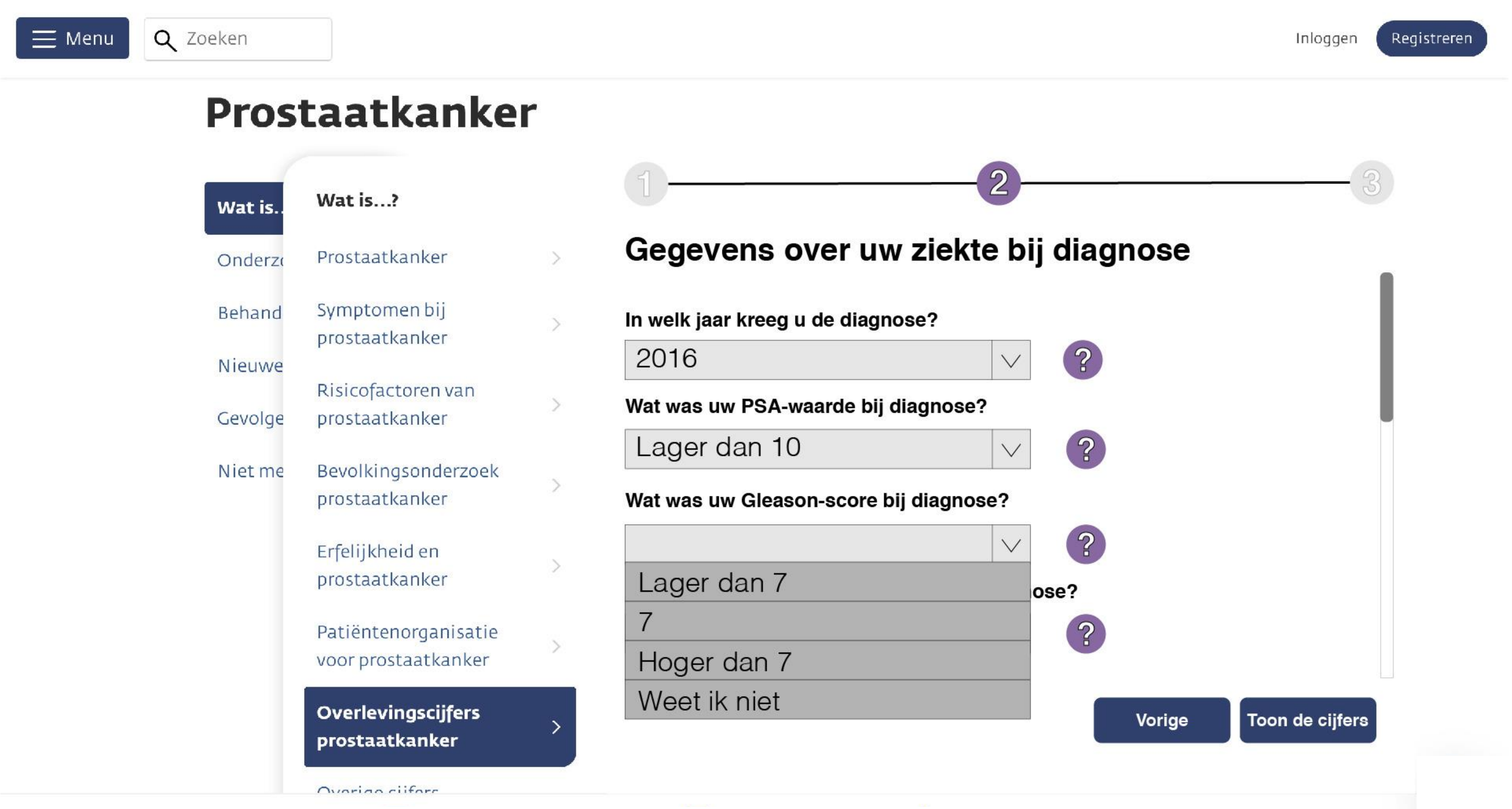

Translation:

Disease-related data

- Year of diagnosis? (2016)
- PSA-value at diagnosis? (Lower than 10)
- Gleason-score at diagnosis? (Lower than 7, 7, Higher than 7, I don’t know)
- Tumor stage at diagnosis?

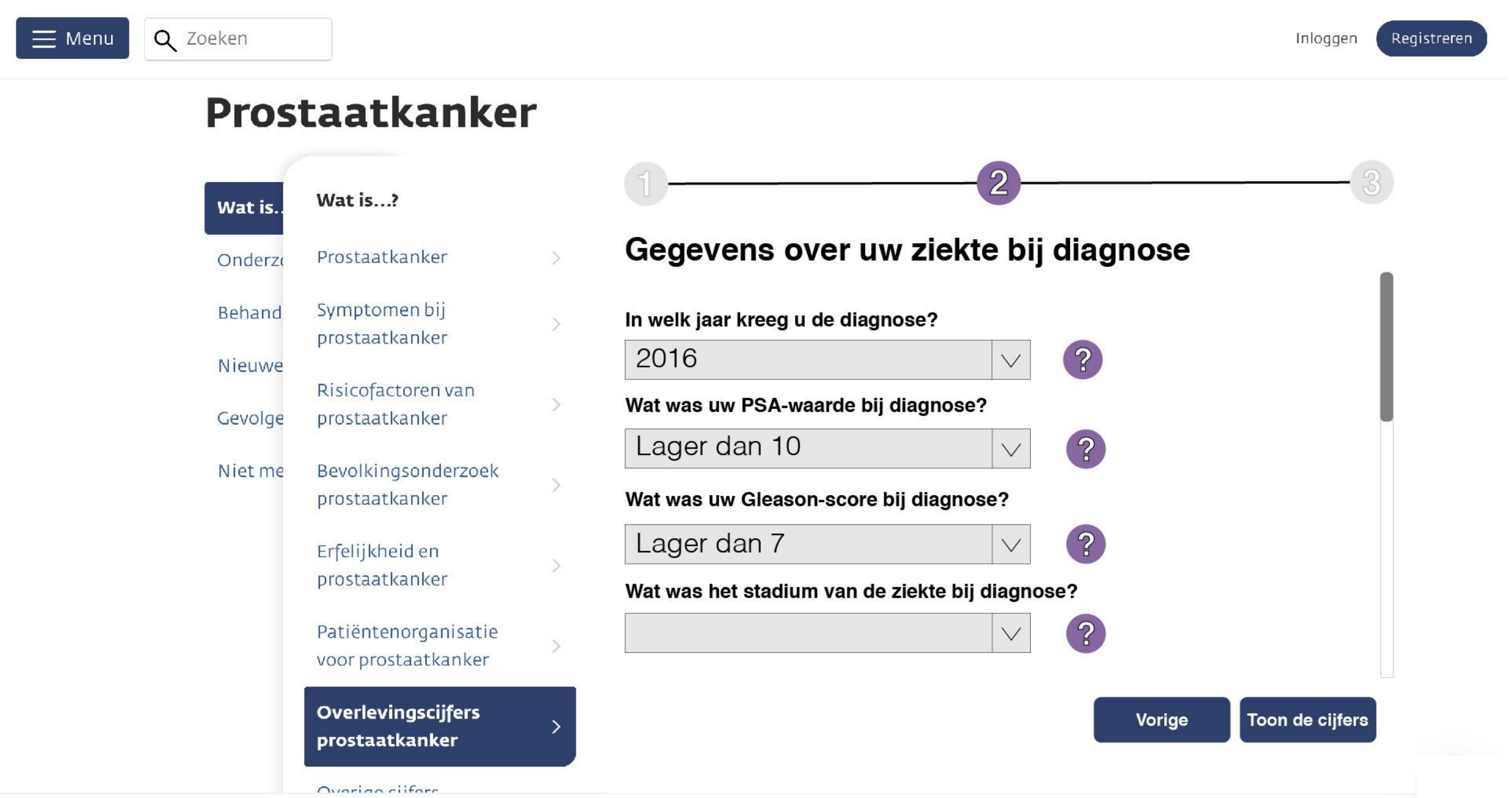

Translation:

Disease-related data

- Year of diagnosis? (2016)
- PSA-value at diagnosis? (Lower than 10
- Gleason-score at diagnosis? (Lower than 7)
- Tumor stage at diagnosis?

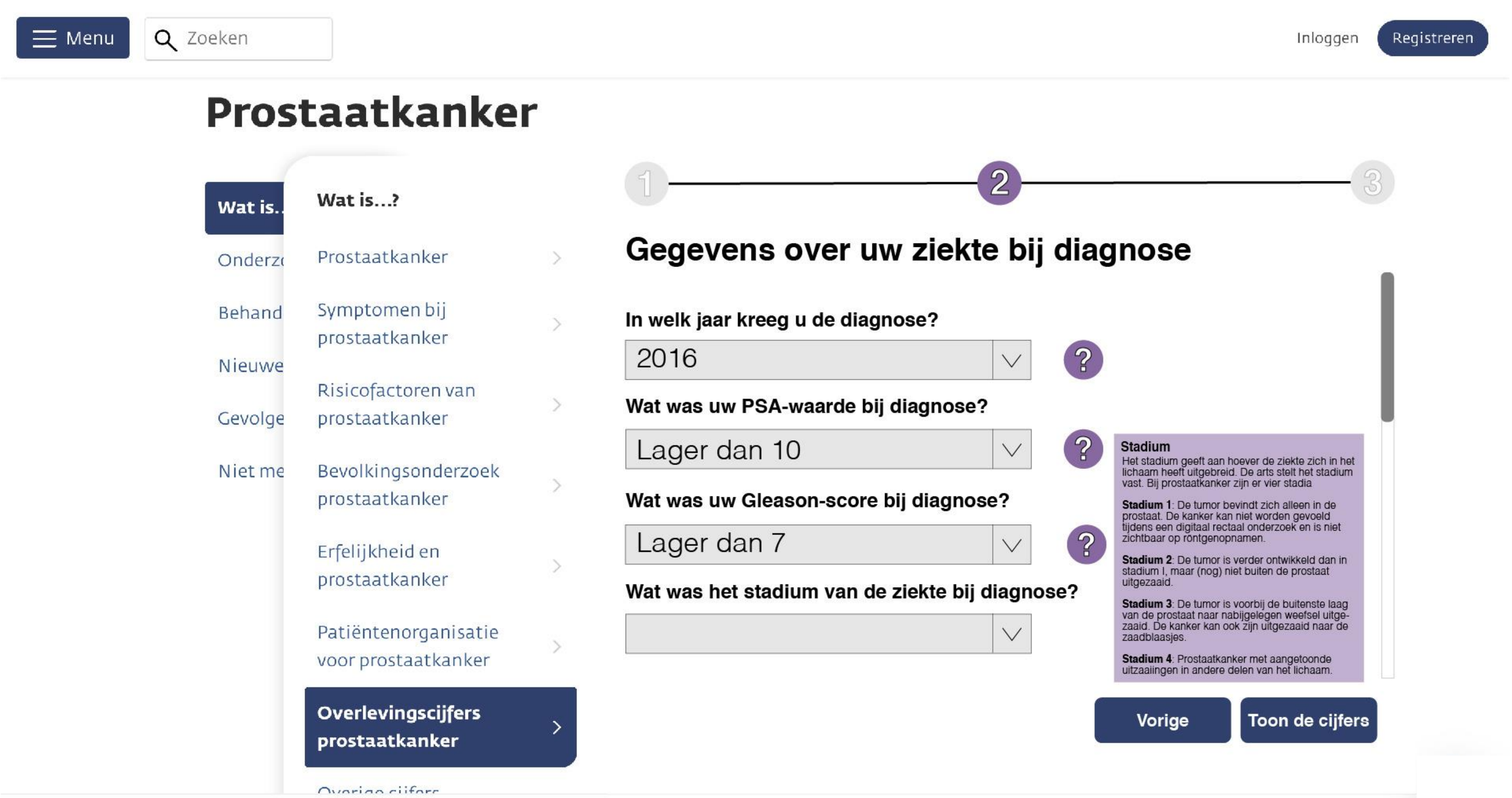

Translation:
The tumor stage says something about how far the cancer has progressed. The doctor will determine the tumor stage. For prostate cancer, there are four stages.
Stage 1: the tumor is still within the prostate. The doctor cannot feel the tumor during a rectal exam, or the tumor is not visible on a scan.
Stage 2: the tumor remains confined to the prostate gland, but it has not spread outside the prostate.
Stage 3: the tumor is locally advanced. The tumor has progressed and is more likely to grow and spread.
Stage 4: the tumor has spread to lymph nodes or to other parts of the body.

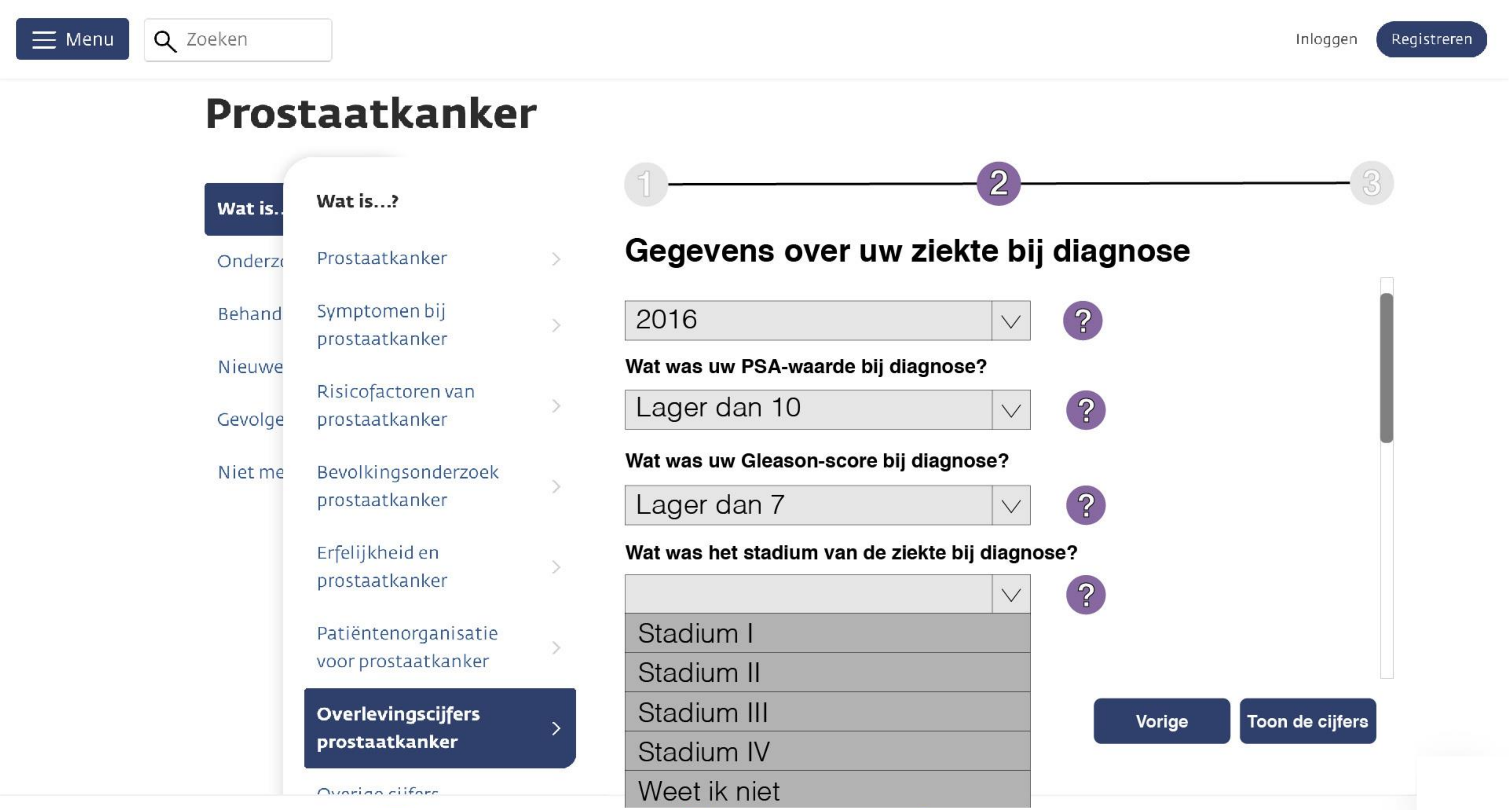

Translation:

Disease-related data

- Year of diagnosis? (2016)
- PSA-value at diagnosis? (Lower than 10
- Gleason-score at diagnosis? (Lower than 7)
- Tumor stage at diagnosis? (Stage I, Stage II, Stage III, Stage IV, I don’t know)

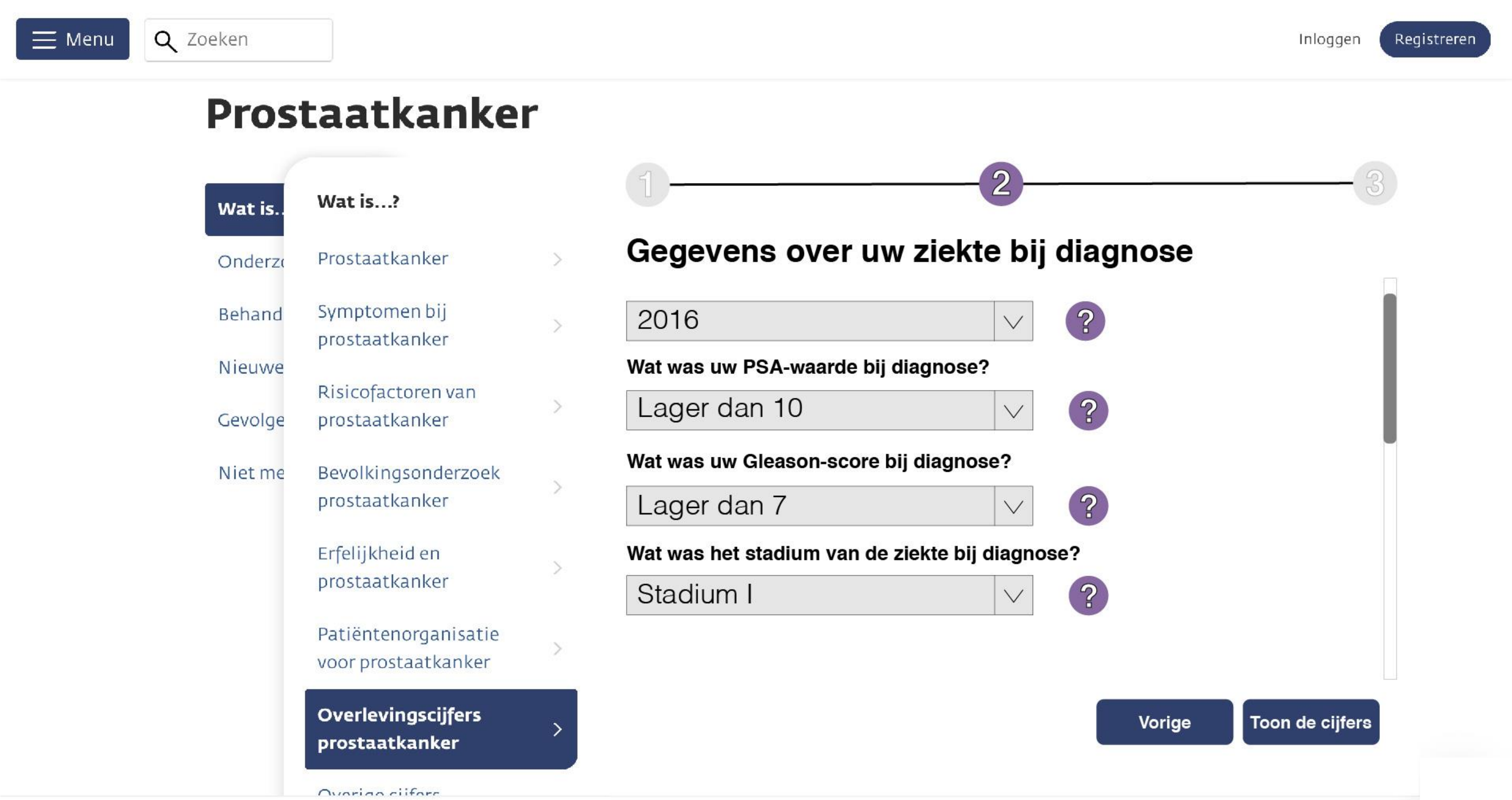

Translation:

Disease-related data

- Year of diagnosis? (2016)
- PSA-value at diagnosis? (Lower than 10
- Gleason-score at diagnosis? (Lower than 7)
- Tumor stage at diagnosis? (Stage I)

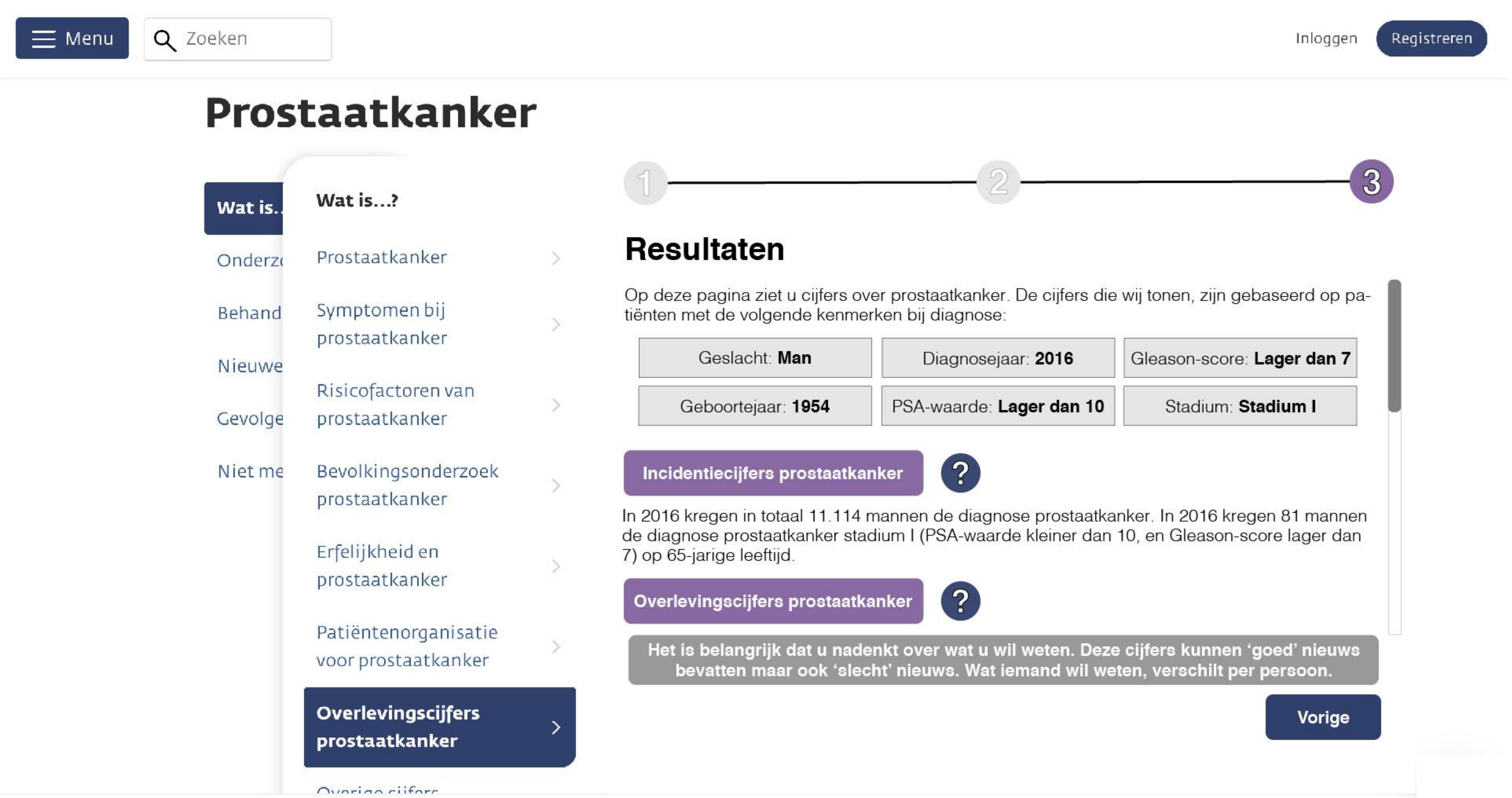

Translation:

Results

On this page, you will see statistics about prostate cancer. The statistics that we show are based on patients with the following characteristics:

Incidence rates prostate cancer: In 2016, in total 11.114 men were diagnosed with prostate cancer. In 2016, 81 men like you were diagnosed with prostate cancer stage I (PSA-value lower than 10, Gleason-score lower than 7) aged 65 years old.

Survival statistics prostate cancer

It is important that you think about what you want to know. These statistics may contain 'good' or 'bad' new. . What someone wants to know, differs per person.

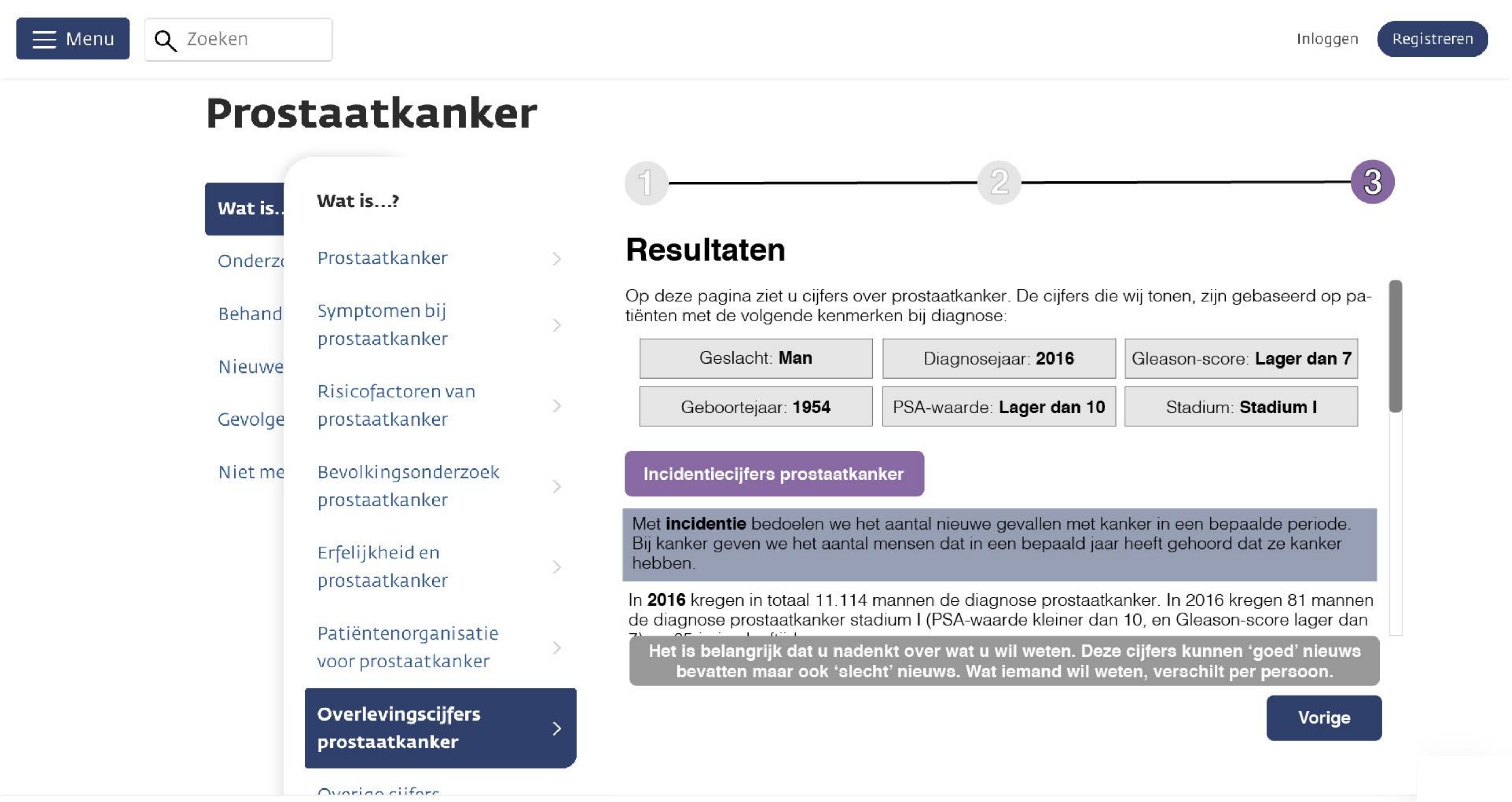

Translation:
With incidence rate we mean the number of new cancers of cancer occurring in a specified population in a certain period (typically in a specific year).

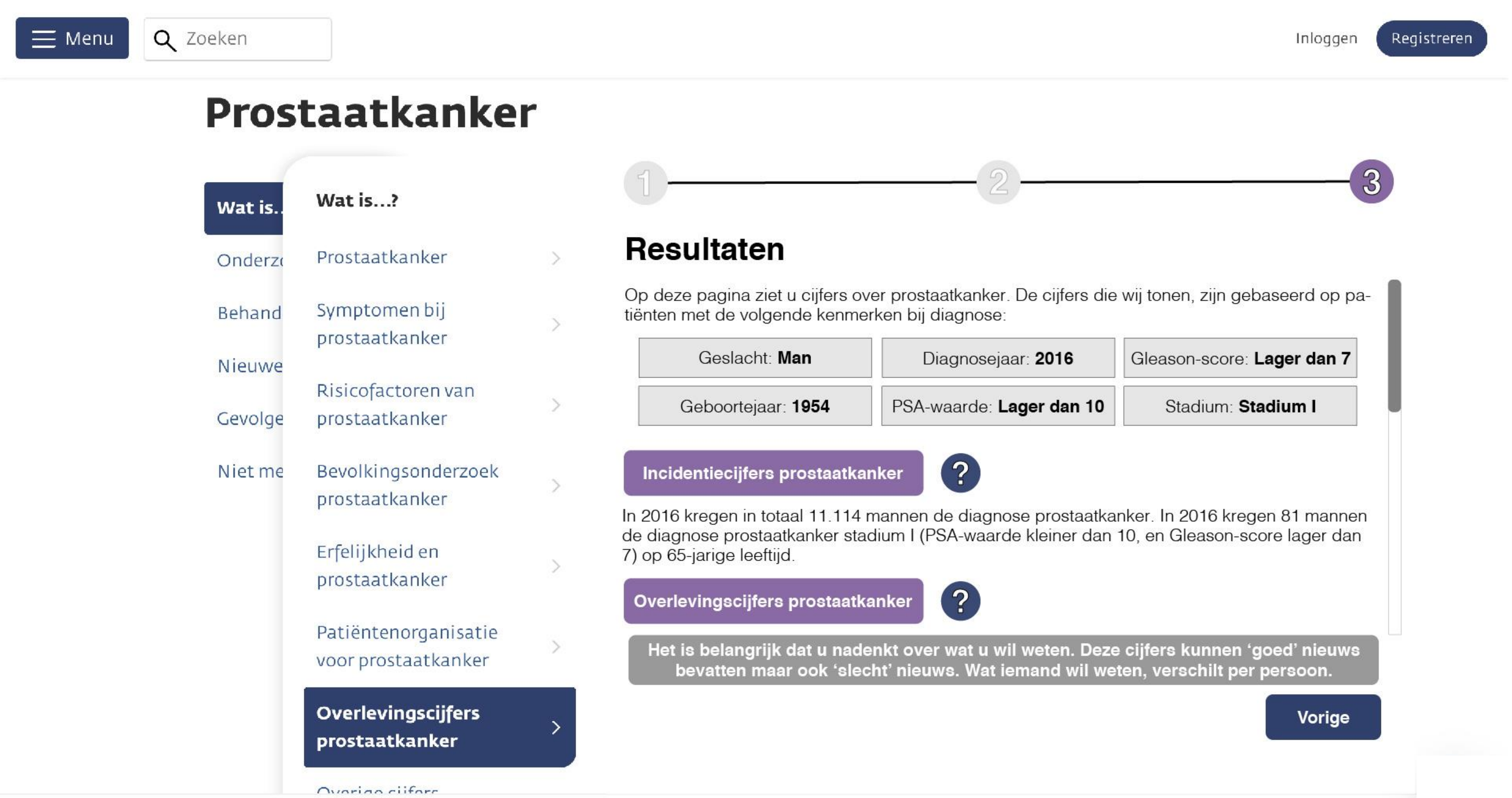

Translation:

Results

On this page, you will see statistics about prostate cancer. The statistics that we show are based on patients with the following characteristics:

Incidence rates prostate cancer: In 2016, in total 11.114 men were diagnosed with prostate cancer. In 2016, 81 men like you were diagnosed with prostate cancer stage I (PSA-value lower than 10, Gleason-score lower than 7) aged 65 years old.

Survival statistics prostate cancer

It is important that you think about what you want to know. These statistics may contain 'good' or 'bad' new. . What someone wants to know, differs per person.

Menu Zoeken Inloggen Registreren

Prostaatkanker

Wat is...
Onderz
Behand
Nieuwe
Gevolge
Niet me

Wat is...?

- Prostaatkanker
- Symptomen bij prostaatkanker
- Risicofactoren van prostaatkanker
- Bevolkingsonderzoek prostaatkanker
- Erfelijkheid en prostaatkanker
- Patiëntenorganisatie voor prostaatkanker
- **Overlevingscijfers prostaatkanker**
- Overige cijfers

1 2 3

Resultaten

Overlevingscijfers prostaatkanker ?

Algemene cijfer: 5 jaar na diagnose was ongeveer **89 procent** van de prostaatkanker patiënten nog in leven (89 van de 100 mannen).

Gepersonaliseerd cijfer: 5 jaar na diagnose was ongeveer **94 procent** van de patiënten met bovengenoemde kenmerken nog in leven (94 van de 100 mannen).

Het is belangrijk om te weten dat uw vooruitzichten nog steeds beter of slechter kunnen zijn dan de vooruitzichten voor deze specifieke groep. Bespreek uw vooruitzichten met uw arts.

Figuur

5 jaar na diagnose

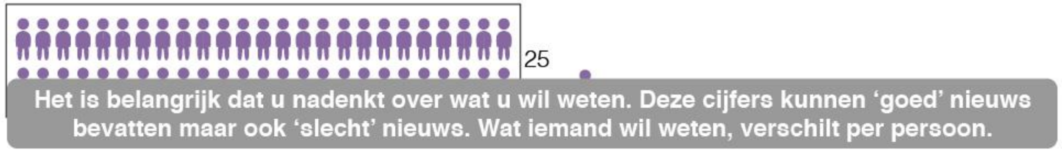

Het is belangrijk dat u nadenkt over wat u wil weten. Deze cijfers kunnen 'goed' nieuws bevatten maar ook 'slecht' nieuws. Wat iemand wil weten, verschilt per persoon.

Vorige

Translation:
Survival statistics prostate cancer
Generic statistic: 5 years after diagnosis, about 89 percent of these patients with prostate cancer are alive (89 out of 100 men).
Personalized statistic: 5 years after diagnosis, about **94 percent** of these patients like you with prostate cancer are alive (89 out of 100 men).
It is important to know that your prognosis may be better or worse than the prognosis of this specific group of patients. Please discuss your prognostic situation with your doctor.

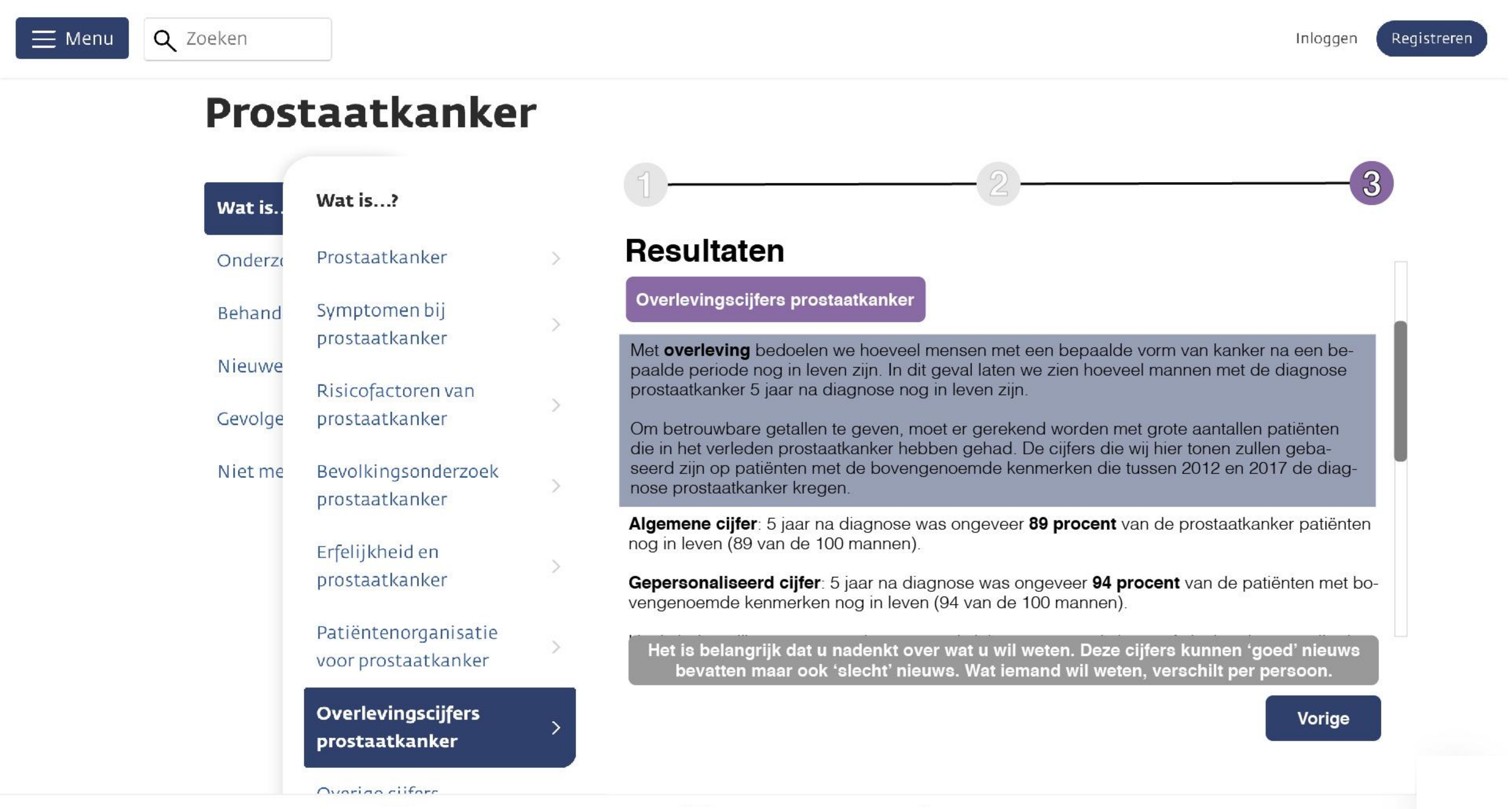

Translation:
With survival we mean the percentage of people with cancer still alive after a particular amount of time. Survival statistics for cancer are usually written as 5 year survival. In order to provide you with reliable statistics we need to perform statistics on a large group of patients that have been diagnosed with prostate cancer in the past. The statistics that we show here are performed on data from patients diagnosed between 2012 and 2017.

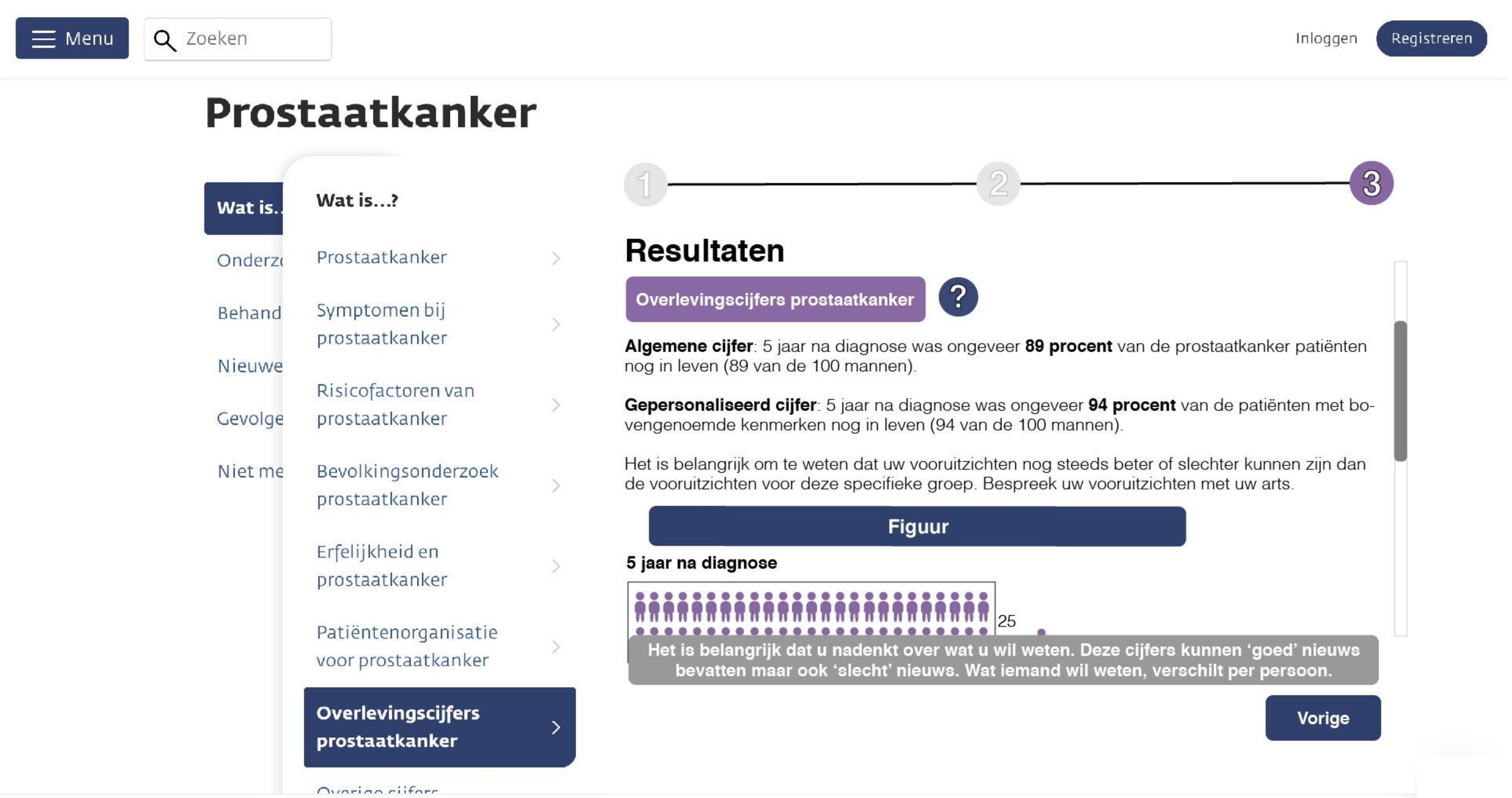

Translation:

Survival statistics prostate cancer

Generic statistic: 5 years after diagnosis, about **89 percent** of these patients with prostate cancer are alive (89 out of 100 men).

Personalized statistic: 5 years after diagnosis, about **94 percent** of these patients like you with prostate cancer are alive (89 out of 100 men).

It is important to know that your prognosis may be better or worse than the prognosis of this specific group of patients. Please discuss your prognostic situation with your doctor.

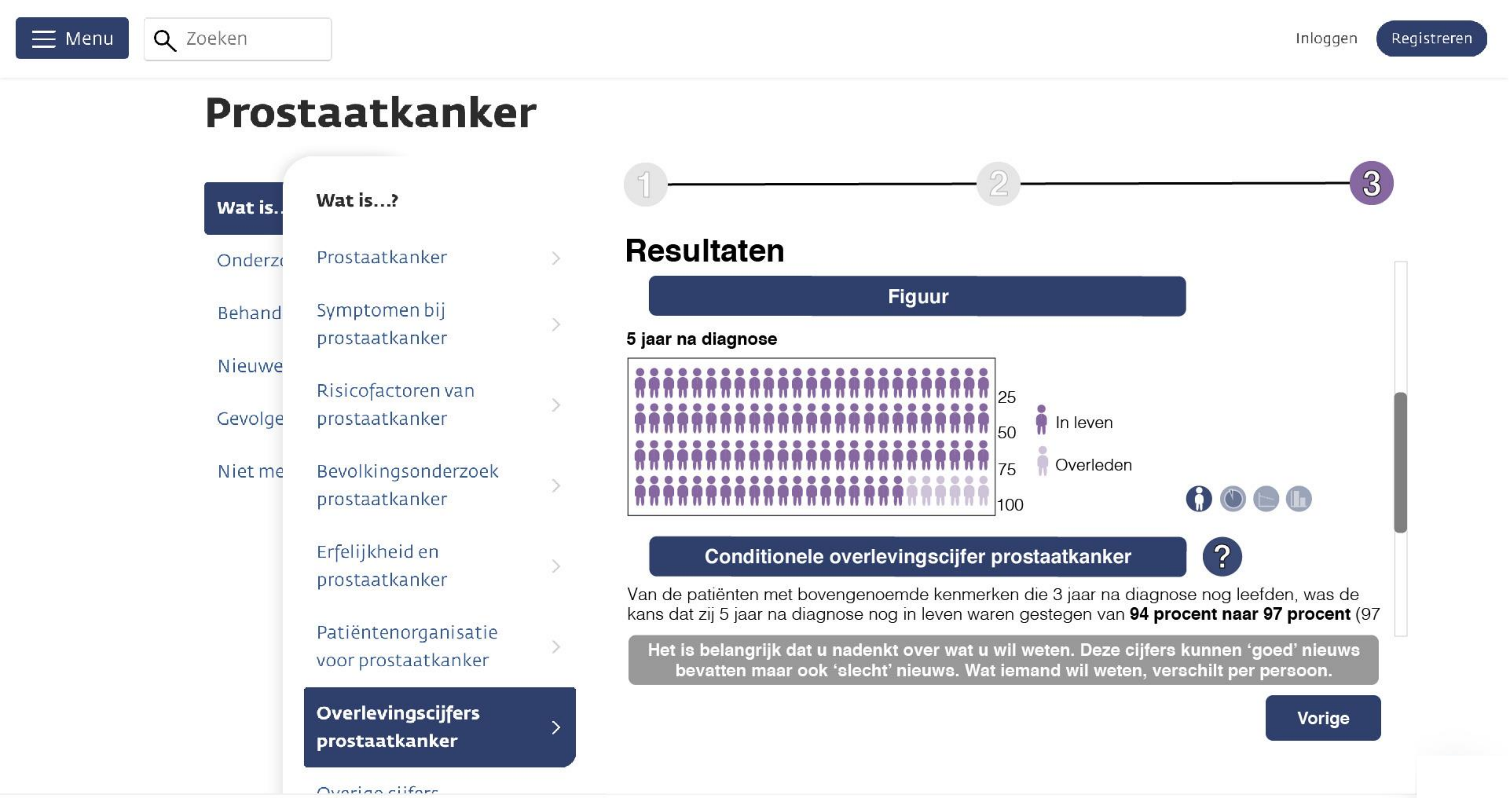

Translation:

Conditional survival statistics prostate cancer

Of those patients like you who were still alive 3 years after diagnosis, the chance that they would still be alive 5 years after diagnosis increased from **94 percent to 97 percent** (97 out of 100 men).

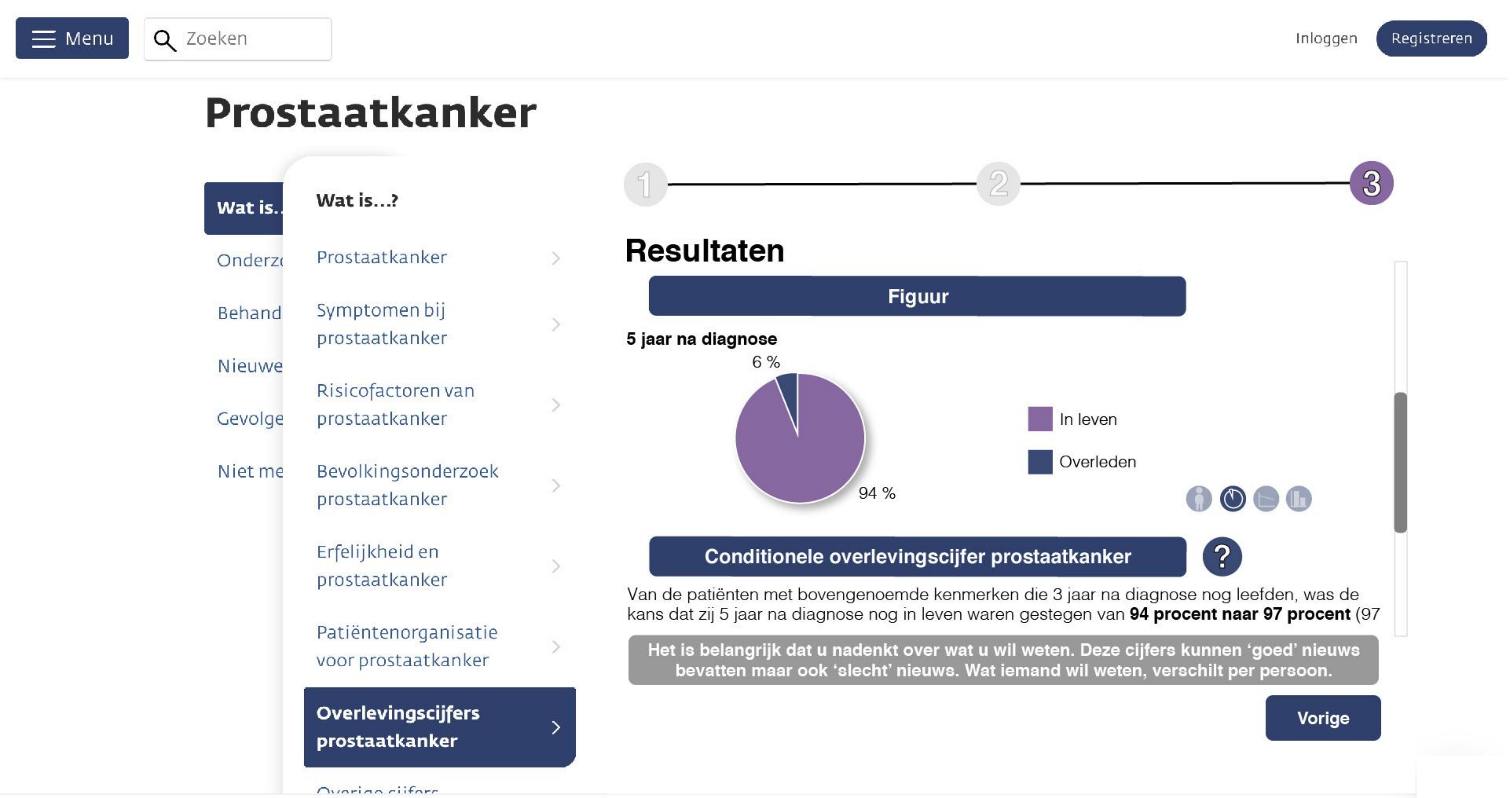

Translation:

Conditional survival statistics prostate cancer

Of those patients like you who were still alive 3 years after diagnosis, the chance that they would still be alive 5 years after diagnosis increased from **94 percent to 97 percent** (97 out of 100 men).

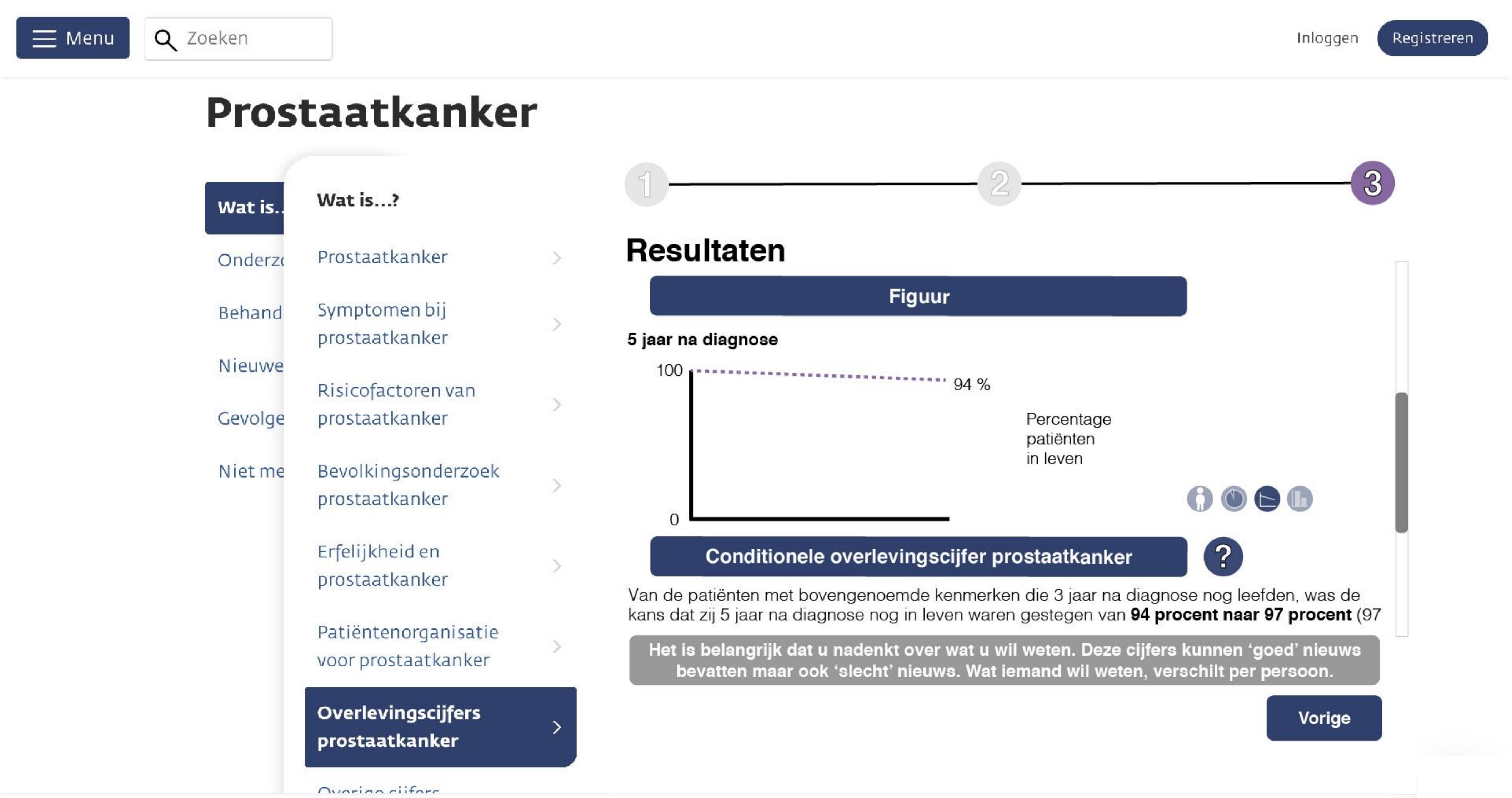

Translation:

Conditional survival statistics prostate cancer

Of those patients like you who were still alive 3 years after diagnosis, the chance that they would still be alive 5 years after diagnosis increased from **94 percent to 97 percent** (97 out of 100 men).

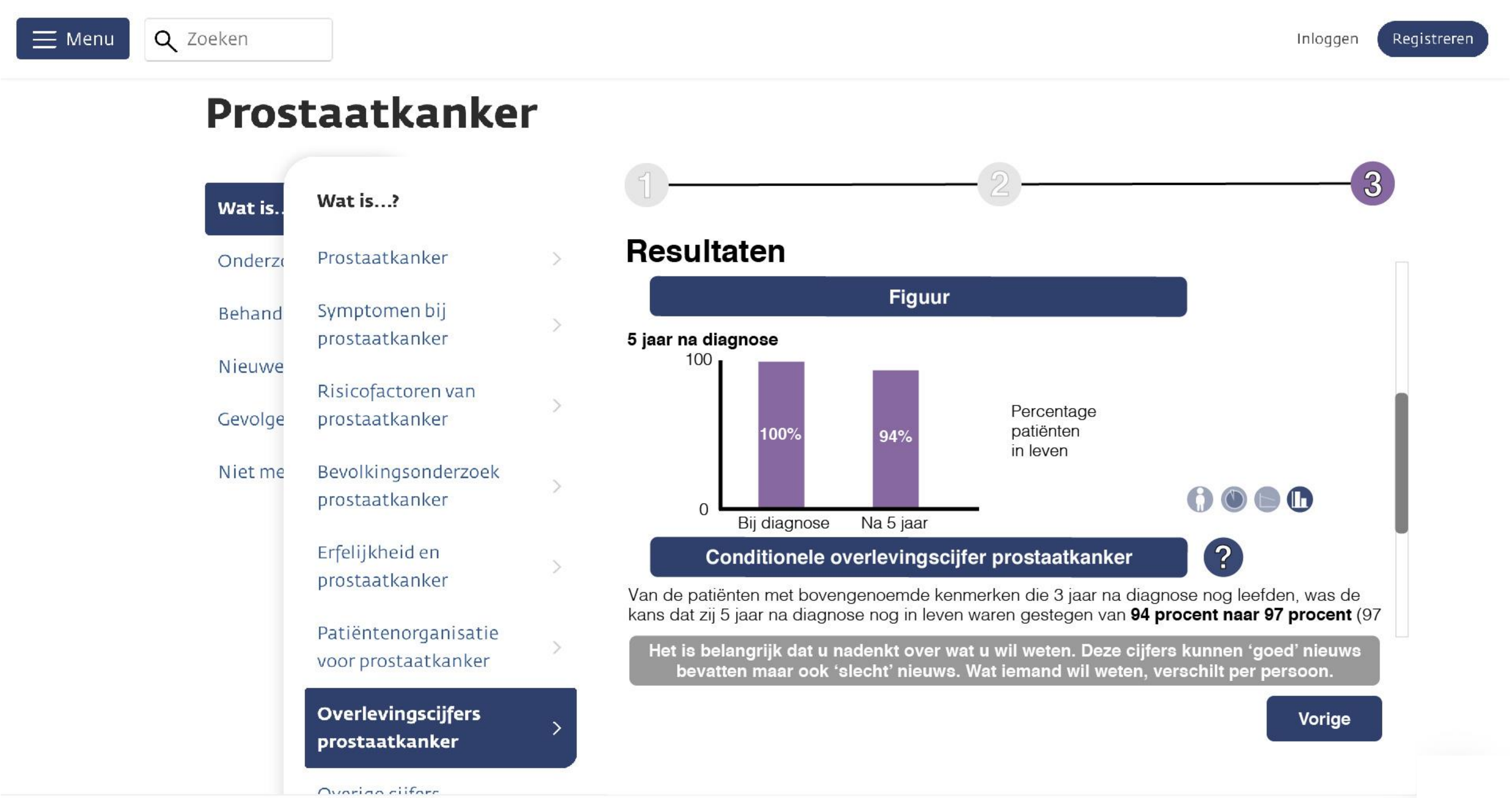

Translation:
Conditional survival statistics prostate cancer
Of those patients like you who were still alive 3 years after diagnosis, the chance that they would still be alive 5 years after diagnosis increased from **94 percent to 97 percent** (97 out of 100 men).

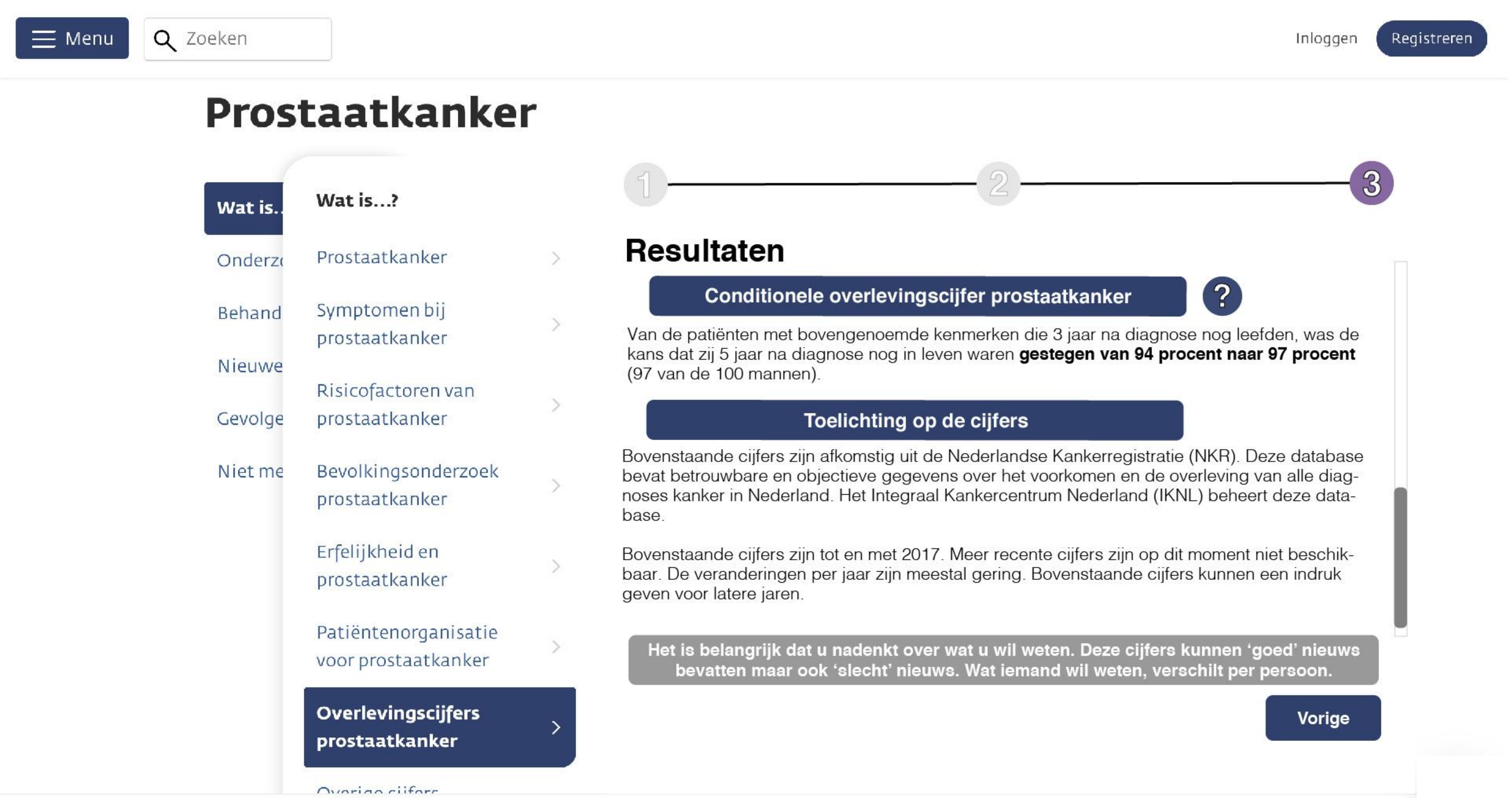

Translation:

About the statistics

The statistics that we show are based on data from the Netherlands Cancer Registry (NCR). This database contains reliable and objective data about the prevalence and survival of all cancer patients in the Netherlands. The Netherlands Comprehensive Cancer Organisation hosts this database.

The statistics that we showed are until 2017. More recent statistics are currently not available. However, annual changes are typically limited. The statistics provided here may therefore give an impression for future years.

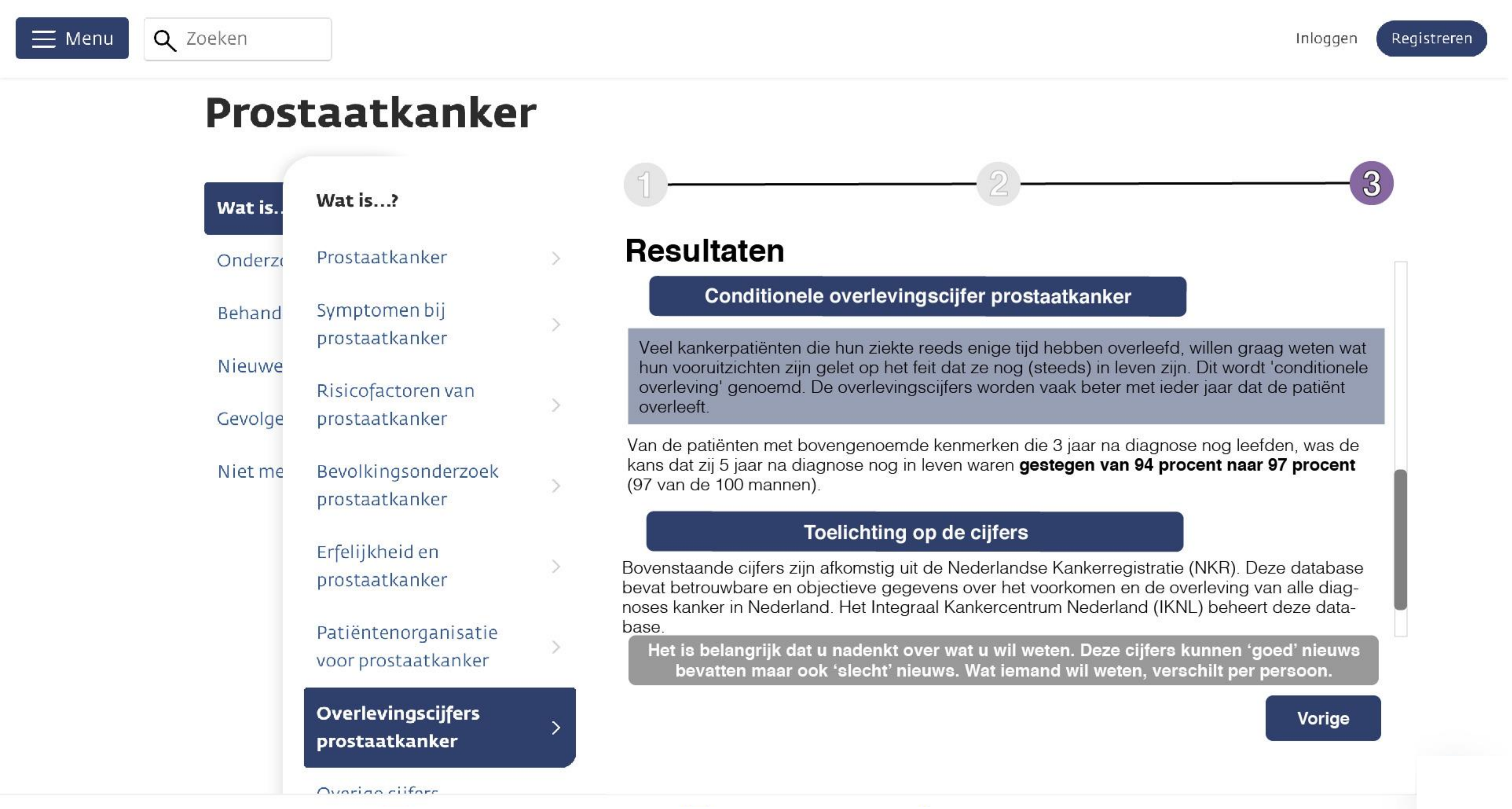

Translation:
Many patients with cancer who have survived for a couple of years want to know the chance that they will survive another x years (given that they are still alive). We call this conditional survival. In general, for cancer patients who have already survived a number of years, survival expectations at diagnosis are typically more favorable.

Menu Zoeken Inloggen Registreren

Prostaatkanker

Wat is...

Onderz

Behand

Nieuwe

Gevolge

Niet me

Wat is...?

- Prostaatkanker
- Symptomen bij prostaatkanker
- Risicofactoren van prostaatkanker
- Bevolkingsonderzoek prostaatkanker
- Erfelijkheid en prostaatkanker
- Patiëntenorganisatie voor prostaatkanker
- **Overlevingscijfers prostaatkanker**
- Overige cijfers

1 2 3

Resultaten

Conditionele overlevingscijfer prostaatkanker

?

Van de patiënten met bovengenoemde kenmerken die 3 jaar na diagnose nog leefden, was de kans dat zij 5 jaar na diagnose nog in leven waren **gestegen van 94 procent naar 97 procent** (97 van de 100 mannen).

Toelichting op de cijfers

Bovenstaande cijfers zijn afkomstig uit de Nederlandse Kankerregistratie (NKR). Deze database bevat betrouwbare en objectieve gegevens over het voorkomen en de overleving van alle diagnoses kanker in Nederland. Het Integraal Kankercentrum Nederland (IKNL) beheert deze database.

Bovenstaande cijfers zijn tot en met 2017. Meer recente cijfers zijn op dit moment niet beschikbaar. De veranderingen per jaar zijn meestal gering. Bovenstaande cijfers kunnen een indruk geven voor latere jaren.

Het is belangrijk dat u nadenkt over wat u wil weten. Deze cijfers kunnen 'goed' nieuws bevatten maar ook 'slecht' nieuws. Wat iemand wil weten, verschilt per persoon.

Vorige

Example 2: prostate cancer

- Survival statistic: Less favorable
- Version: Long and detailed tool version

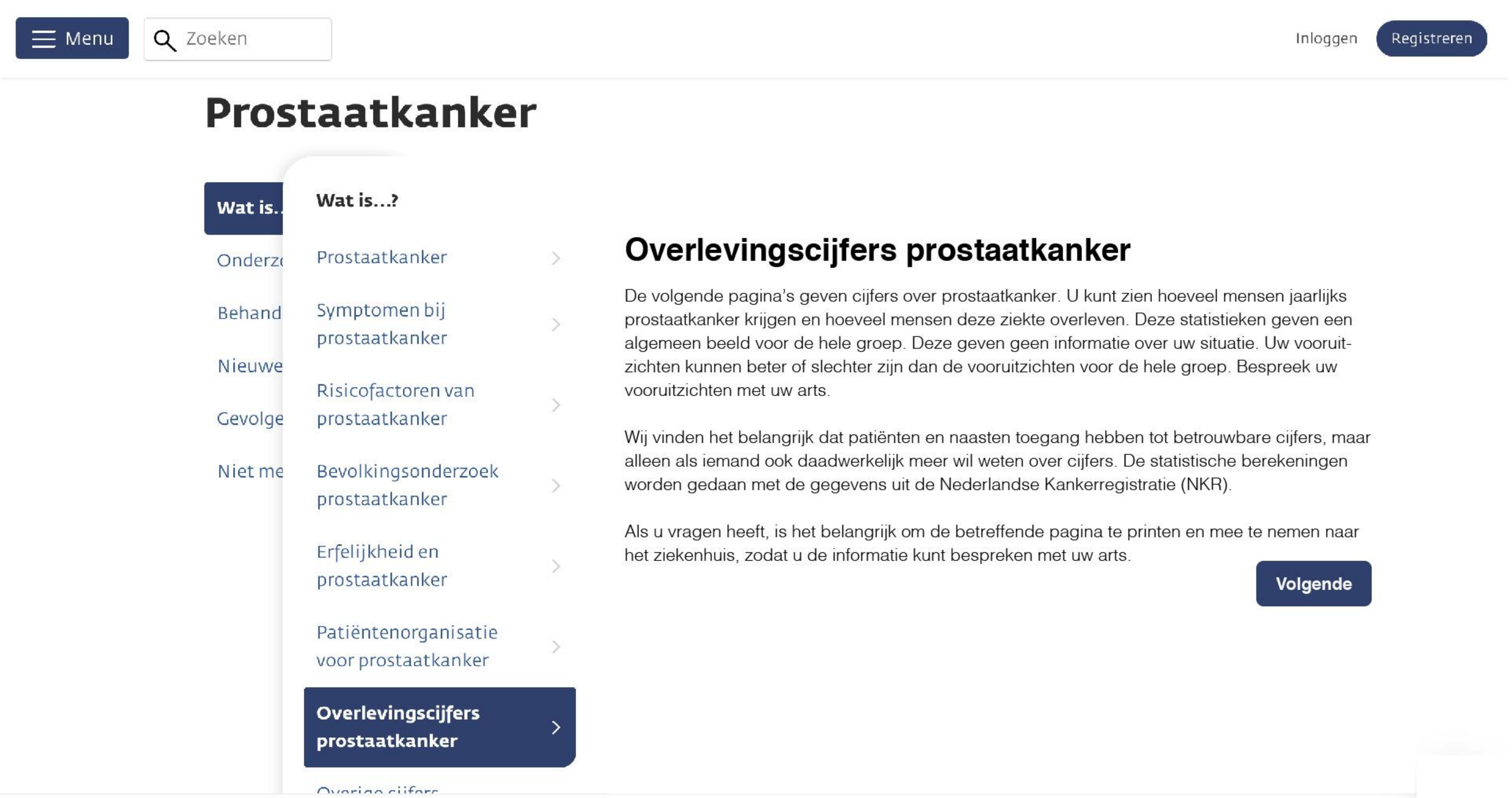

Translation:

Survival statistics prostate cancer

The next pages will show you statistics about prostate cancer. You will see how many men are diagnosed with prostate cancer, and how many of these men will survive prostate cancer. The statistics will give you a general idea for the entire group of patients. They do not say anything about your own situation. Your prognosis could be better or worse than the prognostic information that we show here. Please discuss your prognosis with your doctor.

We believe it is very important that patients and relatives have access to reliable statistics, but only if someone wants to know more about the statistics. The statistics are based on data from the Netherlands Cancer Registry (NCR). If you have any questions, you could always print the respective pages and take them with you to the next visit with your doctor, so you can discuss the results with him/her.

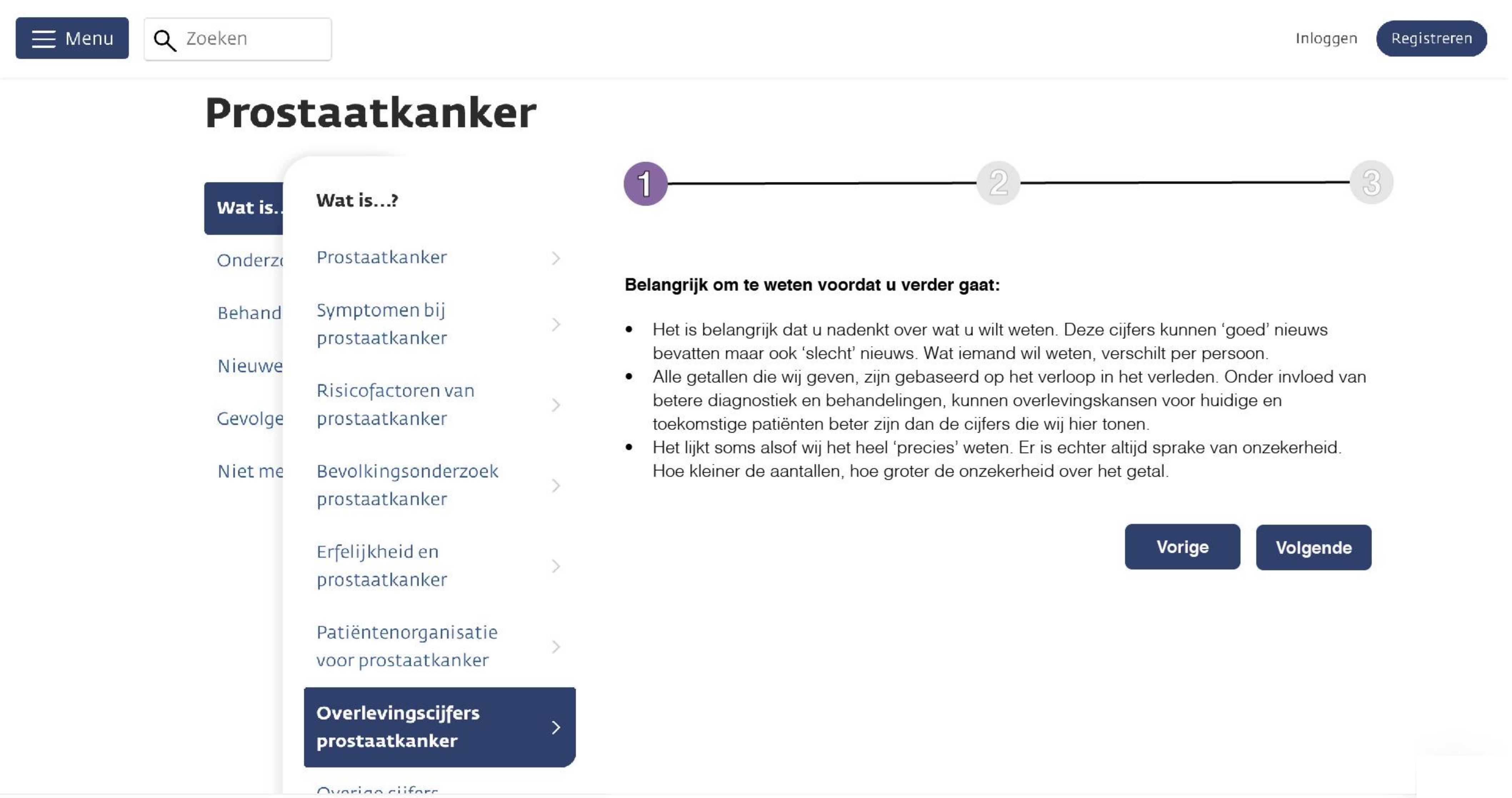

Translation:

Important to know before you continue:

- It is important that you think about what you want to know. These statistics may contain 'good' or 'bad' news. What someone wants to know, differs per person.
- All statistics that we provide are based on what happened in the past. Given the improvements in terms of diagnosis and treatments, the survival statistics for recently diagnosed and future patients may be better than those statistics that we show here.
- It may seem that we know everything without any uncertainty. However, there is always uncertainty around statistics.

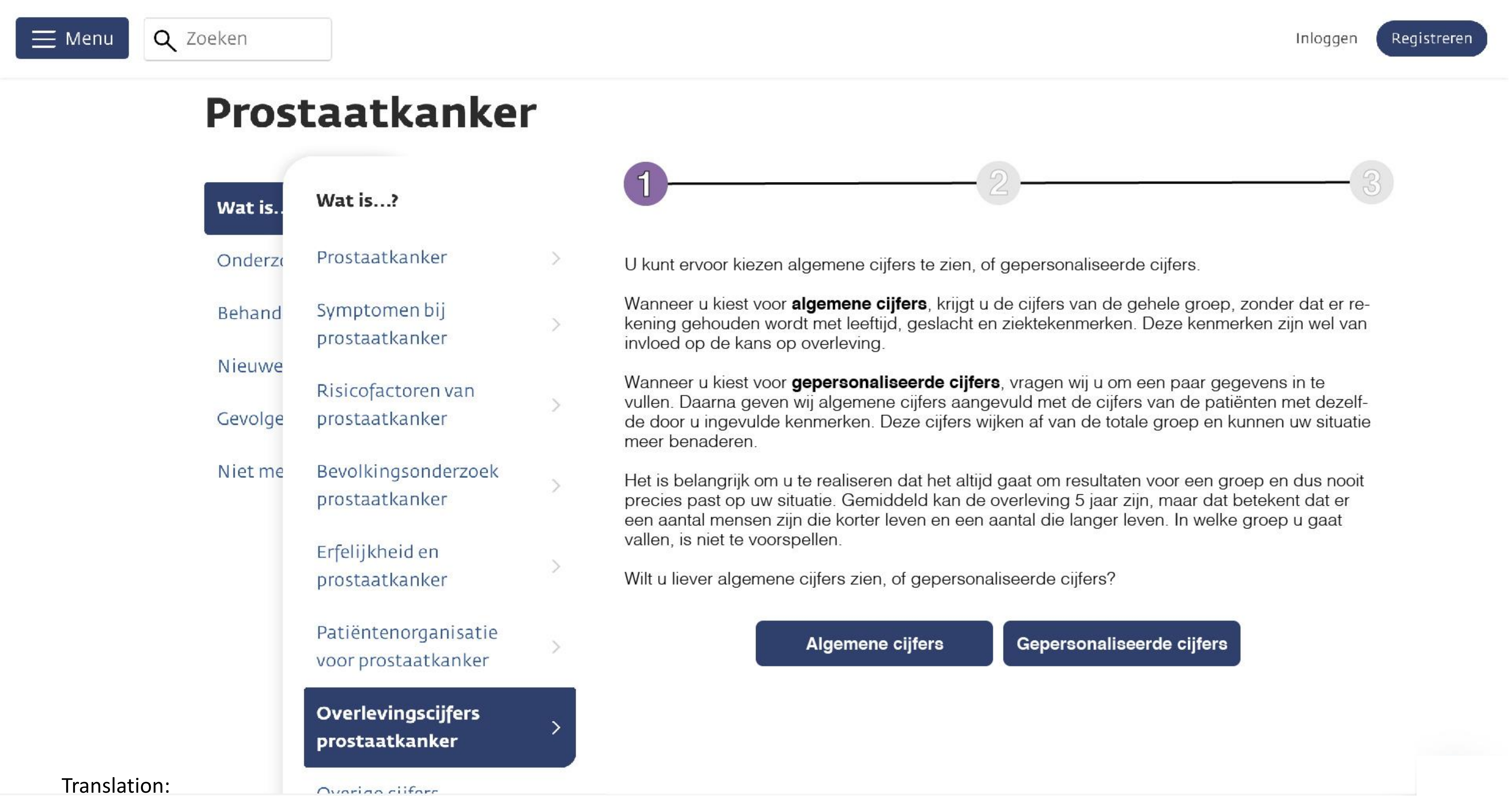

Translation:

You may choose to see generic statistics, of personalized statistics. If you click on **generic statistics**, you will see statistics for all prostate cancer patients, without taking into account factors such as age, gender, or disease-related factors. These factors do have an influence on survival. If you click on **personalized statistics**, you will be asked to enter some data, after which you will be presented with statistics that are based on patients like you (with the same personal and disease-related characteristics).

These personalized statistics deviate from the generic, group-based statistics, and may therefore better relate to your own situation. It is important to realize that we will always present statistics that are applicable to a group (and they will never really apply to your situation). For example, the average survival rate could be 5 years, but that means that some patients will have a shorter life, whereas other will life a bit longer. We cannot predict in which category or group you will belong to. Would you like to see generic statistics, or personalized statistics?

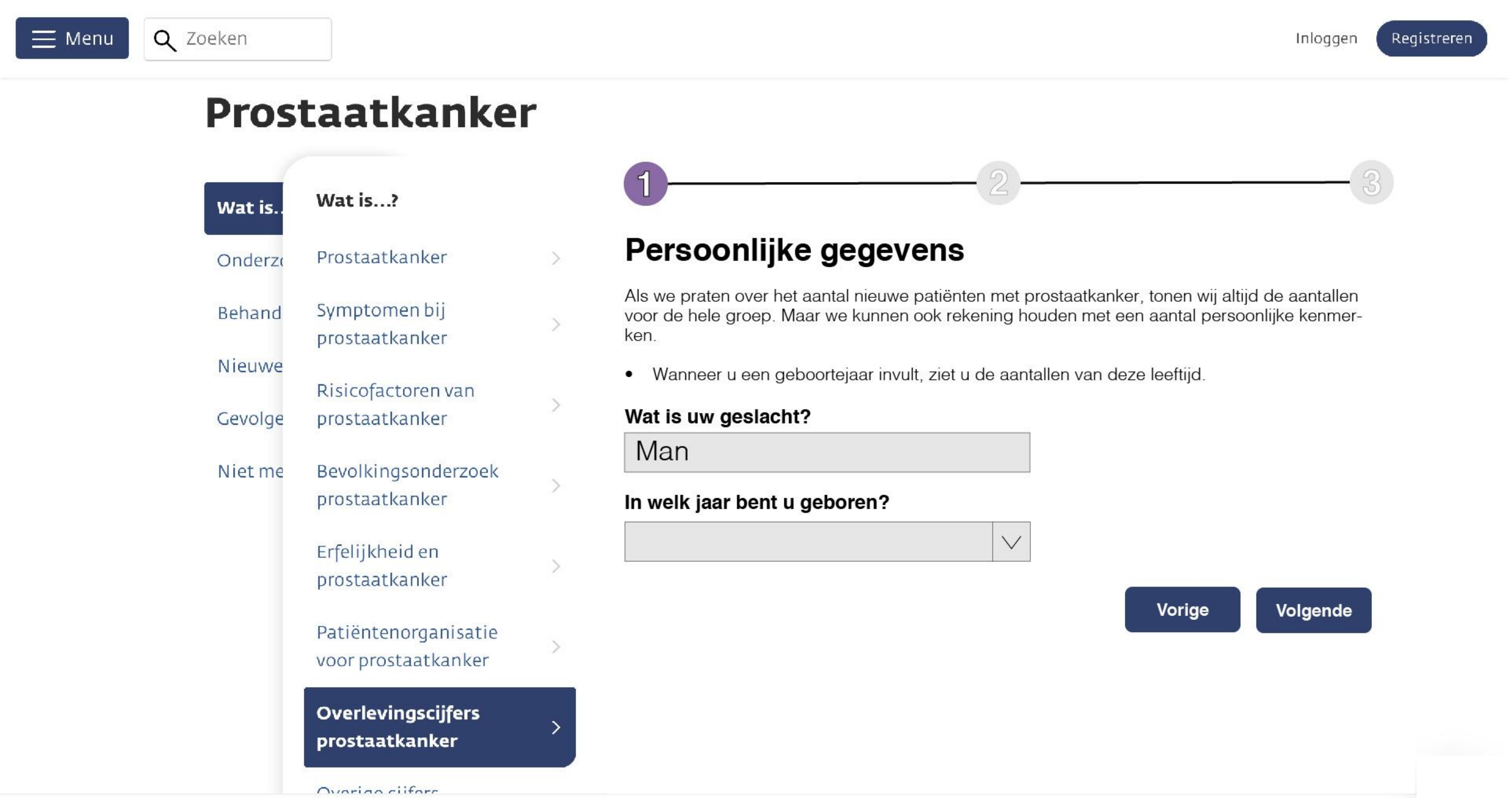

Translation:
Personal data
If we talk about the number of recently diagnosed patients with prostate cancer, we always show group-based statistics. However, we can also take a number of personal characteristics into account. In case you enter you year of birth, you will see the number of patients of that particular age.

- What is your gender? (men)
- What is your year of birth?

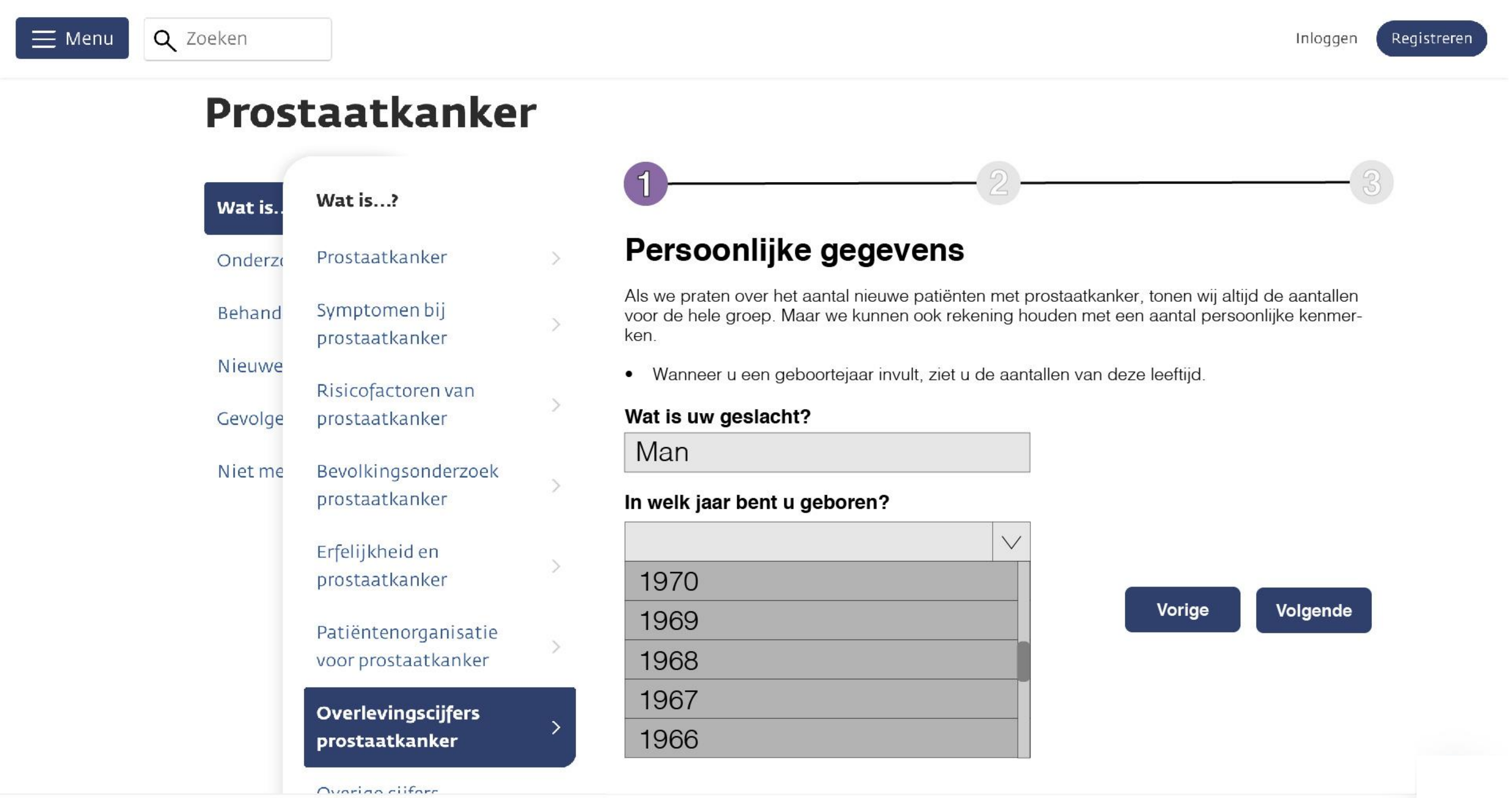

Translation:

Personal data

If we talk about the number of recently diagnosed patients with prostate cancer, we always show group-based statistics. However, we can also take a number of personal characteristics into account. In case you enter you year of birth, you will see the number of patients of that particular age.

- What is your gender? (men)
- What is your year of birth?

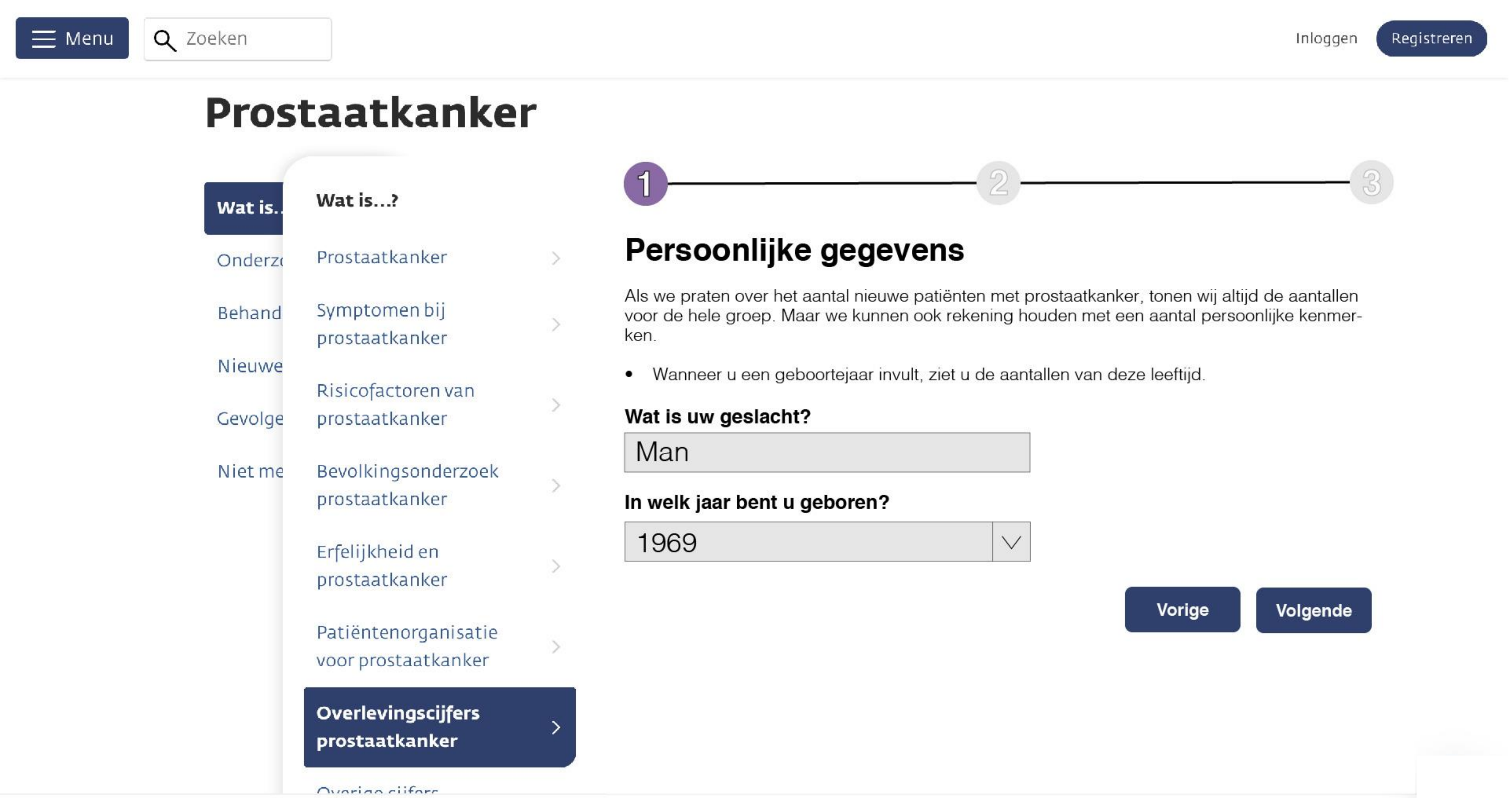

Translation:

Personal data

If we talk about the number of recently diagnosed patients with prostate cancer, we always show group-based statistics. However, we can also take a number of personal characteristics into account. In case you enter you year of birth, you will see the number of patients of that particular age.

- What is your gender? (men)
- What is your year of birth?

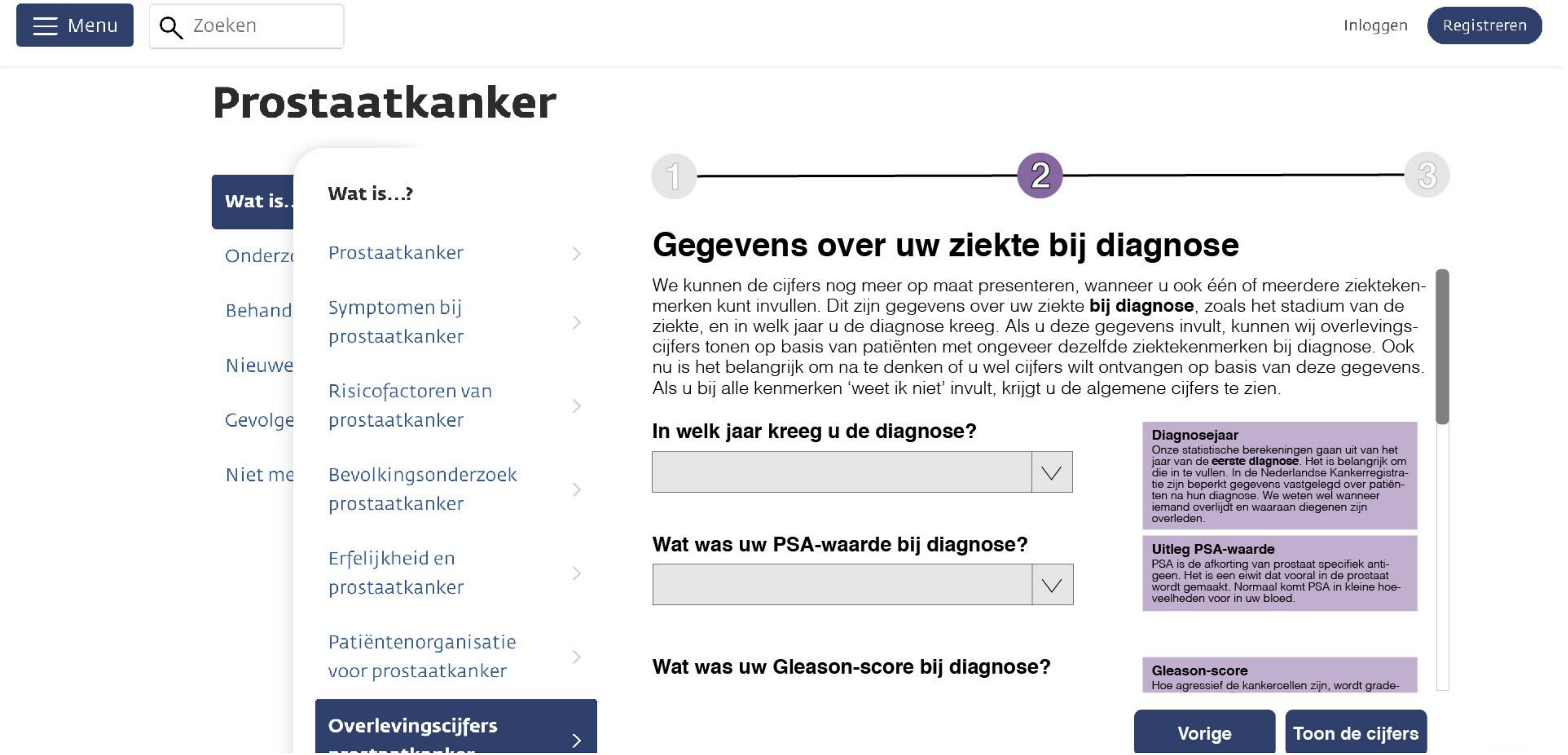

Translation:

Disease-related data

We can further personalize the statistics, for instance if you enter some disease-related characteristics at your diagnosis, such as the tumor stage, or the year of diagnosis. If you enter these data, we can show statistics based on patients like you with similar characteristics at their diagnosis. Again, please think about whether you really want to see personalized statistics. If you enter "I don't know" for each characteristic, you will only see the generic statistics.

- Year of diagnosis? (Our calculations are based on the first cancer diagnosis. It is important to fill out this one, because the Netherlands Cancer Registry contains limited data of patients after their diagnosis. However, the NCR does know when a patient died (and its cause)).
- PSA-value at diagnosis? (PSA stands for Prostate-specific antigen. This is a protein produced by normal, as well as malignant, cells of the prostate gland).

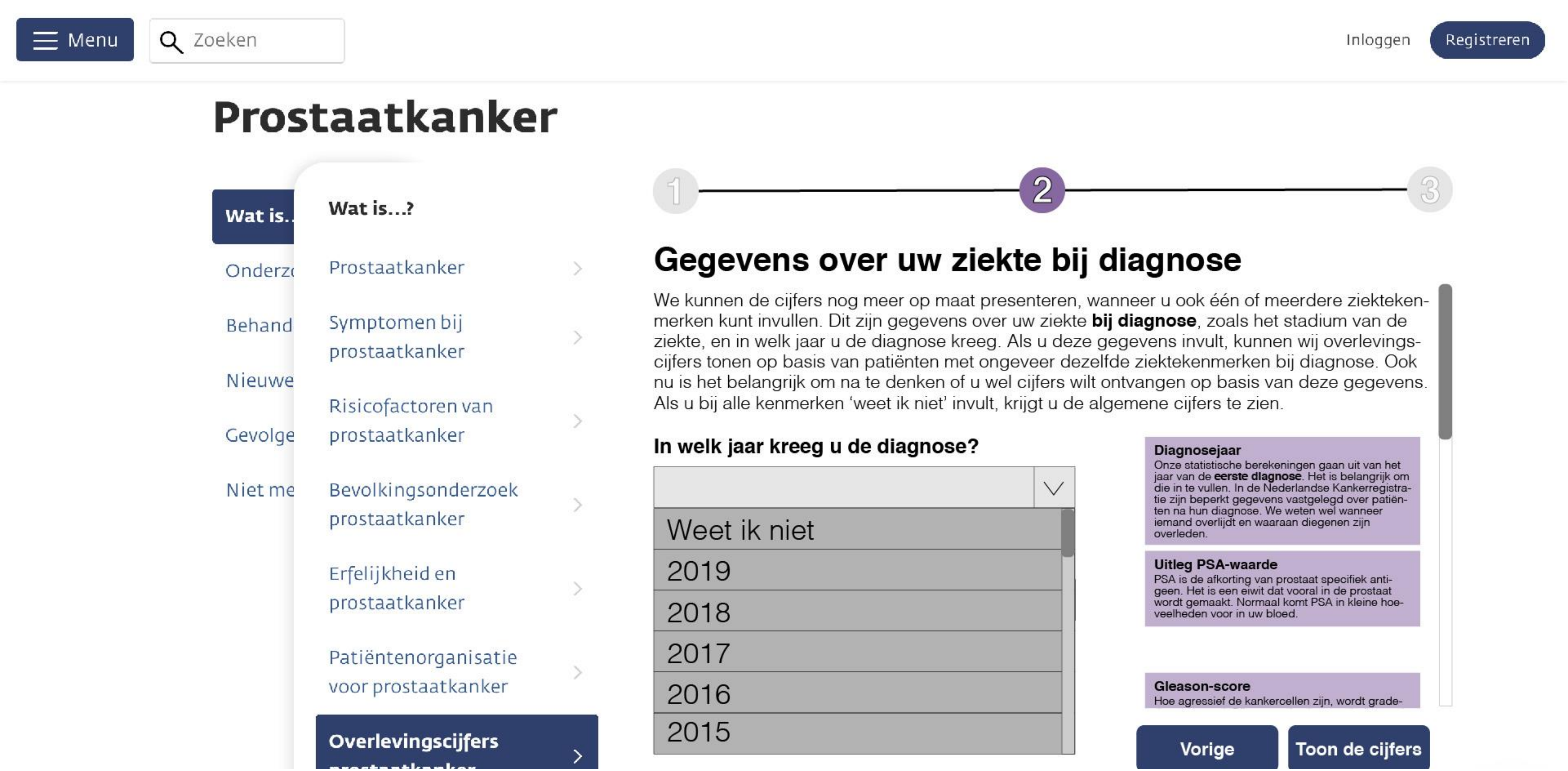

Translation:

Disease-related data

We can further personalize the statistics, for instance if you enter some disease-related characteristics at your diagnosis, such as the tumor stage, or the year of diagnosis. If you enter these data, we can show statistics based on patients like you with similar characteristics at their diagnosis. Again, please think about whether you really want to see personalized statistics. If you enter "I don't know" for each characteristic, you will only see the generic statistics.

- Year of diagnosis? (Our calculations are based on the first cancer diagnosis. It is important to fill out this one, because the Netherlands Cancer Registry contains limited data of patients after their diagnosis. However, the NCR does know when a patient died (and its cause)).
- PSA-value at diagnosis? (PSA stands for Prostate-specific antigen. This is a protein produced by normal, as well as malignant, cells of the prostate gland).

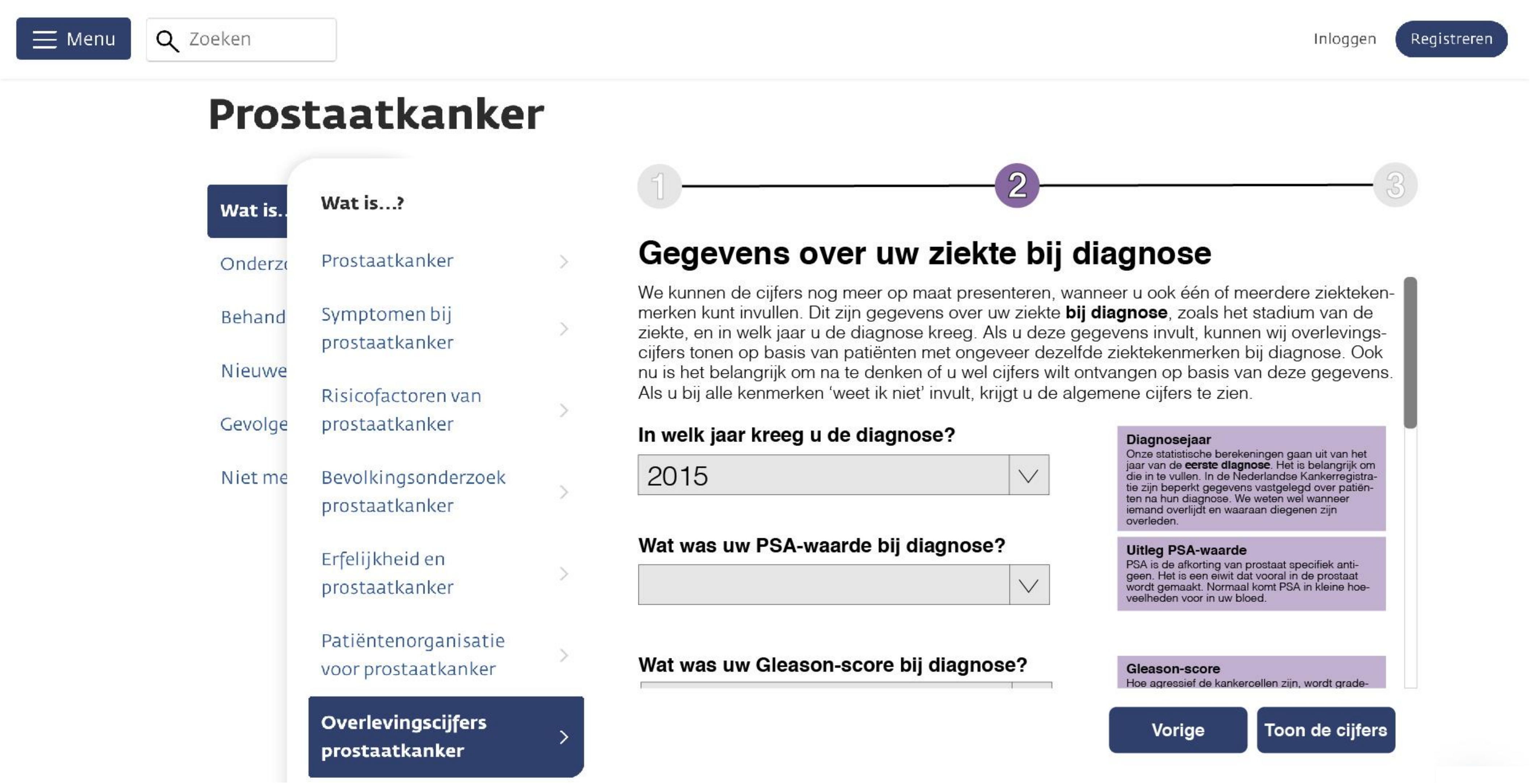

Translation:

Disease-related data

We can further personalize the statistics, for instance if you enter some disease-related characteristics at your diagnosis, such as the tumor stage, or the year of diagnosis. If you enter these data, we can show statistics based on patients like you with similar characteristics at their diagnosis. Again, please think about whether you really want to see personalized statistics. If you enter "I don't know" for each characteristic, you will only see the generic statistics.

- Year of diagnosis? (2015)
- PSA-value at diagnosis?

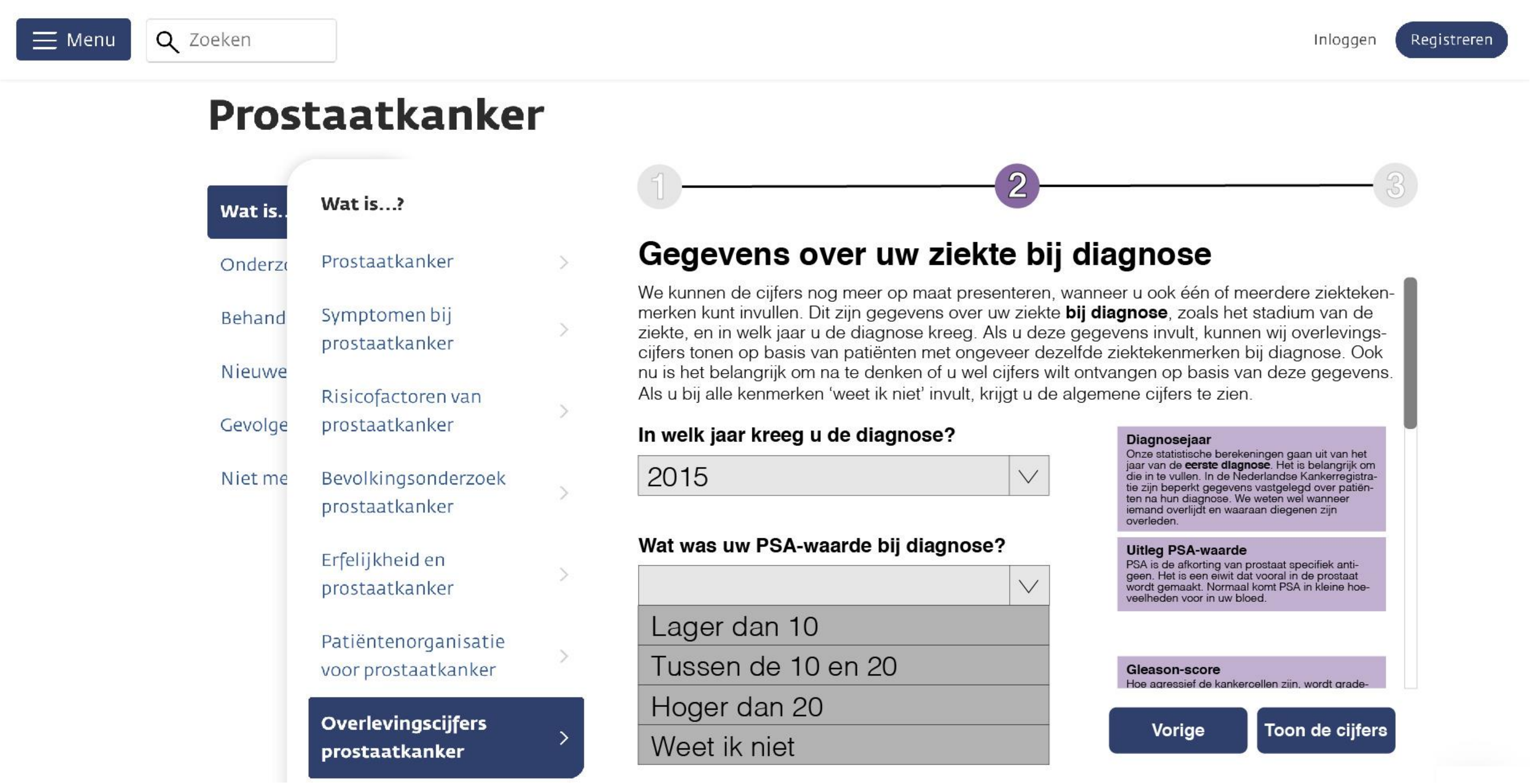

Translation:

Disease-related data

We can further personalize the statistics, for instance if you enter some disease-related characteristics at your diagnosis, such as the tumor stage, or the year of diagnosis. If you enter these data, we can show statistics based on patients like you with similar characteristics at their diagnosis. Again, please think about whether you really want to see personalized statistics. If you enter "I don't know" for each characteristic, you will only see the generic statistics.

- Year of diagnosis? (2015)
- PSA-value at diagnosis? (Lower than 10, Between 10 and 20, Higher than 20, I don't know)

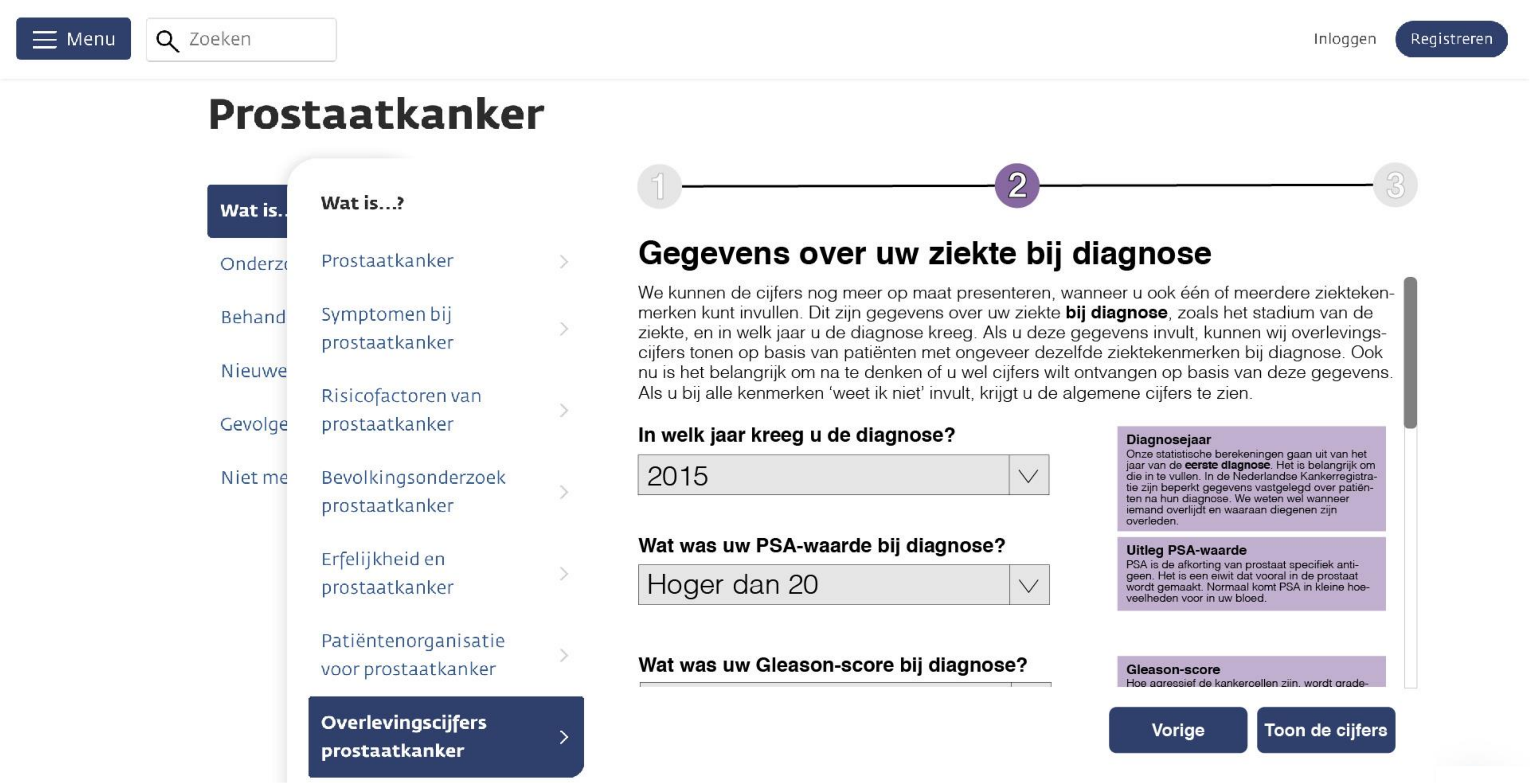

Translation:

Disease-related data

We can further personalize the statistics, for instance if you enter some disease-related characteristics at your diagnosis, such as the tumor stage, or the year of diagnosis. If you enter these data, we can show statistics based on patients like you with similar characteristics at their diagnosis. Again, please think about whether you really want to see personalized statistics. If you enter "I don't know" for each characteristic, you will only see the generic statistics.

- Year of diagnosis? (2015)
- PSA-value at diagnosis? (Higher than 20)

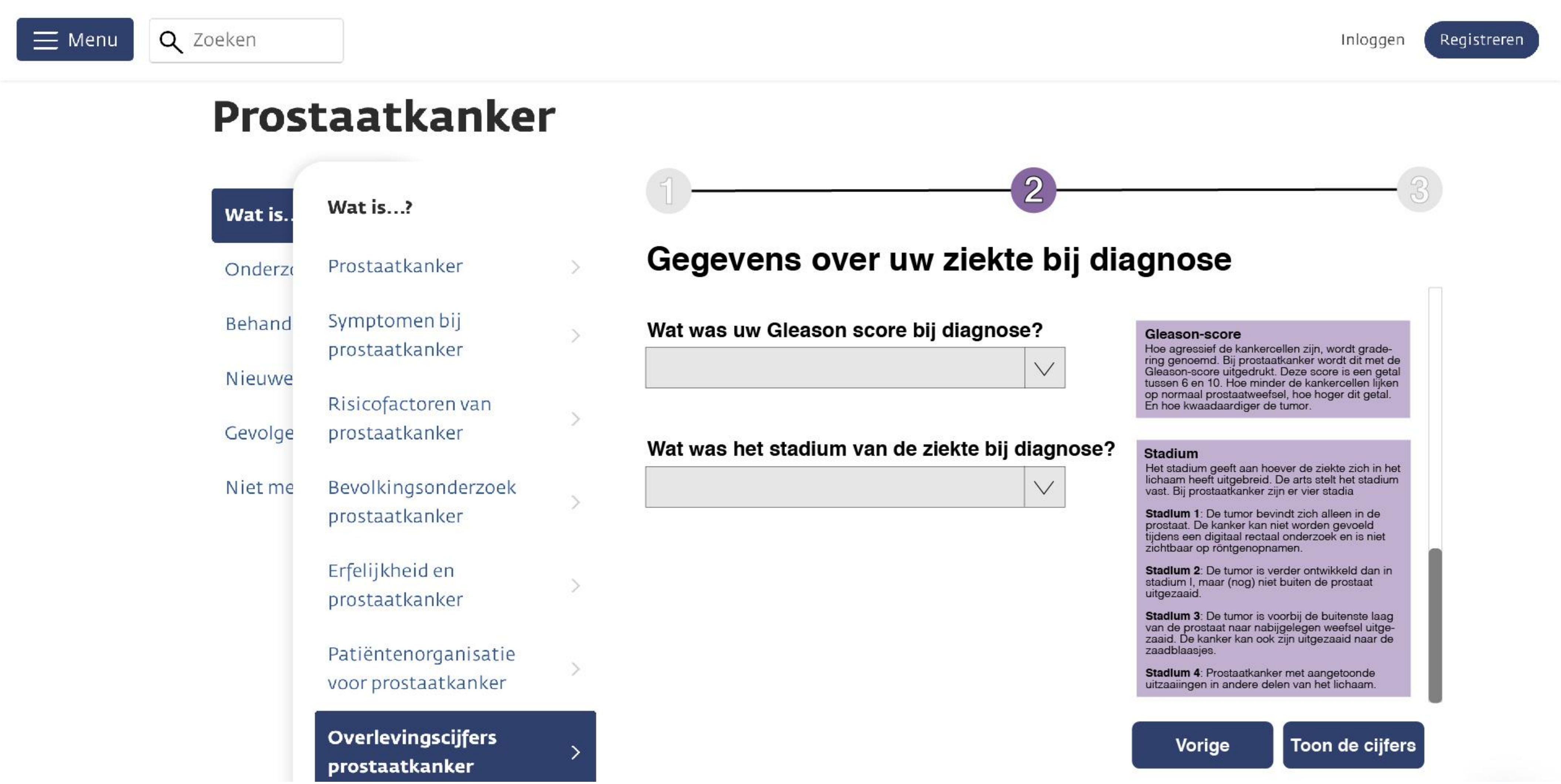

Translation:

Disease-related data

- Gleason-score at diagnosis? (The tumor grade says something about the aggressiveness of the tumor. For prostate cancer, this is expressed by means of the Gleason-score. This score is a number between 6 and 10. Cancer cells that deviate from healthy cells receive a high score. The higher this score, the more aggressive the tumor is).
- Tumor stage at diagnosis? The tumor stage says something about how far the cancer has progressed. The doctor will determine the tumor stage. For prostate cancer, there are four stages. **Stage 1:** the tumor is still within the prostate. The doctor cannot feel the tumor during a rectal exam, or the tumor is not visible on a scan. **Stage 2:** the tumor remains confined to the prostate gland, but it has not spread outside the prostate. **Stage 3:** the tumor is locally advanced. The tumor has progressed and is more likely to grow and spread. **Stage 4:** the tumor has spread to lymph nodes or to other parts of the body.

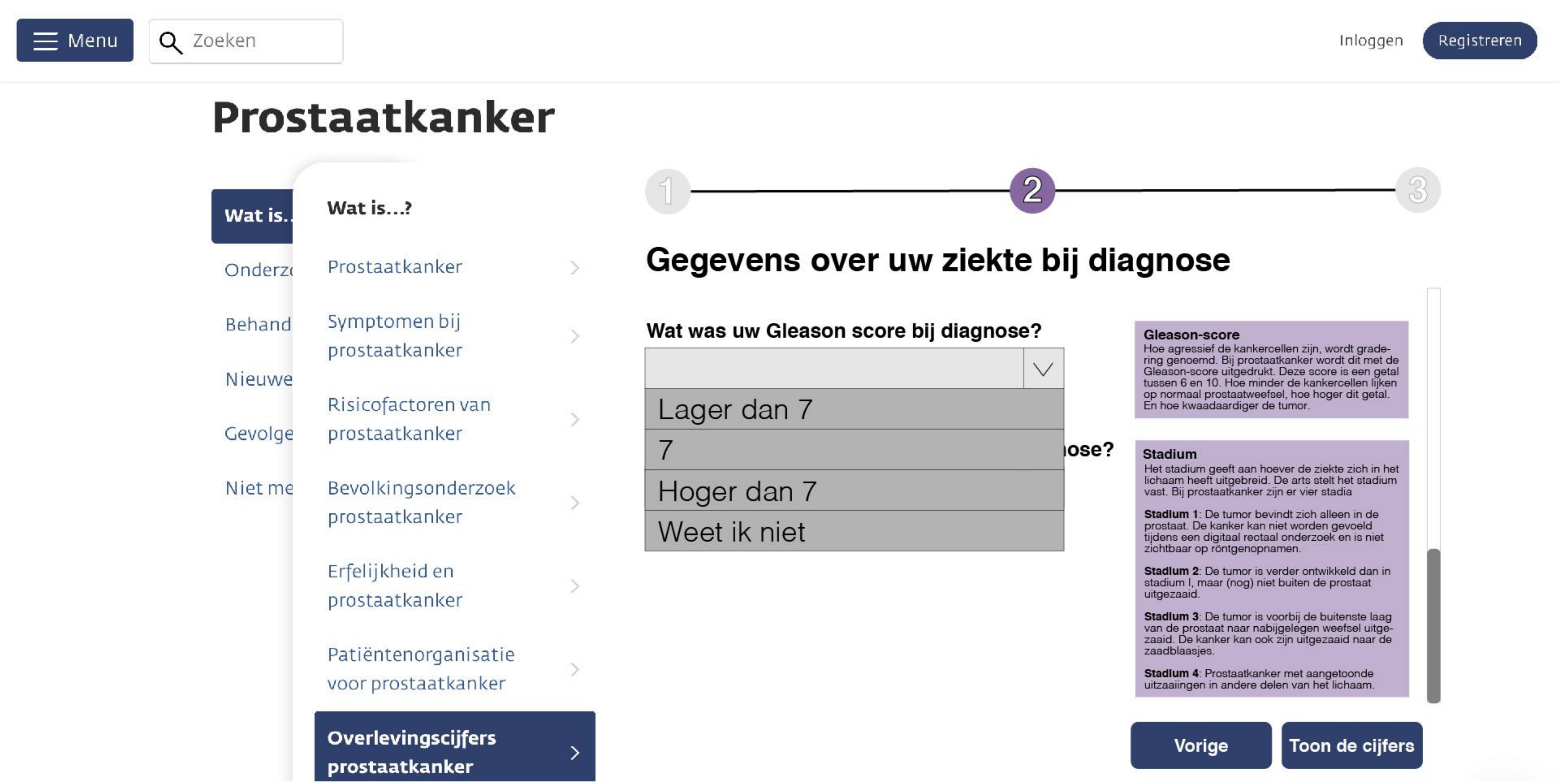

Translation:

Disease-related data

- Gleason-score at diagnosis? (Lower than 7, 7, Higher than 7, I don’t know)
- Tumor stage at diagnosis?

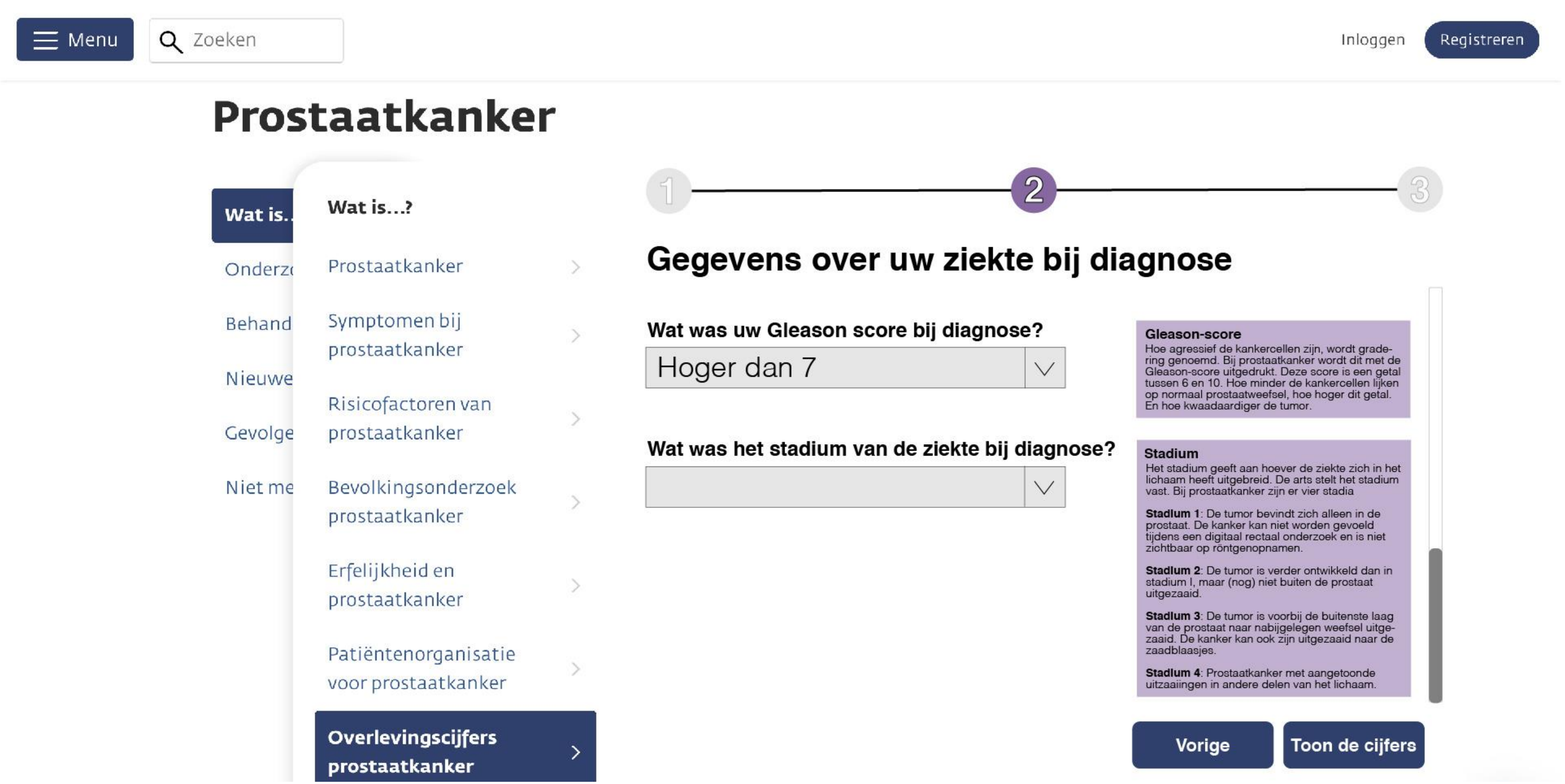

Translation:

Disease-related data

- Gleason-score at diagnosis? (Higher than 7)
- Tumor stage at diagnosis?

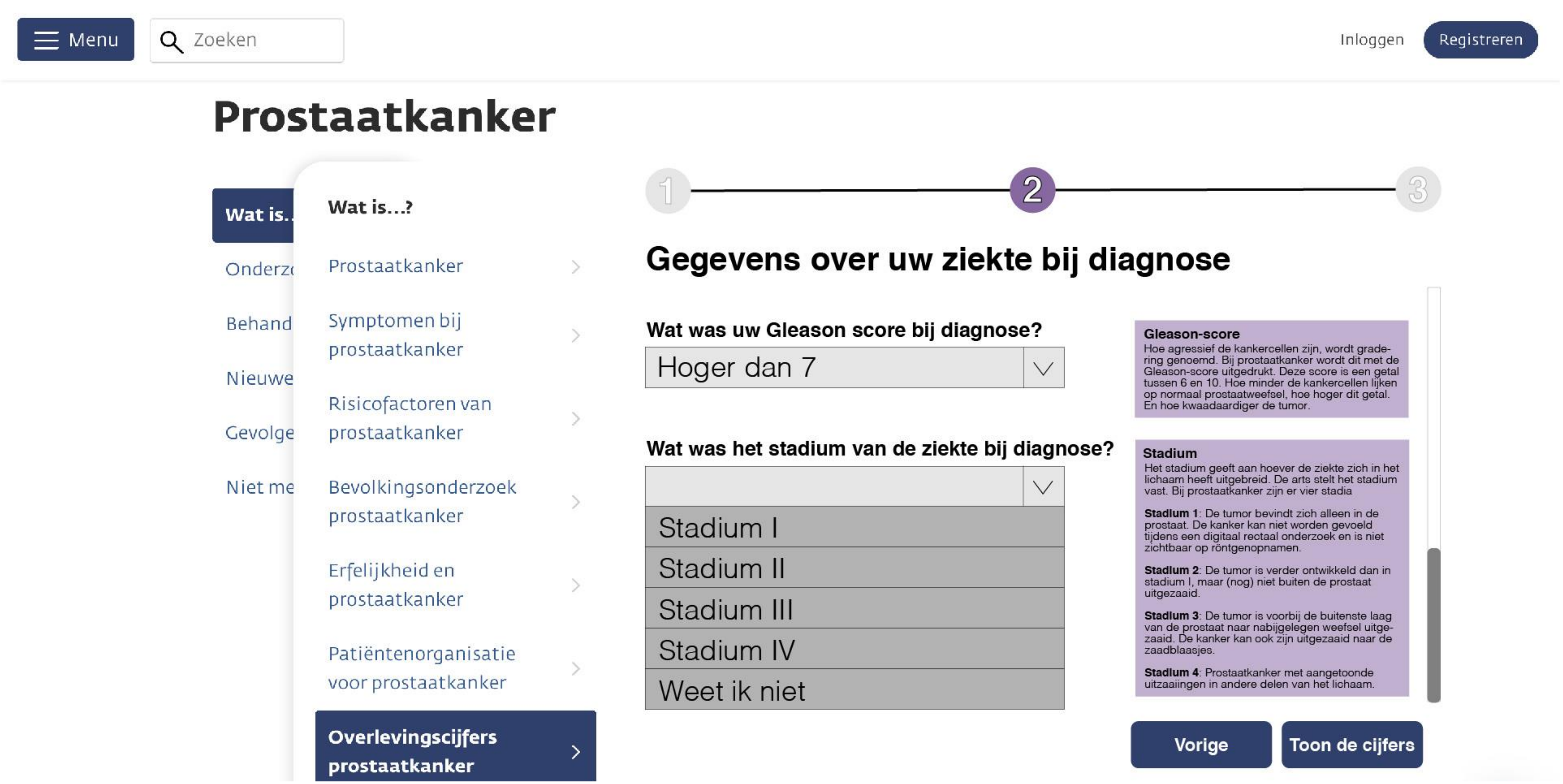

Translation:

Disease-related data

- Gleason-score at diagnosis? (Higher than 7)
- Tumor stage at diagnosis? (Stage I, stage II, stage II, Stage IV)

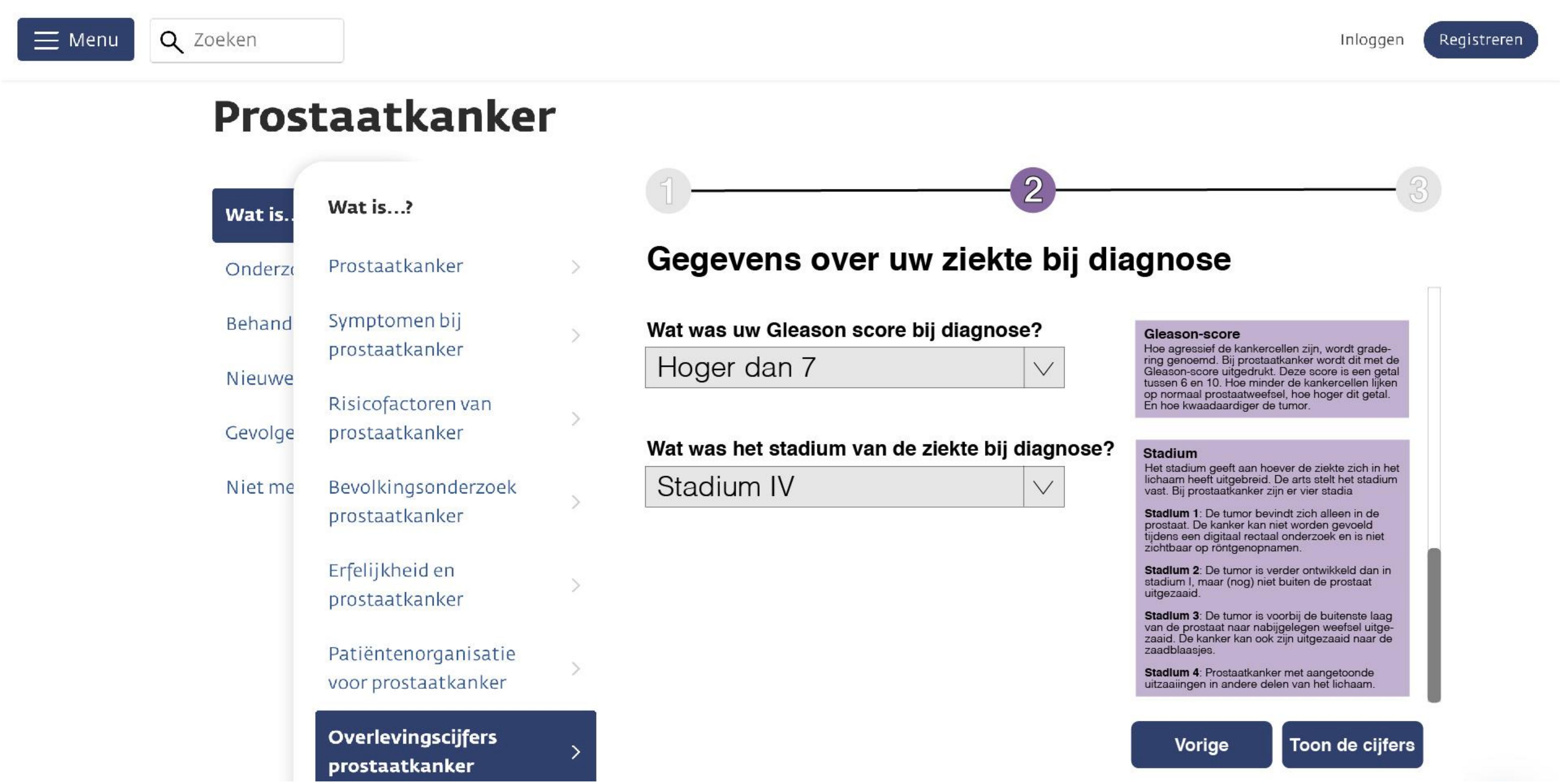

Translation:
Disease-related data
- Gleason-score at diagnosis? (Higher than 7)
- Tumor stage at diagnosis? (stage IV)

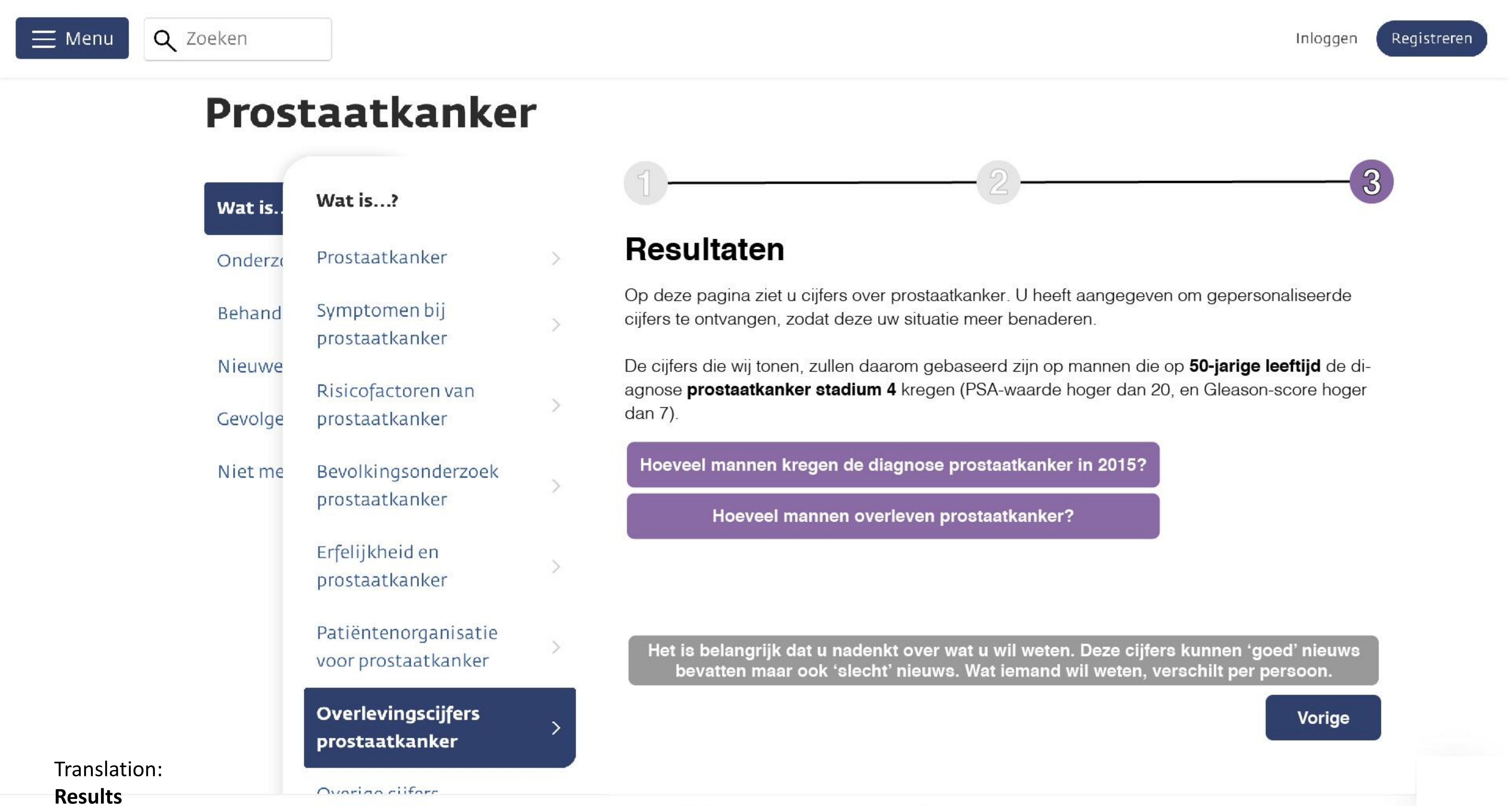

Translation:

Results

On this page, you will see some prostate cancer statistics. You have indicated to receive personalized statistics that are closer to your personal situation. For this reason, the statistics will be based on men **aged 50**, diagnosed with **prostate cancer stage 4** (PSA-value higher than 20 and Gleason score higher than 7).

How many men were diagnosed with prostate cancer in 2015?

How many men survive prostate cancer?

It is important that you think about what you would like to know. These statistics can contain 'good' but also 'bad' news. What someone wants to know differs per person.

Menu Zoeken Inloggen Registreren

Prostaatkanker

Wat is...

Onderz

Behand

Nieuwe

Gevolge

Niet me

Wat is...?

- Prostaatkanker
- Symptomen bij prostaatkanker
- Risicofactoren van prostaatkanker
- Bevolkingsonderzoek prostaatkanker
- Erfelijkheid en prostaatkanker
- Patiëntenorganisatie voor prostaatkanker
- **Overlevingscijfers prostaatkanker**
- Overige cijfers

1 — 2 — 3

Resultaten

Op deze pagina ziet u cijfers over prostaatkanker. U heeft aangegeven om gepersonaliseerde cijfers te ontvangen, zodat deze uw situatie meer benaderen.

De cijfers die wij tonen, zullen daarom gebaseerd zijn op mannen die op **50-jarige leeftijd** de diagnose **prostaatkanker stadium 4** kregen (PSA-waarde hoger dan 20, en Gleason-score hoger dan 7).

Hoeveel mannen kregen de diagnose prostaatkanker in 2015?

In **2015** kregen in totaal 10.629 mannen de diagnose prostaatkanker. Als we rekening houden met de bovengenoemde kenmerken, kregen in 2015 70 mannen de diagnose prostaatkanker stadium 4 (PSA-waarde hoger dan 20, en Gleason-score hoger dan 7) op 50-jarige leeftijd.

Hoeveel mannen overleven prostaatkanker?

Het is belangrijk dat u nadenkt over wat u wil weten. Deze cijfers kunnen 'goed' nieuws bevatten maar ook 'slecht' nieuws. Wat iemand wil weten, verschilt per persoon.

Vorige

Translation:
In **2015**, in total 10.629 men were diagnosed with prostate cancer. If we take the above mentioned characteristics into account, 70 men like you (aged 50, PSA-value higher than 20, and Gleason score higher than 7) were diagnosed with prostate cancer stage IV in 2015.

Menu Zoeken Inloggen Registreren

Prostaatkanker

Wat is...

Onderz[illegible]

Behand[illegible]

Nieuwe[illegible]

Gevolge[illegible]

Niet me[illegible]

Wat is...?

- Prostaatkanker
- Symptomen bij prostaatkanker
- Risicofactoren van prostaatkanker
- Bevolkingsonderzoek prostaatkanker
- Erfelijkheid en prostaatkanker
- Patiëntenorganisatie voor prostaatkanker
- **Overlevingscijfers prostaatkanker**

1 2 3

Resultaten

Hoeveel mannen overleven prostaatkanker?

Met **overleving** bedoelen we hoeveel mensen met een bepaalde vorm van kanker na een bepaalde periode nog in leven zijn. In dit geval laten we zien hoeveel mannen met de diagnose prostaatkanker 5 jaar na diagnose nog in leven zijn.

Om betrouwbare getallen te geven, moet er gerekend worden met grote aantallen patiënten die in het verleden prostaatkanker hebben gehad. De cijfers die wij hier tonen zullen gebaseerd zijn op patiënten met de bovengenoemde kenmerken die tussen 2012 en 2017 de diagnose prostaatkanker kregen.

We laten eerst de kans op overleving zien van alle patiënten met prostaatkanker. 5 jaar na diagnose was ongeveer **89 procent** van de prostaatkanker patiënten nog in leven (89 van de 100 mannen).

We kunnen ook de kans op overleving laten zien van prostaatkanker patiënten die bij diagnose de bovengenoemde kenmerken hadden. 5 jaar na diagnose was ongeveer **47 procent** van deze patiënten nog in leven (47 van de 100 mannen).

Het is belangrijk dat u nadenkt over wat u wil weten. Deze cijfers kunnen 'goed' nieuws bevatten maar ook 'slecht' nieuws. Wat iemand wil weten, verschilt per persoon.

Vorige

Translation:
With **survival** we mean the percentage of people with cancer still alive after a particular amount of time. Here we will show you how many men are still alive 5 years after diagnosis.
In order to provide you with reliable statistics we need to perform statistics on a large group of patients that have been diagnosed with prostate cancer in the past. The statistics that we show here are performed on data from patients diagnosed between 2012 and 2017.
We will first show you the chance of survival for all men with prostate cancer. 5 years after diagnosis, about **89 percent** of these patients with prostate cancer are alive (89 out of 100 men). We can also show you the chance of survival for men with prostate cancer diagnosed with the above mentioned characteristics. 5 years after diagnosis, about **47 percent** of these patients with prostate cancer are alive (47 out of 100 men).

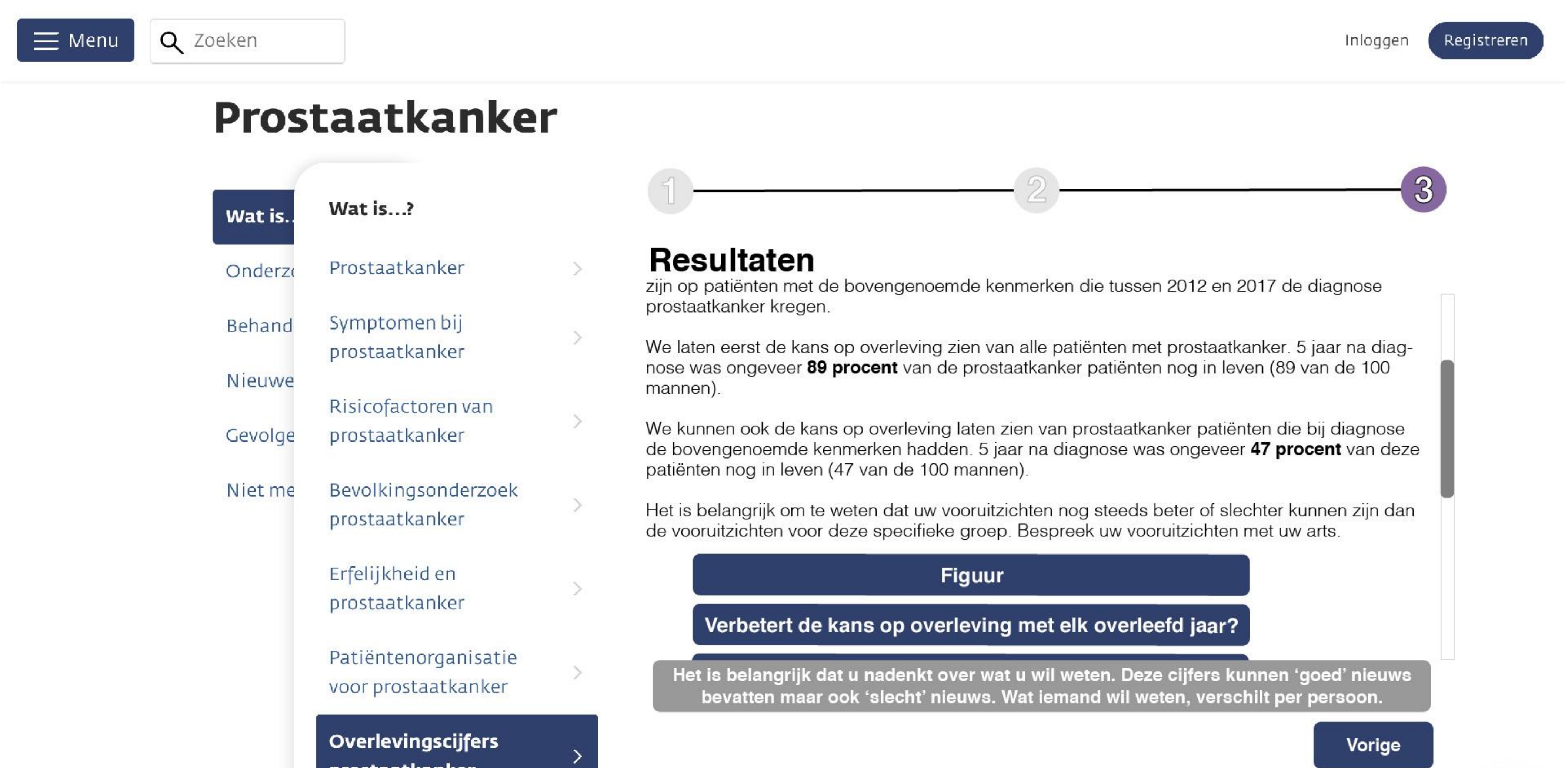

Translation:
In order to provide you with reliable statistics we need to perform statistics on a large group of patients that have been diagnosed with prostate cancer in the past. The statistics that we show here are performed on data from patients diagnosed between 2012 and 2017.
We will first show you the chance of survival for all men with prostate cancer. 5 years after diagnosis, about **89 percent** of these patients with prostate cancer are alive (89 out of 100 men). We can also show you the chance of survival for men with prostate cancer diagnosed with the above mentioned characteristics. 5 years after diagnosis, about **47 percent** of these patients with prostate cancer are alive (47 out of 100 men).
It is important to know that your prognosis can still be better or worse than the prognosis of this specific group of patients. Discuss your prognosis with your doctor.
Figure
What is the survival statistic after having survived a period of time?

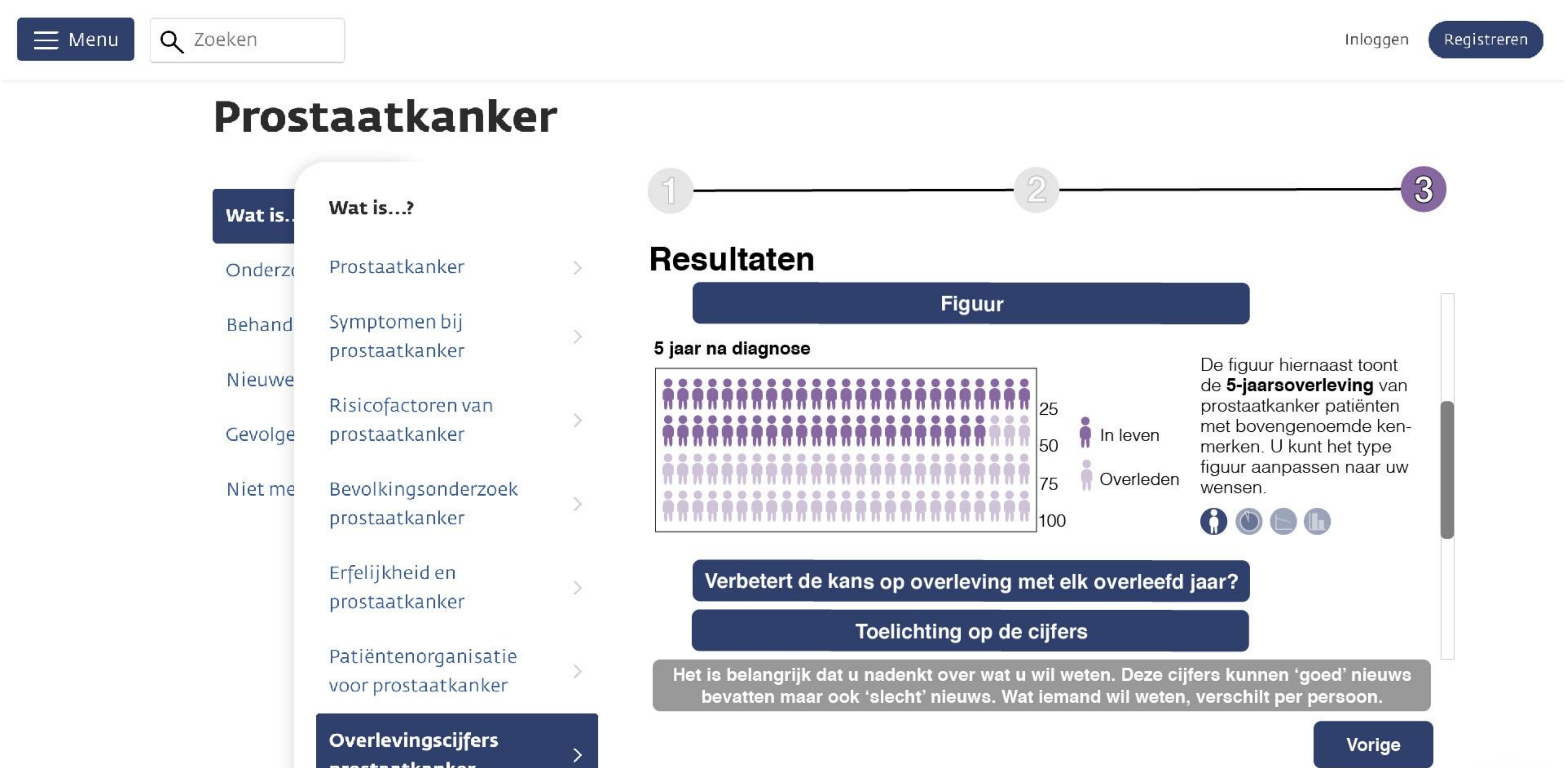

Translation:
This figure shows the **5 year survival statistic** for prostate cancer with the above mentioned characteristics. You can change the type of figure.
In leven = are alive
Overleden = die

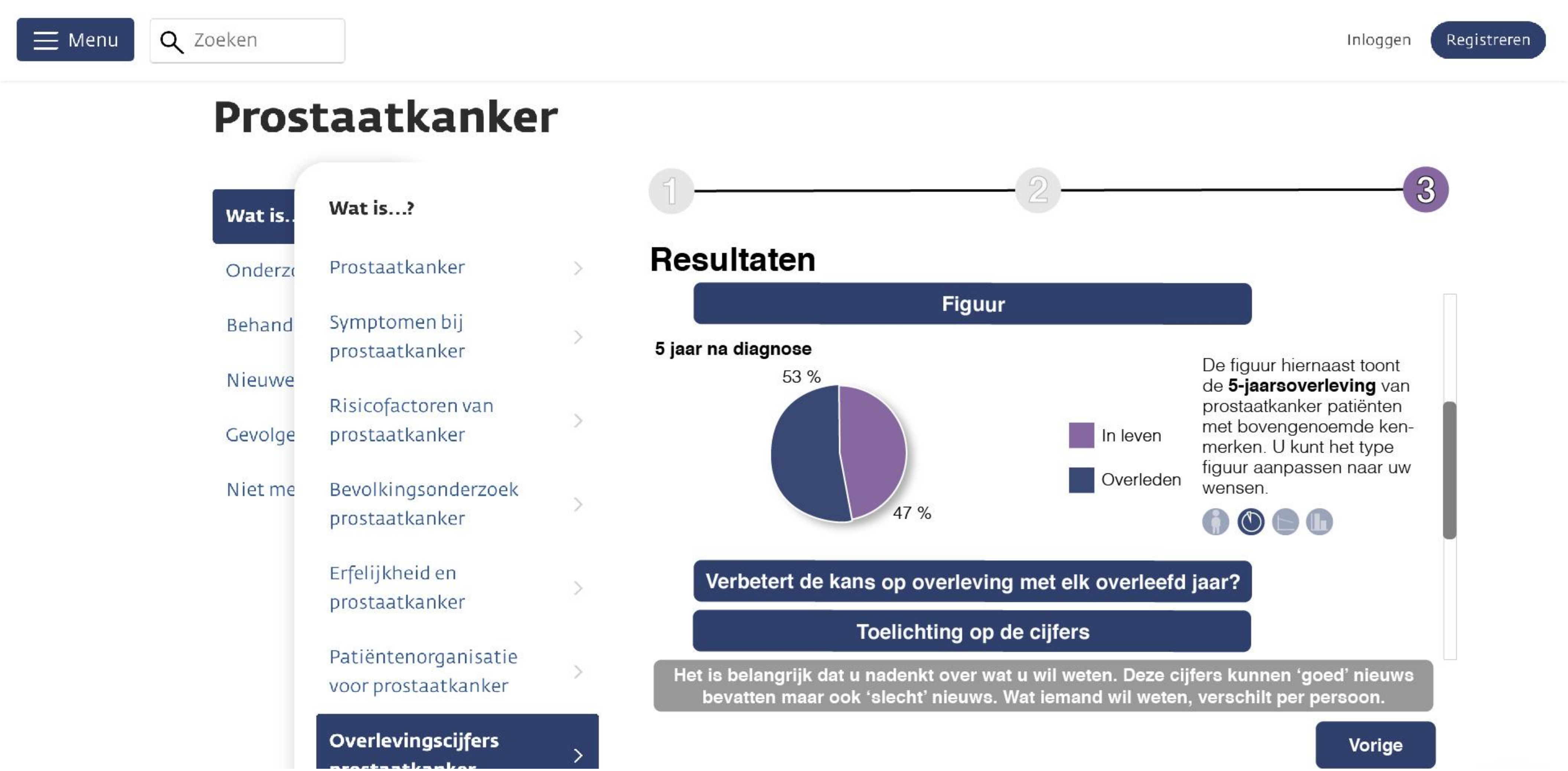

Translation:
This figure shows the **5 year survival statistic** for prostate cancer with the above mentioned characteristics. You can change the type of figure.
In leven = are alive
Overleden = die

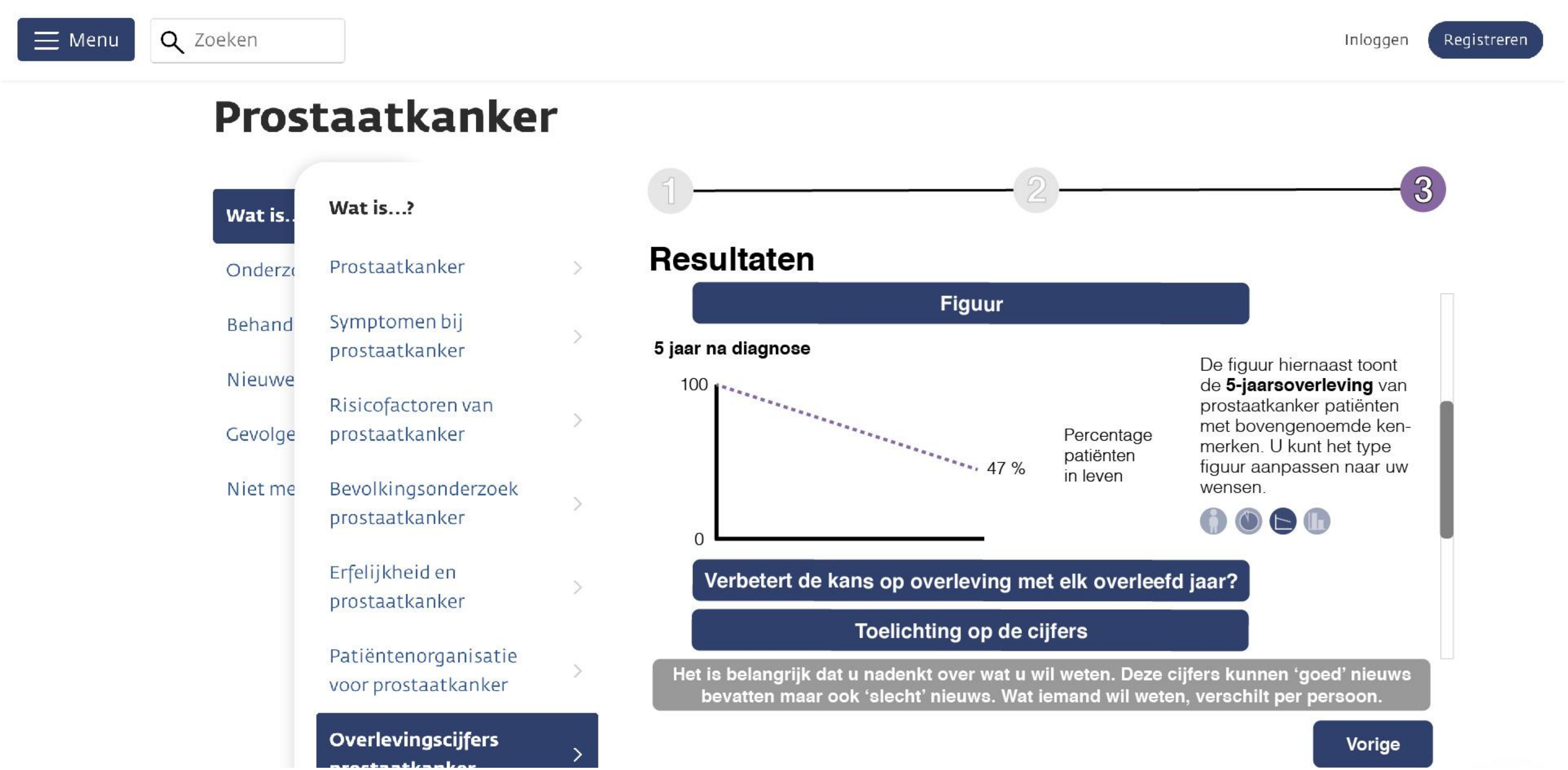

Translation:
This figure shows the **5 year survival statistic** for prostate cancer with the above mentioned characteristics. You can change the type of figure.
In leven = are alive
Overleden = die

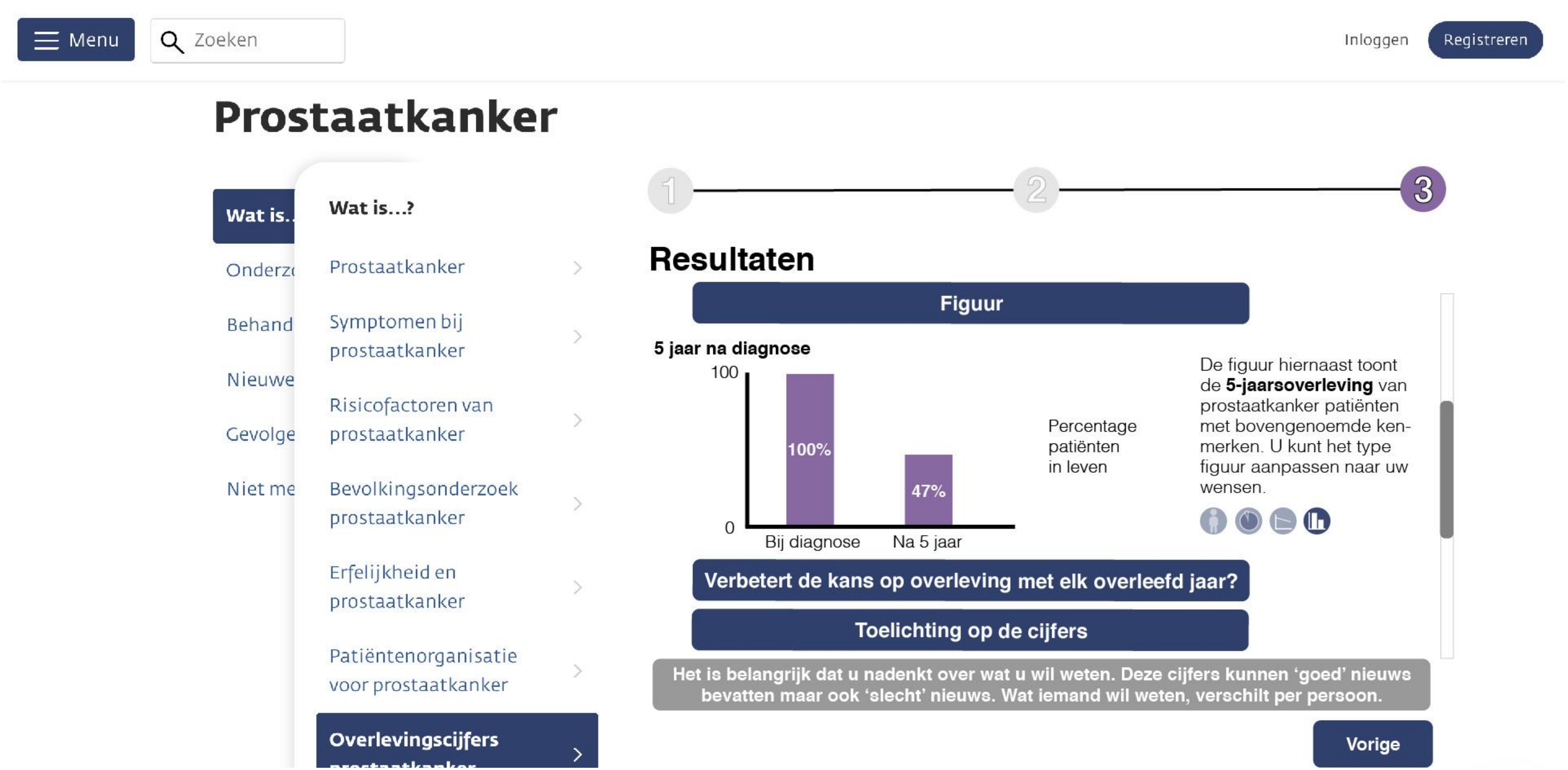

Translation:
This figure shows the **5 year survival statistic** for prostate cancer with the above mentioned characteristics. You can change the type of figure.
In leven = are alive
Overleden = die

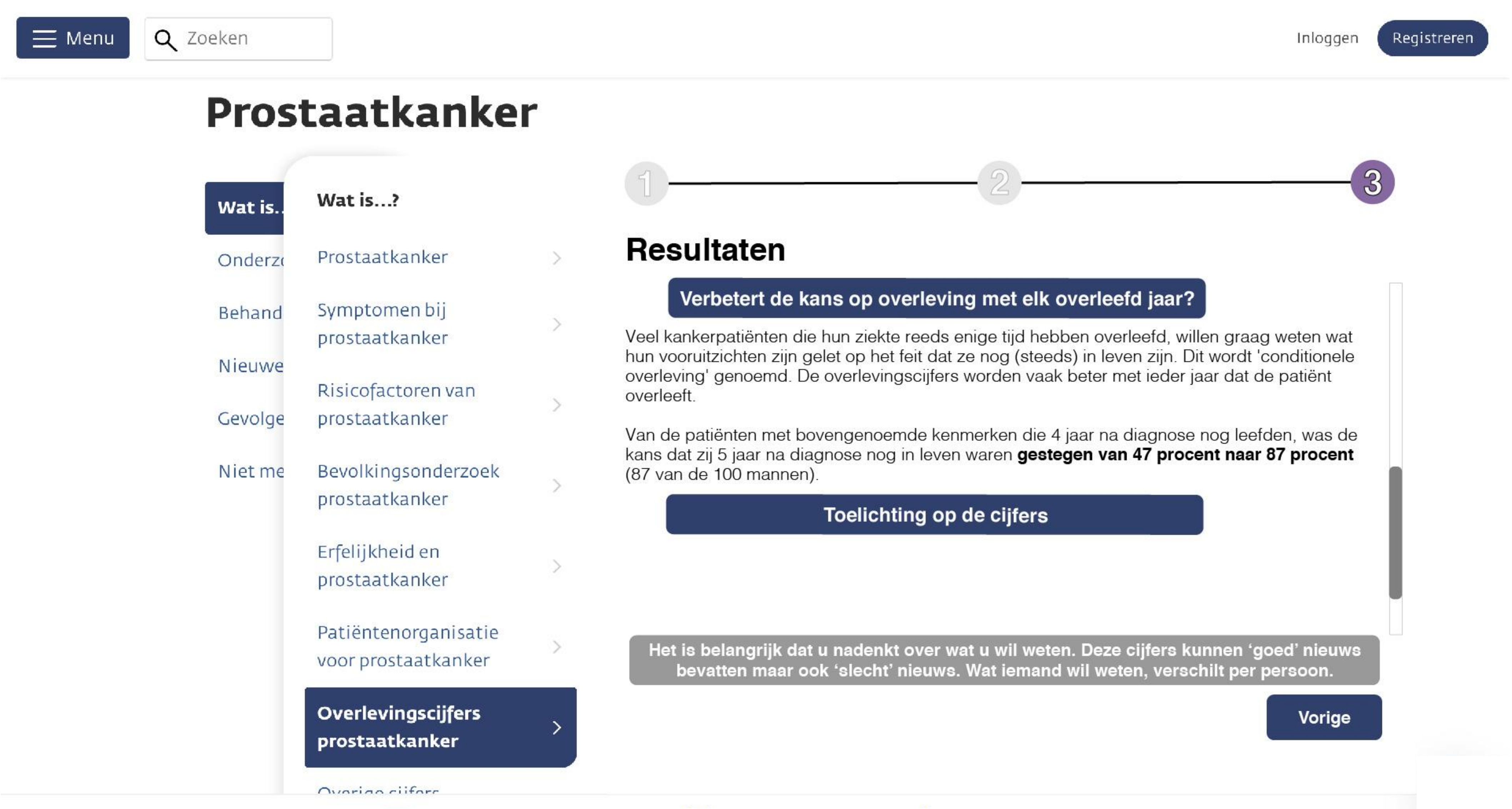

Translation:
Many patients with cancer who have survived for a couple of years want to know the chance that they will survive another x years (given that they are still alive). We call this conditional survival. In general, for cancer patients who have already survived a number of years, survival expectations at diagnosis are typically more favorable.
Of those patients like you who were still alive 4 years after diagnosis, the chance that they would still be alive 5 years after diagnosis increased from **47 percent to 87 percent** (87 out of 100 men).

Menu Zoeken Inloggen Registreren

Prostaatkanker

Wat is...

Onderz[illegible]

Behand[illegible]

Nieuwe[illegible]

Gevolge[illegible]

Niet me[illegible]

Wat is...?

- Prostaatkanker
- Symptomen bij prostaatkanker
- Risicofactoren van prostaatkanker
- Bevolkingsonderzoek prostaatkanker
- Erfelijkheid en prostaatkanker
- Patiëntenorganisatie voor prostaatkanker
- **Overlevingscijfers prostaatkanker**
- Overige cijfers

1 2 3

Resultaten

hun vooruitzichten zijn gelet op het feit dat ze nog (steeds) in leven zijn. Dit wordt 'conditionele overleving' genoemd. De overlevingscijfers worden vaak beter met ieder jaar dat de patiënt overleeft.

Van de patiënten met bovengenoemde kenmerken die 4 jaar na diagnose nog leefden, was de kans dat zij 5 jaar na diagnose nog in leven waren **gestegen van 47 procent naar 87 procent** (87 van de 100 mannen).

Toelichting op de cijfers

Bovenstaande cijfers zijn afkomstig uit de Nederlandse Kankerregistratie (NKR). Deze database bevat betrouwbare en objectieve gegevens over het voorkomen en de overleving van alle diagnoses kanker in Nederland. Het Integraal Kankercentrum Nederland (IKNL) beheert deze database.

Bovenstaande cijfers zijn tot en met 2017. Meer recente cijfers zijn op dit moment niet beschikbaar. De veranderingen per jaar zijn meestal gering. Bovenstaande cijfers kunnen een indruk geven voor latere jaren.

Het is belangrijk dat u nadenkt over wat u wil weten. Deze cijfers kunnen 'goed' nieuws bevatten maar ook 'slecht' nieuws. Wat iemand wil weten, verschilt per persoon.

Vorige

Translation:

About the statistics

The statistics that we show are based on data from the Netherlands Cancer Registry (NCR). This database contains reliable and objective data about the prevalence and survival of all cancer patients in the Netherlands. The Netherlands Comprehensive Cancer Organisation hosts this database.

The statistics that we showed are until 2017. More recent statistics are currently not available. However, annual changes are typically limited. The statistics provided here may therefore give an impression for future years.

Example 3: breast cancer

- Survival statistic: Favorable
- Version: Long and detailed version

Menu Zoeken Inloggen Registreren

Borstkanker

Algeme
Soorten
Onderz
De diag
Behand
Nieuwe
Uitgeza
Gevolge
Niet me
Over de

Algemeen

- Borstkanker
- Symptomen bij borstkanker
- Risicofactoren van borstkanker
- Erfelijke aanleg voor borstkanker
- Hormonen en borstkanker
- Dicht borstklierweefsel
- Bevolkingsonderzoek borstkanker
- **Overlevingscijfers borstkanker**

Overlevingscijfers borstkanker

De volgende pagina's geven cijfers over borstkanker. U kunt zien hoeveel mensen jaarlijks borstkanker krijgen en hoeveel mensen deze ziekte overleven. Deze statistieken geven een algemeen beeld voor de hele groep. Deze geven geen informatie over uw situatie. Uw vooruitzichten kunnen beter of slechter zijn dan de vooruitzichten voor de hele groep. Bespreek uw vooruitzichten met uw arts.

Wij vinden het belangrijk dat patiënten en naasten toegang hebben tot betrouwbare cijfers, maar alleen als iemand ook daadwerkelijk meer wil weten over cijfers. De statistische berekeningen worden gedaan met de gegevens uit de Nederlandse Kankerregistratie (NKR).

Als u vragen heeft, is het belangrijk om de betreffende pagina te printen en mee te nemen naar het ziekenhuis, zodat u de informatie kunt bespreken met uw arts.

Volgende

Translation:
Survival statistics breast cancer
The next pages will show you statistics about breast cancer. You will see how many women are diagnosed with breast cancer, and how many of these women will survive breast cancer. The statistics will give you a general idea for the entire group of patients. They do not say anything about your own situation. Your prognosis could be better or worse than the prognostic information that we show here. Please discuss your prognosis with your doctor.
We believe it is very important that patients and relatives have access to reliable statistics, but only if someone wants to know more about the statistics. The statistics are based on data from the Netherlands Cancer Registry (NCR). If you have any questions, you could always print the respective pages and take them with you to the next visit with your doctor, so you can discuss the results with him/her.

Menu Zoeken Inloggen Registreren

Borstkanker

Algeme
Soorten
Onderz
De diag
Behand
Nieuwe
Uitgeza
Gevolge
Niet me
Over de

Algemeen

- Borstkanker
- Symptomen bij borstkanker
- Risicofactoren van borstkanker
- Erfelijke aanleg voor borstkanker
- Hormonen en borstkanker
- Dicht borstklierweefsel
- Bevolkingsonderzoek borstkanker
- **Overlevingscijfers borstkanker**

1 2 3

Belangrijk om te weten voordat u verder gaat:

- Het is belangrijk dat u nadenkt over wat u wilt weten. Deze cijfers kunnen ‘goed’ nieuws bevatten maar ook ‘slecht’ nieuws. Wat iemand wil weten, verschilt per persoon.
- Alle getallen die wij geven, zijn gebaseerd op het verloop in het verleden. Onder invloed van betere diagnostiek en behandelingen, kunnen overlevingskansen voor huidige en toekomstige patiënten beter zijn dan de cijfers die wij hier tonen.
- Het lijkt soms alsof wij het heel ‘precies’ weten. Er is echter altijd sprake van onzekerheid. Hoe kleiner de aantallen, hoe groter de onzekerheid over het getal.

Vorige Volgende

Translation:

Important to know before you continue:

- It is important that you think about what you want to know. These statistics may contain ‘good’ or ‘bad’ news. What someone wants to know, differs per person.
- All statistics that we provide are based on what happened in the past. Given the improvements in terms of diagnosis and treatments, the survival statistics for recently diagnosed and future patients may be better than those statistics that we show here.
- It may seem that we know everything without any uncertainty. However, there is always uncertainty around statistics.

Menu Zoeken Inloggen Registreren

Borstkanker

Algeme
Soorten
Onderz
De diag
Behand
Nieuwe
Uitgeza
Gevolge
Niet me
Over de

Algemeen

- Borstkanker
- Symptomen bij borstkanker
- Risicofactoren van borstkanker
- Erfelijke aanleg voor borstkanker
- Hormonen en borstkanker
- Dicht borstklierweefsel
- Bevolkingsonderzoek borstkanker
- **Overlevingscijfers borstkanker**

1 2 3

U kunt ervoor kiezen algemene cijfers te zien, of gepersonaliseerde cijfers.

Wanneer u kiest voor **algemene cijfers**, krijgt u de cijfers van de gehele groep, zonder dat er rekening gehouden wordt met leeftijd, geslacht en ziektekenmerken. Deze kenmerken zijn wel van invloed op de kans op overleving.

Wanneer u kiest voor **gepersonaliseerde cijfers**, vragen wij u om een paar gegevens in te vullen. Daarna geven wij algemene cijfers aangevuld met de cijfers van de patiënten met dezelfde door u ingevulde kenmerken. Deze cijfers wijken af van de totale groep en kunnen uw situatie meer benaderen.

Het is belangrijk om u te realiseren dat het altijd gaat om resultaten voor een groep en dus nooit precies past op uw situatie. Gemiddeld kan de overleving 5 jaar zijn, maar dat betekent dat er een aantal mensen zijn die korter leven en een aantal die langer leven. In welke groep u gaat vallen, is niet te voorspellen.

Wilt u liever algemene cijfers zien, of gepersonaliseerde cijfers?

Algemene cijfers **Gepersonaliseerde cijfers**

Translation:

You may choose to see generic statistics, of personalized statistics. If you click on **generic statistics**, you will see statistics for all breast cancer patients, without taking into account factors such as age, gender, or disease-related factors. These factors do have an influence on survival. If you click on **personalized statistics**, you will be asked to enter some data, after which you will be presented with statistics that are based on patients like you (with the same personal and disease-related characteristics).

These personalized statistics deviate from the generic, group-based statistics, and may therefore better relate to your own situation. It is important to realize that we will always present statistics that are applicable to a group (and they will never really apply to your situation). For example, the average survival rate could be 5 years, but that means that some patients will have a shorter life, whereas other will life a bit longer. We cannot predict in which category or group you will belong to. Would you like to see generic statistics, or personalized statistics?

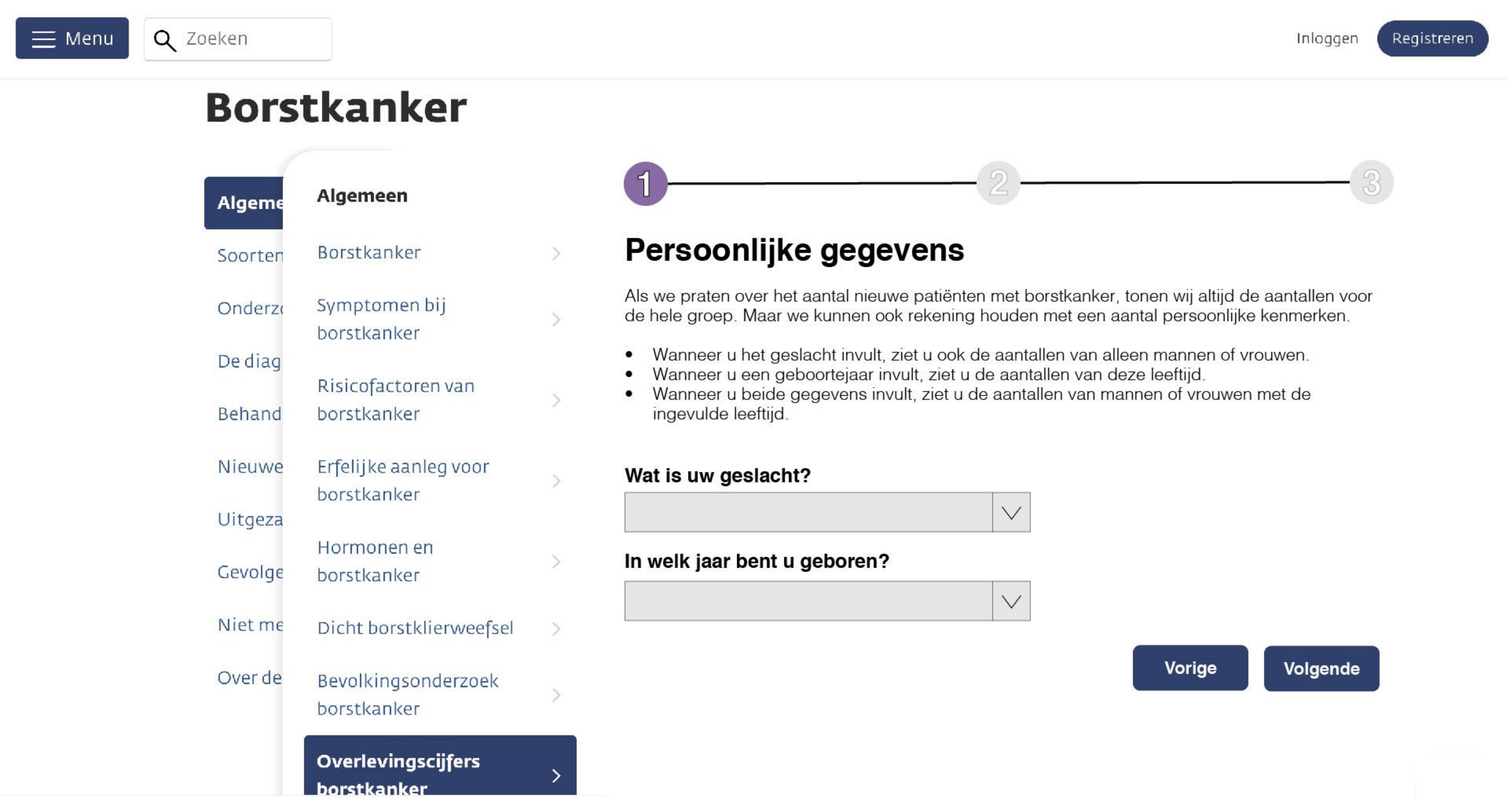

Translation:
Personal data
If we talk about the number of recently diagnosed patients with breast cancer, we always show group-based statistics. However, we can also take a number of personal characteristics into account. In case you enter your gender, you will only see statistics for either men or women. If you enter your year of birth, you will see the number of patients of that particular age. You can also enter both characteristics.

- What is your gender?
- What is your year of birth?

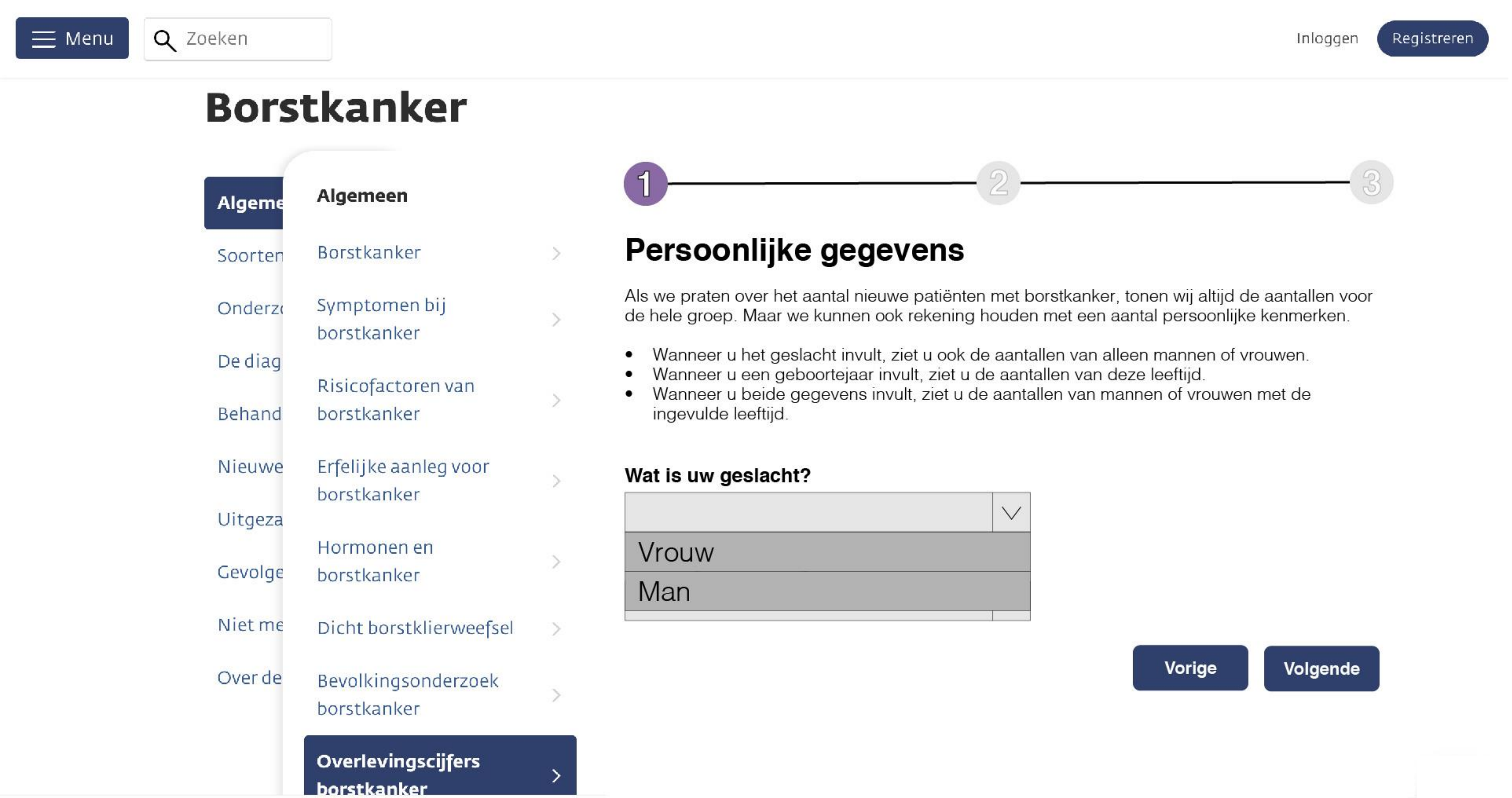

Translation:

Personal data

If we talk about the number of recently diagnosed patients with breast cancer, we always show group-based statistics. However, we can also take a number of personal characteristics into account. In case you enter your gender, you will only see statistics for either men or women. If you enter your year of birth, you will see the number of patients of that particular age. You can also enter both characteristics.

- What is your gender? (woman, men)
- What is your year of birth?

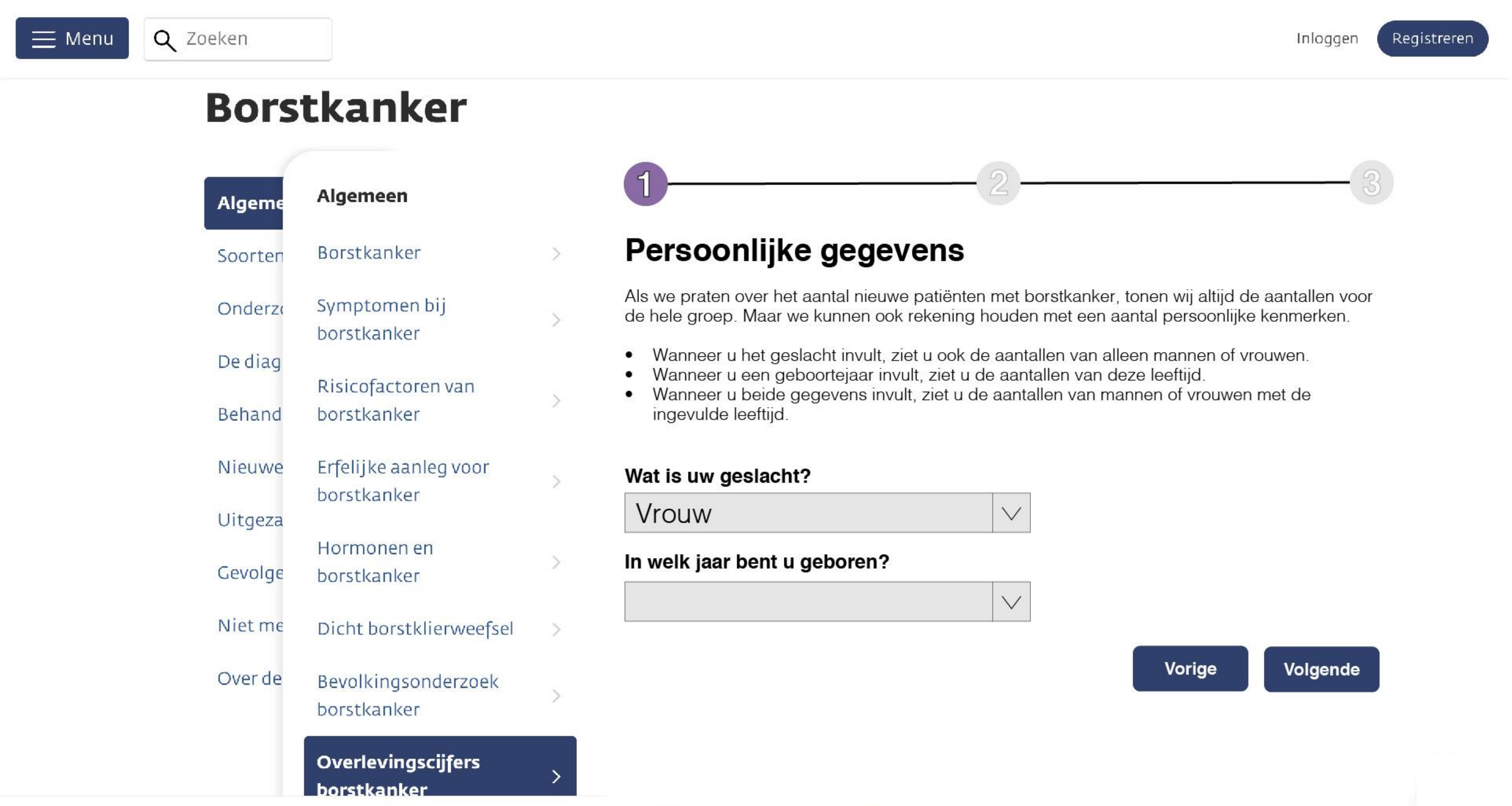

Translation:
Personal data
If we talk about the number of recently diagnosed patients with breast cancer, we always show group-based statistics. However, we can also take a number of personal characteristics into account. In case you enter your gender, you will only see statistics for either men or women. If you enter your year of birth, you will see the number of patients of that particular age. You can also enter both characteristics.

- What is your gender? (woman)
- What is your year of birth?

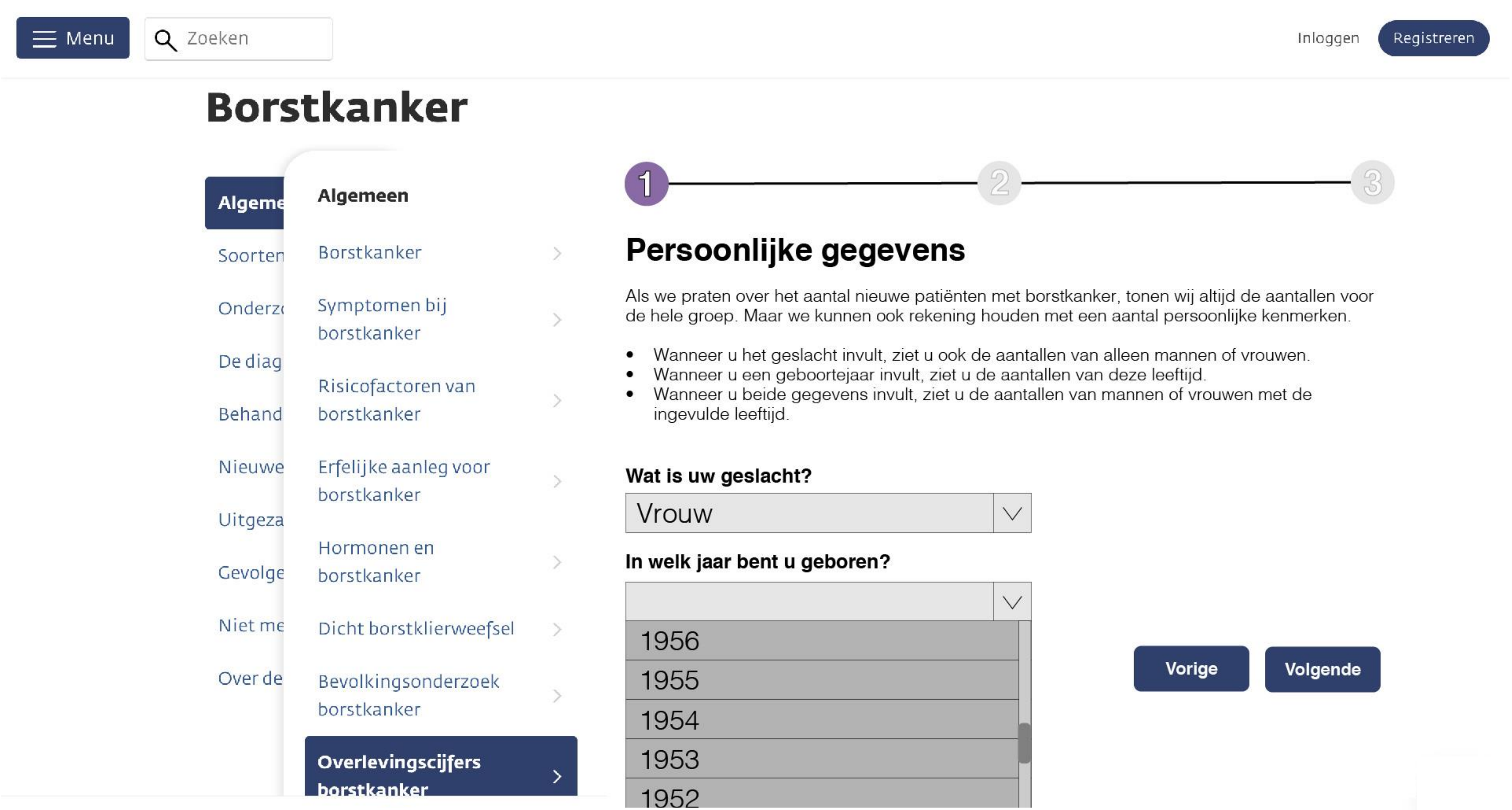

Translation:

Personal data

If we talk about the number of recently diagnosed patients with breast cancer, we always show group-based statistics. However, we can also take a number of personal characteristics into account. In case you enter your gender, you will only see statistics for either men or women. If you enter your year of birth, you will see the number of patients of that particular age. You can also enter both characteristics.

- What is your gender? (woman)
- What is your year of birth?

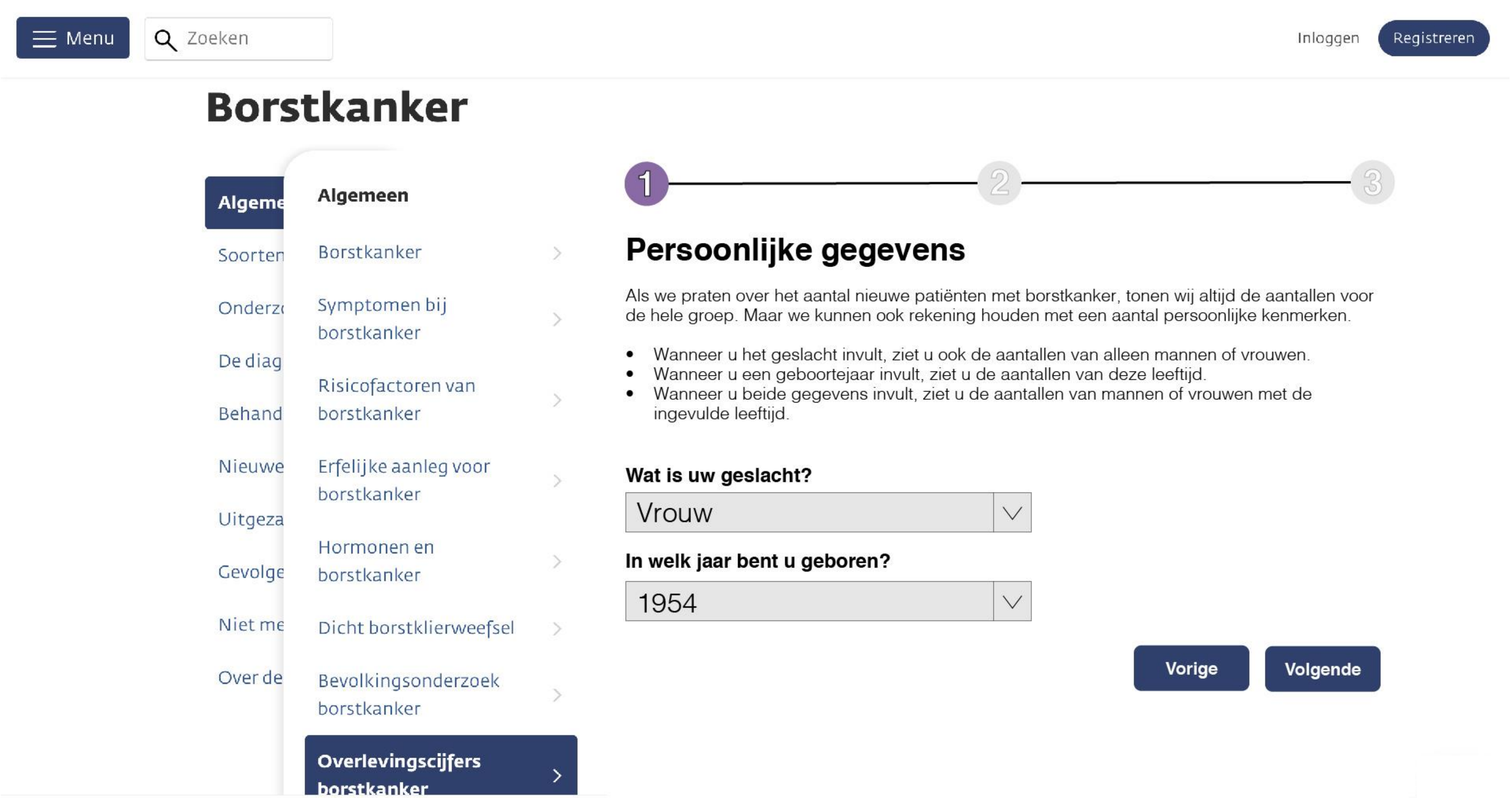

Translation:

Personal data

If we talk about the number of recently diagnosed patients with breast cancer, we always show group-based statistics. However, we can also take a number of personal characteristics into account. In case you enter your gender, you will only see statistics for either men or women. If you enter your year of birth, you will see the number of patients of that particular age. You can also enter both characteristics.

- What is your gender? (woman)
- What is your year of birth? (1954)

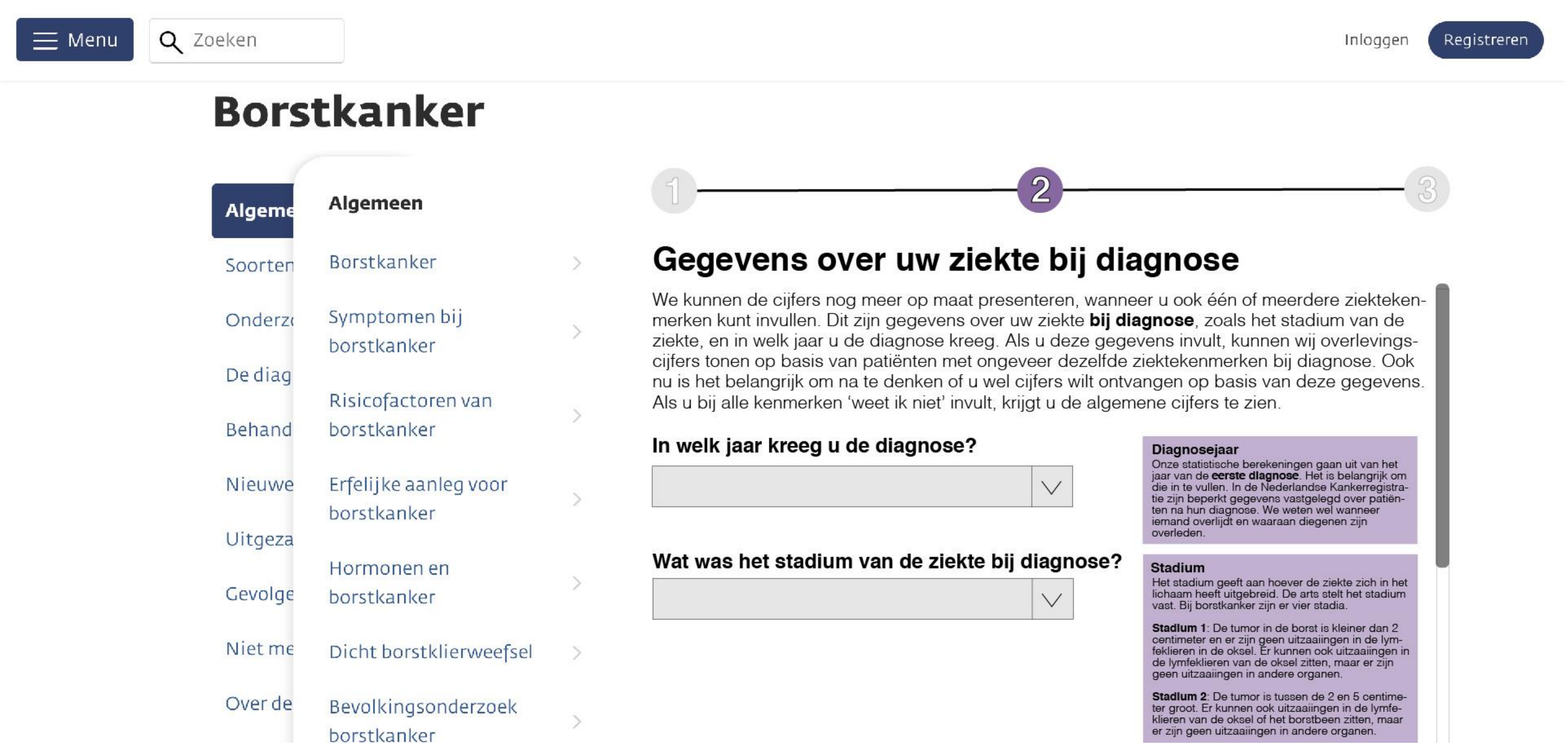

Translation:

Disease-related data

We can further personalize the statistics, for instance if you enter some disease-related characteristics **at your diagnosis**, such as the tumor stage, or the year of diagnosis. If you enter these data, we can show statistics based on patients like you with similar characteristics at their diagnosis. Again, please think about whether you really want to see personalized statistics. If you enter “I don’t know” for each characteristic, you will only see the generic statistics.

- Year of diagnosis? (Our calculations are based on the first cancer diagnosis. It is important to fill out this one, because the Netherlands Cancer Registry contains limited data of patients after their diagnosis. However, the NCR does know when a patient died (and its cause)).
- Tumor stage at diagnosis? The tumor stage says something about how far the cancer has progressed. The doctor will determine the tumor stage. For breast cancer, there are four stages. **Stage 1:** the tumor is smaller than 2 cm but it has not spread to the nearby lymph nodes or to distant organs. **Stage 2:** the tumor size is between 2 and 5 cm, and may have spread to lymph nodes under the arm or near the breast bone, but not to distant organs.

Menu Zoeken Inloggen Registreren

Borstkanker

Algeme[illegible]
Soorten[illegible]
Onderz[illegible]
De diag[illegible]
Behand[illegible]
Nieuwe[illegible]
Uitgeza[illegible]
Gevolge[illegible]
Niet me[illegible]
Over de[illegible]

Algemeen

- Borstkanker
- Symptomen bij borstkanker
- Risicofactoren van borstkanker
- Erfelijke aanleg voor borstkanker
- Hormonen en borstkanker
- Dicht borstklierweefsel
- Bevolkingsonderzoek borstkanker
- **Overlevingscijfers borstkanker**

1 — 2 — 3

Gegevens over uw ziekte bij diagnose

We kunnen de cijfers nog meer op maat presenteren, wanneer u ook één of meerdere ziektekenmerken kunt invullen. Dit zijn gegevens over uw ziekte **bij diagnose**, zoals het stadium van de ziekte, en in welk jaar u de diagnose kreeg. Als u deze gegevens invult, kunnen wij overlevingscijfers tonen op basis van patiënten met ongeveer dezelfde ziektekenmerken bij diagnose. Ook nu is het belangrijk om na te denken of u wel cijfers wilt ontvangen op basis van deze gegevens. Als u bij alle kenmerken 'weet ik niet' invult, krijgt u de algemene cijfers te zien.

In welk jaar kreeg u de diagnose?

- Weet ik niet
- 2019
- 2018
- 2017
- 2016
- 2015

Diagnosejaar
Onze statistische berekeningen gaan uit van het jaar van de **eerste diagnose**. Het is belangrijk om die in te vullen. In de Nederlandse Kankerregistratie zijn beperkt gegevens vastgelegd over patiënten na hun diagnose. We weten wel wanneer iemand overlijdt en waaraan diegenen zijn overleden.

Stadium
Het stadium geeft aan hoever de ziekte zich in het lichaam heeft uitgebreid. De arts stelt het stadium vast. Bij borstkanker zijn er vier stadia.

Stadium 1: De tumor in de borst is kleiner dan 2 centimeter en er zijn geen uitzaaiingen in de lymfeklieren in de oksel. Er kunnen ook uitzaaiingen in de lymfeklieren van de oksel zitten, maar er zijn geen uitzaaiingen in andere organen.

Stadium 2: De tumor is tussen de 2 en 5 centimeter groot. Er kunnen ook uitzaaiingen in de lymfeklieren van de oksel of het borstbeen zitten, maar er zijn geen uitzaaiingen in andere organen.

Stadium 3: De tumor is groter dan 5 centimeter

Vorige | Toon de cijfers

Menu Zoeken Inloggen Registreren

Borstkanker

Algeme
Soorten
Onderz
De diag
Behand
Nieuwe
Uitgeza
Gevolge
Niet me
Over de

Algemeen

- Borstkanker
- Symptomen bij borstkanker
- Risicofactoren van borstkanker
- Erfelijke aanleg voor borstkanker
- Hormonen en borstkanker
- Dicht borstklierweefsel
- Bevolkingsonderzoek borstkanker

1 2 3

Gegevens over uw ziekte bij diagnose

We kunnen de cijfers nog meer op maat presenteren, wanneer u ook één of meerdere ziektekenmerken kunt invullen. Dit zijn gegevens over uw ziekte **bij diagnose**, zoals het stadium van de ziekte, en in welk jaar u de diagnose kreeg. Als u deze gegevens invult, kunnen wij overlevingscijfers tonen op basis van patiënten met ongeveer dezelfde ziektekenmerken bij diagnose. Ook nu is het belangrijk om na te denken of u wel cijfers wilt ontvangen op basis van deze gegevens. Als u bij alle kenmerken 'weet ik niet' invult, krijgt u de algemene cijfers te zien.

In welk jaar kreeg u de diagnose?

2016

Wat was het stadium van de ziekte bij diagnose?

Diagnosejaar
Onze statistische berekeningen gaan uit van het jaar van de **eerste diagnose**. Het is belangrijk om die in te vullen. In de Nederlandse Kankerregistratie zijn beperkt gegevens vastgelegd over patiënten na hun diagnose. We weten wel wanneer iemand overlijdt en waaraan diegenen zijn overleden.

Stadium
Het stadium geeft aan hoever de ziekte zich in het lichaam heeft uitgebreid. De arts stelt het stadium vast. Bij borstkanker zijn er vier stadia.

Stadium 1: De tumor in de borst is kleiner dan 2 centimeter en er zijn geen uitzaaiingen in de lymfeklieren in de oksel. Er kunnen ook uitzaaiingen in de lymfeklieren van de oksel zitten, maar er zijn geen uitzaaiingen in andere organen.

Stadium 2: De tumor is tussen de 2 en 5 centimeter groot. Er kunnen ook uitzaaiingen in de lymfeklieren van de oksel of het borstbeen zitten, maar er zijn geen uitzaaiingen in andere organen.

Translation:
Disease-related data

- Year of diagnosis? (2016)
- Tumor stage at diagnosis?

- **Stage 3:** the tumor is locally advanced. The tumor has progressed and is more likely to grow and spread. **Stage 4:** the tumor has spread to lymph nodes or to other parts of the body.

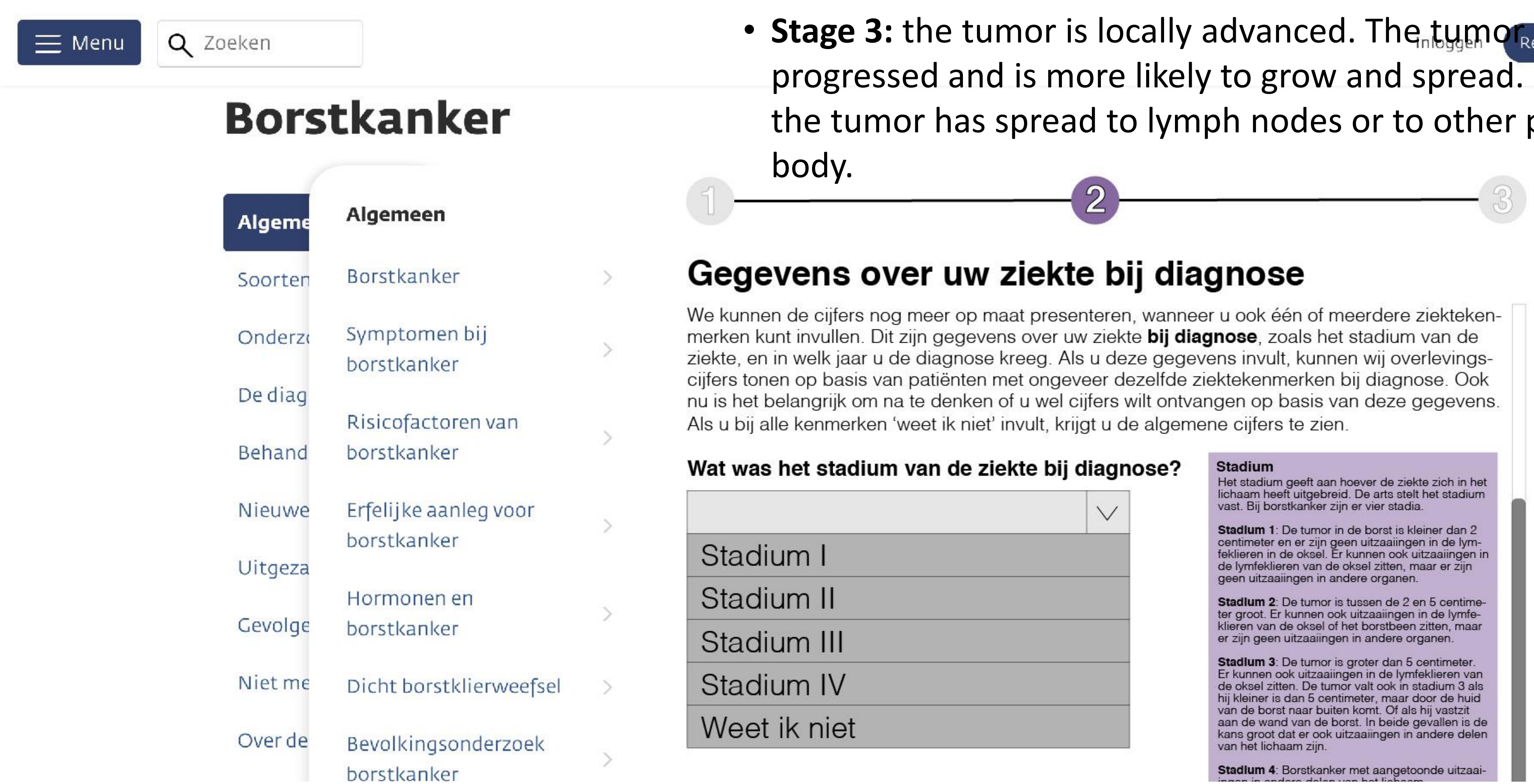

Translation:

Disease-related data

- Year of diagnosis? (2016)
- Tumor stage at diagnosis? (The tumor stage says something about how far the cancer has progressed. The doctor will determine the tumor stage. For breast cancer, there are four stages. **Stage 1:** the tumor is smaller than 2 cm but it has not spread to the nearby lymph nodes or to distant organs. **Stage 2:** the tumor size is between 2 and 5 cm, and may have spread to lymph nodes under the arm or near the breast bone, but not to distant organs. **Stage 3:** The size of the tumor is bigger than 5 cm, and it may have spread to the nearby lymph nodes under the arm. The tumor may also be smaller than 3 cm, but may have spread to the chest wall and/or skin of the breast. **Stage 4:** The tumor has spread beyond the breast and nearby lymph nodes to other organs of the body.

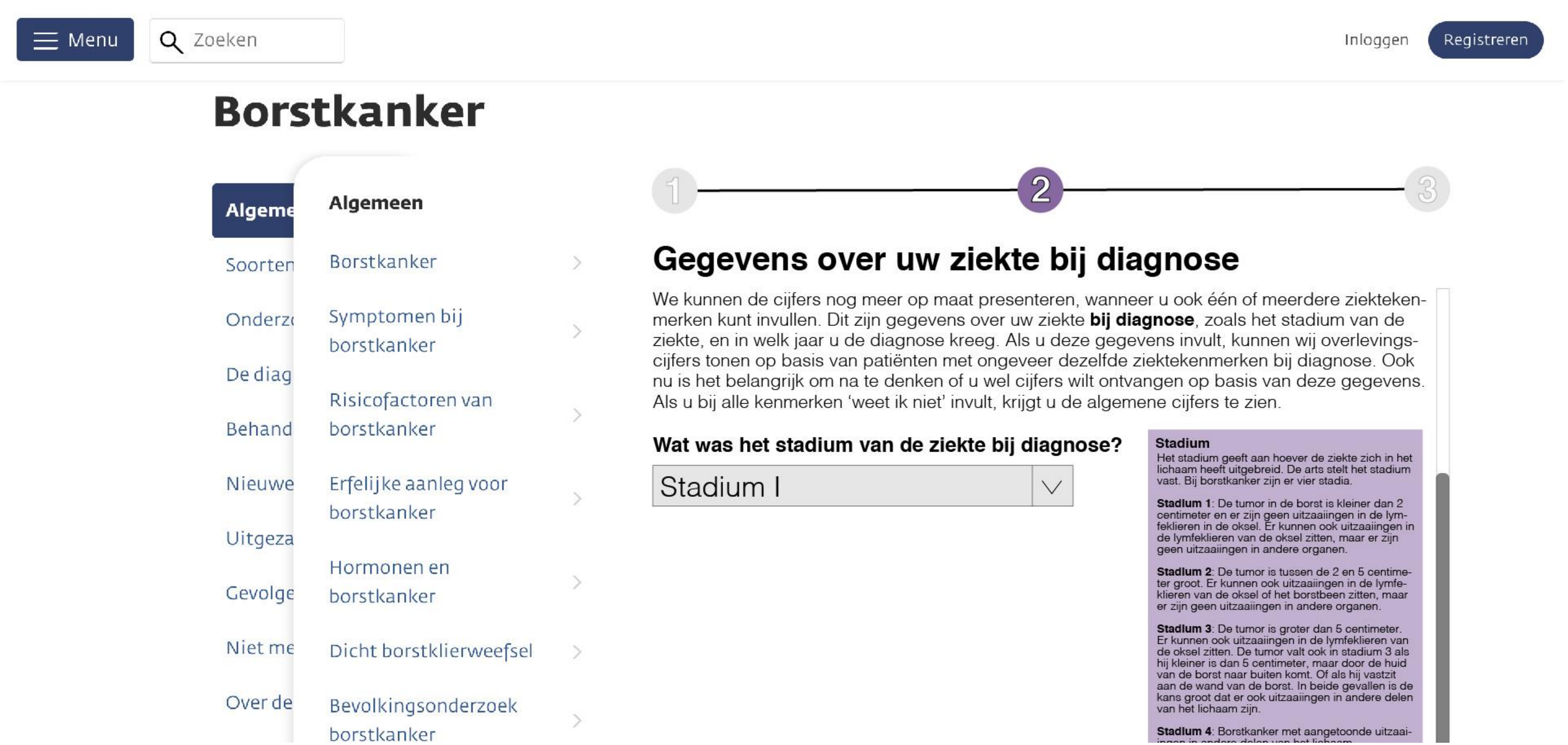

Translation:

Disease-related data

- Year of diagnosis? (2016)
- Tumor stage at diagnosis? (stage I)

Menu Zoeken Inloggen Registreren

Borstkanker

Algemeen
Soorten
Onderz
De diag
Behand
Nieuwe
Uitgeza
Gevolge
Niet me
Over de

Algemeen

- Borstkanker
- Symptomen bij borstkanker
- Risicofactoren van borstkanker
- Erfelijke aanleg voor borstkanker
- Hormonen en borstkanker
- Dicht borstklierweefsel
- Bevolkingsonderzoek borstkanker
- **Overlevingscijfers borstkanker**

1 — 2 — 3

Resultaten

Op deze pagina ziet u cijfers over borstkanker. U heeft aangegeven om gepersonaliseerde cijfers te ontvangen, zodat deze uw situatie meer benaderen.

De cijfers die wij tonen, zullen daarom gebaseerd zijn op **vrouwen** die op **65-jarige leeftijd** de diagnose borstkanker **stadium I** kregen.

Hoeveel vrouwen kregen de diagnose borstkanker in 2016?

Hoeveel vrouwen overleven borstkanker?

Het is belangrijk dat u nadenkt over wat u wil weten. Deze cijfers kunnen 'goed' nieuws bevatten maar ook 'slecht' nieuws. Wat iemand wil weten, verschilt per persoon.

Vorige

Translation:
Results
On this page, you will see some breast cancer statistics. You have indicated to receive personalized statistics that are closer to your personal situation. For this reason, the statistics will be based on women **aged 65**, diagnosed with **breast cancer stage 1.**
How many women were diagnosed with breast cancer in 2016?
How many women survive breast cancer?

It is important that you think about what you would like to know. These statistics can contain 'good' but also 'bad' news. What someone wants to know differs per person.

Menu Zoeken Inloggen Registreren

Borstkanker

Algeme
Soorten
Onderz
De diag
Behand
Nieuwe
Uitgeza
Gevolge
Niet me
Over de

Algemeen

- Borstkanker
- Symptomen bij borstkanker
- Risicofactoren van borstkanker
- Erfelijke aanleg voor borstkanker
- Hormonen en borstkanker
- Dicht borstklierweefsel
- Bevolkingsonderzoek borstkanker
- **Overlevingscijfers borstkanker**

1 — 2 — 3

Resultaten

Op deze pagina ziet u cijfers over borstkanker. U heeft aangegeven om gepersonaliseerde cijfers te ontvangen, zodat deze uw situatie meer benaderen.

De cijfers die wij tonen, zullen daarom gebaseerd zijn op **vrouwen** die op **65-jarige leeftijd** de diagnose borstkanker **stadium I** kregen.

Hoeveel vrouwen kregen de diagnose borstkanker in 2016?

In **2016** kregen in totaal 14.752 vrouwen de diagnose borstkanker. Als we rekening houden met de bovengenoemde kenmerken, kregen in 2016 81 vrouwen de diagnose borstkanker stadium I op 65-jarige leeftijd.

Hoeveel vrouwen overleven borstkanker?

Het is belangrijk dat u nadenkt over wat u wil weten. Deze cijfers kunnen 'goed' nieuws bevatten maar ook 'slecht' nieuws. Wat iemand wil weten, verschilt per persoon.

Vorige

Translation:
In **2016**, in total 14.752 women were diagnosed with breast cancer. If we take the above mentioned characteristics into account, 81 women like you aged 65 were diagnosed with breast cancer stage I in 2016.

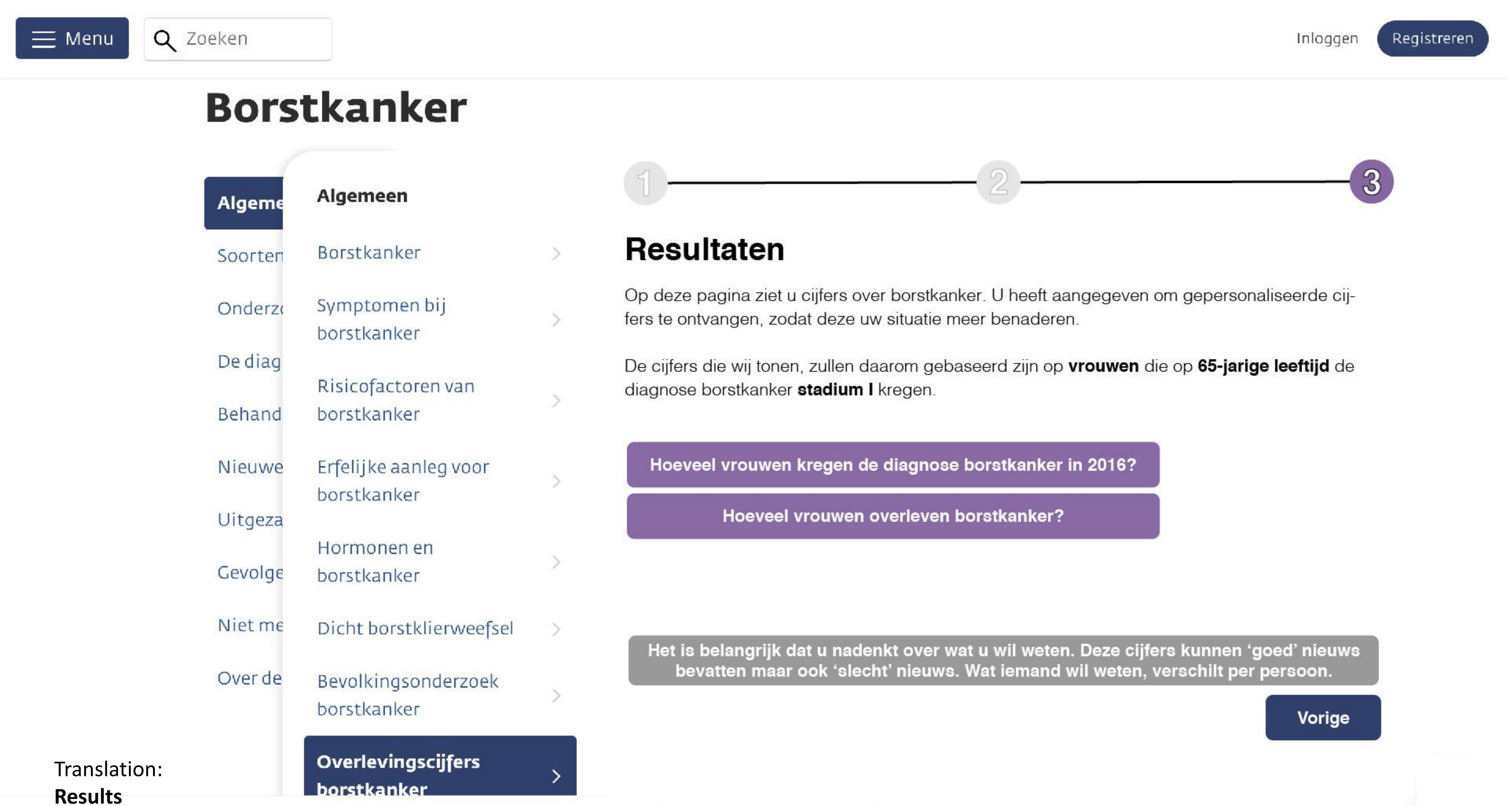

Translation:
Results
On this page, you will see some breast cancer statistics. You have indicated to receive personalized statistics that are closer to your personal situation. For this reason, the statistics will be based on women **aged 65**, diagnosed with **breast cancer stage 1.**
How many women were diagnosed with breast cancer in 2016?
How many women survive breast cancer?

It is important that you think about what you would like to know. These statistics can contain 'good' but also 'bad' news. What someone wants to know differs per person.

Menu Zoeken Inloggen Registreren

Borstkanker

Algemeen

Soorten

Onderz

De diag

Behand

Nieuwe

Uitgeza

Gevolge

Niet me

Over de

Algemeen

Borstkanker

Symptomen bij borstkanker

Risicofactoren van borstkanker

Erfelijke aanleg voor borstkanker

Hormonen en borstkanker

Dicht borstklierweefsel

Bevolkingsonderzoek borstkanker

1 2 3

Resultaten

Hoeveel vrouwen overleven borstkanker?

Met **overleving** bedoelen we hoeveel mensen met een bepaalde vorm van kanker na een bepaalde periode nog in leven zijn. In dit geval laten we zien hoeveel vrouwen met de diagnose borstkanker 5 jaar na diagnose nog in leven zijn.

Om betrouwbare getallen te geven, moet er gerekend worden met grote aantallen patiënten die in het verleden borstkanker hebben gehad. De cijfers die wij hier tonen zullen gebaseerd zijn op patiënten met de bovengenoemde kenmerken die tussen 2012 en 2017 de diagnose borstkanker kregen.

We laten eerst de kans op overleving zien van alle patiënten met borstkanker. 5 jaar na diagnose was ongeveer **88 procent** van de borstkanker patiënten nog in leven (88 van de 100 vrouwen).

We kunnen ook de kans op overleving laten zien van borstkanker patiënten die bij diagnose de bovengenoemde kenmerken hadden. 5 jaar na diagnose was ongeveer **89 procent** van deze patiënten nog in leven (89 van de 100 vrouwen).

Het is belangrijk dat u nadenkt over wat u wil weten. Deze cijfers kunnen 'goed' nieuws bevatten maar ook 'slecht' nieuws. Wat iemand wil weten, verschilt per persoon.

Vorige

Translation:
With **survival** we mean the percentage of people with cancer still alive after a particular amount of time. Here we will show you how many women are still alive 5 years after diagnosis.
In order to provide you with reliable statistics we need to perform statistics on a large group of patients that have been diagnosed with breast cancer in the past. The statistics that we show here are performed on data from patients diagnosed between 2012 and 2017.
We will first show you the chance of survival for all women with breast cancer. 5 years after diagnosis, about **88 percent** of these patients with breast cancer are alive (88 out of 100 women). We can also show you the chance of survival for women with breast cancer diagnosed with the above mentioned characteristics. 5 years after diagnosis, about **89 percent** of these patients with breast cancer are alive (89 out of 100 women).

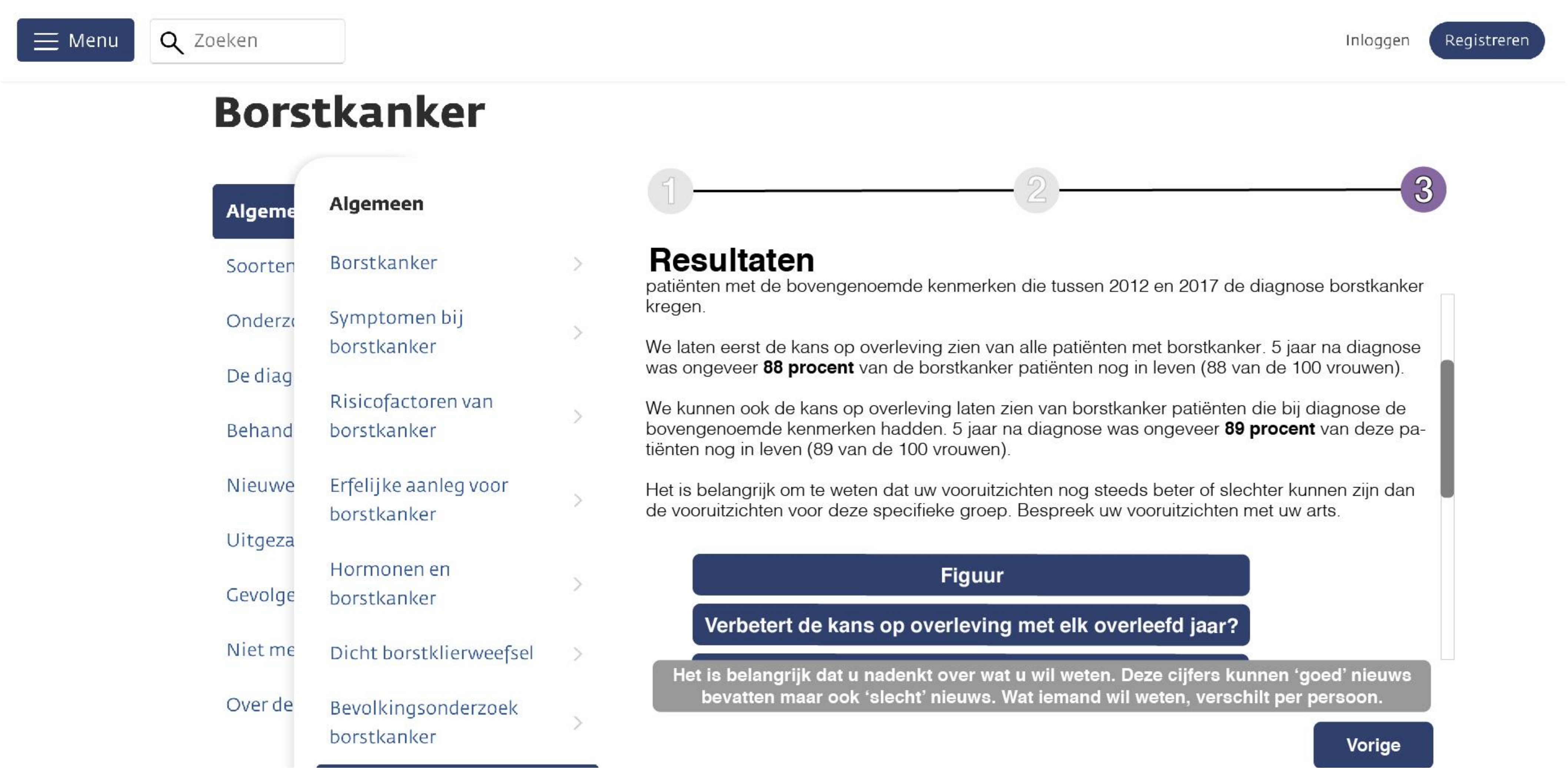

Translation:
We will first show you the chance of survival for all women with breast cancer. 5 years after diagnosis, about **88 percent** of these patients with breast cancer are alive (88 out of 100 women). We can also show you the chance of survival for women with breast cancer diagnosed with the above mentioned characteristics. 5 years after diagnosis, about **89 percent** of these patients with breast cancer are alive (89 out of 100 women). It is important to know that your prognosis can still be better or worse than the prognosis of this specific group of patients. Discuss your prognosis with your doctor.
Figure
What is the survival statistic after having survived a period of time?

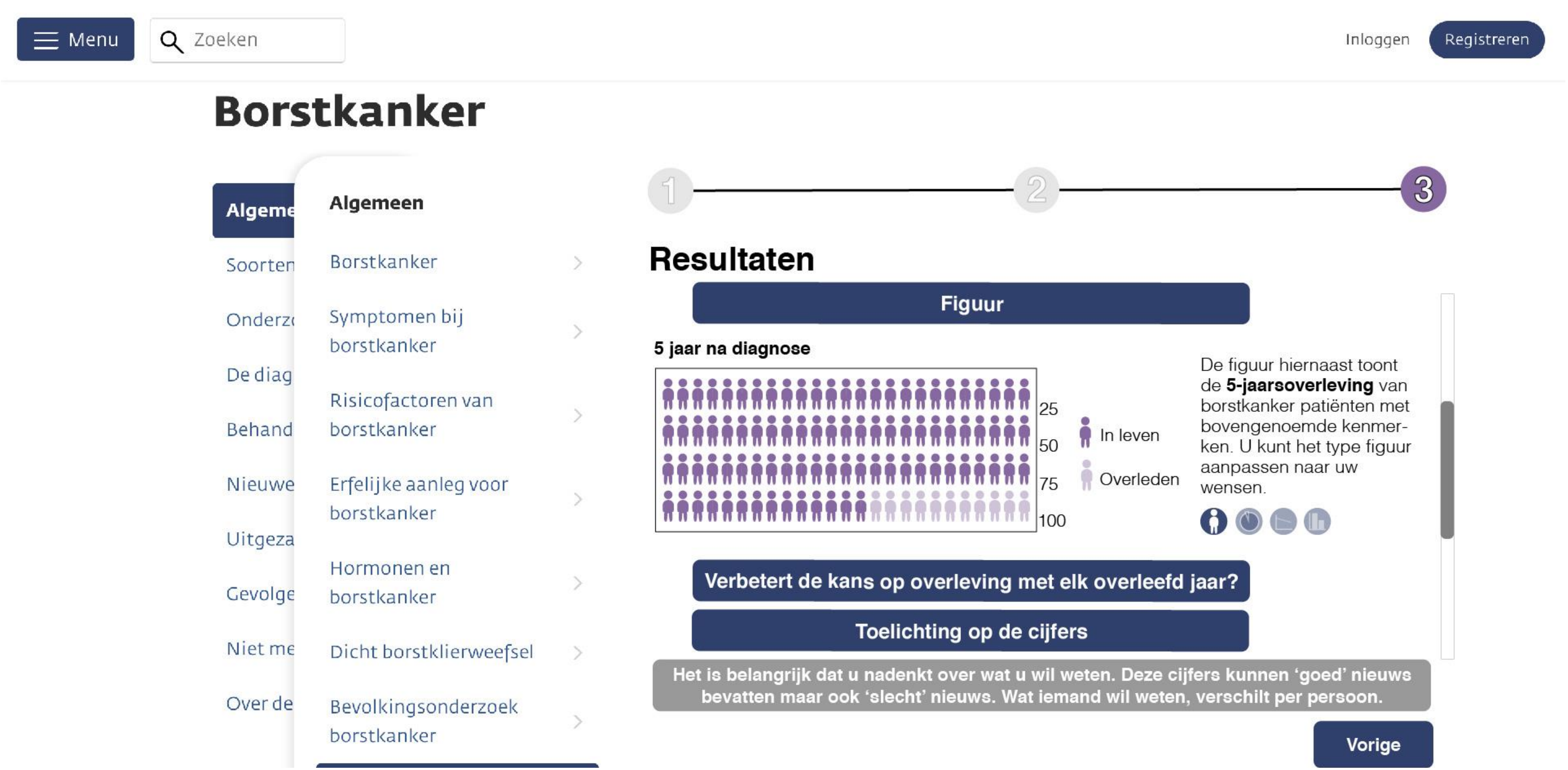

Translation:
This figure shows the **5 year survival statistic** for breast cancer with the above mentioned characteristics. You can change the type of figure.
In leven = are alive
Overleden = die

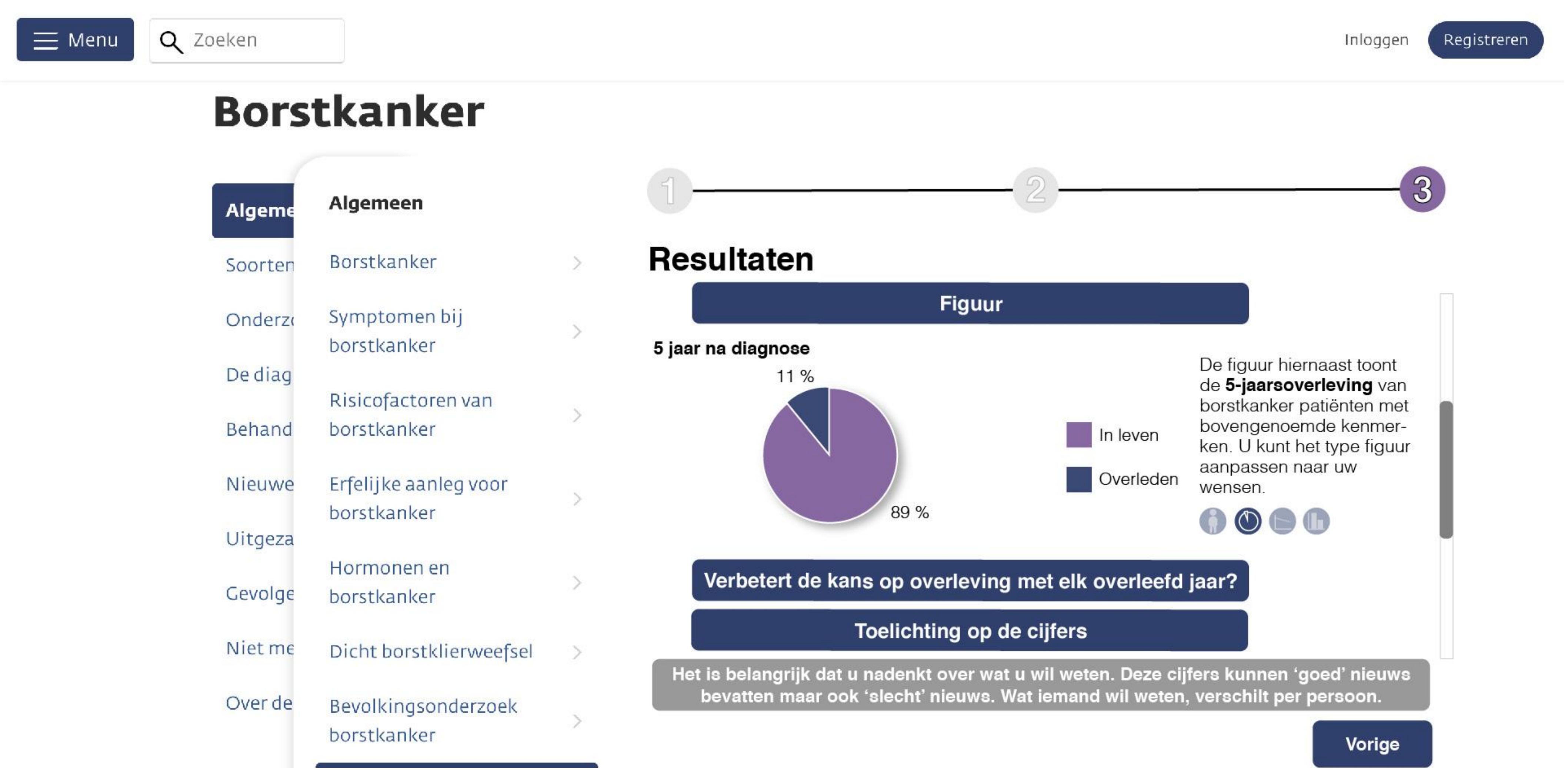

Translation:
This figure shows the **5 year survival statistic** for breast cancer with the above mentioned characteristics. You can change the type of figure.
In leven = are alive
Overleden = die

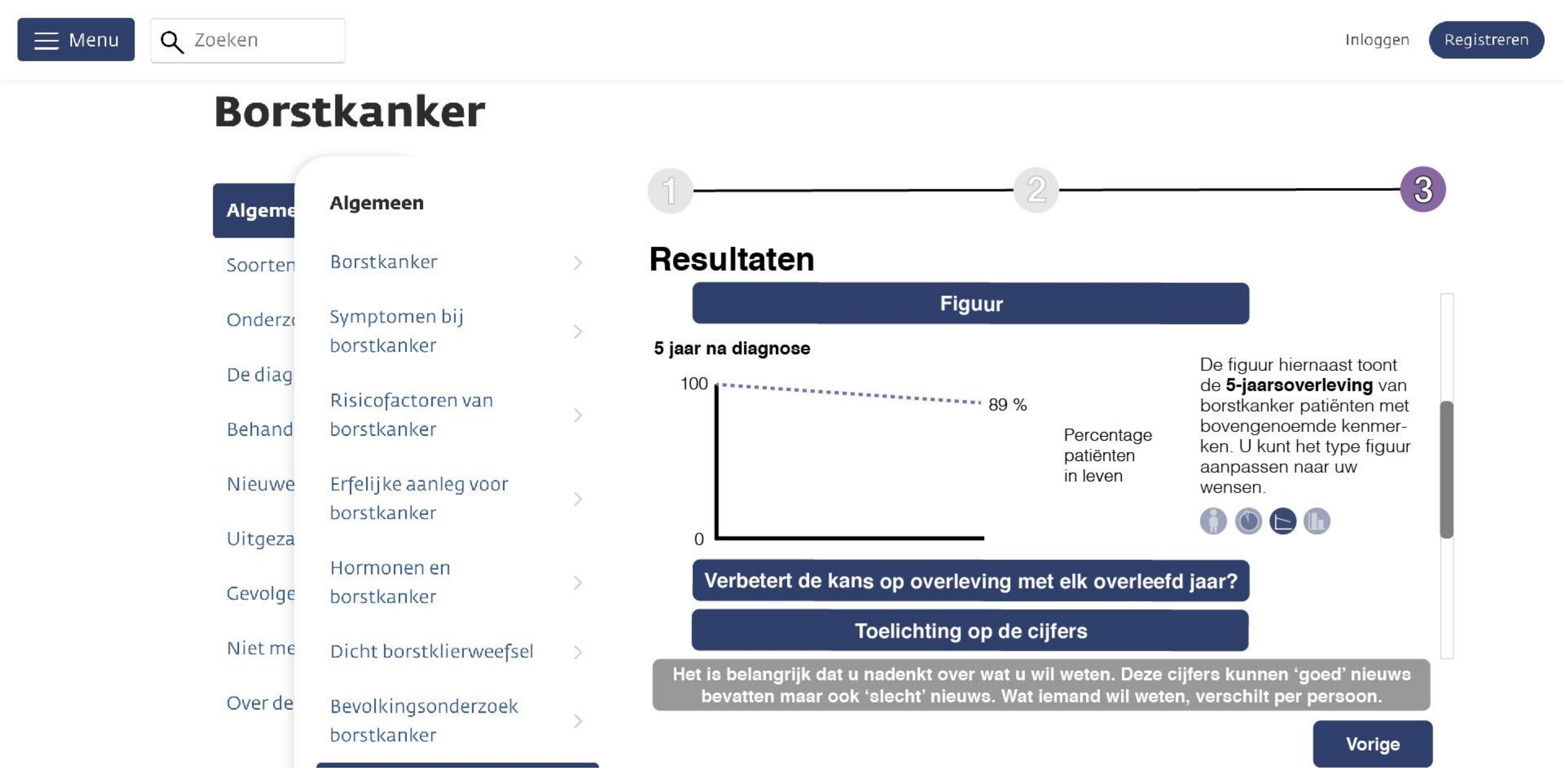

Translation:
This figure shows the **5 year survival statistic** for breast cancer with the above mentioned characteristics. You can change the type of figure.
In leven = are alive
Overleden = die

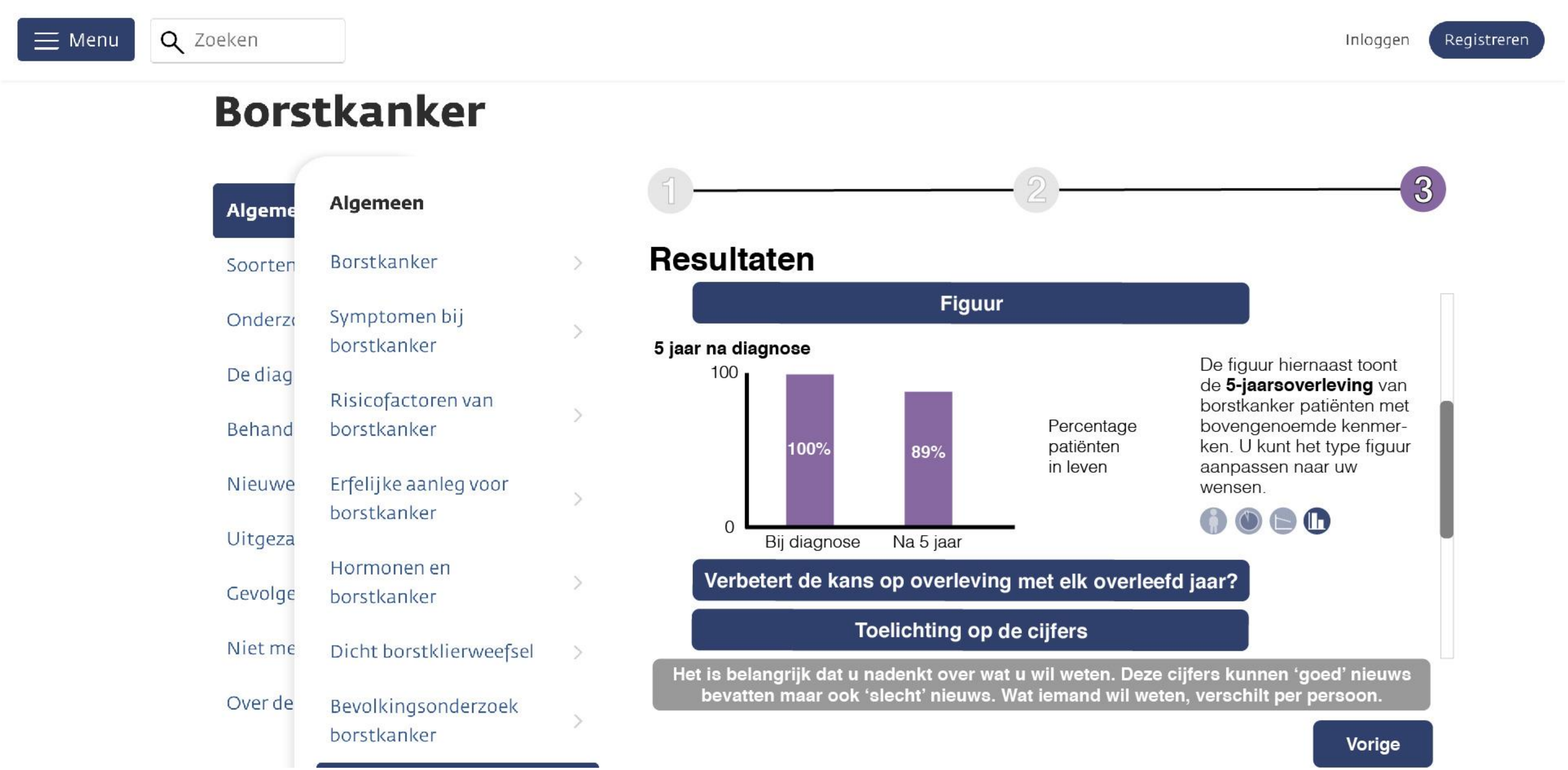

Translation:
This figure shows the **5 year survival statistic** for breast cancer with the above mentioned characteristics. You can change the type of figure.
In leven = are alive
Overleden = die

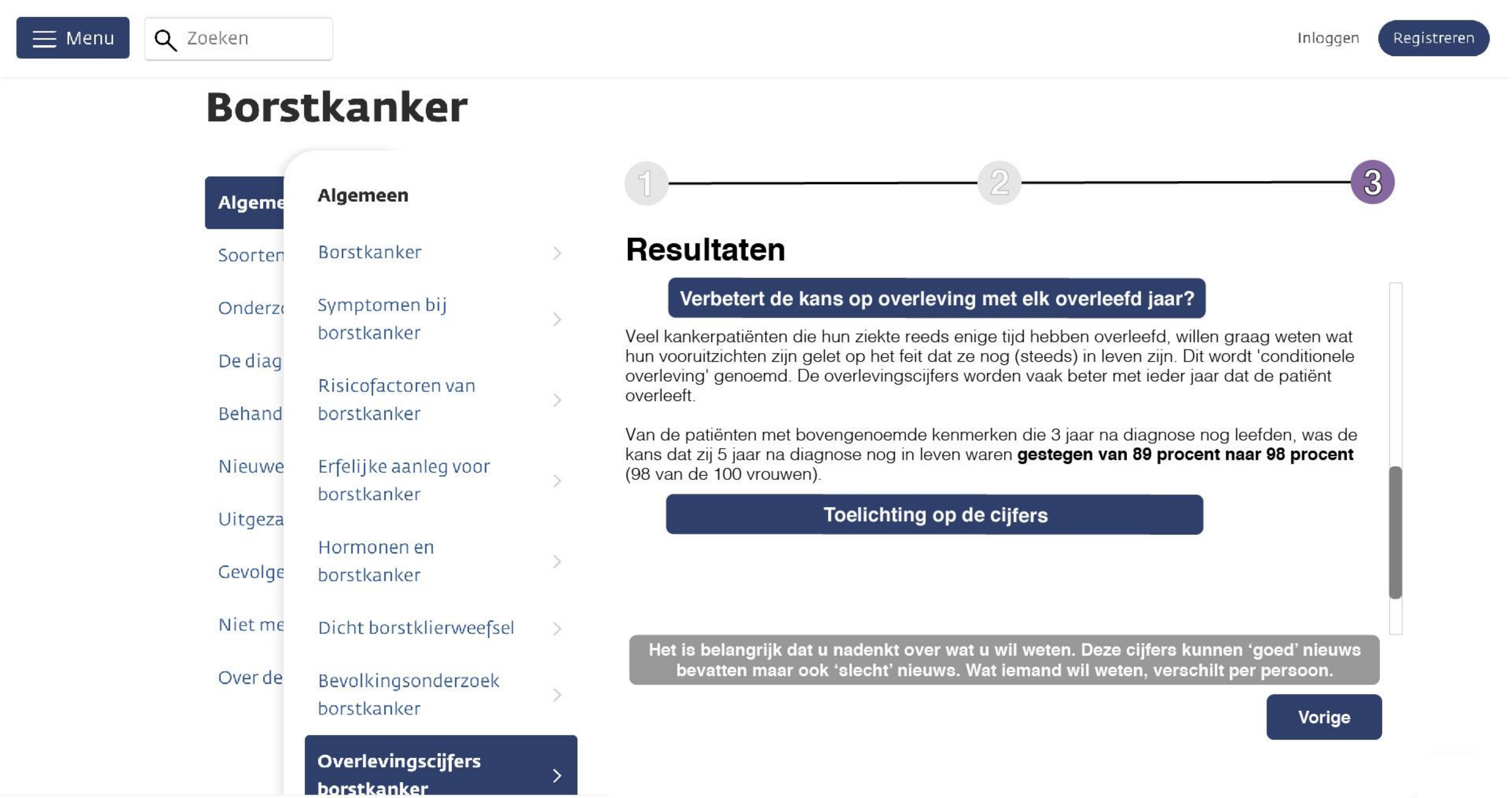

Translation:
Many patients with cancer who have survived for a couple of years want to know the chance that they will survive another x years (given that they are still alive). We call this conditional survival. In general, for cancer patients who have already survived a number of years, survival expectations at diagnosis are typically more favorable.
Of those patients like you who were still alive 3 years after diagnosis, the chance that they would still be alive 5 years after diagnosis increased from **89 percent to 98 percent** (98 out of 100 women).

Menu Zoeken Inloggen Registreren

Borstkanker

Algemeen
Soorten
Onderz
De diag
Behand
Nieuwe
Uitgeza
Gevolge
Niet me
Over de

Algemeen

- Borstkanker
- Symptomen bij borstkanker
- Risicofactoren van borstkanker
- Erfelijke aanleg voor borstkanker
- Hormonen en borstkanker
- Dicht borstklierweefsel
- Bevolkingsonderzoek borstkanker
- **Overlevingscijfers borstkanker**

1 2 3

Resultaten

hun vooruitzichten zijn gelet op het feit dat ze nog (steeds) in leven zijn. Dit wordt 'conditionele overleving' genoemd. De overlevingscijfers worden vaak beter met ieder jaar dat de patiënt overleeft.

Van de patiënten met bovengenoemde kenmerken die 3 jaar na diagnose nog leefden, was de kans dat zij 5 jaar na diagnose nog in leven waren **gestegen van 89 procent naar 98 procent** (98 van de 100 vrouwen).

Toelichting op de cijfers

Bovenstaande cijfers zijn afkomstig uit de Nederlandse Kankerregistratie (NKR). Deze database bevat betrouwbare en objectieve gegevens over het voorkomen en de overleving van alle diagnoses kanker in Nederland. Het Integraal Kankercentrum Nederland (IKNL) beheert deze database.

Bovenstaande cijfers zijn tot en met 2017. Meer recente cijfers zijn op dit moment niet beschikbaar. De veranderingen per jaar zijn meestal gering. Bovenstaande cijfers kunnen een indruk geven voor latere jaren.

Het is belangrijk dat u nadenkt over wat u wil weten. Deze cijfers kunnen 'goed' nieuws bevatten maar ook 'slecht' nieuws. Wat iemand wil weten, verschilt per persoon.

Vorige

Translation:
About the statistics
The statistics that we show are based on data from the Netherlands Cancer Registry (NCR). This database contains reliable and objective data about the prevalence and survival of all cancer patients in the Netherlands. The Netherlands Comprehensive Cancer Organisation hosts this database.
The statistics that we showed are until 2017. More recent statistics are currently not available. However, annual changes are typically limited. The statistics provided here may therefore give an impression for future years.

Example 4: breast cancer

- Survival statistic: Less favorable
- Version: Short and concise version

Menu Zoeken

Inloggen Registreren

Borstkanker

Algeme
Soorten
Onderz
De diag
Behand
Nieuwe
Uitgeza
Gevolge
Niet me
Over de

Algemeen

Borstkanker
Symptomen bij borstkanker
Risicofactoren van borstkanker
Erfelijke aanleg voor borstkanker
Hormonen en borstkanker
Dicht borstklierweefsel
Bevolkingsonderzoek borstkanker
Overlevingscijfers borstkanker

Overlevingscijfers borstkanker

De volgende pagina's geven cijfers over borstkanker. U kunt zien hoeveel mensen jaarlijks borstkanker krijgen en hoeveel mensen deze ziekte overleven. De statistische berekeningen worden gedaan met de gegevens uit de Nederlandse Kankerregistratie (NKR).

Als u vragen heeft, is het belangrijk om de betreffende pagina te printen en mee te nemen naar het ziekenhuis, zodat u de informatie kunt bespreken met uw arts.

Volgende

Translation:

Survival statistics breast cancer

The next pages will show you statistics about breast cancer. You will see how many women are diagnosed with breast cancer, and how many of these women will survive breast cancer. The statistics are based on data from the Netherlands Cancer Registry (NCR).

If you have any questions, you could always print the respective pages and take them with you to the next visit with your doctor, so you can discuss the results with him/her.

Menu Zoeken Inloggen Registreren

Borstkanker

Algeme
Soorten
Onderz
De diag
Behand
Nieuwe
Uitgeza
Gevolge
Niet me
Over de

Algemeen

Borstkanker
Symptomen bij borstkanker
Risicofactoren van borstkanker
Erfelijke aanleg voor borstkanker
Hormonen en borstkanker
Dicht borstklierweefsel
Bevolkingsonderzoek borstkanker
Overlevingscijfers borstkanker

1 2 3

Belangrijk om te weten voordat u verder gaat:

- Het is belangrijk dat u nadenkt over wat u wilt weten. Deze cijfers kunnen 'goed' nieuws bevatten maar ook 'slecht' nieuws. Wat iemand wil weten, verschilt per persoon.
- Alle getallen die wij geven, zijn gebaseerd op het verloop in het verleden. Onder invloed van betere diagnostiek en behandelingen, kunnen overlevingskansen voor huidige en toekomstige patiënten beter zijn dan de cijfers die wij hier tonen.
- Het lijkt soms alsof wij het heel 'precies' weten. Er is echter altijd sprake van onzekerheid. Hoe kleiner de aantallen, hoe groter de onzekerheid over het getal.

Vorige Volgende

Translation:

Important to know before you continue:

- It is important that you think about what you want to know. These statistics may contain 'good' or 'bad' news. What someone wants to know, differs per person.
- All statistics that we provide are based on what happened in the past. Given the improvements in terms of diagnosis and treatments, the survival statistics for recently diagnosed and future patients may be better than those statistics that we show here.
- It may seem that we know everything without any uncertainty. However, there is always uncertainty around statistics.

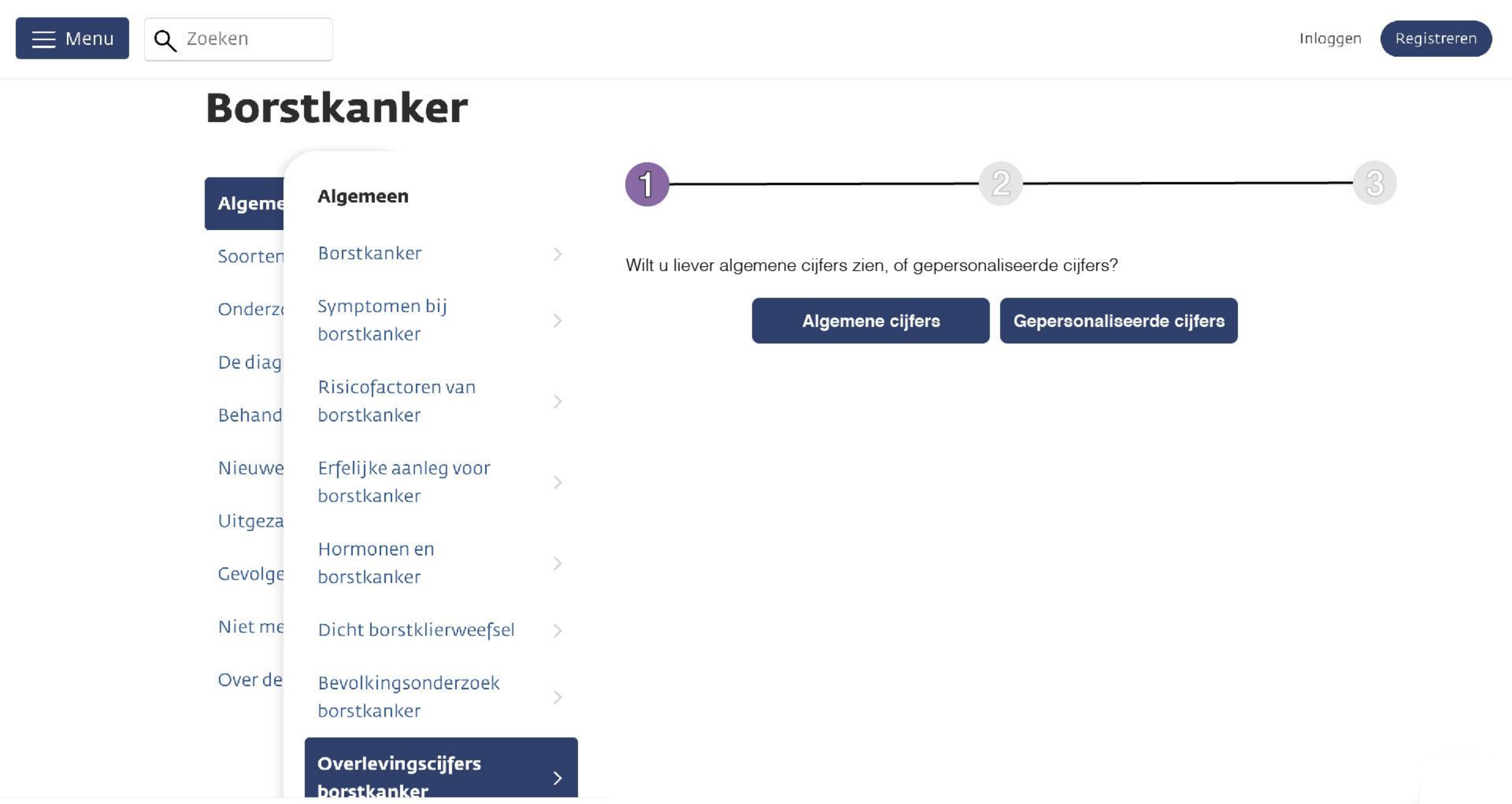

Translation:
Would you like to generic statistics, or personalized statistics?

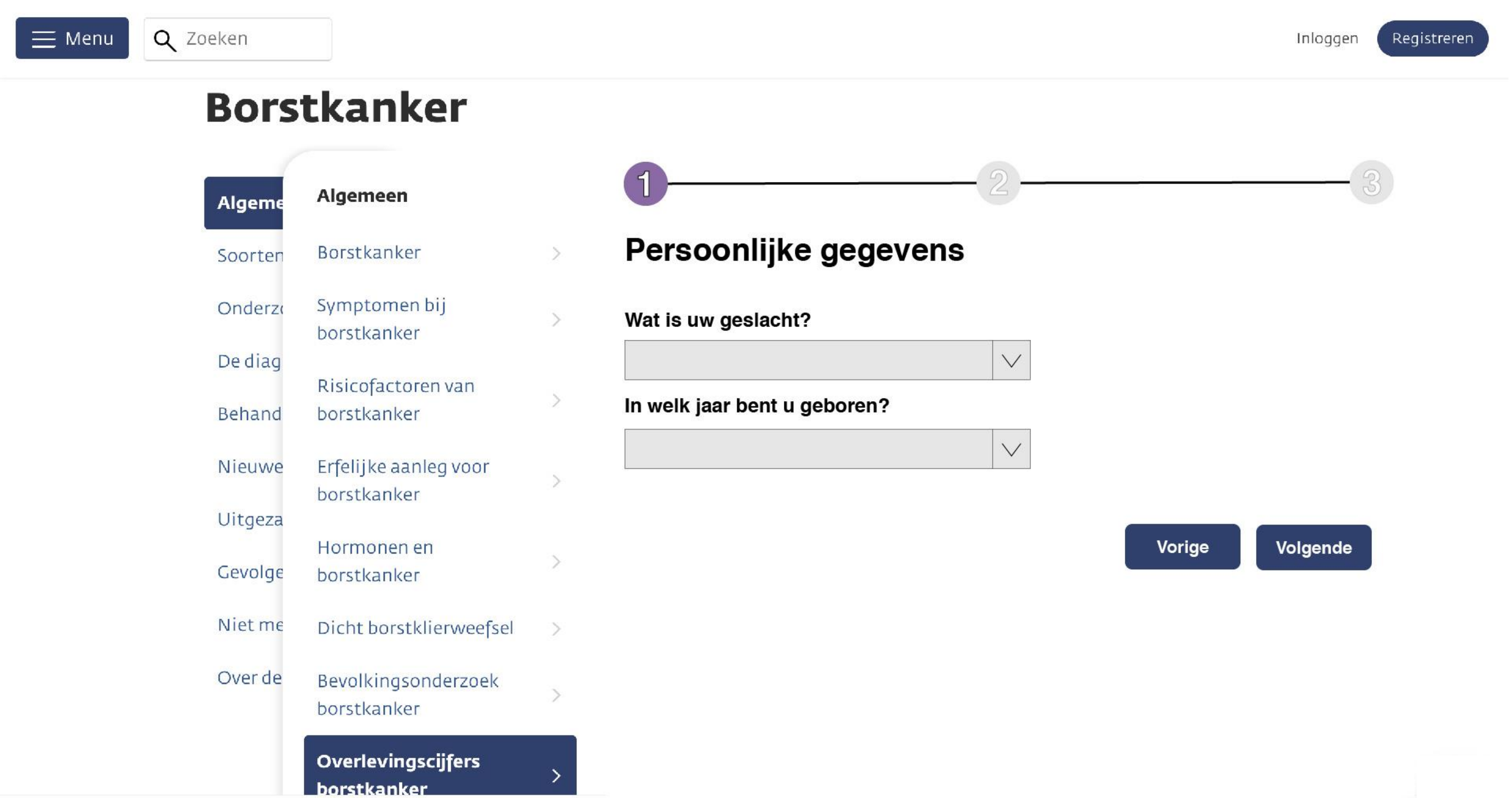

Translation:

Personal data

- What is your gender?
- What is your year of birth?

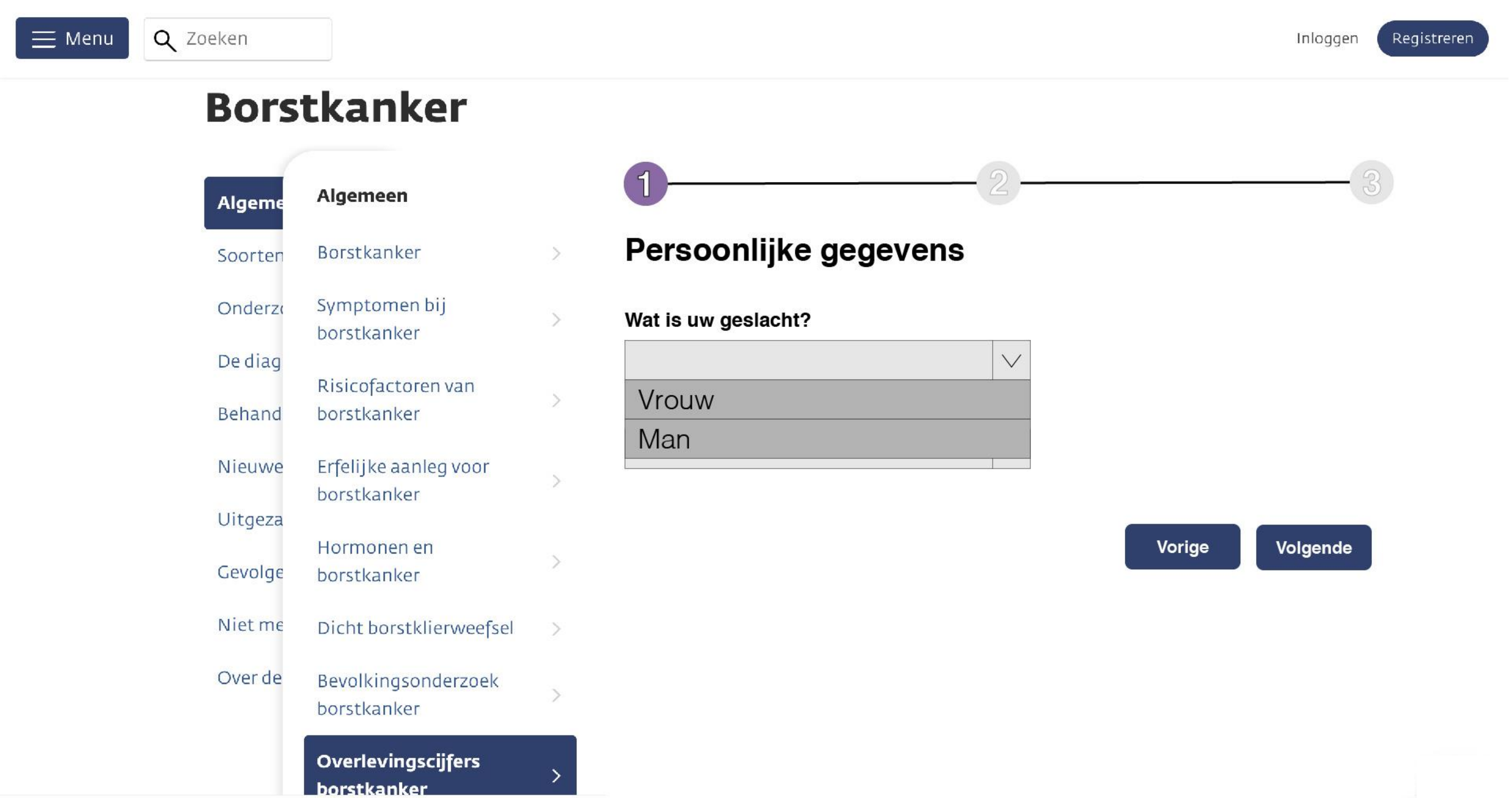

Translation:

Personal data

- What is your gender? (woman, man)
- What is your year of birth?

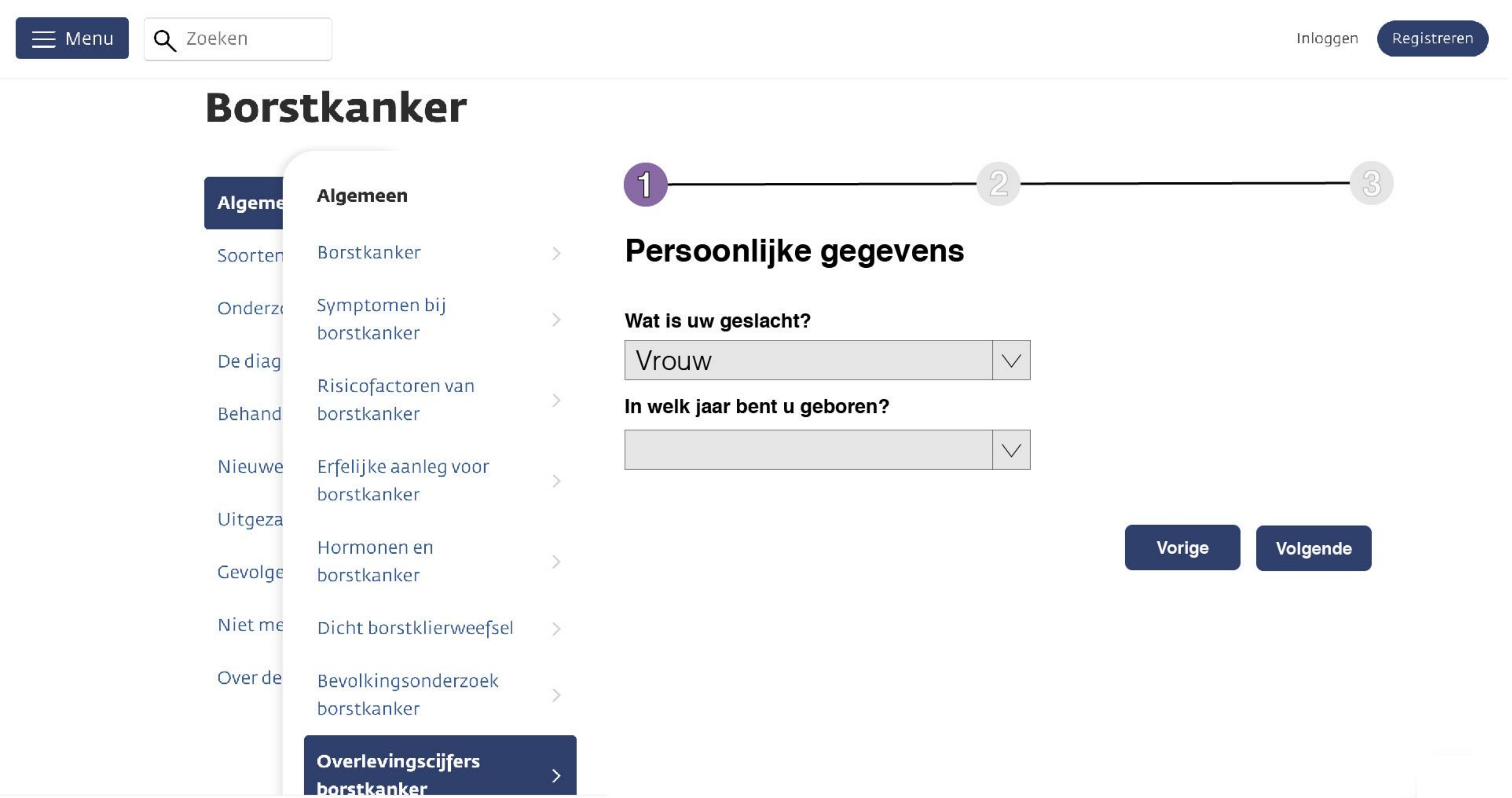

Translation:

Personal data

- What is your gender? (woman)
- What is your year of birth?

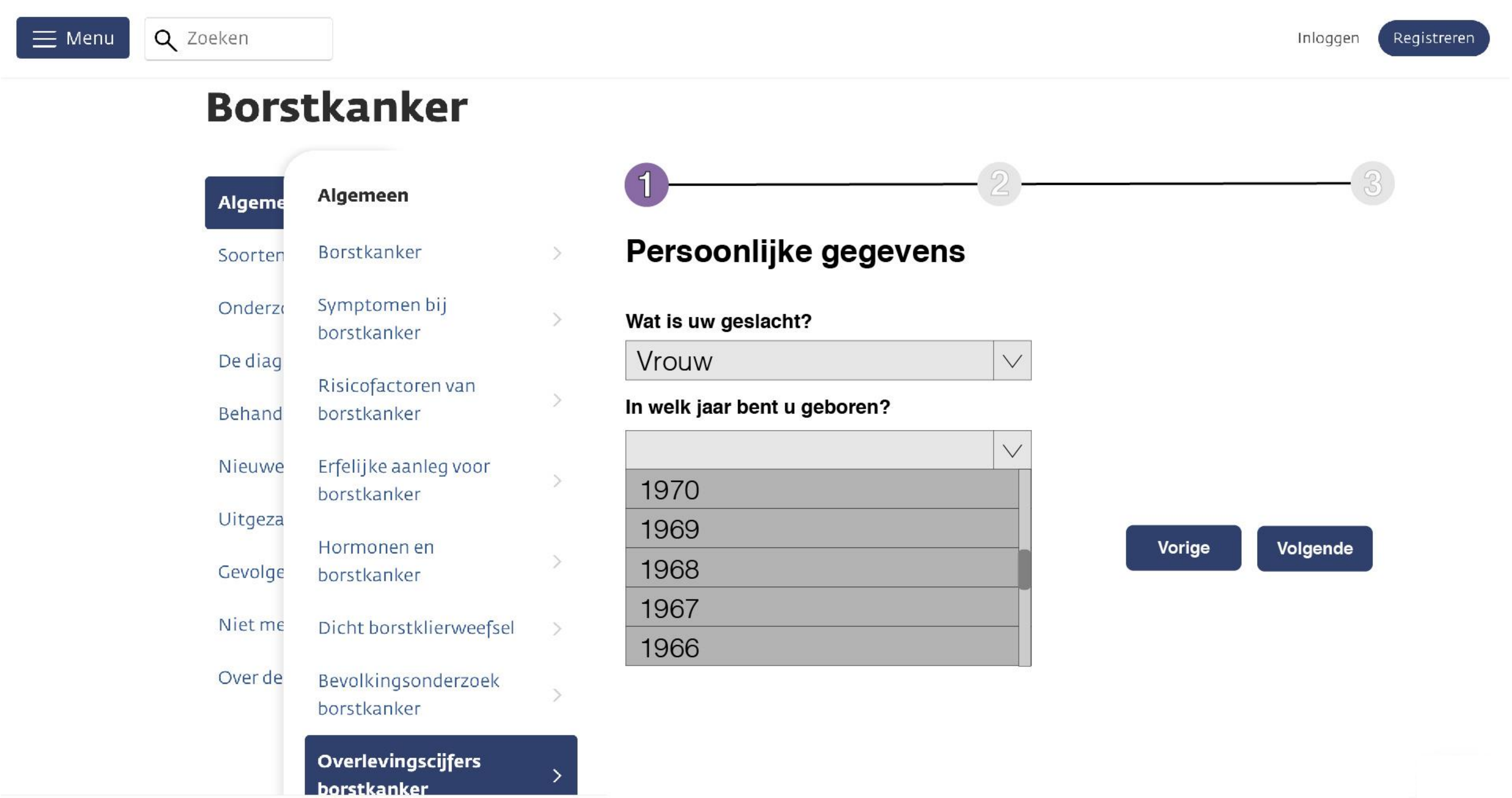

Translation:

Personal data

- What is your gender? (woman)
- What is your year of birth?

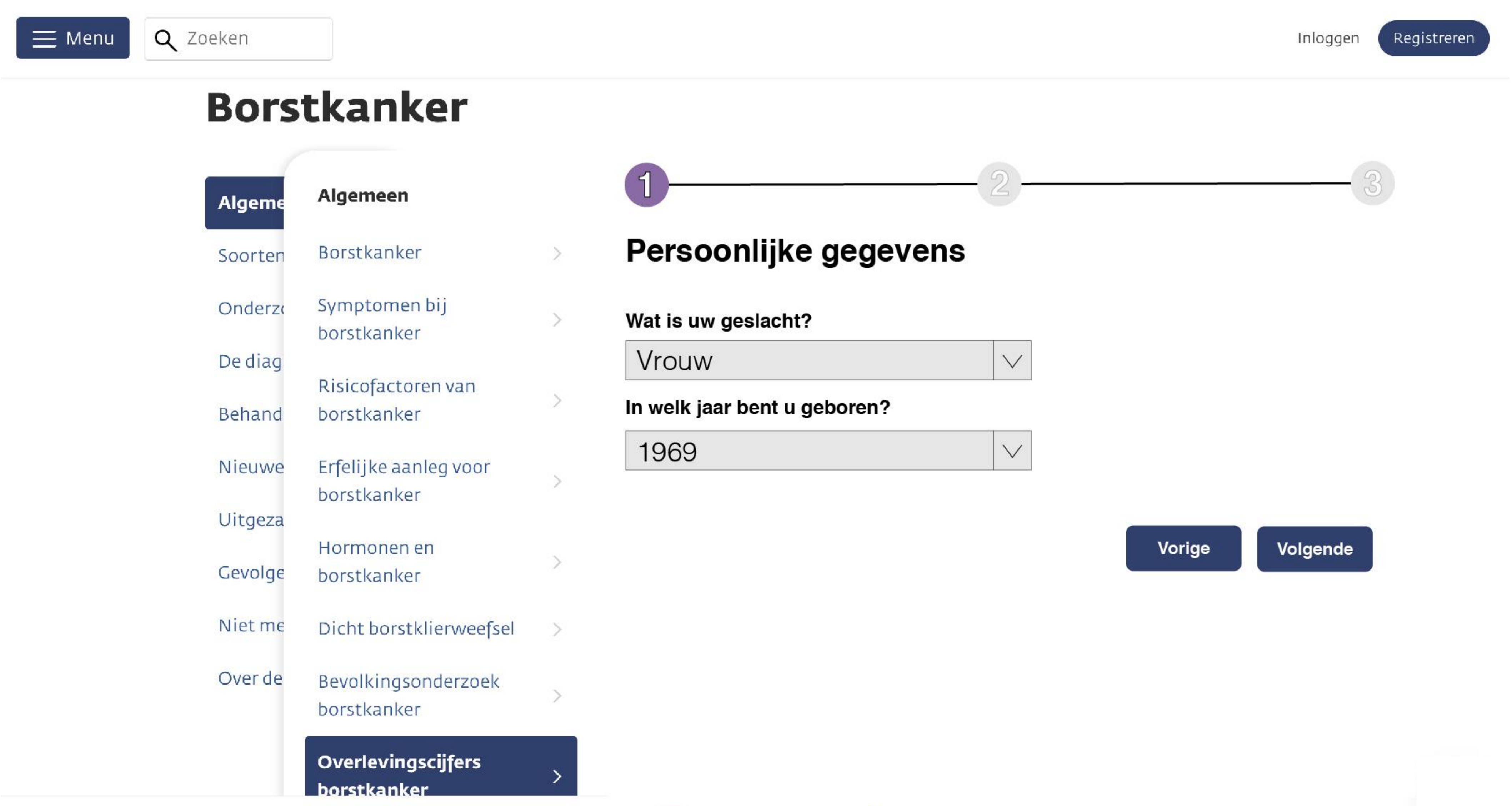

Translation:
Personal data
- What is your gender? (woman)
- What is your year of birth? (1969)

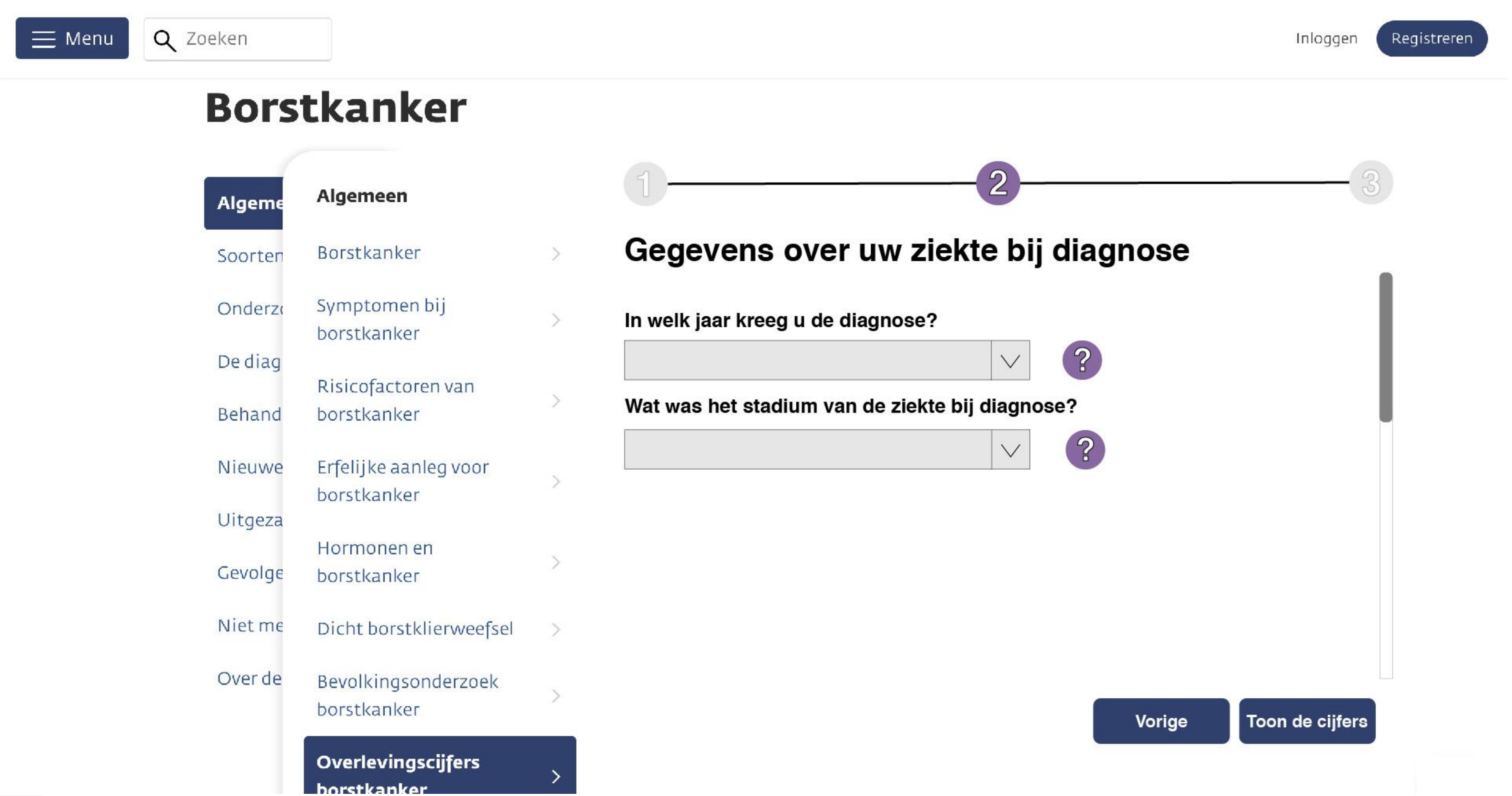

Translation:

Disease-related data

- Year of diagnosis?
- Tumor stage at diagnosis?

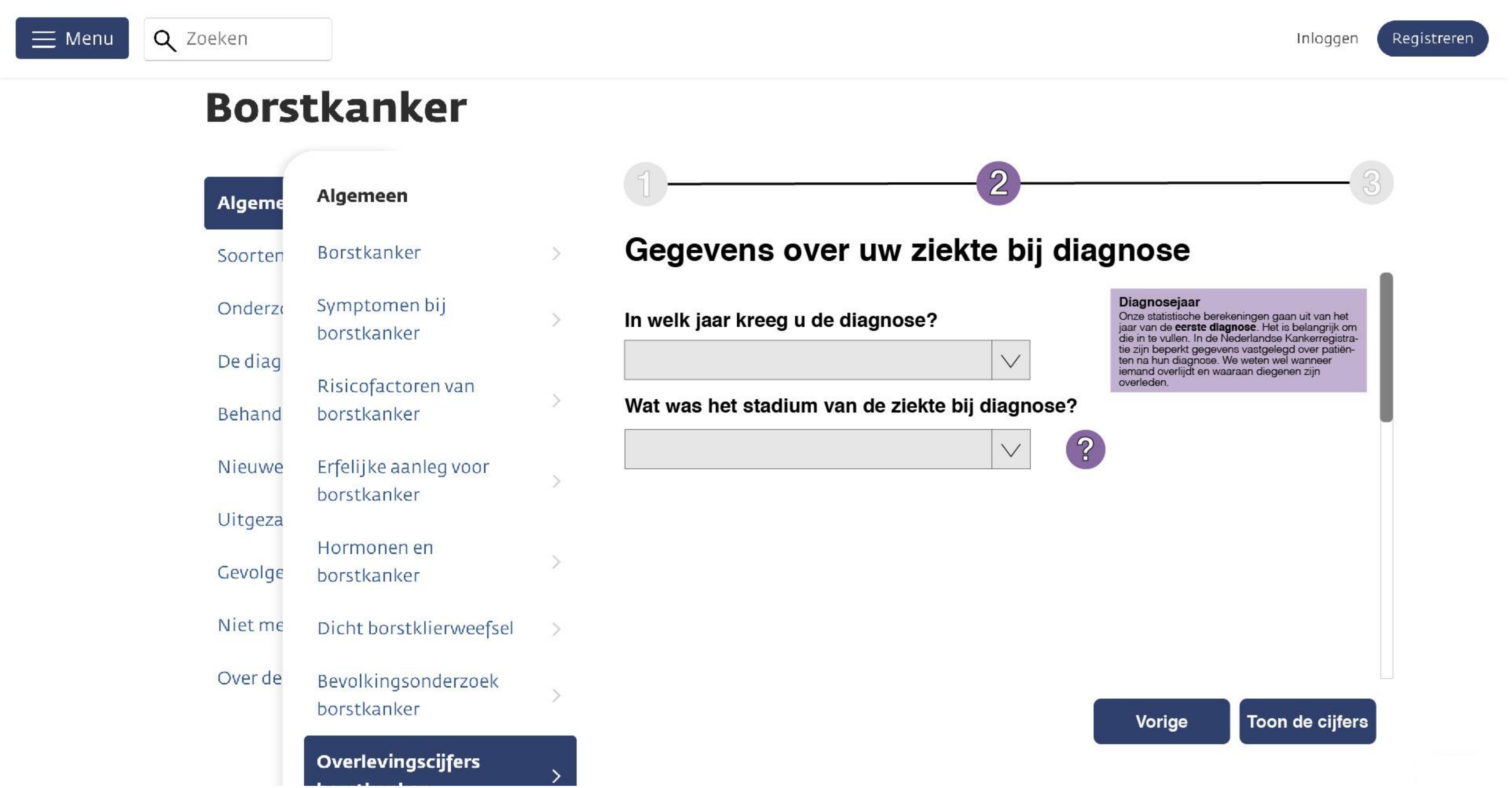

Translation:

Disease-related data

- Year of diagnosis? (Our calculations are based on the first cancer diagnosis. It is important to fill out this one, because the Netherlands Cancer Registry contains limited data of patients after their diagnosis. However, the NCR does know when a patient died (and its cause)).
- Tumor stage at diagnosis?

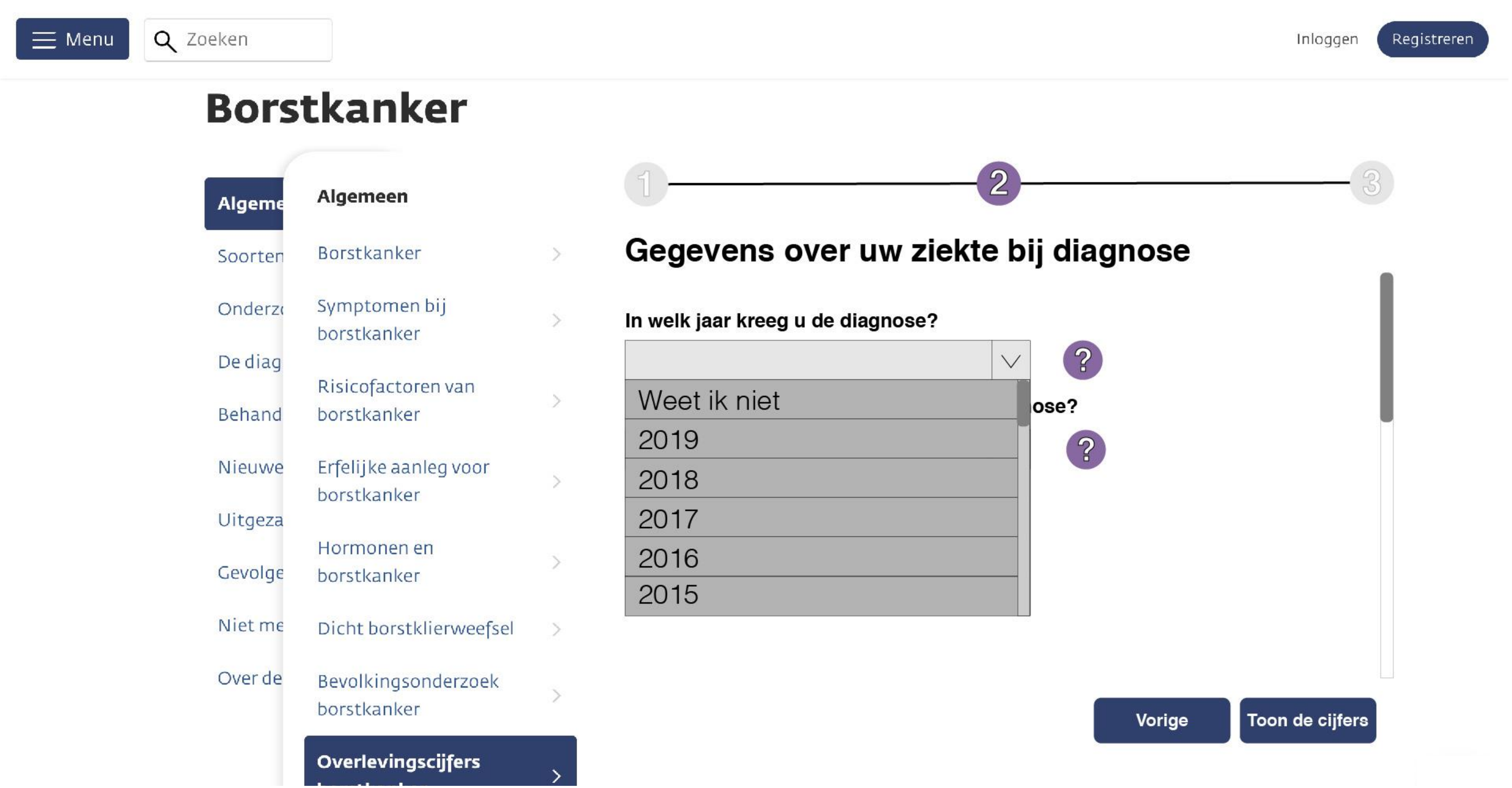

Translation:

Disease-related data

- Year of diagnosis?

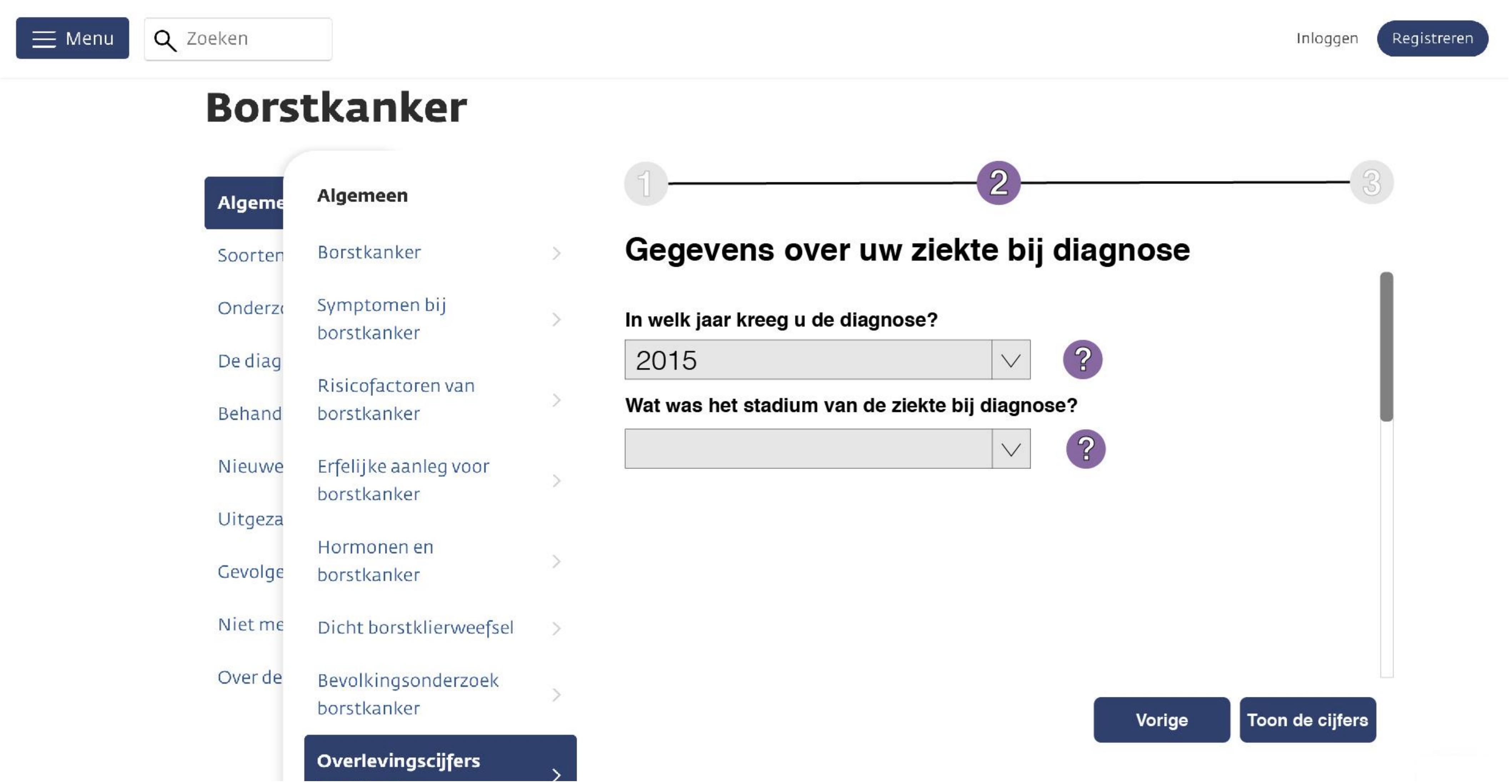

Translation:

Disease-related data

- Year of diagnosis? (2015)
- Tumor stage at diagnosis?

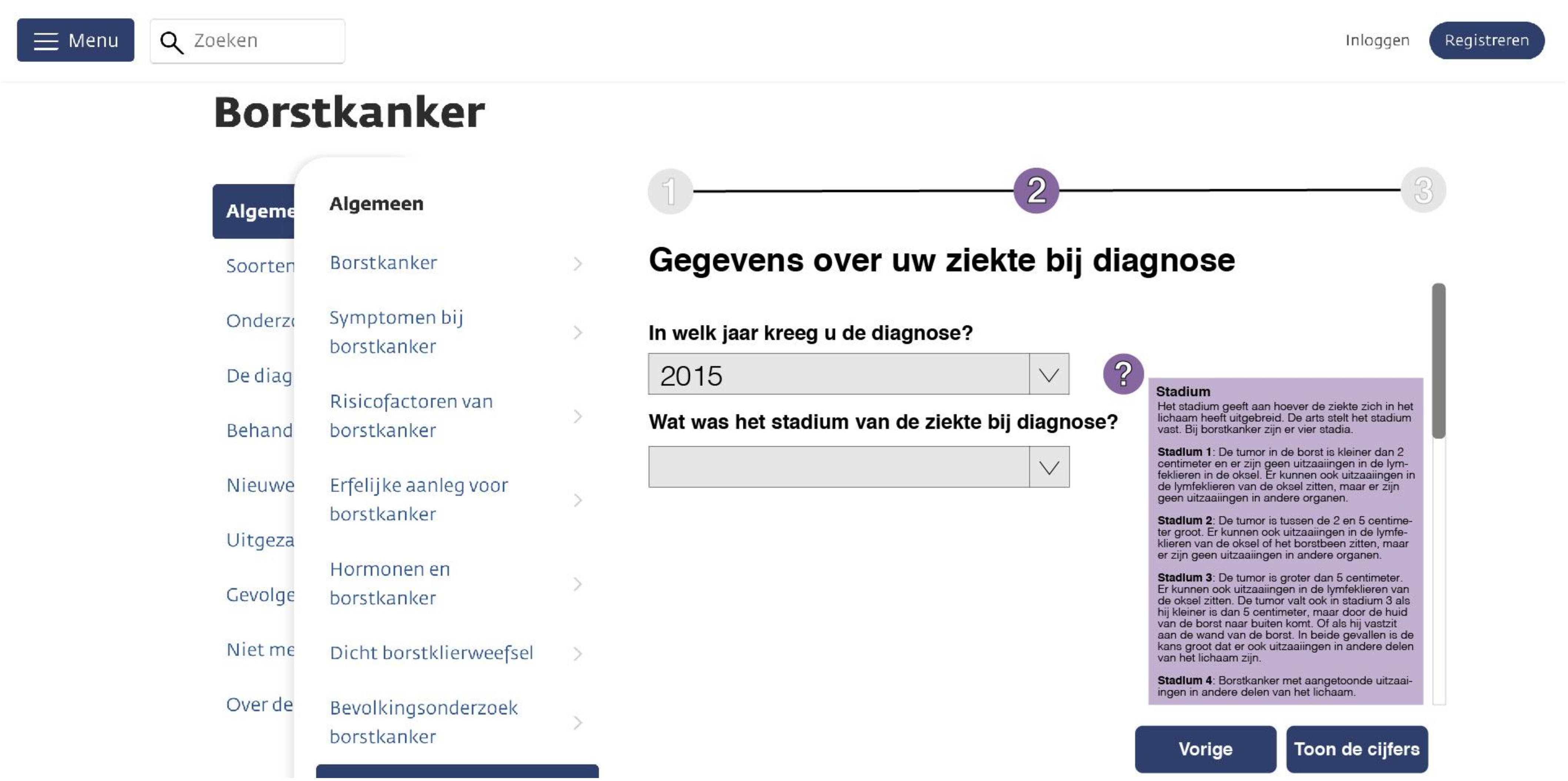

Translation:

Disease-related data

- Year of diagnosis? (2015)
- Tumor stage at diagnosis? (The tumor stage says something about how far the cancer has progressed. The doctor will determine the tumor stage. For breast cancer, there are four stages. **Stage 1:** the tumor is smaller than 2 cm but it has not spread to the nearby lymph nodes or to distant organs. **Stage 2:** the tumor size is between 2 and 5 cm, and may have spread to lymph nodes under the arm or near the breast bone, but not to distant organs. **Stage 3:** The size of the tumor is bigger than 5 cm, and it may have spread to the nearby lymph nodes under the arm. The tumor may also be smaller than 3 cm, but may have spread to the chest wall and/or skin of the breast. **Stage 4:** The tumor has spread beyond the breast and nearby lymph nodes to other organs of the body.

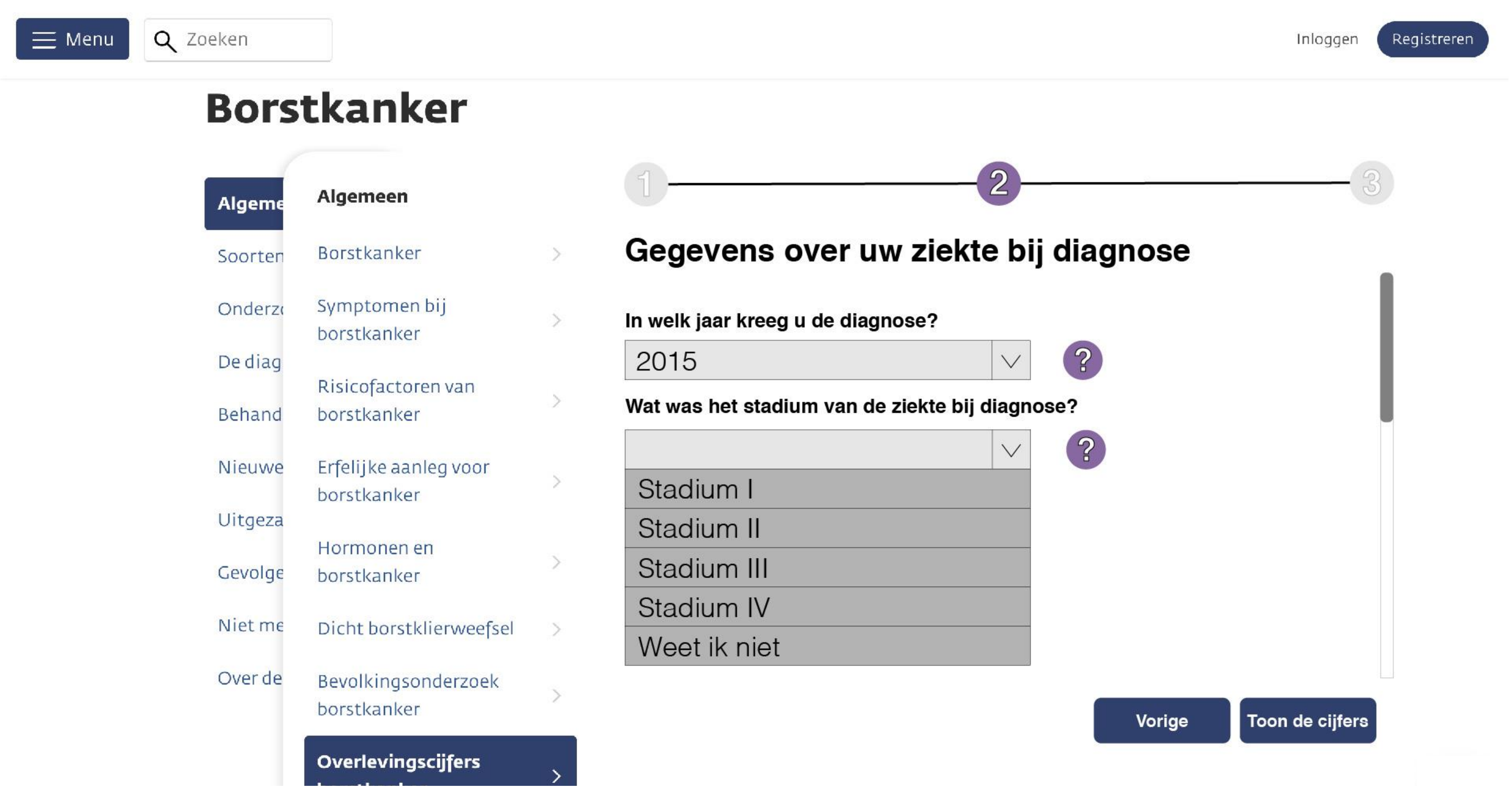

Translation:

Disease-related data

- Year of diagnosis? (2015)
- Tumor stage at diagnosis? (Stage I, Stage II, Stage III, Stage IV)

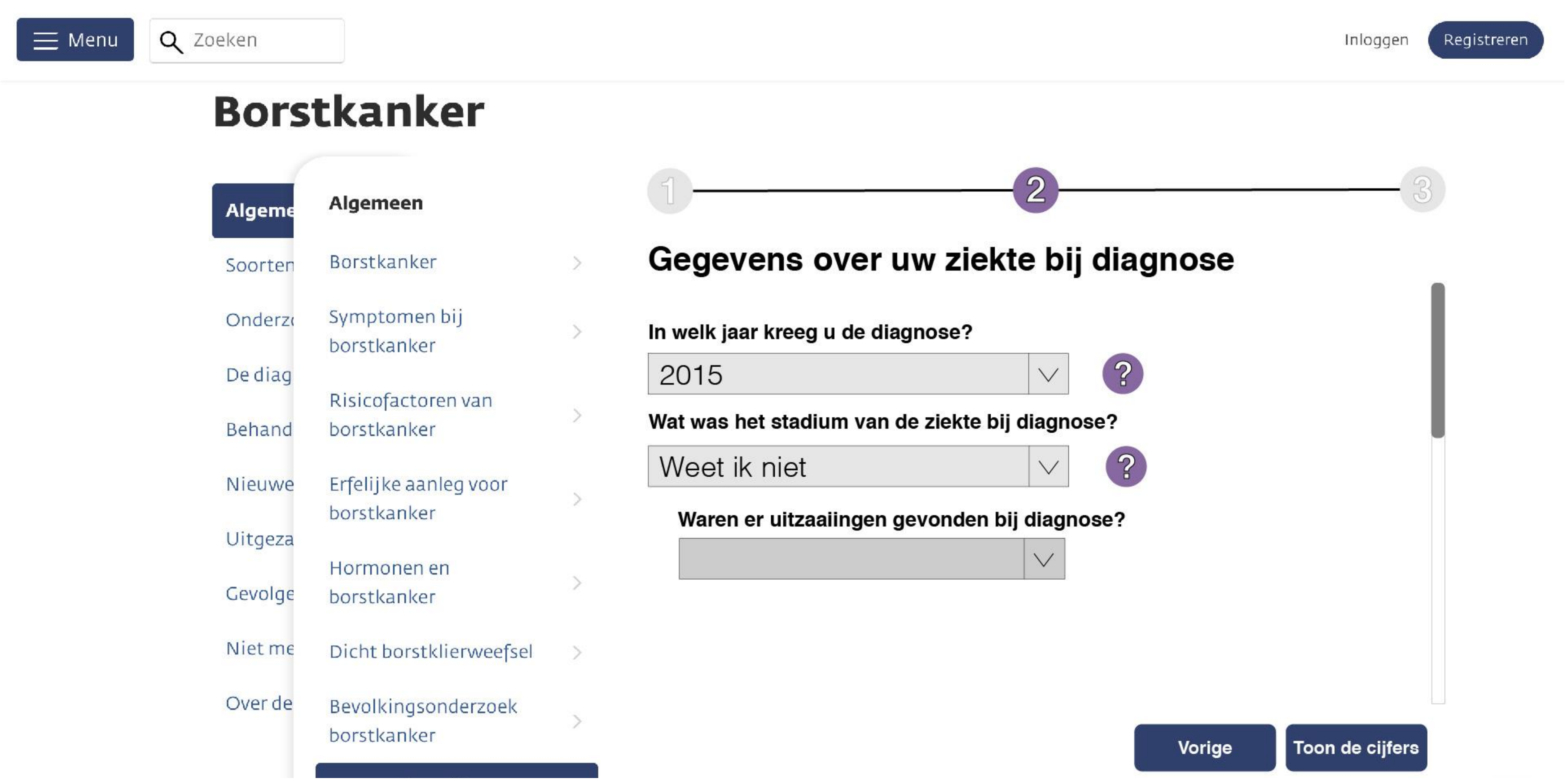

Translation:

Disease-related data

- Year of diagnosis? (2015)
- Tumor stage at diagnosis? (I don't know)
    - Did the tumor spread to any nearby lymph nodes or other parts of the body?

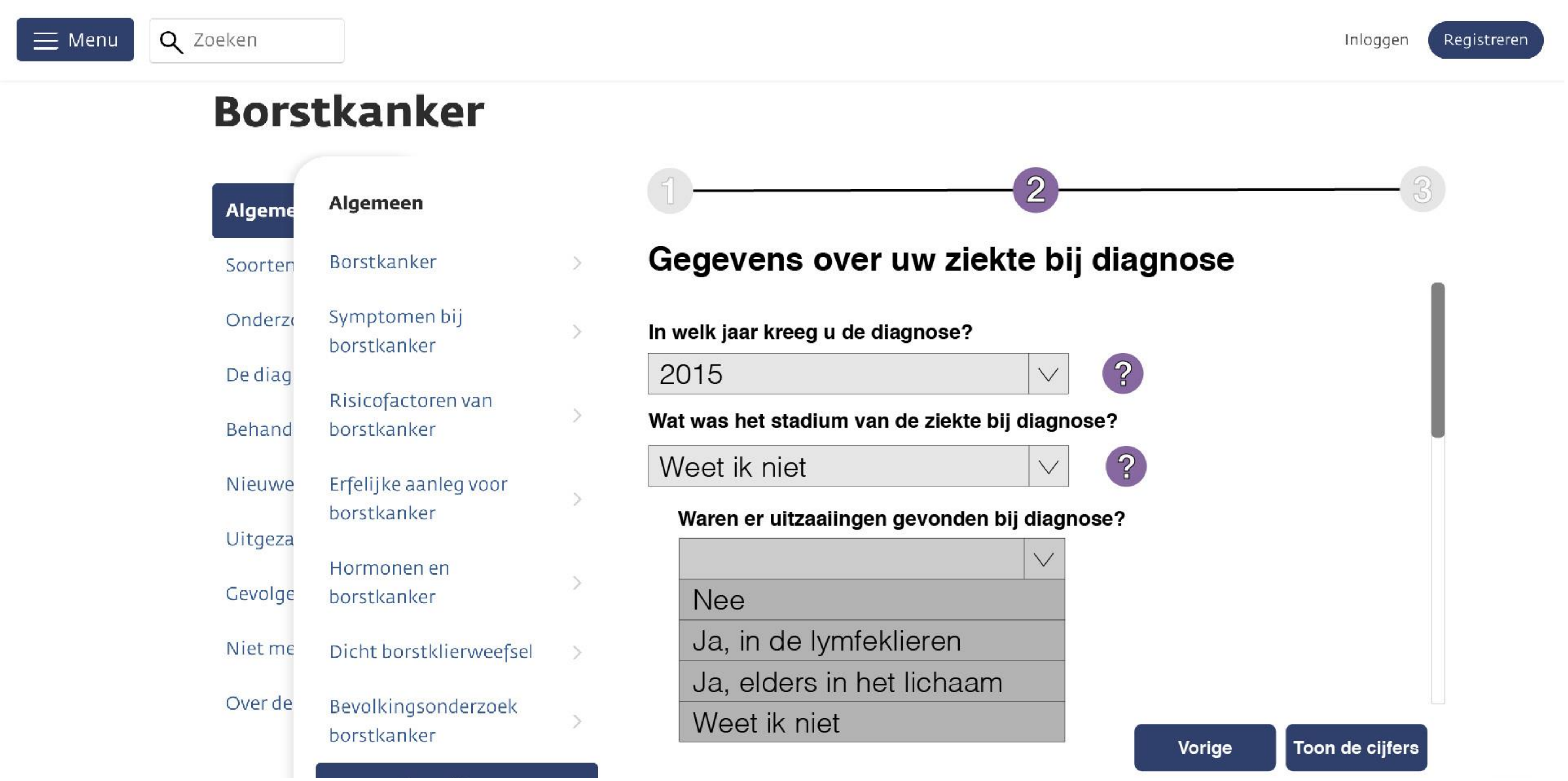

Translation:

Disease-related data

- Year of diagnosis? (2015)
- Tumor stage at diagnosis? (I don't know)
    - Did the tumor spread to any nearby lymph nodes or other parts of the body? (No, yes in the nearby lymph nodes, yes to other parts of the body, I don't know)

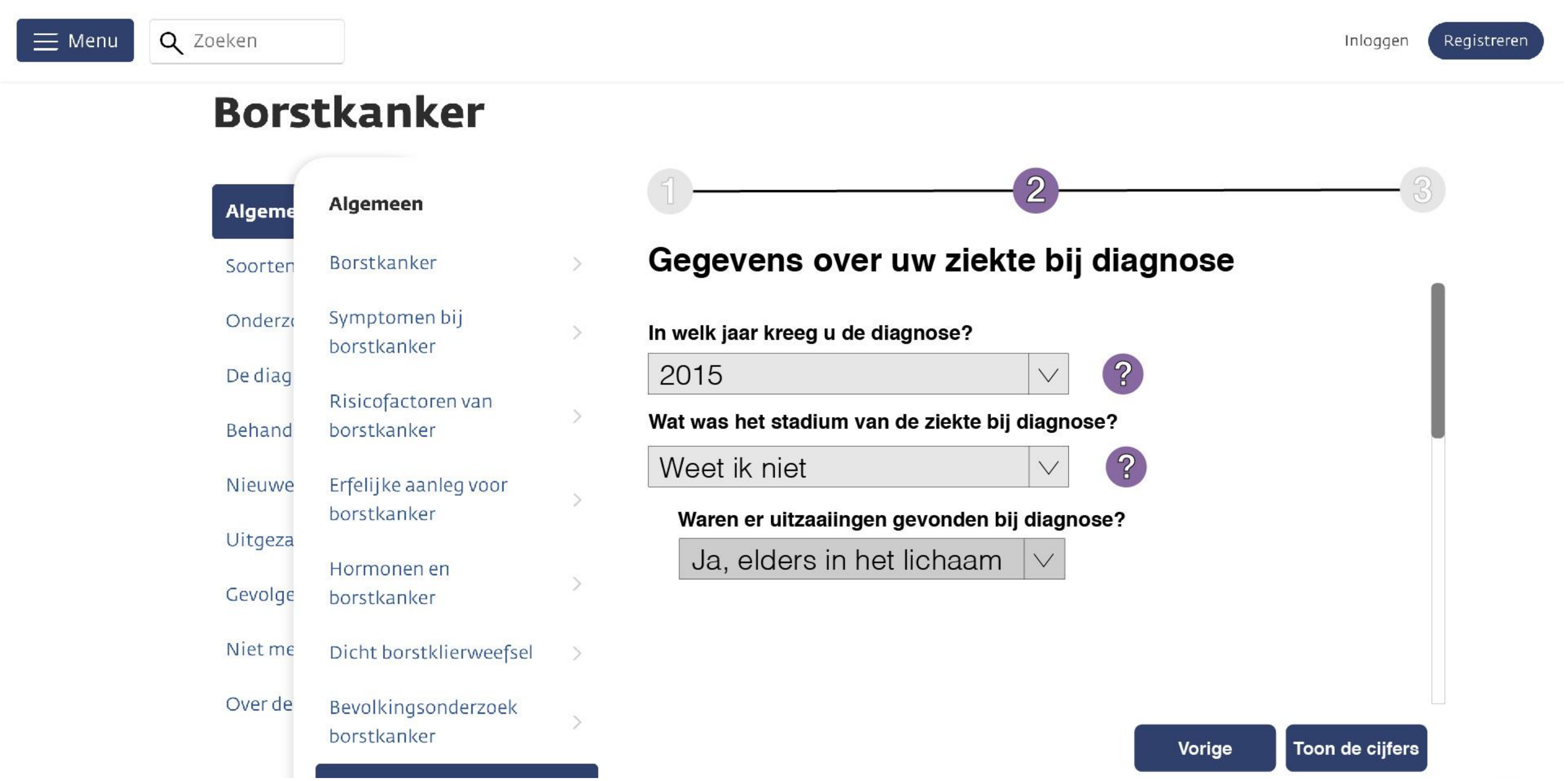

Translation:

Disease-related data

- Year of diagnosis? (2015)
- Tumor stage at diagnosis? (I don’t know)
  - Did the tumor spread to any nearby lymph nodes or other parts of the body? (yes to other parts of the body)

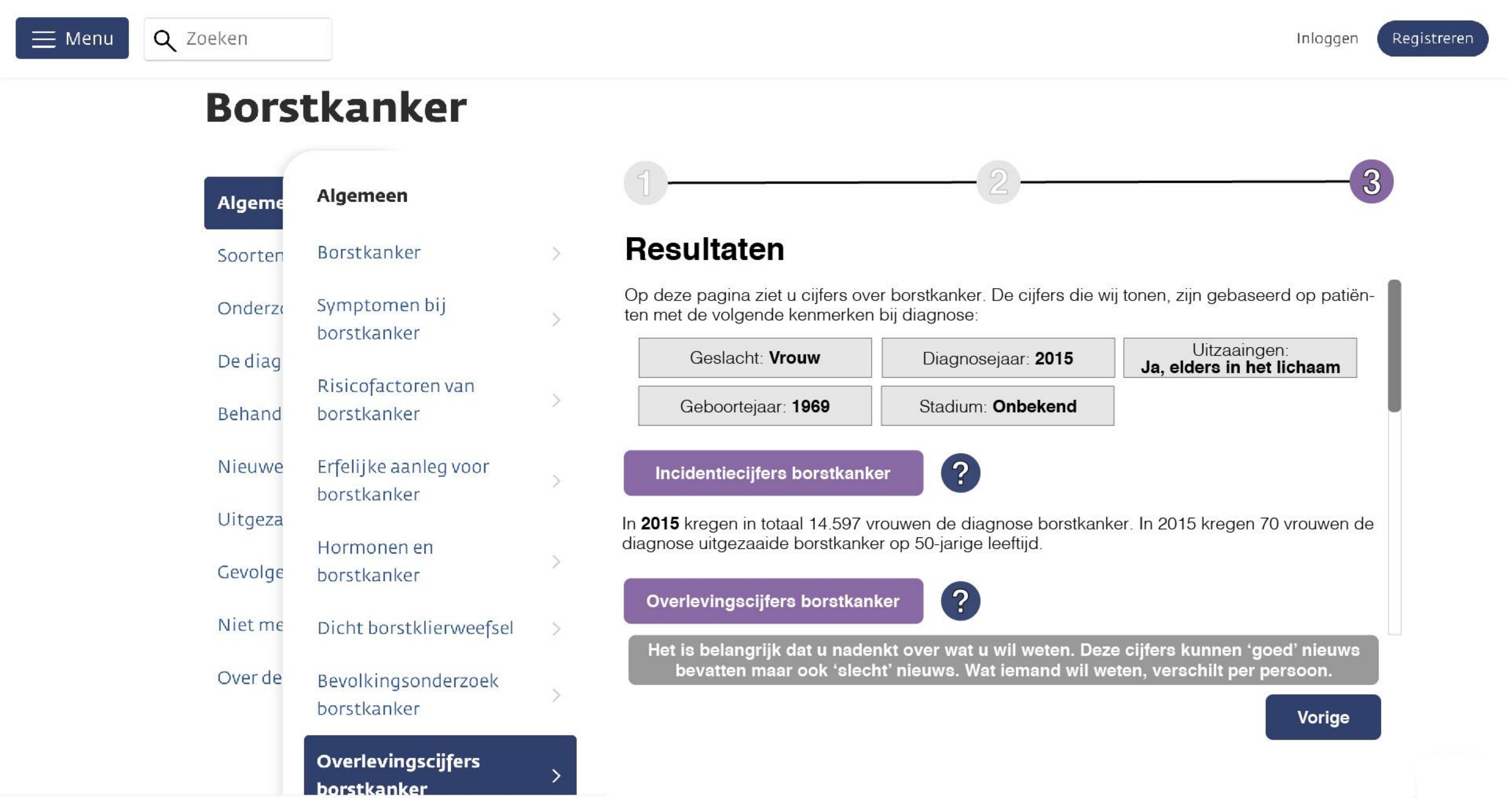

Translation:
Results
On this page, you will see statistics about breast cancer. The statistics that we show are based on patients with the following characteristics:
Incidence rates breast cancer: In 2015, in total 14.597 women were diagnosed with breast cancer. In 2015, 70 women like you were diagnosed with breast cancer that has spread to other parts of the body, aged 50 years old.
Survival statistics breast cancer
It is important that you think about what you want to know. These statistics may contain 'good' or 'bad' new. . What someone wants to know, differs per person.

Menu Zoeken Inloggen Registreren

Borstkanker

Algeme
Soorten
Onderz
De diag
Behand
Nieuwe
Uitgeza
Gevolge
Niet me
Over de

Algemeen

- Borstkanker
- Symptomen bij borstkanker
- Risicofactoren van borstkanker
- Erfelijke aanleg voor borstkanker
- Hormonen en borstkanker
- Dicht borstklierweefsel
- Bevolkingsonderzoek borstkanker
- **Overlevingscijfers borstkanker**

1 — 2 — 3

Resultaten

Op deze pagina ziet u cijfers over borstkanker. De cijfers die wij tonen, zijn gebaseerd op patiënten met de volgende kenmerken bij diagnose:

Geslacht: **Vrouw**	Diagnosejaar: **2015**	Uitzaaingen: **Ja, elders in het lichaam**
Geboortejaar: **1969**	Stadium: **Onbekend**	

Incidentiecijfers borstkanker

Met **incidentie** bedoelen we het aantal nieuwe gevallen met kanker in een bepaalde periode. Bij kanker geven we het aantal mensen dat in een bepaald jaar heeft gehoord dat ze kanker hebben.

In **2015** kregen in totaal 14.597 vrouwen de diagnose borstkanker. In 2015 kregen 70 vrouwen de diagnose uitgezaaide borstkanker op 50-jarige leeftijd.

Het is belangrijk dat u nadenkt over wat u wil weten. Deze cijfers kunnen 'goed' nieuws bevatten maar ook 'slecht' nieuws. Wat iemand wil weten, verschilt per persoon.

Vorige

Translation:
With incidence rate we mean the number of new cancers of cancer occurring in a specified population in a certain period (typically in a specific year).

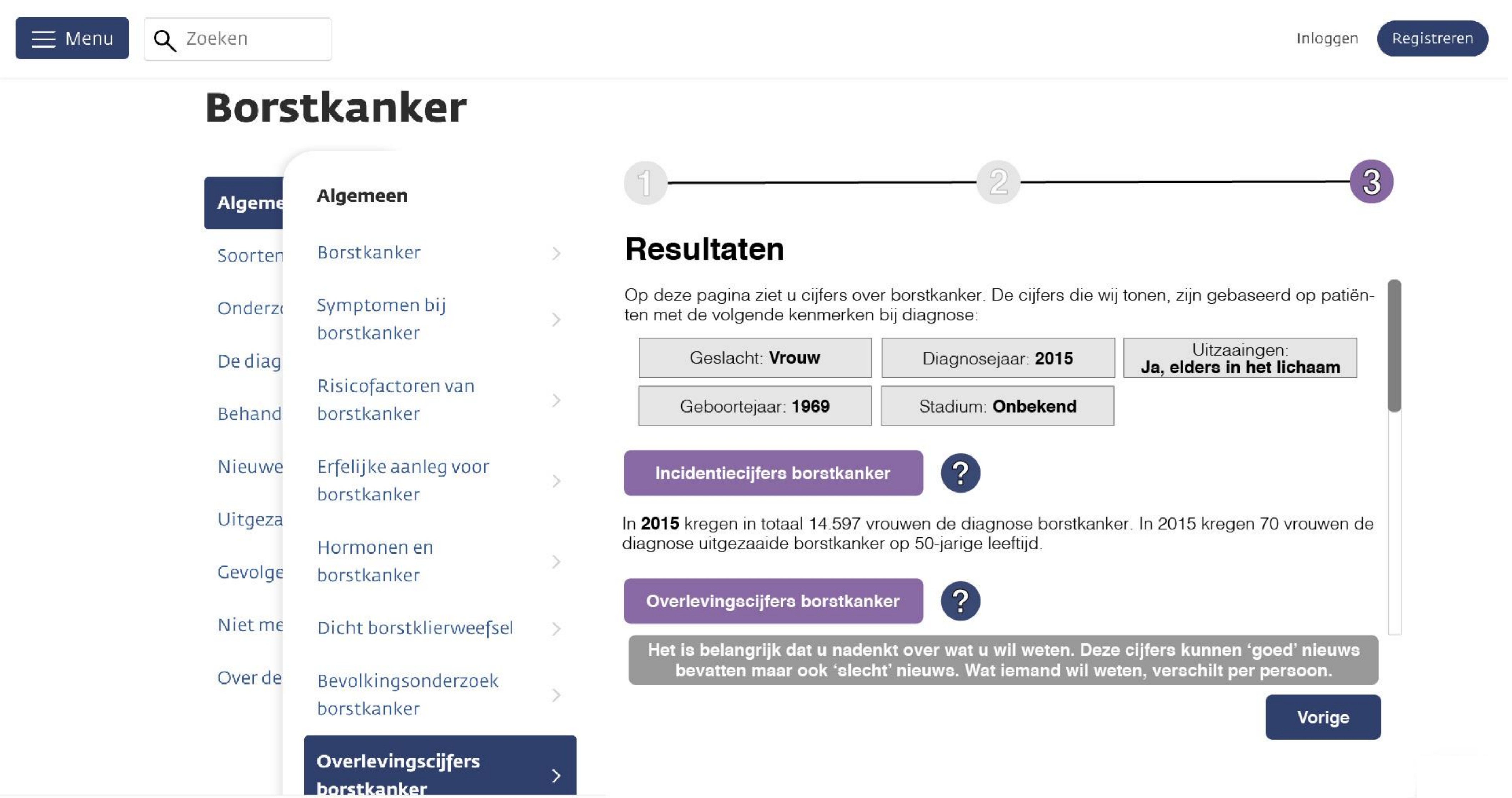

Translation:

Results

On this page, you will see statistics about breast cancer. The statistics that we show are based on patients with the following characteristics:

Incidence rates breast cancer: In 2015, in total 14.597 women were diagnosed with breast cancer. In 2015, 70 women like you were diagnosed with breast cancer that has spread to other parts of the body, aged 50 years old.

Survival statistics breast cancer

It is important that you think about what you want to know. These statistics may contain 'good' or 'bad' new. . What someone wants to know, differs per person.

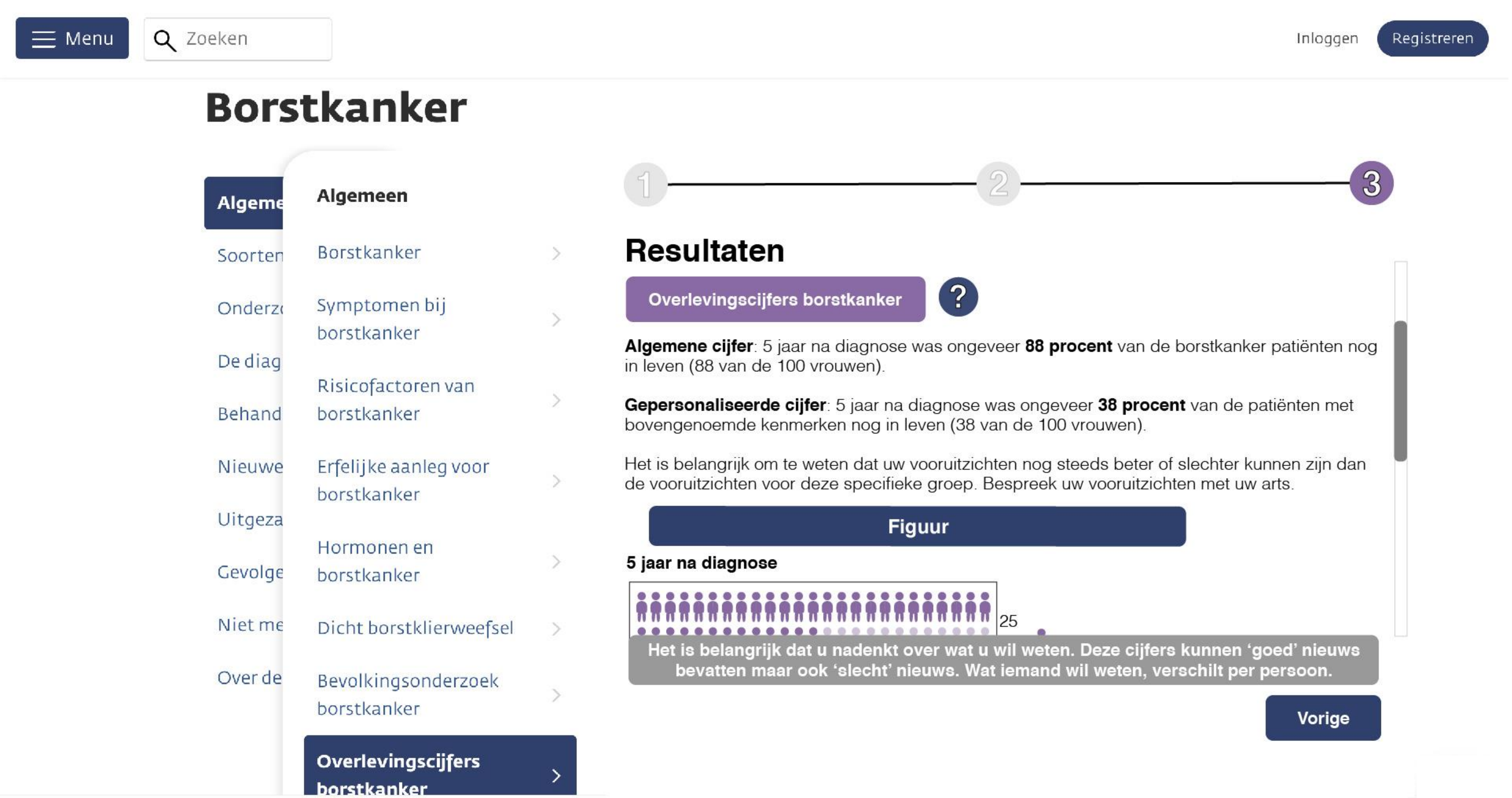

Translation:

Survival statistics breast cancer

Generic statistic: 5 years after diagnosis, about **88 percent** of these patients with breast cancer are alive (88 out of 100 women).

Personalized statistic: 5 years after diagnosis, about **38 percent** of these patients like you with breast cancer are alive (38 out of 100 women).

It is important to know that your prognosis may be better or worse than the prognosis of this specific group of patients. Please discuss your prognostic situation with your doctor.

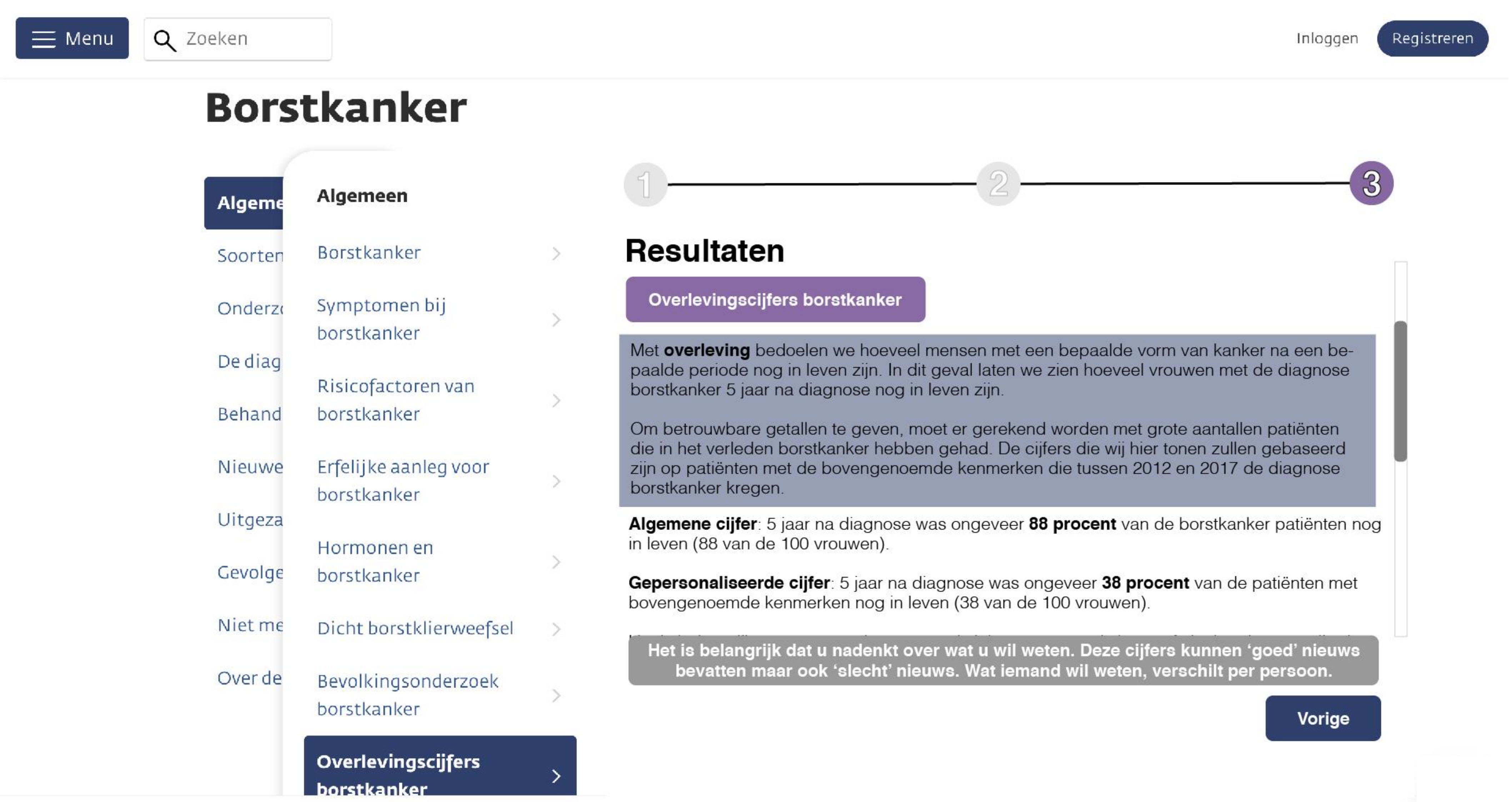

Translation:
With survival we mean the percentage of people with cancer still alive after a particular amount of time. Survival statistics for cancer are usually written as 5 year survival.
In order to provide you with reliable statistics we need to perform statistics on a large group of patients that have been diagnosed with breast cancer in the past. The statistics that we show here are performed on data from patients diagnosed between 2012 and 2017.

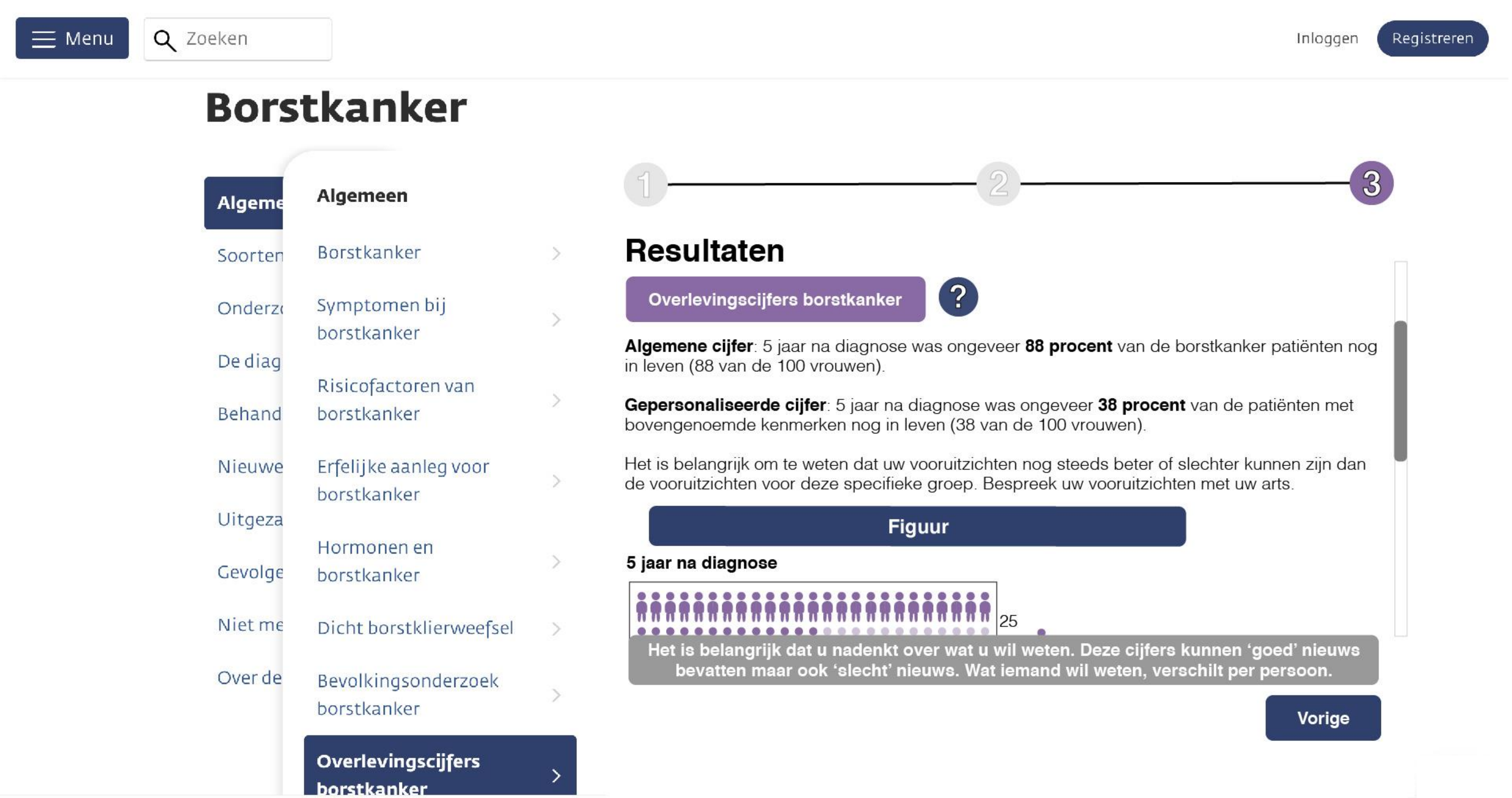

Translation:

Survival statistics breast cancer

Generic statistic: 5 years after diagnosis, about **88 percent** of these patients with breast cancer are alive (88 out of 100 women).

Personalized statistic: 5 years after diagnosis, about **38 percent** of these patients like you with breast cancer are alive (38 out of 100 women).

It is important to know that your prognosis may be better or worse than the prognosis of this specific group of patients. Please discuss your prognostic situation with your doctor.

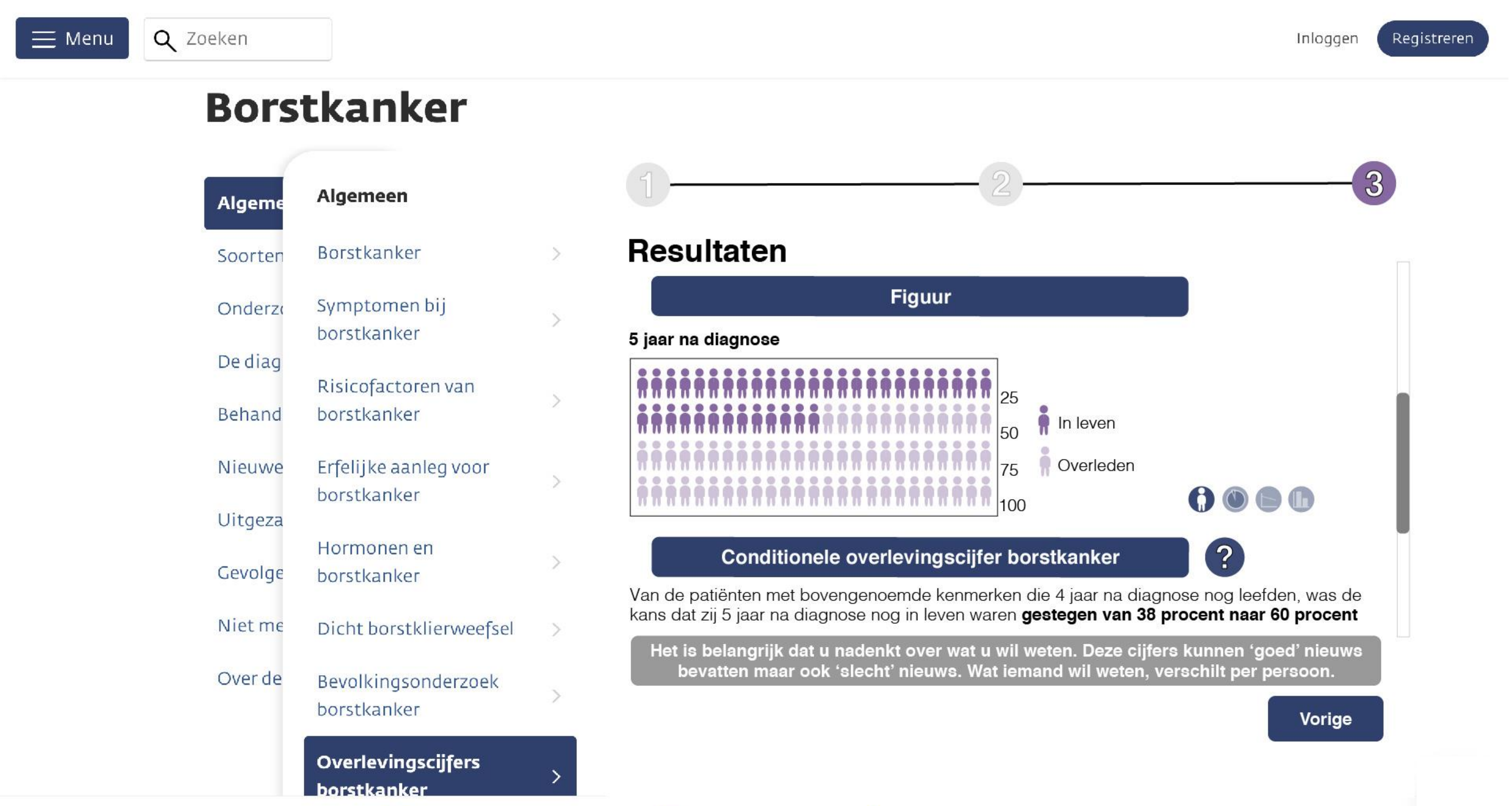

Translation:

Conditional survival statistics breast cancer

Of those patients like you who were still alive 4 years after diagnosis, the chance that they would still be alive 5 years after diagnosis increased from **38 percent to 60 percent** (60 out of 100 women).

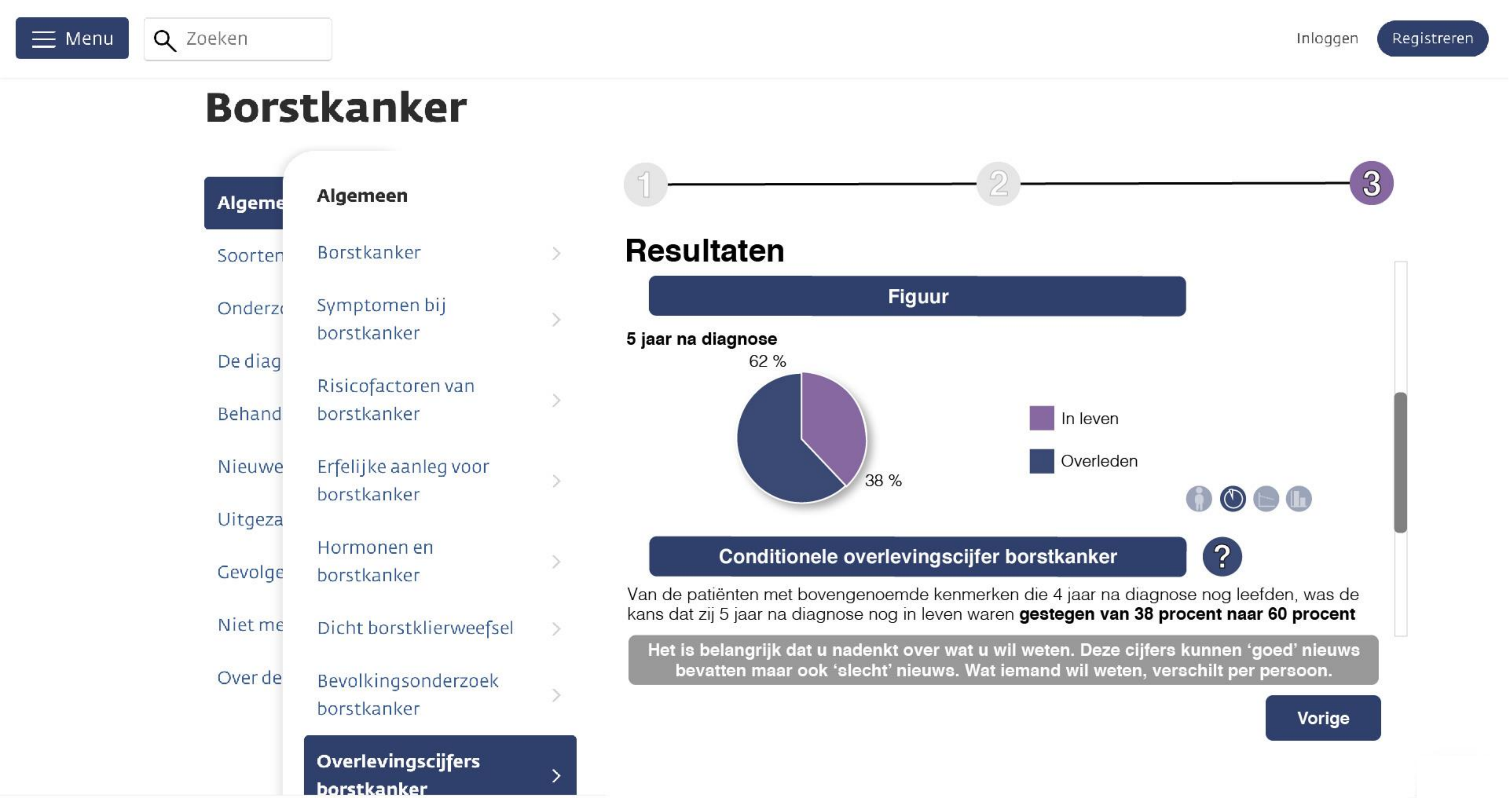

Translation:

Conditional survival statistics breast cancer

Of those patients like you who were still alive 4 years after diagnosis, the chance that they would still be alive 5 years after diagnosis increased from **38 percent to 60 percent** (60 out of 100 women).

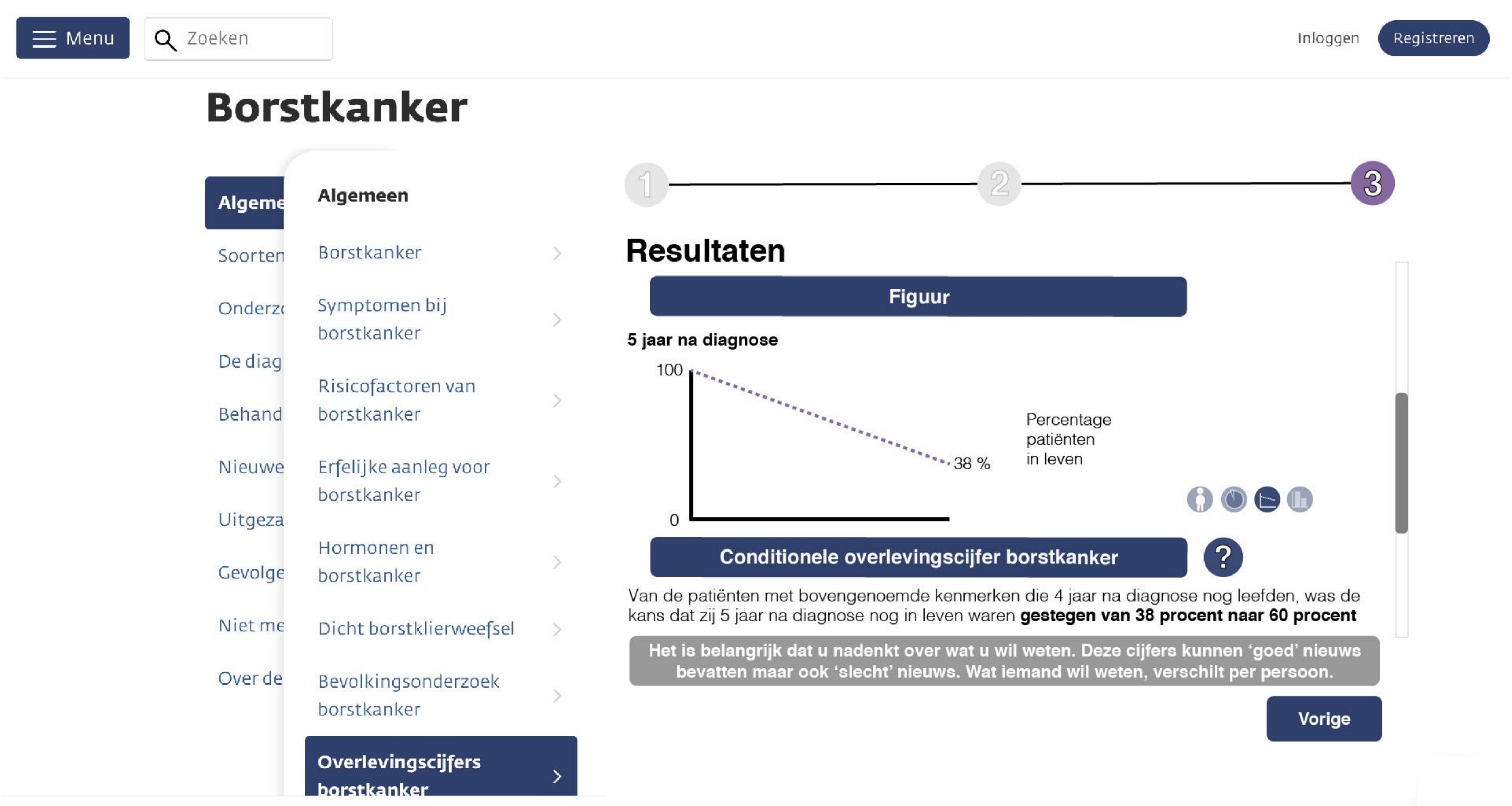

Translation:

Conditional survival statistics breast cancer

Of those patients like you who were still alive 4 years after diagnosis, the chance that they would still be alive 5 years after diagnosis increased from **38 percent to 60 percent** (60 out of 100 women).

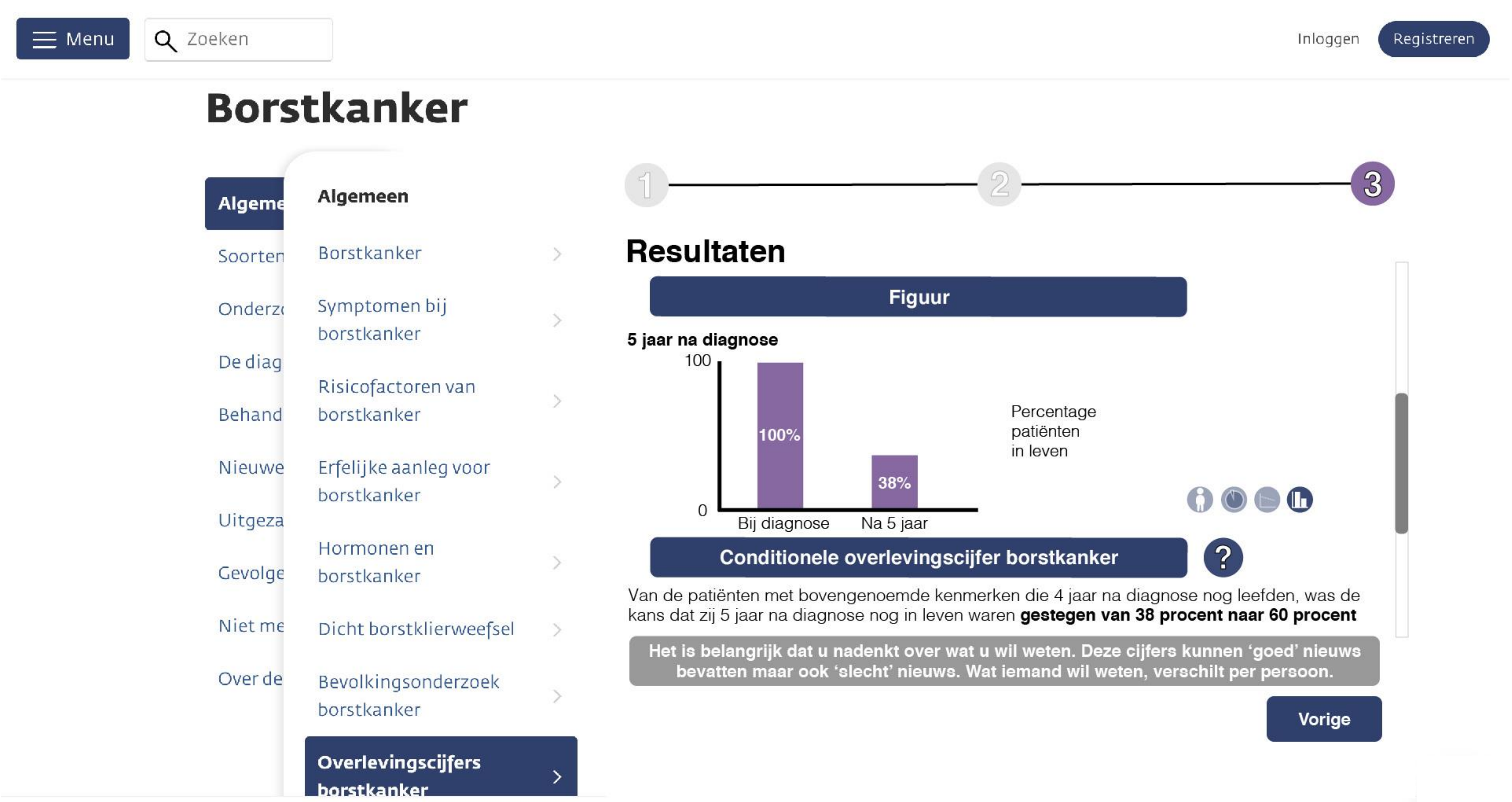

Translation:

Conditional survival statistics breast cancer

Of those patients like you who were still alive 4 years after diagnosis, the chance that they would still be alive 5 years after diagnosis increased from **38 percent to 60 percent** (60 out of 100 women).

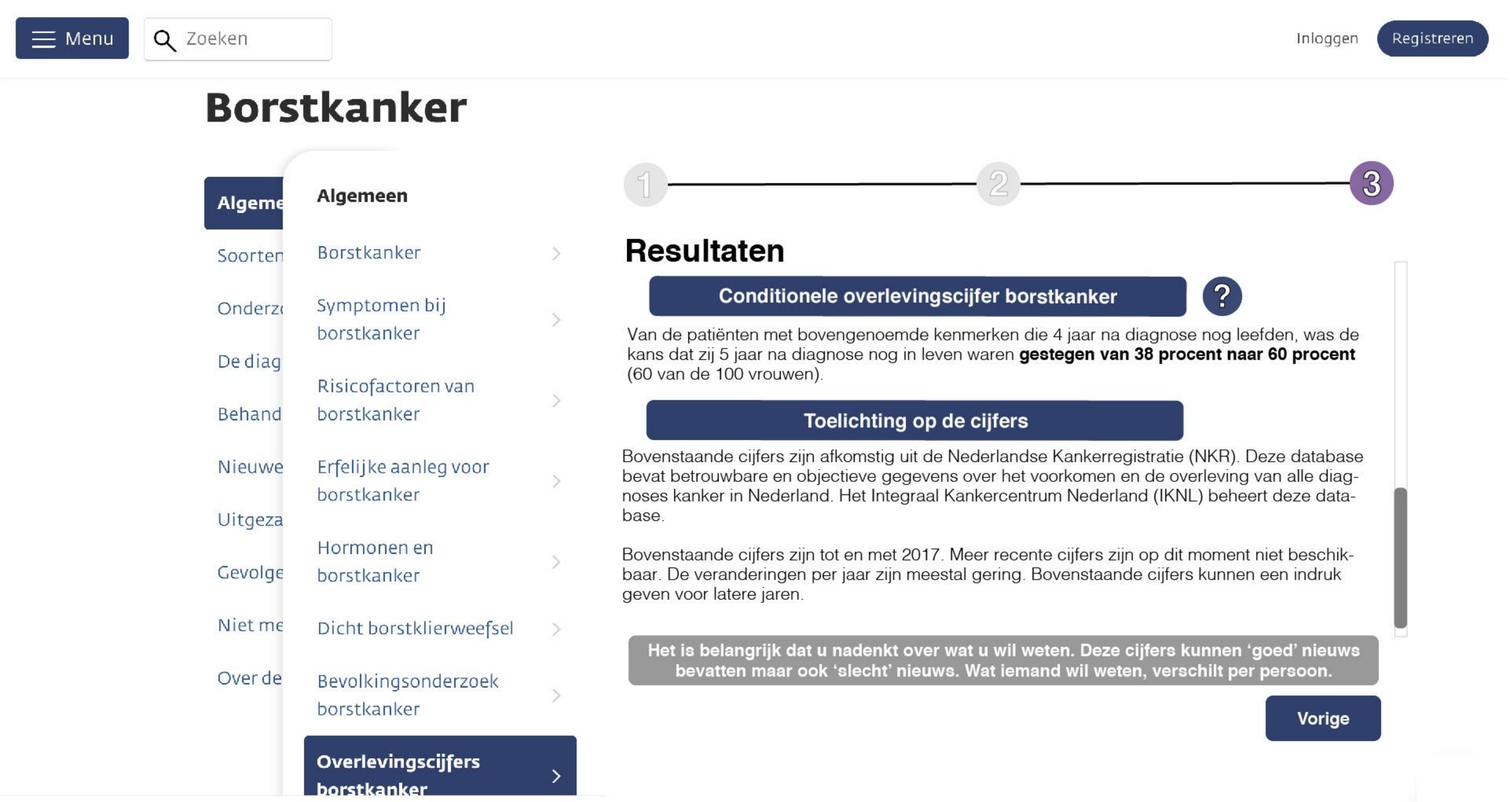

Translation:
About the statistics
The statistics that we show are based on data from the Netherlands Cancer Registry (NCR). This database contains reliable and objective data about the prevalence and survival of all cancer patients in the Netherlands. The Netherlands Comprehensive Cancer Organisation hosts this database.
The statistics that we showed are until 2017. More recent statistics are currently not available. However, annual changes are typically limited. The statistics provided here may therefore give an impression for future years.

Menu Zoeken Inloggen Registreren

Borstkanker

Algemeen

Soorten

Onderz

De diag

Behand

Nieuwe

Uitgeza

Gevolge

Niet me

Over de

Algemeen

- Borstkanker
- Symptomen bij borstkanker
- Risicofactoren van borstkanker
- Erfelijke aanleg voor borstkanker
- Hormonen en borstkanker
- Dicht borstklierweefsel
- Bevolkingsonderzoek borstkanker
- **Overlevingscijfers borstkanker**

1 2 3

Resultaten

Conditionele overlevingscijfer borstkanker

Veel kankerpatiënten die hun ziekte reeds enige tijd hebben overleefd, willen graag weten wat hun vooruitzichten zijn gelet op het feit dat ze nog (steeds) in leven zijn. Dit wordt 'conditionele overleving' genoemd. De overlevingscijfers worden vaak beter met ieder jaar dat de patiënt overleeft.

Van de patiënten met bovengenoemde kenmerken die 4 jaar na diagnose nog leefden, was de kans dat zij 5 jaar na diagnose nog in leven waren **gestegen van 38 procent naar 60 procent** (60 van de 100 vrouwen).

Toelichting op de cijfers

Bovenstaande cijfers zijn afkomstig uit de Nederlandse Kankerregistratie (NKR). Deze database bevat betrouwbare en objectieve gegevens over het voorkomen en de overleving van alle diagnoses kanker in Nederland. Het Integraal Kankercentrum Nederland (IKNL) beheert deze database.

Het is belangrijk dat u nadenkt over wat u wil weten. Deze cijfers kunnen 'goed' nieuws bevatten maar ook 'slecht' nieuws. Wat iemand wil weten, verschilt per persoon.

Vorige

Translation:
Many patients with cancer who have survived for a couple of years want to know the chance that they will survive another x years (given that they are still alive). We call this conditional survival. In general, for cancer patients who have already survived a number of years, survival expectations at diagnosis are typically more favorable.

Menu Zoeken Inloggen Registreren

Borstkanker

Algeme
Soorten
Onderz
De diag
Behand
Nieuwe
Uitgeza
Gevolge
Niet me
Over de

Algemeen

Borstkanker

Symptomen bij borstkanker

Risicofactoren van borstkanker

Erfelijke aanleg voor borstkanker

Hormonen en borstkanker

Dicht borstklierweefsel

Bevolkingsonderzoek borstkanker

Overlevingscijfers borstkanker

1 2 3

Resultaten

Conditionele overlevingscijfer borstkanker ?

Van de patiënten met bovengenoemde kenmerken die 4 jaar na diagnose nog leefden, was de kans dat zij 5 jaar na diagnose nog in leven waren **gestegen van 38 procent naar 60 procent** (60 van de 100 vrouwen).

Toelichting op de cijfers

Bovenstaande cijfers zijn afkomstig uit de Nederlandse Kankerregistratie (NKR). Deze database bevat betrouwbare en objectieve gegevens over het voorkomen en de overleving van alle diagnoses kanker in Nederland. Het Integraal Kankercentrum Nederland (IKNL) beheert deze database.

Bovenstaande cijfers zijn tot en met 2017. Meer recente cijfers zijn op dit moment niet beschikbaar. De veranderingen per jaar zijn meestal gering. Bovenstaande cijfers kunnen een indruk geven voor latere jaren.

Het is belangrijk dat u nadenkt over wat u wil weten. Deze cijfers kunnen ‘goed’ nieuws bevatten maar ook ‘slecht’ nieuws. Wat iemand wil weten, verschilt per persoon.

Vorige
