# Supplementary material for: Exploring Cancer Survivor Needs and Preferences for Communicating Personalized Cancer Statistics From Registry Data: Qualitative Multimethod Study
Source: JMIR Cancer. 2021 Oct 25;7(4):e25659. doi: 10.2196/25659 (PMC8576563; doi:10.2196/25659)
Supplement: Multimedia Appendix 4 [file cancer_v7i4e25659_app4.pdf]

Multimedia 4

Materials: Topic guide semi-structured interviews
Study 2: Think-aloud observations and semi-structured interviews
Authors: Vromans et al.

Description: After each think-aloud session, we conducted a semi-structured interview to provide participants the opportunity to elaborate on statements made during the think-aloud sessions, and to further capture participant preferences for communicating the statistics. Note that not all questions were asked during the interview, as some participants may have answered them during the think-aloud sessions.

Categories:

- Content
- Presentation
- Emotions
- Other / Depending on statements made during the think-aloud sessions

Part	Question	Category
**1. Introduction and supporting statements**	Was the general goal of the tool clear to you?<br>Do you think the starting page contains enough information?	Content
	What did you feel while reading the supporting statements?	Emotions
	Was it clear to you why these supporting statements were shown to you?	Content
	Could you tell me how you felt while reading the supporting statements?	Emotions
**2. Patient data entry**	What was your general impression about the patient data entry page?<br>• While thinking aloud, you mentioned … Could you elaborate on this?	Other
	Was it clear to you why you should enter personal characteristics?	Content
	Was it easy/difficult for you to enter the personal characteristics?	Presentation
	Could you tell me how you felt while entering the personal characteristics?	Emotions
	What did/didn't you like about this page?<br>• While thinking aloud, you mentioned … Could you elaborate on this?	Other

	Do you think the textual information was understandable to you?
**3. Tumor data entry**	What was your general impression about the tumor data entry page?<br><br>Was it clear to you why you should enter tumor characteristics?<br><br>Was it easy/difficult for you to enter the tumor characteristics?<br><br>Could you tell me how you felt while entering the tumor characteristics?<br><br>What did/didn't you like about this page?<br><br>• While thinking aloud, you mentioned … Could you elaborate on this?<br><br>Do you think the textual information was understandable to you?
**4. Output display**	What was your general impression about the results page?<br><br>Could you tell me how you felt while seeing the results?<br><br>What did/didn't you like about this page?<br><br>• While thinking aloud, you mentioned … Could you elaborate on this?<br><br>Do you think the textual information was understandable to you?<br><br>Do you think the figures on this page were understandable to you?<br><br>What do you think of the option to change the type of figure?
**5. Preference for presentation format**	Do you have a preference for a type of figure?<br><br>• Could you explain why?<br><br>Do you have a preference for the amount of information presented in the tool? Do you prefer the short and concise version, or the long and detailed version?<br><br>• Could you explain why?

	Do you prefer to see the information all at once, or to tailor the amount of information and expand texts for certain topics.<br>• Could you explain why?		
**6. Intention to use**	If this tool will become available online, will you make use of it?<br>• Could you explain why?		
**7. Other remarks**	Do you have any other remarks or comments?<br>While thinking aloud, you also mentioned … What did you mean with this?		
